# Evaluation of the Genetic Response of U937 and Jurkat Cells to 10-Nanosecond Electrical Pulses (nsEP)

**DOI:** 10.1371/journal.pone.0154555

**Published:** 2016-05-02

**Authors:** Caleb C. Roth, Randolph D. Glickman, Gleb P. Tolstykh, Larry E. Estlack, Erick K. Moen, Ibtissam Echchgadda, Hope T. Beier, Ronald A. Barnes, Bennett L. Ibey

**Affiliations:** 1 Department of Radiological Sciences, University of Texas Health Science Center San Antonio, San Antonio, Texas 78229, United States of America; 2 General Dynamics IT, JBSA Fort Sam Houston, San Antonio, Texas 78234, United States of America; 3 Department of Ophthalmology, University of Texas Health Science Center San Antonio, San Antonio, Texas 78229, United States of America; 4 Department of Electrical Engineering, University of Southern California, Los Angeles, California, 90089, United States of America; 5 Bioeffects Division, Air Force Research Laboratory, Radiofrequency Branch, JBSA Fort Sam Houston, San Antonio, Texas 78234, United States of America; 6 Bioeffects Division, Air Force Research Laboratory, Optical Radiation Branch, JBSA Fort Sam Houston, San Antonio, Texas 78234, United States of America; 7 National Research Council Research Associateship Program, JBSA Fort Sam Houston, San Antonio, Texas 78234, United States of America; Medical College of Georgia, UNITED STATES

## Abstract

Nanosecond electrical pulse (nsEP) exposure activates signaling pathways, produces oxidative stress, stimulates hormone secretion, causes cell swelling and induces apoptotic and necrotic death. The underlying biophysical connection(s) between these diverse cellular reactions and nsEP has yet to be elucidated. Using global genetic analysis, we evaluated how two commonly studied cell types, U937 and Jurkat, respond to nsEP exposure. We hypothesized that by studying the genetic response of the cells following exposure, we would gain direct insight into the stresses experienced by the cell and in turn better understand the biophysical interaction taking place during the exposure. Using Ingenuity Systems software, we found genes associated with cell growth, movement and development to be significantly up-regulated in both cell types 4 h post exposure to nsEP. In agreement with our hypothesis, we also found that both cell lines exhibit significant biological changes consistent with mechanical stress induction. These results advance nsEP research by providing strong evidence that the interaction of nsEPs with cells involves mechanical stress.

## Introduction

Cell exposure to high intensity millisecond and microsecond electrical pulses (electroporation) is theorized to cause the formation of membrane pores. These “electro-pores” allow for the transfer of genetic and proteomic material, drugs and chemicals into a cell, for the purpose of inducing a biochemical change [1–6]. Thus, electroporation is a very useful tool for molecular biological research and as such is widely used in many laboratories. Despite the widespread use of electroporation, very little is known how pulsed electric fields in general, affect the molecular processes of cells, especially those associated with gene expression. Our laboratory studies a specific type of electroporation that utilizes nanosecond duration pulses (referred to hereafter as nanosecond electrical pulses or nsEP). The nsEP induced events include swelling [7,8], blebbing [7,8], phospholipid translocation [9,10], prolonged membrane permeablization (nanoporation) [11–13], apoptosis [7,14–17], and necrosis [7,14]. Despite this wealth of evidence, much remains unknown about how a cell reacts genetically to nsEP-delivered stress.

Events associated with nsEP exposure that can cause changes in gene expression have been identified. Using high speed imaging, Beier et al. observed a rapid increase in intracellular calcium originating from membrane regions closest to the electrodes, illustrating a unique directionality to the nsEP response [18]. In agreement with previous studies, they suggested that the rapid increase in intracellular calcium was likely due to several mechanisms, including the formation of nanopores, intracellular calcium release from internal calcium stores such as endoplasmic or sarcoplasmic reticulum, and possible activation of voltage-gated or unspecific cation ion channels [18,19]. One possibility is that calcium enters the cell via mechanically activated channels or through the pore forming subunits of the piezo proteins found in cell membranes. Supporting this hypothesis, work done by Tolstykh et al. has conclusively shown that nsEP exposure activates the intracellular phosphoinositide signaling pathway [20–22], a well-known regulator of mechanically stimulated channel (MSC) activity and IP_3_ dependent intracellular calcium release [23–25]. The production of reactive oxygen species has also been observed to occur during nsEP exposures, although the connection to the other cellular effects of nsEPs is unknown at this time [26]. Nevertheless, based on these observations, it is clear that cells exposed to nsEP experience an intense stress that would lead to changes in gene expression.

To better characterize and understand this stress, and hopefully shed light on the biophysical mechanisms responsible for nanoporation, we performed a microarray analysis of both U937 and Jurkat cells exposed to 100 nsEPs at a duration of 10 ns and an electric field of 150kV/cm. Real-time quantitative PCR and luminex multiplexing assays were used to confirm the microarray data. The genomics and proteomic data presented in this paper provide the genetic evidence necessary to characterize the nature of the stress endured by both cells types when exposed to nsEP. This is the first time global genetic analysis has been applied to the cells exposed to nsEP.

## Materials and Methods

### Exposure System

**Fig 1:**
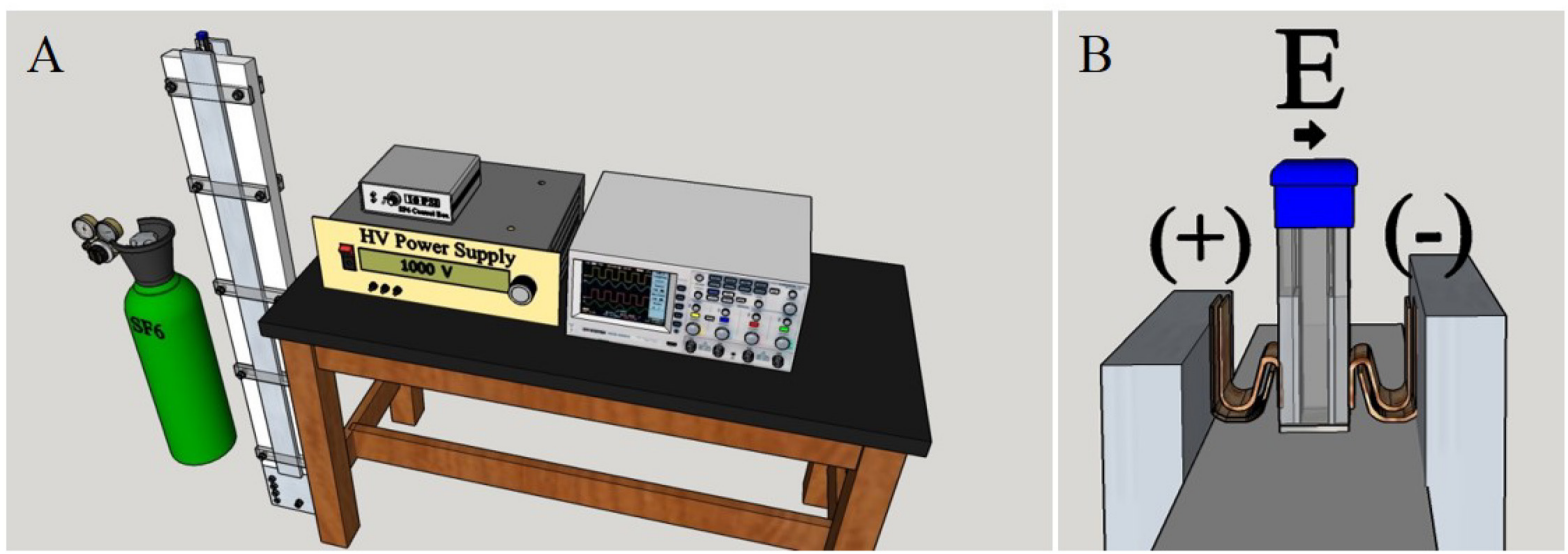
Blumlein line cuvette-based, 10 ns pulser apparatus. A) Drawing of the complete 10-ns set up, including the Tektronix ocilloscope, Glassman high voltage power supply and custom contol module for regulating the pressure of SF6 in the spark gap chamber. B) This is an enhanced view of the cuvette and its placement/orientation in regards to the pulser.

The 10-ns exposure system used in this study has been previously described in great detail [27,28]. In short, a custom pulser was constructed by investigators at Old Dominion University, consisting of a spark gap containing pressurized sulfur hexafluoride (SF_6_) (Fig 1). This chamber is charged using a high voltage power supply to breakdown the gas within the spark gap generating a pulse. At a constant pressure, increasing the voltage applied to the gap increases the repetition frequency of the pulses, while changing the gas pressure impacts the breakdown voltage resulting in a variable amplitude pulse. A custom controller box was designed that contains a computer-controlled pressure regulator to supply the pulser with SF_6_ at a controlled pressure. In addition, this box communicates with a power supply and a high speed oscilloscope to initiate pulse generation and count the pulses delivered to the exposure cuvette. This system is controlled using a LabVIEW (National Instruments, Austin, Texas) program that sets the exposure parameters and records the pulse amplitude.

### Cell Culture and Exposure

Both Jurkat (ATCC-TIB-152) and U937 (ATCC-CRL-1593.2) cells were acquired from ATCC (Manassas, VA) and sub-cultured according to supplier’s protocol. All cells were maintained at 37°C/5% CO_2_/95% humidity. Cells were grown and exposed in complete growth medium (ATCC, RPMI-1640 supplemented with 10% FBS, 1% Penicillin/Streptamycin). All cells were counted using the Countess^®^ Cell Counter from Life Technologies (Grand Island, NY) and the final concentration was adjusted to 1200 cells/μL. The cells were then aliquoted into electroporation cuvettes with a 1mm gap between plates (150 μL volume). The cuvettes were exposed to either 100, 10 ns electrical pulses at 150kV/cm or they were sham exposed. nsEP and sham exposures occurred in a random fashion. The sham control samples were treated identically to the nsEP exposed samples except, when they were placed on the pulser, zero power was applied. Following either sham or nsEP exposure, the cells were transferred into a well plate in triplicate and incubated in the appropriate cell culture conditions for the allotted time necessary for each assay. In order to induce heat shock stress, cells were placed in identical electroporation cuvettes and incubated in a circulating water bath at 44°C for 40 minutes [29].

### Viability Assay (MTT)

Cellular viability was evaluated 0.5, 4 and 24 h post-exposure using MTT (3-(4,5-Dimethylthiazol-2-yl)-2,5-diphenyltetrazolium bromide) assays, as per manufacturer’s instructions (ATCC). In brief, cells were exposed to nsEP and incubated at 37°C/5% CO_2_/95% humidity for a predetermined amount of time. 10 μL of MTT reagent was then added to each well and incubated for 2 h. After incubation, 100 μL of detergent was added to each well, the plate was covered in foil, placed on an orbital shaker at 100 rpm and incubated at room temperature overnight. The absorbance was measured at 570 nm with a Synergy HT Plate Reader (BioTek, Winooski, VT). A ladder of serial dilutions of cells (10^3^ to 10^6^ cells) in culture medium was prepared and absorbance measurements were conducted. The absorbance values were plotted versus the cell number and curves were generated and used to determine the number of viable cells in each well. A two-tailed unpaired t-test was performed using GraphPad Prism (GraphPad Software, Inc, La Jolla, CA).

### Flow Cytometry Analysis

Cells were prepared and exposed identically as in cell viability experiments (section 2.3). FITC-Annexin V and propidium iodide were added to each sample at 10μL/mL and 2μL/mL respectively. Both reagents were added to the cells within 5 minutes of nsEP exposure and then were allowed to incubate in the exposure media at room temperature (26°C) for 10 minutes to insure proper fluorescent staining. The effects of 10-ns exposures were analyzed using an Accuri Flow Cytometer from BD Biosciences (San Jose, California). A total volume of 75 μL of media was analyzed resulting in typical cellular counts of ~40,000 cells. Cellular expression was measured for each individual channel using sham exposure and digitonin (0.4%) exposures as positive and negative controls. A single threshold was determined and percent of cells expressing each dye was measured. A two-tailed unpaired t-test was performed using GraphPad Prism. Digitonin (Sigma-Aldrich, St. Louis, MO), a non-ionic detergent that is routinely used to permeablize cells, was used as a positive control in this assay.

### RNA Isolation

Total RNA was isolated from exposed cells and harvested 4 h after exposure. RNA was isolated with the Qiagen RNeasy Mini Kit and subjected to DNase digestion by the Qiagen RNase-free DNase Kit (QIAGEN Inc. Valencia, CA). RNA quantity was assessed by UV spectrometry at 260 nm / 280 nm absorbance on a NanoDrop Spectrophotometer (NanoDrop Technologies, Wilmington, DE). RNA quality was assessed on a Agilent Bioanalyzer using the Agilent RNA Nano Chips (Agilent Technologies, Waldbronn Germany).

### Microarray

Gene expression analysis was performed in triplicate (three sham, three nsEP exposed and three heat-shock treated) using the Affymetrix GeneChip^®^ Human Genome U133 (HG-U133) plus 2.0 Array that contains 54,675 probe sets. Briefly, two micrograms of RNA were used for preparation of biotin-labeled targets (cRNA) using MessageAmp^™^-based protocols (Ambion, Inc., Austin, TX). Labeled cRNA was fragmented (0.5 μg/μL per reaction) and used for array hybridization and washing. The cRNA was mixed with a hybridization cocktail, heated to 99°C for 5 min and then incubated at 45°C for 5 min. Hybridization arrays were conducted for 16 h in an Affymetrix Model 640 hybridization oven (45°C, and 60 rpm). Arrays were washed and stained on an FS450 Fluidics station and were scanned on a GeneChip^®^ Scanner 3000 7G. Image signal data, detection calls and annotations were generated for every gene using the Affymetrix Statistical Algorithm MAS 5.0 (GeneChip^®^ Operating Software v1.3). A log2 transformation was conducted and a Student’s t-test was performed for comparison of the nsEP exposed samples to the two control groups (sham and heat-shocked). We conducted multiple testing correction—Benjamini and Hochberg—to determine the false discovery rate, and statistically significant genes were identified using Bonferroni correction procedures.

### Microarray Data Analysis

For interpretation of the results, the Ingenuity Pathways Analysis tool (IPA version 8.7, Ingenuity^®^ Systems Inc., Redwood City, CA) was used. IPA is a web-based software application, which enables filtering and dataset comparisons, to identify biological mechanisms, pathways and functions most relevant to experimental datasets or differentially expressed genes. The cut-off criteria for our IPA analysis were: an absolute value of log ratio ≥2 or ≤-2 and a p-value ≤0.05. Other web-based resources, such as the GeneCards^®^ Human Gene Database, the HUGO Gene Nomenclature Committee (HGNC,) and the Gene Ontology Consortium were also used to further supplement the analysis.

### Quantitative Real Time Polymerase Chain Reaction

Each gene selected for validation was validated by quantitative real time polymerase chain reaction (qRT-PCR) using the Applied Biosystems StepOne^™^ Plus PCR system from ThermoFisher Scientific (Carlsbad, CA). Pre-made, validated TaqMan^®^ Gene Expression Assays were selected for each gene to be validated (ThermoFisher Scientific). Samples were run in triplicate with all reagents from ThermoFisher Scientific including the TaqMan^®^ One-Step RT-PCR Master Mix. Relative quantification (RQ) values were computed using the StepOne^™^ Plus software.

### Protein Isolation and Quantification

Cells were exposed to 10-ns pulse trains, then seeded into three different 12-well plates, and were allowed to incubate for up to twelve hours. One well plate of cells was processed at each time point: 4, 8 and 12 hours post-exposure. The harvested cells were aliquotted in triplicate into microcentrifuge tubes and placed on ice. The tubes were centrifuged at 1,400 rpm for five minutes to pellet the cells. The supernatant fluid was removed and this process was repeated one time using ice-cold PBS to ensure all foreign matter was removed before the cells were lysed by adding Complete Cell Extraction buffer from ThermoFisher Scientific (1% Triton X-100, 20 mM Tris-HCL, pH 7.4, 100 mM NaCl, 0.1 M EDTA, 0.2% SDS, 0.2 mM PMSF and 0.1 mM Leupepsin). The cell lysates were vortexed for two minutes. The lysate was clarified by centrifugation at 10,000 × g for 20 minutes at 4°C. The supernatant was collected and analyzed for protein content using a bicinchoninic acid (BCA) protein assay kit (Pierce^™^, Rockford, IL) and the analysis of the individual proteins was carried out via Luminex’s bead-based multiplexing immunoassay (EMD Millipore, Billerica, MA) following the manufacturer’s protocol. Briefly, the cell lysates were diluted 1:1 in assay buffer and 5 μg of total protein (25 μl/well) was loaded into the 96-well immunoassay plate along with lysate from positive controls (unstimulated HepG2 cells). Antibody-immobilized beads were added to each well and incubated for 2 h at room temperature (RT) followed by a 1 h incubation with detection antibodies. A streptavidin substrate was then added to each well and incubated for 30 minutes at RT after which the plate was run on a Luminex 200TM. The MILLIPLEX^®^ multiplex detection assay is a rapid alternative to Western blotting and immunoprecipitation. Assays such as this, have the capacity for multiple, conjugated beads to be added to each sample resulting in the ability to obtain multiplexed results from every sample. Two different MILLIPLEX^®^ multiplex detection assay were used, one testing proteins identified in the MAPK/SAPK pathway (MILLIPLEX MAP MAPK/SAPK Signaling 10-Plex Kit—Cell Signaling Multiplex Assay) and the other identifying proteins connected to the oxidative stress response pathway (MILLIPLEX MAP Human Oxidative Stress Magnetic Bead Panel—Cellular Metabolism Multiplex Assay).

## Results

### Viability

**Fig 2:**
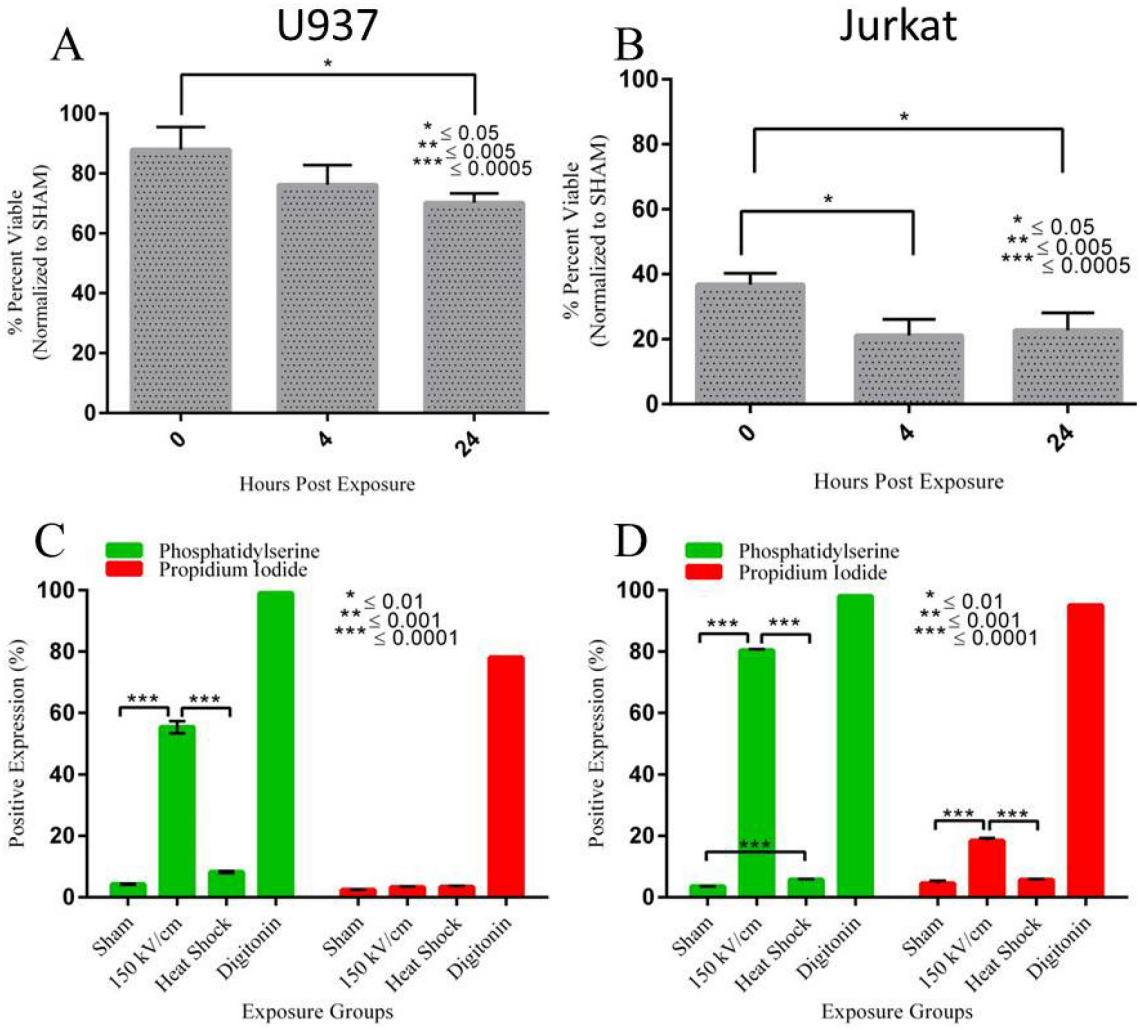
MTT and Flow data for U937 and Jurkat cells exposed to nsEP. A) Viability of U937 cells exposed to 100 x 10 ns pulses at 150 kV/cm. The lowest level of viability occurred 24 h post exposure. B) Viability of Jurkat cells exposed to 100 x 10 ns pulses at 150 kV/cm. The lowest level of viability occurred 4 h post exposure. C) Phosphatidylserine (PS) and propidium iodide (PI) expression in U937 cells exposed to 100 x 10 ns pulses at 150 kV/cm. D) Phosphatidylserine (PS) and propidium iodide (PI) expression in Jurkat cells exposed to 100 x 10 ns pulses at 150 kV/cm. Heat shock (exposure of cells to 44°C for 40 min) is a stress (apoptosis) control. Digitonin was the positive (necrotic) control.

We assessed the viability of both Jurkat and U937 cells at 0, 4 and 24 hours post nsEP exposure. Fig 2A and 2B show the resultant data for both U937 and Jurkat cells from the MTT assay. The values were normalized to the sham absorbance (100%) resulting in percentage of cell survival. U937 viability was only moderately affected by nsEP exposure at any of the time points. Immediately (within 15 minutes) after the nsEP exposure, approximately 88% of cells were viable. This number fell at both 4 and 24 h post exposure, with a viability of 76% and 70% respectively. Jurkat cells appear to have been more susceptible to the detrimental effects of nsEP stress, a finding that agrees well with previously published results [28,30]. Immediately after exposure, only 37% of Jurkat cells were viable. This number fell to 21% and 23% at 4 and 24 h post exposure respectively.

### Membrane Disruption and Nanoporation

FITC-Annexin V and propidium iodide (PI) dyes were used to measure the structure and permeability of the plasma membranes for each cell line exposed to nsEP. FITC-Annexin V dye is used to detect externalization of phosphatidylserine (PS), possibly indicating plasma membrane disruption. We used propidium iodide to detect the formation of nanopores. Fig 2C and 2D show PS/PI flow cytometry data for U937 and Jurkat cells exposed to nsEP. Gating thresholds were set on both fluorescent channels to determine whether a cell was positive or negative in expressing the dye of interest. sham exposure in both cell lines produced little to no expression in either channel, indicating no membrane disruption or nanoporation. Forty-one percent of exposed U937 cells had positive expression of FITC-Annexin V. However, only 5% of exposed U937 cells were positive for PI uptake indicating little to no nanoporation. The majority of exposed Jurkat cells, 79%, displayed positive FITC-Annexin V. Approximately 19% of exposed Jurkat cells were positive for PI expression. This data indicates that both cells experienced membrane disruption, with approximately twice as many Jurkat cells being affected.

### Microarray Analysis

**Fig 3:**
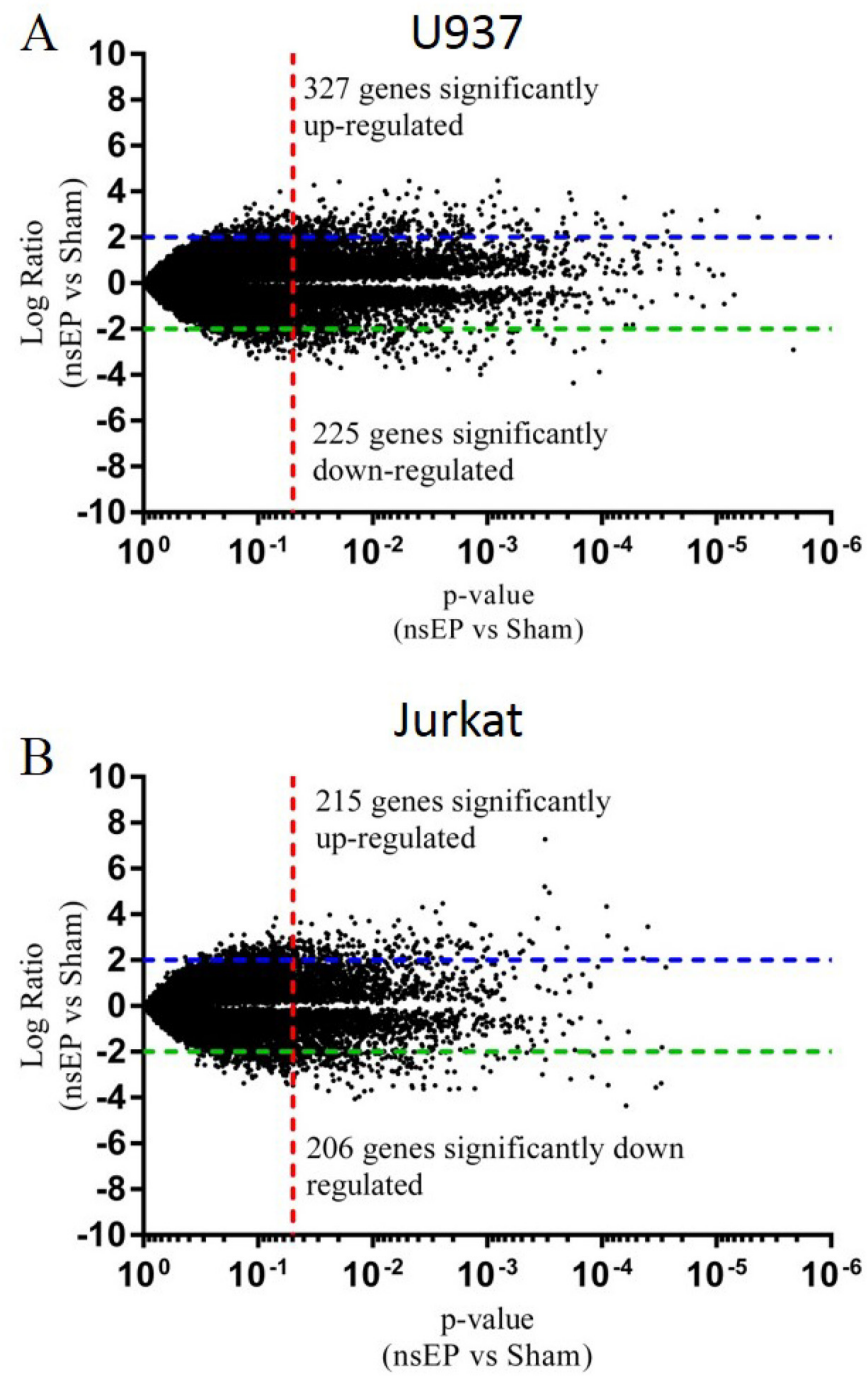
Volcano plots of significant gene expression. A) U937 cells exposed to nsEP had 327 genes significantly up-regulated as compared to sham (≥2 log ratio and p-value ≤ 0.05). 225 genes were significantly down regulated (≤-2 log ratio and p-value ≤ 0.05). B) Jurkat cells exposed to nsEP had 215 genes significantly up-regulated as compared to sham (≥2 log ratio and p-value ≤ 0.05). 206 genes were significantly down regulated (≤-2 log ratio and p-value ≤ 0.05).

The mRNA from Jurkat and U937 cells exposed to either thermal or nsEP stress was analyzed using standard microarray data analysis techniques. The nsEP exposed samples were compared to sham exposed samples and the log ratio for each gene was plotted with respect to their respective p-values. The resultant volcano plots are displayed in Fig 3A and 3B. The volcano plots for the heat-shocked vs sham-exposed cells can be found in the supplementary information (S1 and S2 Figs). Lines were inserted into the graph at a log ratio of +2 and -2. An additional line was inserted on the X-axis at a p-value of 0.05. Gene expression ratios above the +2 (or below the -2) line and to the right of the p-value line were considered to be significant. Thus, the significant genes were those that had a log ratio ≥2 or ≤-2 and a p-value ≤ 0.05. Of the genes analyzed, 327 were significantly up-regulated and 225 were significantly down-regulated in nsEP exposed U937 cells. Jurkat cells had 215 genes significantly up-regulated and 206 significantly down-regulated. The top 40 responding genes (20 genes with the highest log ratio and the 20 genes with lowest log ratio) for each cell line are listed in Tables 1 and 2. These tables are truncated. The complete tables (S1 and S2 Tables) for U937 and Jurkat cells exposed to nsEP can be found in the supplementary information. The complete tables of gene expression changes for the positive controls (heat shocked) for each cell line are also in the supplementary information (S3 and S4 Tables).

**Table 1:** Top 20 genes up- and down-regulated in U937 cells exposed to nsEP.

UniGene ID	Gene name	Symbol	Fold change 150kVnsEP vs. sham	p-Value 150kv nsEP vs. sham
Hs.446125	male germ cell-associated kinase	MAK	4.483	0.00081
Hs.155111	hepatitis A virus cellular receptor 2	HAVCR2	4.453	0.00479
Hs.351316	transmembrane 4 L six family member 1	TM4SF1	4.417	0.01954
Hs.154057	matrix metalloproteinase 19	MMP19	4.122	0.00389
Hs.370036	chemokine (C-C motif) receptor 7	CCR7	4.022	0.00779
Hs.551526	Brain-specific protein p25 alpha	TPPP	3.991	0.00274
Hs.267038	premature ovarian failure, 1B	POF1B	3.974	0.00078
Hs.369063	Zic family member 2	ZIC2	3.944	0.00019
Hs.351316	transmembrane 4 L six family member 1	TM4SF1	3.896	0.00504
Hs.315369	aquaporin 4	AQP4	3.886	0.00755
Hs.129794	spermatogenesis associated 12	SPATA12	3.745	0.00410
Hs.414795	serine (or cysteine) proteinase inhibitor, clade E	SERPINE1	3.692	0.00591
Hs.362807	interleukin 7 receptor /// interleukin 7 receptor	IL7R	3.652	0.02727
Hs.436298	epithelial membrane protein 1	EMP1	3.638	0.00018
Hs.436550	Na2+ channel, voltage gated, type VIII, alpha subunit	SCN8A	3.606	0.02903
Hs.504908	LIM domain only 3 (rhombotin-like 2)	LMO3	3.551	0.00110
Hs.91791	Transmembrane protein 16C	TMEM16C	3.468	0.00232
Hs.249718	eukaryotic translation initiation factor 4E	EIF4E	3.417	0.01264
Hs.514665	DLGAP1 antisense RNA 2	DLGAP1-AS2	3.404	0.00187
Hs.2258	matrix metallopeptidase 10 (stromelysin 2)	MMP10	3.331	0.02632
Hs.388715	small leucine-rich protein 1	SMLR1	-3.009	0.02216
Hs.433586	PPP5 tetratricopeptide repeat domain containing 1	PPP5D1	-3.032	0.00243
Hs.534859	Kazal-type serine peptidase inhibitor domain 1	KAZALD1	-3.066	0.00610
Hs.177193	synaptotagmin IX	SYT9	-3.082	0.00727
Hs.195298	sarcoglycan, zeta	SGCZ	-3.114	0.00341
Hs.549092	suppression of tumorigenicity 18, zinc finger	ST18	-3.199	0.00272
Hs.322444	RAMP2 antisense RNA 1	RAMP2-AS1	-3.224	0.00613
Hs.4290	RAB3C, member RAS oncogene family	RAB3C	-3.255	0.00912
Hs.208544	potassium channel, subfamily K, member 1	KCNK1	-3.273	0.00093
Hs.537383	olfactory receptor, family 5, subfamily H, member 1	OR5H1	-3.290	0.00431
Hs.549149	catenin (cadherin-associated protein), alpha 3	CTNNA3	-3.323	0.03010
Hs.131152	long intergenic non-protein coding RNA 643	LINC00643	-3.372	0.02482
Hs.408453	Wilms tumor 1	WT1	-3.440	0.00425
Hs.471162	Ras association (RalGDS/AF-6) and pleckstrin homology domains 1	RAPH1	-3.563	0.00105
Hs.274264	visual system homeobox 1	VSX1	-3.633	0.00772
Hs.27043	K+ voltage-gated channel, subfamily H member 5	KCNH5	-3.687	0.00549
Hs.387367	cytochrome P450, family 39, subfamily A, polypeptide 1	CYP39A1	-3.693	0.01892
Hs.147471	zinc finger protein 749	ZNF749	-3.713	0.00114
Hs.173536	protein kinase D3	PRKD3	-3.882	0.00010
Hs.440722	zinc finger protein 587	ZNF417	-3.996	0.00115
Hs.146040	chromosome 14 open reading frame 105	C14orf105	-4.364	0.00018

**Table 2:** Top 20 genes up- and down-regulated in Jurkat cells exposed to nsEP.

UniGene ID	Gene name	Symbol	Fold change 150kVnsEP vs. sham	p-Value 150kv nsEP vs. sham
Hs.25647	v-fos FBJ murine osteosarcoma viral oncogene homolog	FOS	7.269	0.00031
Hs.326035	Early growth response 1	EGR1	5.213	0.00031
Hs.326035	early growth response 1	EGR1	4.941	0.00029
Hs.549031	early growth response 4	EGR4	4.472	0.00244
Hs.326035	early growth response 1	EGR1	4.349	0.00009
Hs.494326	basic leucine zipper nuclear factor 1 (JEM-1)	BLZF1	4.106	0.00281
Hs.1395	early growth response 2	EGR2	3.981	0.02375
Hs.536535	dual specificity phosphatase 16	DUSP16	3.832	0.00036
Hs.529512	zinc finger protein 167	ZNF167	3.700	0.01073
Hs.525704	v-jun sarcoma virus 17 oncogene homolog	JUN	3.606	0.00257
Hs.195398	oligodendrocyte transcription factor 3	OLIG3	3.604	0.01432
Hs.413099	glycine receptor, alpha 3	GLRA3	3.579	0.03397
Hs.75678	FBJ murine osteosarcoma viral oncogene homolog B	FOSB	3.577	0.00137
Hs.549086	discs, large (Drosophila) homolog-associated protein 1	DLGAP1	3.523	0.00130
Hs.532933	purinergic receptor P2Y, G-protein coupled, 12	P2RY12	3.499	0.00649
Hs.525704	v-jun sarcoma virus 17 oncogene homolog	JUN	3.466	0.00004
Hs.519601	Inhibitor of DNA binding 4, dominant negative helix-loop-helix	ID4	3.400	0.00024
Hs.56247	inducible T-cell co-stimulator	ICOS	3.390	0.00468
Hs.162246	transmembrane protein 171	TMEM171	3.265	0.01988
Hs.498513	aldo-keto reductase family 1, member C2	AKR1C1/AKR1C2	3.232	0.00894
Hs.369263	PDS5, regulator of cohesion maintenance, homolog B (S. cerevisiae)	PDS5B	-3.059	0.03108
Hs.24115	miR-17-92 cluster host gene (non-protein coding)	MIR17HG	-3.098	0.04827
Hs.510093	Abelson helper integration site 1	AHI1	-3.113	0.00354
Hs.551839	uncharacterized LOC284600	LOC284600	-3.155	0.04887
Hs.159234	forkhead box E1 (thyroid transcription factor 2)	FOXE1	-3.184	0.01954
Hs.271791	ATR serine/threonine kinase	ATR	-3.186	0.00019
Hs.525700	small nuclear ribonucleoprotein polypeptide N	SNRPN	-3.377	0.00003
Hs.371903	glycophorin E (MNS blood group)	GYPE	-3.398	0.01643
Hs.72901	cyclin-dependent kinase inhibitor 2B (p15, inhibits CDK4)	CDKN2B	-3.451	0.00363
Hs.2799	hyaluronan and proteoglycan link protein 1	HAPLN1	-3.471	0.00454
Hs.44685	ring finger protein 141	RNF141	-3.474	0.04947
Hs.549172	calmodulin-like 4	CALML4	-3.495	0.00665
Hs.54973	cadherin 26	CDH26	-3.551	0.03073
Hs.519523	serpin peptidase inhibitor, clade B (ovalbumin), member 6	SERPINB6	-3.560	0.00003
---	long intergenic non-protein coding RNA 644	LINC00644	-3.568	0.00450
Hs.76561	zinc finger protein 404	ZNF404	-3.603	0.00177
Hs.170849	coiled-coil domain containing 122	CCDC122	-3.614	0.00129
Hs.389945	WD repeat domain 60	WDR60	-3.641	0.00213
Hs.242520	uromodulin-like 1	UMODL1	-3.680	0.01025
Hs.436380	MAM domain containing glycosylphosphatidylinositol anchor 2	MDGA2	-3.703	0.02430
Hs.72307	G protein-coupled receptor 110	GPR110	-3.720	0.01181

### Ingenuity Systems IPA Pathway Analysis

The microarray data for each cell line was loaded into Ingenuity Systems IPA pathway analysis software and a core analysis was performed for the top 5000 genes from each set of microarray data. A summary table for each cell line can be found in Tables 3 and 4 respectively. Table 3 contains the top molecular and cellular functions impacted by the gene expression pattern created by nsEP exposure. The functions of cellular movement, cell-to-cell signaling/interaction and cellular development were among the top functions in both cell lines. In the U937 cells exposed to nsEP, 751 molecules associated with cell growth represented the largest number of molecules changing that were associated with a specific cellular function. In the Jurkat cell line, the category of molecular function of cellular development had the largest number of molecules, 575 in all, affected by the nsEP pulse.

**Table 3:** Pathway Analysis: Top Molecular Functions.

Molecular and Cellular Functions		
Function	p-values	# of Molecules
*U937*		
Cellular Movement	5.48E^-13^–1.32E^-03^	455
Cell-To-Cell Signaling and Interaction	1.95E^-11^–1.28E^-03^	533
Cellular Development	4.35E^-08^–1.28E^-03^	593
Cellular Growth and Proliferation	9.37E^-08^–1.28E^-03^	751
Cell Death and Survival	2.65E^-07^–1.36E^-03^	734
*Jurkat*		
Cellular Movement	1.52E^-14^–1.10E^-03^	429
Cell-To-Cell Signaling and Interaction	8.03E^-08^–1.17E^-03^	508
Nucleic Acid Metabolism	1.23E^-07^–5.33E^-05^	70
Small Molecule Biochemistry	1.23E^-07^–1.10E^-03^	283
Cellular Development	1.50E^-07^–1.11E^-03^	575

**Table 4:** Top Canonical Pathways.

Canonical Pathway		
Function	p-values	# of Molecules
*U937*		
VDR/RXR Activation	4.86E^-06^	33/77 (0.429)
B Cell Development	9.09E^-05^	15/28 (0.536)
ILK Signaling	1.03E^-04^	58/181 (0.32)
tRNA Splicing	1.54E^-04^	17/35 (0.486)
Hepatic Fibrosis / Hepatic Stellate Cell Activation	1.74E^-04^	61/196 (0.311)
*Jurkat*		
Hepatic Fibrosis / Hepatic Stellate Cell Activation	2.9E^-06^	63/196 (0.321)
G-Protein Coupled Receptor Signaling	1.17E^-05^	75/254 (0.295)
LPS/IL-1 Mediated Inhibition of RXR Function	1.2E^-05^	64/208 (0.308)
cAMP-mediated signaling	8.37E^-05^	63/216 (0.292)
Atherosclerosis Signaling	1.62E^-04^	39/120 (0.325)

Canonical pathways are “idealized or generalized pathways”; they are considered to be pathways that have previously been well established and classically characterized. The core analysis function in the IPA software identified the top 5 canonical pathways affected by nsEP exposure (Table 4). For the U937 cells, the top pathway affected (based on lowest p-value) was the VDR/RXR Activation pathway with 33 of the 77 (43%) genes changing due to nsEP exposure. For the Jurkat cells, the top canonical pathway was the “Hepatic Fibrosis / Hepatic Stellate Cell Activation”, with a p-value of 2.9E^-06^ and 63 of 196 molecules (32%) effected. The IPA software core analysis does not suggest which, if any, non-canonical pathways may be effected.

Comparative analysis of the top 5000 genes changing within in each cell line due to nsEP exposure was conducted. Both cell lines shared 1624 common genes changing due to nsEP exposure. However, only 890 of the shared genes had changed in the same manner, i.e. both up-or both down-regulated. Of these 890 genes, only 59 genes had a log ratio ≥1.0 (fold change = 2) and a p-value of ≤0.05 (Table 5).

**Table 5:** Genes up- or down-regulated in common by nsEP exposure in Jurkat and U937 cells.

Gene Symbol	Gene name	Jurkat Fold Change	p-Value	U937 Fold Change	p-Value
JUN	jun proto-oncogene	3.606	0.0026	2.444	0.0001
VEGFA	vascular endothelial growth factor A	2.594	0.0396	1.32	0.0146
RORA	RAR-related orphan receptor A	2.569	0.0471	1.949	0.0072
MXD1	MAX dimerization protein 1	2.354	0.0095	1.635	0.0072
ATF3	activating transcription factor 3	2.301	0.0005	1.216	0.0014
PPP1R15A	protein phosphatase 1, regulatory subunit 15A	2.298	0.0032	2.21	0.0002
NDUFA10	NADH dehydrogenase (ubiquinone) 1 alpha, 10, 42kDa	2.286	0.0018	1.83	0.0424
BHLHE40	basic helix-loop-helix family, member e40	2.235	0.0338	1.253	0.0013
LDLR	low density lipoprotein receptor	2.218	0.0096	2.463	0.0386
NRP2	neuropilin 2	2.082	0.0295	3.022	0.0031
SASH1	SAM and SH3 domain containing 1	2.081	0.0000	1.768	0.0204
TBX3	T-box 3	2.024	0.0420	2.052	0.0060
DOCK4	dedicator of cytokinesis 4	1.942	0.0134	1.307	0.0303
SLC7A11	solute carrier family 7 member 11	1.875	0.0041	2.309	0.0040
ULK2	unc-51 like autophagy activating kinase 2	1.848	0.0293	1.38	0.0177
S1PR3	sphingosine-1-phosphate receptor 3	1.824	0.0463	1.18	0.0047
DUSP10	dual specificity phosphatase 10	1.821	0.0046	1.571	0.0108
CHD2	chromodomain helicase DNA binding protein 2	1.75	0.0034	1.511	0.0231
TSC22D3	TSC22 domain family, member 3	1.729	0.0370	1.807	0.0408
CACNB2	calcium channel, voltage-dependent, beta 2 subunit	1.718	0.0290	2.412	0.0409
EYA3	EYA transcriptional coactivator and phosphatase 3	1.613	0.0322	2.8	0.0480
RNA45S5	RNA, 45S pre-ribosomal 5	1.599	0.0003	2.887	0.0009
CREBRF	CREB3 regulatory factor	1.578	0.0267	1.092	0.0232
RNF31	ring finger protein 31	1.545	0.0488	1.72	0.0123
CAMK2B	calcium/calmodulin-dependent protein kinase II beta	1.529	0.0252	1.706	0.0387
KLF6	Kruppel-like factor 6	1.473	0.0016	1.939	0.0029
SESN2	sestrin 2	1.463	0.0123	1.606	0.0000
MASP2	mannan-binding lectin serine peptidase 2	1.448	0.0179	2.265	0.0283
ARG1	arginase 1	1.382	0.0441	1.386	0.0430
NPR1	natriuretic peptide receptor 1	1.362	0.0080	2.291	0.0118
TM4SF1	transmembrane 4 L six family member 1	1.349	0.0249	4.417	0.0195
FAM122C	family with sequence similarity 122C	1.31	0.0341	1.058	0.0162
JUND	jun D proto-oncogene	1.308	0.0042	1.733	0.0057
SPATA6	spermatogenesis associated 6	1.27	0.0237	1.924	0.0105
PHLDA1	pleckstrin homology-like domain, family A, member 1	1.142	0.0074	2.861	0.0002
NEU1	sialidase 1 (lysosomal sialidase)	1.134	0.0296	1.201	0.0014
CD55	CD55 molecule, decay accelerating factor for complement	1.11	0.0324	1.321	0.0062
ABL2	ABL proto-oncogene 2, non-receptor tyrosine kinase	1.079	0.0455	1.267	0.0052
SKIL	SKI-like proto-oncogene	1.073	0.0026	1.554	0.0302
HAO2	hydroxyacid oxidase 2 (long chain)	1.042	0.0494	1.618	0.0393
MAFF	v-maf avian musculoaponeurotic fibrosarcoma oncogene F	1.007	0.0170	1.653	0.0022
MEX3D	mex-3 RNA binding family member D	-1.045	0.0318	-2.098	0.0012
EBF3	early B-cell factor 3	-1.082	0.0122	-1.047	0.0403
ZNF205	zinc finger protein 205	-1.083	0.0039	-2.33	0.0060
PPIL2	peptidylprolyl isomerase (cyclophilin)-like 2	-1.123	0.0417	-2.111	0.0041
XIRP2	xin actin-binding repeat containing 2	-1.19	0.0204	-2.27	0.0255
TEX14	testis expressed 14	-1.22	0.0002	-1.113	0.0242
CLECL1	C-type lectin-like 1	-1.23	0.0407	-2.337	0.0097
HNRNPD	heterogeneous nuclear ribonucleoprotein D	-1.307	0.0083	-1.545	0.0113
GABBR2	gamma-aminobutyric acid (GABA) B receptor, 2	-1.32	0.0411	-1.784	0.0319
PPP2R5A	protein phosphatase 2, regulatory subunit B', alpha	-1.38	0.0121	-1.722	0.0199
GPR98	G protein-coupled receptor 98	-1.515	0.0408	-1.46	0.0232
SATB1	SATB homeobox 1	-1.97	0.0350	-1.548	0.0003
RERE	arginine-glutamic acid dipeptide (RE) repeats	-2.105	0.0373	-2.224	0.0488
FERMT1	fermitin family member 1	-2.548	0.0123	-1.4	0.0182
TSEN54	TSEN54 tRNA splicing endonuclease subunit	-2.707	0.0058	-1.638	0.0389
PHLPP1	PH domain and leucine rich repeat protein phosphatase 1	-2.862	0.0066	-1.037	0.0132
HAPLN1	hyaluronan and proteoglycan link protein 1	-3.471	0.0045	-2.922	0.0221

### qRT-PCR

**Fig 4:**
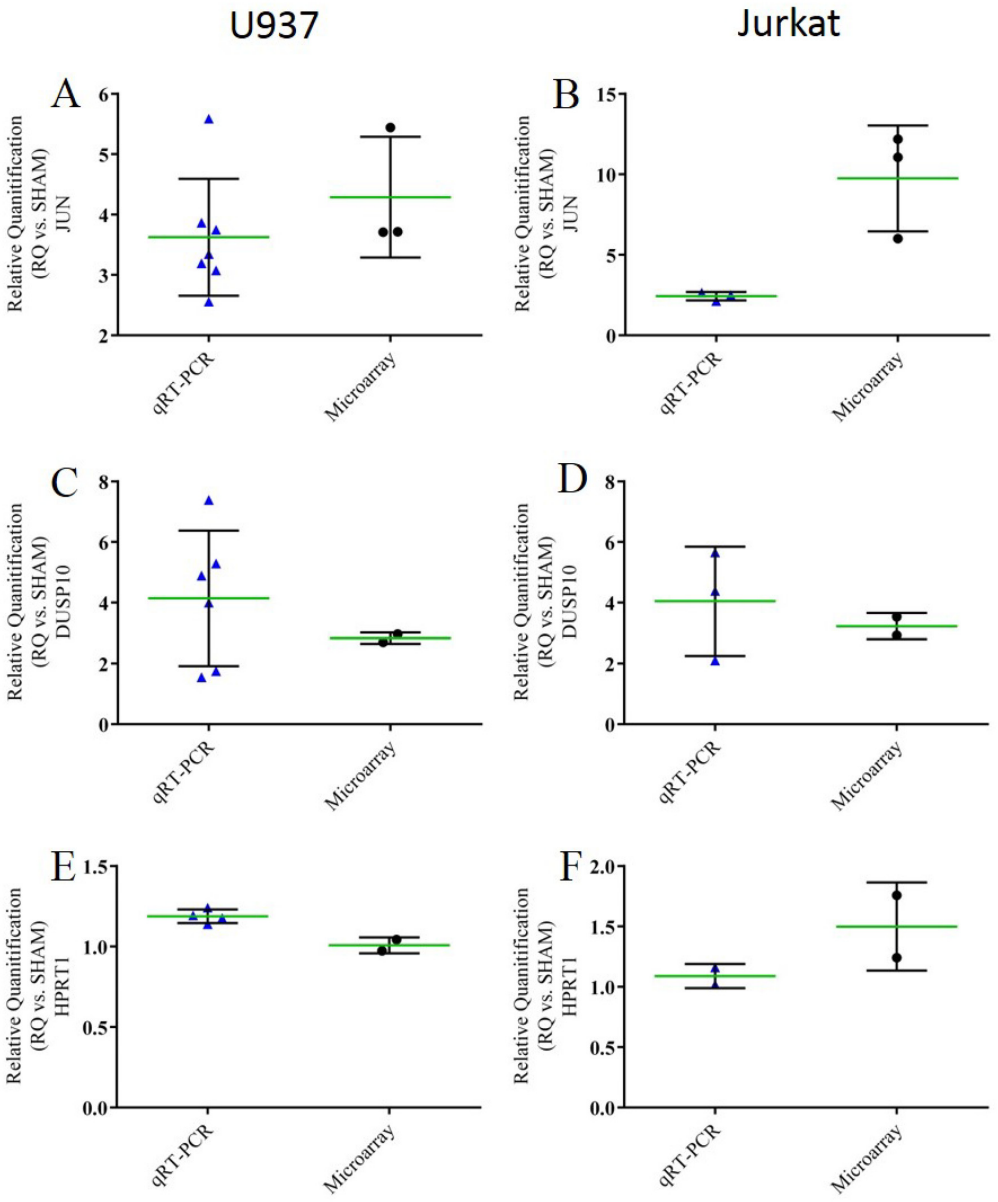
Scatter dot plot for each qRT-PCR validation sample. A) Comparison of the expression levels of JUN for U937 cells exposed to nsEP. B) Comparison of the expression levels of JUN for Jurkat cells exposed to nsEP. C) Comparison of the expression levels of DUSP10 for U937 cells exposed to nsEP. D) Comparison of the expression levels of DUSP10 for Jurkat cells exposed to nsEP. E) Comparison of the expression levels of HPRT1 for U937 cells exposed to nsEP. F) Comparison of the expression levels of HPRT1 for Jurkat cells exposed to nsEP. Mean and standard deviation are plotted as the green and black lines respectively.

We validated microarray expression for 3 common genes in both U937 and Jurkat cells. qRT-PCR was performed for the putative transforming gene of avian sarcoma virus 17, commonly known as *jun proto-oncogene* (JUN) in humans. Microarray data indicated JUN expression was increased approximately 4 fold (±1). We confirmed that JUN expression was up-regulated in U937 cells exposed to nsEP; qRT-PCR indicated JUN expression was increased 4 fold (±1) (Fig 4A). The same result was determined with the Jurkat cells, however, the level of JUN expression as determined by microarray was 3x greater than what was determined by qRT-PCR (9-fold change in microarray as compared to 3-fold change in qRT-PCR) (Fig 4B). We validated the microarray expression level for the gene that codes for the *dual specificity protein phosphatase*, DUSP10. In U937 cells, DUSP10 was significantly up-regulated 5-fold as determined by qRT-PCR and 3-fold as determined by microarray analysis (Fig 4C). In Jurkat cells, DUSP10 was also determined to be significantly up-regulated 5-fold as determined by qRT-PCR and 2.5-fold as determined by microarray analysis (Fig 4D) For the housekeeping gene HPRT1, both qRT-PCR and microarray data agreed in both cell lines, suggesting little to no effect due to nsEP exposure (Fig 4E and 4F). Single reaction qRT-PCR was performed on many other genes of interest for U937 cells. These data can be found in the supplementary information (S3, S4, S5 and S6 Figs).

### Luminex Assays

**Fig 5:**
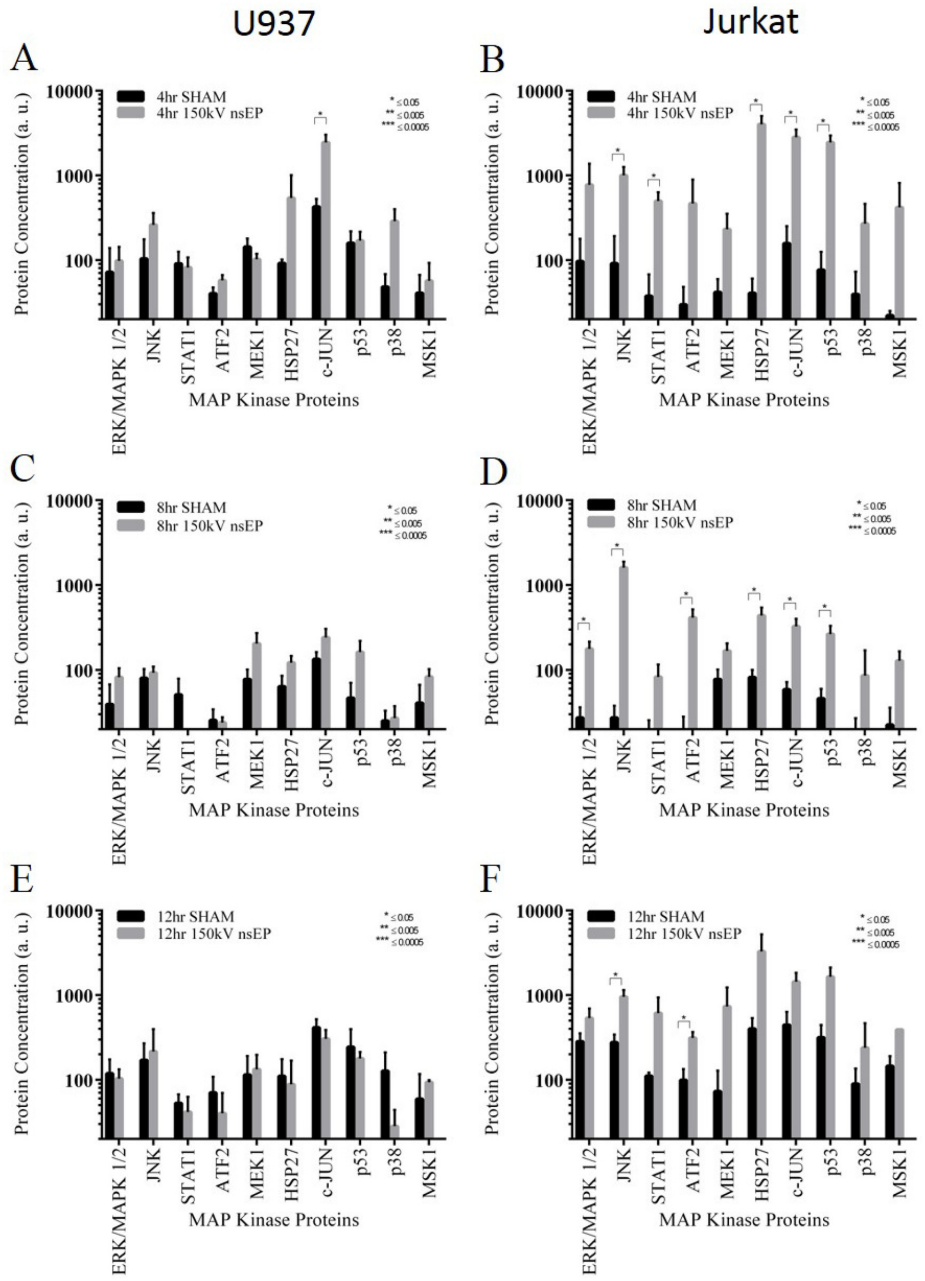
Levels of MAP Kinase associated proteins post nsEP exposure for U937 and Jurkat Cells. A) Levels of MAPK proteins for U937 at 4 h post exposure. B) Levels of MAPK proteins for Jurkat at 4 h post exposure. C) Levels of MAPK proteins for U937 at 8 h post exposure. D) Levels of MAPK proteins for Jurkat at 8 h post exposure. E) Levels of MAPK proteins for U937 at 12 h post exposure. F) Levels of MAPK proteins for Jurkat at 12 h post exposure. Error bars represent standard deviation (SD).

In an effort to link genetic and proteomic data, we performed a bead based multiplexing assay for the MAPK pathway. The bead based kit was used to look for changes in *signal transducer and activator of transcription 1* (STAT1), *activating transcription factor 2* (ATF2), *extracellular signal-regulated kinase* (Erk), *heat shock 27kDa protein 1* (HSP27), *c-Jun N-terminal kinase* (JNK), *jun proto-oncogene* (c-Jun), *dual specificity mitogen-activated protein kinase kinase 1* (Map2K1 or MEK1), *mitogen- and stress-activated protein kinase-1* (MSK1), *p38 mitogen-activated protein kinase* (p38) and *tumor protein 53* (p53). At the 4 h time point, only the protein c-Jun was significantly increased by nsEP exposure in the U937 cell line (Fig 5A). In stark contrast, the Jurkat cells had 5 of the MAPK pathway proteins significantly increased due to nsEP exposure (Fig 5B). At 8 h post exposure, none of the MAPK pathway proteins were increased due to nsEP exposure in the U937 cells (Fig 5C); however, in the Jurkat cells exposed to nsEP, 6 of the MAPK pathway-associated proteins were significantly increased (Fig 5D). These included ERK1, JNK, ATF2, HSP27, c-JUN, and p53. At 12 h post exposure, none of the MAPK proteins were changed in the U937 cells (Fig 5E), suggesting they had reached a steady state. The Jurkat cells, at 12 h post exposure, still had significantly more JNK and ATF2 proteins compared to the sham samples (Fig 5F).

**Fig 6:**
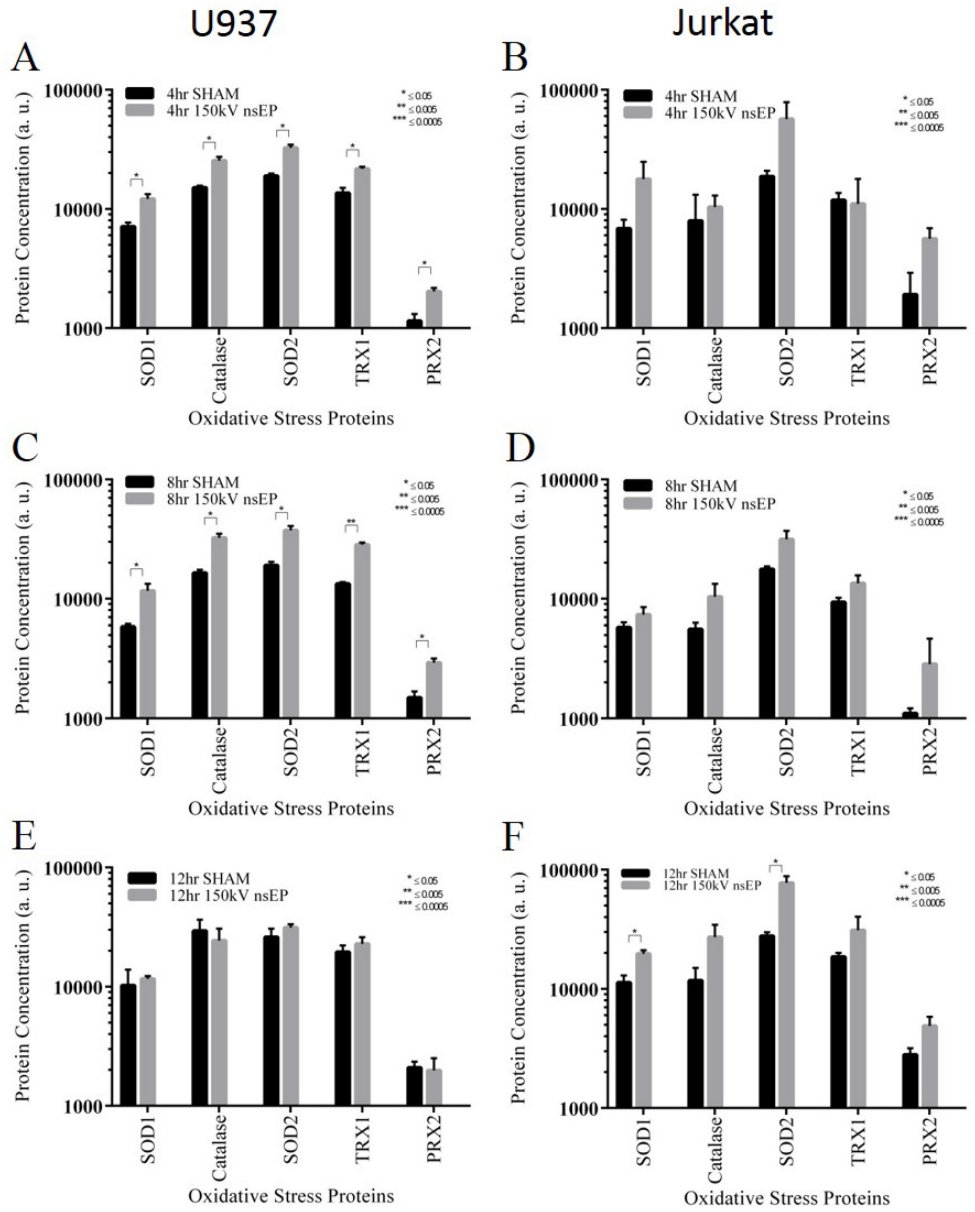
Oxidative stress related protein levels in U937 and Jurkat cells exposed to nsEP. A) Levels of oxidative stress proteins for U937 at 4 h post exposure. B) Levels of oxidative stress proteins for Jurkat at 4 h post exposure. C) Levels of oxidative stress proteins for U937 at 8 h post exposure. D) Levels of oxidative stress proteins for Jurkat at 8 h post exposure. E) Levels of oxidative stress proteins for U937 at 12 h post exposure. F) Levels of oxidative stress proteins for Jurkat at 12 h post exposure. Error bars represent standard deviation (SD).

Proteins associated with oxidative stress were also surveyed via Luminex multiplexing bead assay. The human oxidative stress magnetic bead panel was used to measure the amount of *Catalase*, *Peroxiredoxin 2* (PRX2), *Superoxide dismutase-1* [Cu-Zn] (SOD1), *Superoxide dismutase-2* [Mn] (SOD2), and *Thioredoxin* (TRX1). At 4 h post nsEP exposure, U937 cells had significant increases in all of the assayed proteins, SOD1, Catalase, SOD2, TRX1 and PRX2 (Fig 6A). SOD1, SOD2 and PRX2, in the Jurkat cells at 4 h post exposure, appear to change in response to nsEP exposure, although not to the point of statistical significance (Fig 6B). At 8 h, all of the proteins increased in the U937 cells remained significantly up-regulated (Fig 6C). At 8 h post, as at 4 h post, changes in the levels of the oxidative stress proteins were not statistically significant; however all of the assayed proteins appear to be increasing in the Jurkat cells (Fig 6D). At 12 h post, no proteins appear to be increased in U937 cells, apparently having returned to a steady state level (Fig 6E). In the Jurkat cells, at 12 h post, the only proteins significantly increased were SOD1 and SOD2 (Fig 6F).

## Discussion

The viability (MTT) and cell flow data clearly show that both cells lines are affected by the nsEP exposure parameters used in this paper. Jurkat cells have been used extensively in nsEP research [31–34] and have been shown to be sensitive to these types of ultrashort pulses [28]. Our group speculated that Jurkat sensitivity to nsEP could be based on “inherent susceptibilities.” Thus, we believed the most efficient method for identifying these “inherent susceptibilities” was to perform a microarray analysis thus leading to this current body of work. Given the viability data, we feel confident that the exposure parameters were sufficient to elicit a survival/stress response requiring the up/down regulation of specific genes. Further evidence that the exposure parameters used were sufficient to cause a known effect associated with nsEP can be seen with the flow cytometry data. Vernier was among the first researchers to identify PS rearrangement as a direct effect associated with nsEP exposure [9,34–36]. Our data suggests that the nsEP parameters we used were not only effective at causing a biochemical response (loss of cell viability) but also a physical response (rearrangement of phosphatidylserine residuals in the plasma membrane).

The microarray data presented here is a mere snapshot of the molecular changes that occurred within these cells 4 h post exposure. Both the Jurkat and U937 cells have approximately the same number of genes changing (400–500 genes each) despite their different responses to nsEP exposure (viability and PS expression). This number of genes is low in comparison to a heat shock stress which causes approximately 1200 genes to have a significant response (S1 and S2 Figs). With the filter criteria relaxed to a log ratio of ≥1 or ≤-1 and a p-value of ≤0.05, only 59 genes are equally up- or down-regulated in both cell lines due to nsEP exposure. With the small number of genes being shared by both cell lines, it is difficult to pinpoint the activation of a specific, dominant pathway; however, IPA core analysis suggests many common stress pathways are shared, suggesting a common, generalized physiological response, although the genetic response appears to be tailored to each cell line.

The most striking finding of this entire study is the indication by the IPA software (based on the gene profiles) that the major cellular/molecular function affected by nsEP exposure is cellular growth/development/movement. This is in stark contrast to the MTT data. The viability data suggests the Jurkat cells are quite susceptible to the effects of nsEP with only 23% of the cells surviving at 4 h post exposure. Based on the loss of viability, we expected the genetic profile to indicate possibly necrotic or apoptotic gene pathway up-regulation, but instead, cellular growth development/movement functions are indicated as the top functions up-regulated in each cell line following nsEP exposure.

Despite these findings, identification of a dominant pathway was not possible. The increase in cellular growth development/movement functions is not the result of an individual pathway, but rather is the result of an intricate network of many pathways. Therefore, given this level of complexity, we analyzed individual genes, their response to stress and their associated pathways. Analysis of the top 20 genes changing in both cell lines indicated many of these genes play important roles in the cellular response to mechanical stress (Table 6). Of the top 20 genes from both cell lines, 25% have specific functions associated with either MAPK signaling or directly in cellular growth. The majority of these genes are found in the Jurkat genetic profile. Another 25% of the top 20 genes up-regulated in these cell lines by nsEP are associated with the disruption of the plasma membrane, with the majority of these genes being present in the U937 cells. This finding directly correlates to the level of PS detected in flow cytometry, directly tying an observed bioeffect associated with nsEP to a specific genetic response.

**Table 6:** Mechanical stress-associated genes Up-Regulated by nsEP.

Gene Name	Symbol	Function (from NCBI Resources: Gene)	Cell
***Mechanical*: *Increases in MAP Kinase Signaling Pathway//increases in cell growth***			
male germ cell-associated kinase	MAK	serine/threonine protein kinase related to kinases involved in cell cycle regulation	U937
v-jun sarcoma virus 17 oncogene homolog	JUN	interacts directly with specific target DNA sequences to regulate gene expression, part of the JNK pathway	U937/ Jurkat
FBJ murine osteosarcoma viral oncogene homolog	FOS	regulator of cell proliferation, differentiation, and transformation.	Jurkat
Early Growth Response 1	EGR1	induces the expression of growth factors, growth factor receptors, extracellular matrix proteins, proteins involved in the regulation of cell growth or differentiation, and proteins involved in apoptosis, growth arrest, and stress responses	Jurkat
Early Growth Response 4	EGR4	cell proliferation by increased expression of potassium chloride cotransporter 2b (KCC2b)	Jurkat
basic leucine zipper nuclear factor 1	BLZF1	regulation of cell growth	Jurkat
Early Growth Response 2	EGR2	mediates NFκB and MAPK signaling	Jurkat
dual specificity phosphatase 10	DUSP10	regulates the c-Jun amino-terminal kinase (JNK) and extracellular signal-regulated kinase (ERK) pathways	U937 Jurkat
dual specificity phosphatase 16	DUSP16	regulates the c-Jun amino-terminal kinase (JNK) and extracellular signal-regulated kinase (ERK) pathways	Jurkat
***Mechanical*: *Calcium release from the endoplasmic reticulum***			
flavin containing monooxygenase 3	FMO3	transmembrane protein localizes to the endoplasmic reticulum	U937
***Mechanical*: *Disruption of the plasma membrane*, *extracellular matrix and cytoskeleton***			
transmembrane 4 L six family member 1	TM4SF1	molecular organizer that interacts with membrane and cytoskeleton-associated proteins	U937
matrix metalloproteinase 19	MMP19	breakdown of extracellular matrix	U937
premature ovarian failure, 1B	POF1B	binds non-muscle actin filaments	U937
Brain-specific protein p25 alpha	TPPP	binds to tubulin and microtubules and induces aberrant microtubule assemblies	U937
aquaporin 4	AQP4	function as water-selective channels in the plasma membranes	U937
Anoctamins	TMEM16C	phospholipid scrambling	U937
matrix metalloproteinase 10 (stromelysin 2)	MMP10	breakdown of extracellular matrix	U937
Annexin A1	ANXA1	membrane-localized protein that binds phospholipids	U937
glycine receptor, alpha 3	GLRA3	encodes a member of the ligand-gated ion channel protein family	Jurkat
***Mechanical*: *Increased IP3 production***			
chemokine (C-C motif) receptor 7	CCR7	member of the G protein-coupled receptor family	U937
***Mechanical*: *Increases in GPCR***			
regulator of G-protein signaling 1	RGS1	attenuates the signaling activity of G-proteins by binding to activated, GTP-bound G alpha subunit	U937
polycystic kidney disease 1 like 1	PKD1L1	novel G-protein-binding protein. Ca2+-permeable pore-forming subunits and receptor-like integral membrane proteins	U937
purinergic receptor P2Y, G-protein coupled, 12	P2RY12	belongs to the family of G-protein coupled receptor	Jurkat

Given the level of gene expression associated with cellular growth and the apparent activation of the MAPK pathway, we correlated gene expression with protein levels using a quantitative Luminex assay. The MAPK pathway can be activated by mitogens or through certain stress pathways. Activation of the MAPK pathway in response to nsEP has been shown before, however, it was not linked to a specific genetic response [37,38]. The Luminex data suggests that the MAPK pathway is activated within the Jurkat cells for several hours, eventually returning to a quasi-normal state at 12 h post exposure. These data are reflected by the associated gene data shown in Table 6. The U937 cells do not appear to have MAPK proteins significantly expressed in response to nsEP at the time points examined. Nevertheless, genetic evidence suggests that the MAPK pathway may be activated in the U937 cells, as shown by the increase in the dual specificity protein phosphatase (DUSP) genes. The DUSP genes essentially “turn off” the MAPK pathway, and several of these genes are up-regulated in the U937 cells following nsEP.

Of the top 10 significantly changing genes in response to nsEP, EGR1 appears 3 times, with EGR4 and EGR2 appearing once each. ERG genes have been associated with MAPK pathway activation, but also, they have also been found to be up-regulated as part of the antioxidative response [39]. It is unclear if the ERG genes are up-regulated in response to the MAPK pathway or another pathway, however the production of reactive oxygen species (ROS) have been observed with nsEP exposures [40]. The source of the ROS is not clear, however it is possible that both electrochemistry and mitochondria stimulation contribute to the increased levels. Beebe has published a great deal concerning the effect of nsEP on mitochondria and the subsequent flooding of calcium into cells post exposure [15,19,32,41–43]. The mitochondria appear to be both directly (increased membrane permeability) and indirectly (storage of free calcium) affected by nsEP. Given that mitochondria appear to be a target of nsEP and the possible role of EGRs as initiators of an antioxidant response, we looked at oxidative stress proteins for both Jurkat and U937 cells exposed to nsEP. Proteins associated with oxidative stress were observed as not being statistically significant, but appear to be slightly increased in the Jurkat cells. This was somewhat expected, given the increased levels of EGR gene expression in Jurkat cells. Increased expression of EGR genes give cells an ability to initiate an antioxidant response. On the contrary, the U937 cells, which had no significant genetic increases in EGR genes, had many proteins associated with oxidative stress significantly up-regulated.

Based on the genetic response of both cell lines, it appears that these cells respond to nsEP as a mechanical stress. It has been reported in other studies that osteoblasts undergoing mechanical stretch, preferentially up-regulated FOS, JUN, and EGR1, 2, and 3 genes [44]. The authors of the osteoblast study go on to suggest that EGR2 is actually a “mechanically sensitive gene” [44]. It is important to remember that Jurkat cells are not osteoblasts and any mechanical stress associated with nsEP is most likely due to acute and not tension like stress. The MAPK pathway has also been shown to be specifically up-regulated by mechanical stress [45–47]. Although these genes are associated with mechanical stress, they are and can be up-regulated by other factors, and thus they alone are not indicative of mechanical stress being the dominate force acting upon the cells.

Although circumstantial, other data suggests that nsEP exposure can induce mechanical stress on cells. Biomarkers of mechanical stress and the observed bioeffects of nsEP exposure are strikingly similar. Markers of mechanical stress include calcium release from the endoplasmic reticulum [48], disruption of the extracellular matrix and cytoskeleton [49–52], increased IP3 production [48], increases in GPCR and MAPK pathway signaling [39,53–55], and the production of reactive oxygen species resulting in oxidative stress [56]. These markers of mechanical stress can be directly and indirectly linked to specific changes in gene expression. Evidence suggests that cells *in vitro* interpret mechanical stress as a mitogen or as a signal to grow/enter the cell cycle [45–47].

The findings presented in this paper provide strong evidence that cells exposed to nsEP experience a stress that is interpreted as being mechanical in nature. It is important to note that we did not control for swelling in these experiments. However, it is unlikely that colloid osmotic swelling of these cells is responsible for the specific changes in mechanical stress associated gene expression. These exposures were performed in full growth medium, and analysis of the forward scattering (FSC-H) data collected from the flow cytometry analysis indicated that swelling did not occur (S7 Fig).

We feel that these findings are important in the field of bioelectrics, because it suggests for the first time that the cells exposed to nsEP experience a mechanical stress of significant amplitude to elicit a specific genetic response. Although it is not explicitly mentioned by or accounted for by other nsEP researchers, mechanical stress, whether caused by electrodeformation [1,5,57], cell swelling [7,8], or through the generation of an acoustic pressure transients generated by nsEP [58], could be responsible for the specific gene expression profiles identified in this study. Pakhomov et al. suggested that future research should focus on many likely mechanisms, including “mechanical stress due to thermoelastic expansion effect.”[59]. The rearrangement of the plasma membrane and possible formation of nanopores witnessed in nsEP is consistent with what is seen in sonoporation. Sonoporation uses ultrasonic waves (i.e. mechanical force) to create holes in the biomembranes of cells and vesicles for the purposes of either delivering or releasing compounds, biomolecules, drugs, etc. [60] Sonoporation causes the cavitation microbubbles, leading to poration by one of the following mechanisms: acoustic micro-streaming, bubble oscillations, or inertial cavitation shock waves [60]. Inertial cavitation shock waves, impart mechanical stress on the plasma membranes of nearby cells leading to poration. Our group has identified and characterized acoustic pressure transients generated by nsEP exposure very similar to those used in sonoporation[58].

Further work is underway to identify the source of the mechanical stress and quantify the amount of force generated by each of the previously identified sources of mechanical stress. The overall goal of future work will be to determine how and to what degree each source of mechanical stress contributes to the nanoporation phenomena or to other cellular reactions. Understanding the mechanisms responsible for the bioeffects associated with nsEP exposure is critical to the continued development and application of this technology.

## Supporting Information

S1 Fig Volcano plots of significant gene expression. U937 cells exposed to thermal stress had 1058 genes significantly up-regulated as compared to sham (≥2 log ratio and p-value ≤ 0.05). 101 genes were significantly down regulated (≤-2 log ratio and p-value ≤ 0.05).
(TIF)

S2 Fig Volcano plots of significant gene expression. Jurkat cells exposed to thermal stress had 1004 genes significantly up-regulated as compared to sham (≥2 log ratio and p-value ≤ 0.05). 158 genes were significantly down regulated (≤-2 log ratio and p-value ≤ 0.05).
(TIF)

S3 Fig U937 validated Genes. (TIF)

S4 Fig U937 validated Genes—GPCR. (TIF)

S5 Fig U937 validated Genes—MAPK. (TIF)

S6 Fig U937 validated Genes—Nuclear Receptors. (TIF)

S7 Fig Forward scattering channel (FSC-H) data collected from flow cytometry. (JPG)

S1 Table Complete list of significant genes changing in U937 cells exposed to nsEP. Genes were selected based on log ratio (≥2, or ≤ -2) and with a p-value of ≤ 0.05.
(DOCX)

S2 Table Complete list of significant genes changing in Jurkat cells exposed to nsEP. Genes were selected based on log ratio (≥2, or ≤ -2) and with a p-value of ≤ 0.05.
(DOCX)

S3 Table Complete list of significant genes changing in U937 cells exposed to 44°C for 40 min. Genes were selected based on log ratio (≥2, or ≤ -2) and with a p-value of ≤ 0.05.
(DOCX)

S4 Table Complete list of significant genes changing in Jurkat exposed to 44°C for 40 min. Genes were selected based on log ratio (≥2, or ≤ -2) and with a p-value of ≤ 0.05.
(DOCX)
