# Supplementary figures and images for: Evaluation of the Genetic Response of U937 and Jurkat Cells to 10-Nanosecond Electrical Pulses (nsEP)
Source: PLoS One. 2016 May 2;11(5):e0154555. doi: 10.1371/journal.pone.0154555 (PMC4852903; doi:10.1371/journal.pone.0154555)

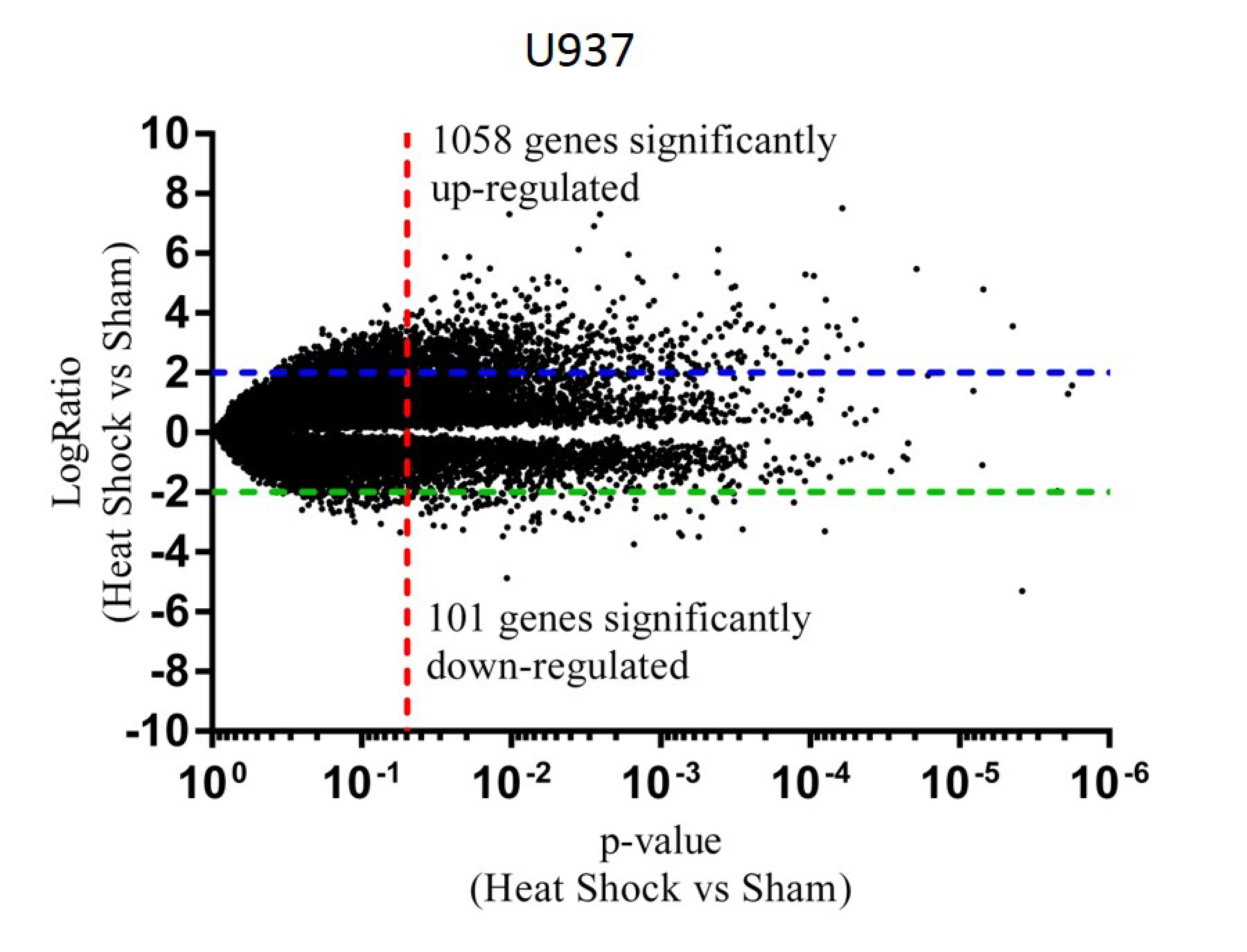

Supplement: S1 Fig — U937 cells exposed to thermal stress had 1058 genes significantly up-regulated as compared to sham (≥2 log ratio and p-value ≤ 0.05). 101 genes were significantly down regulated (≤-2 log ratio and p-value ≤ 0.05). (TIF) [file pone.0154555.s001.tif]

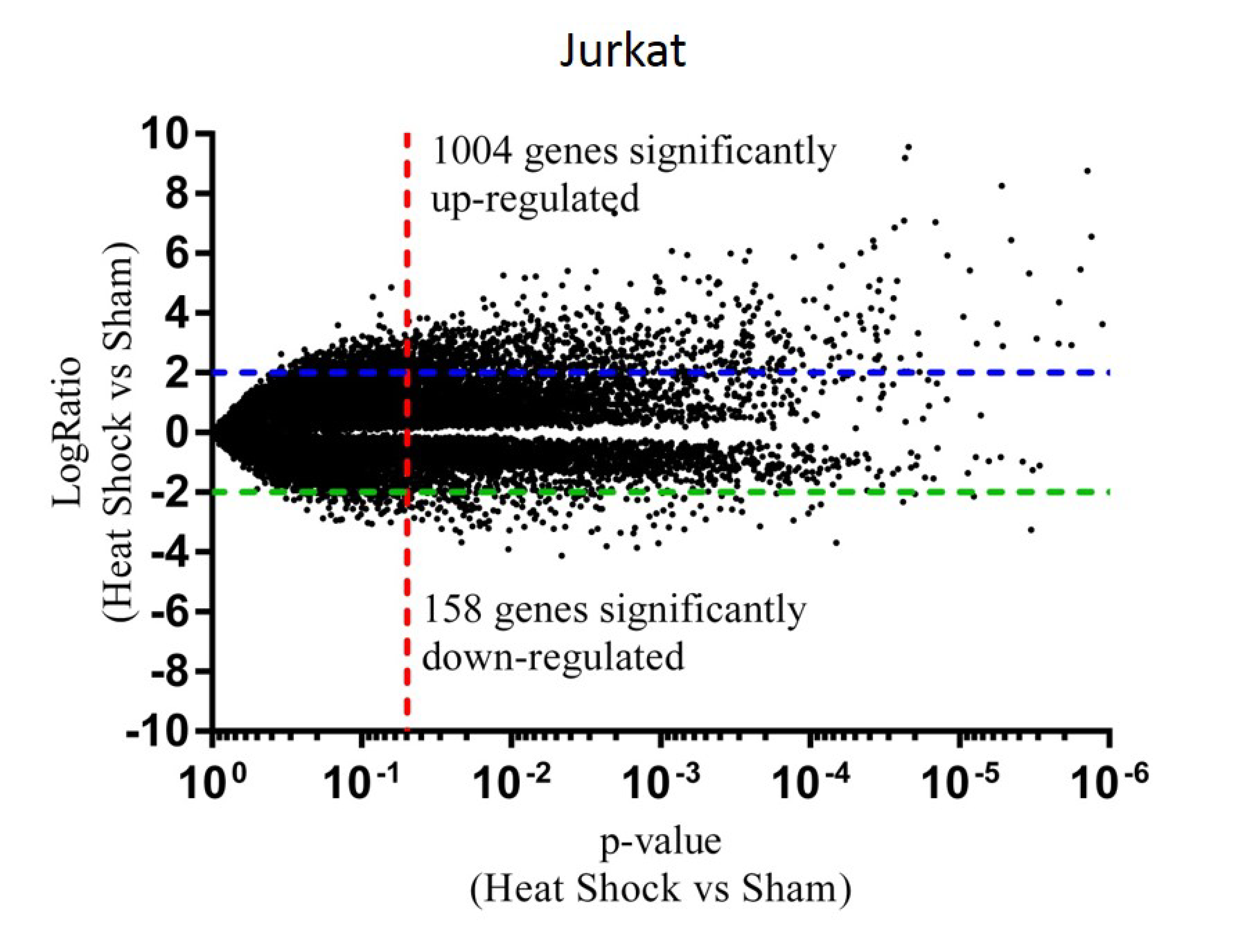

Supplement: S2 Fig — Jurkat cells exposed to thermal stress had 1004 genes significantly up-regulated as compared to sham (≥2 log ratio and p-value ≤ 0.05). 158 genes were significantly down regulated (≤-2 log ratio and p-value ≤ 0.05). (TIF) [file pone.0154555.s002.tif]

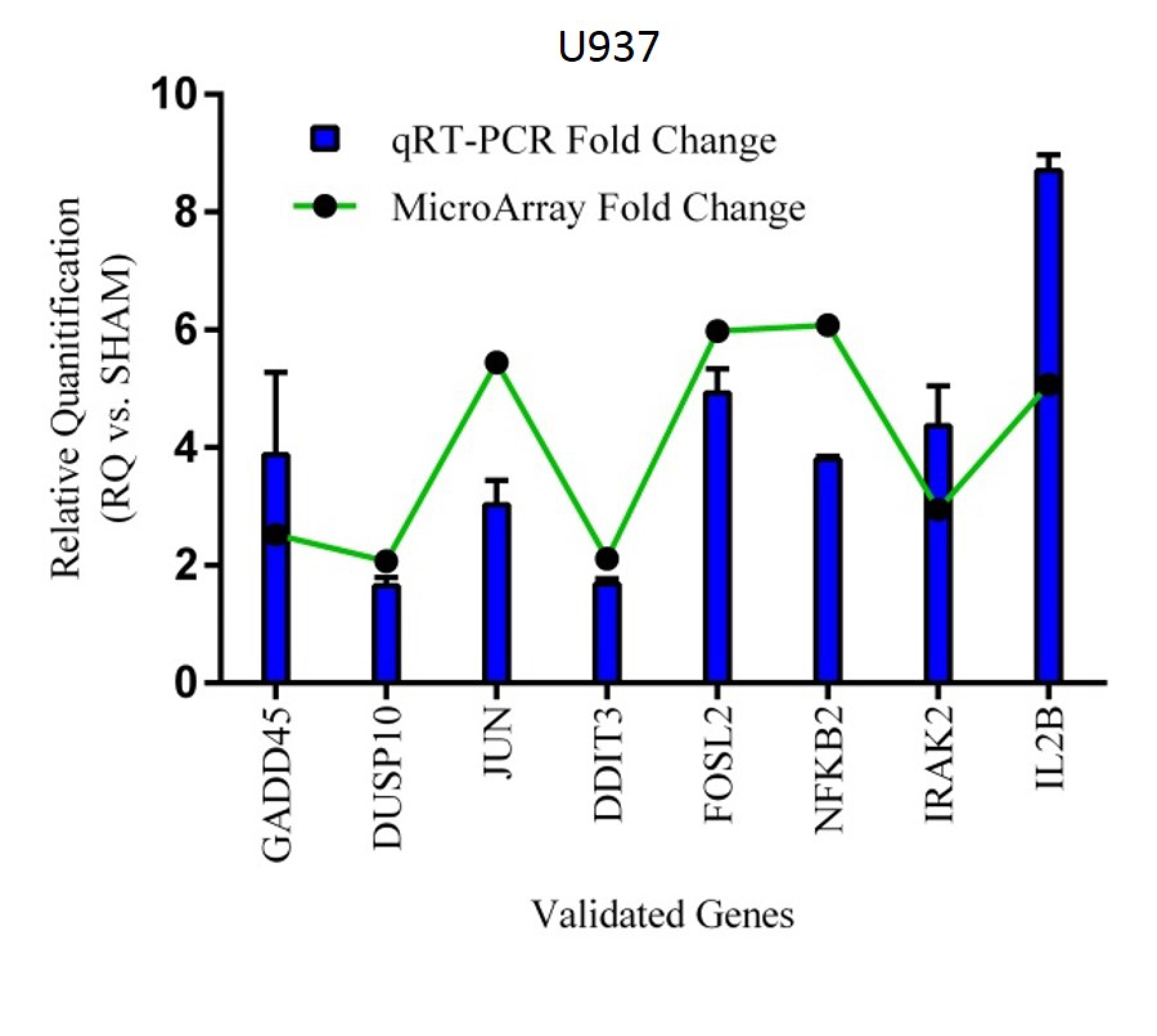

Supplement: S3 Fig — (TIF) [file pone.0154555.s003.tif]

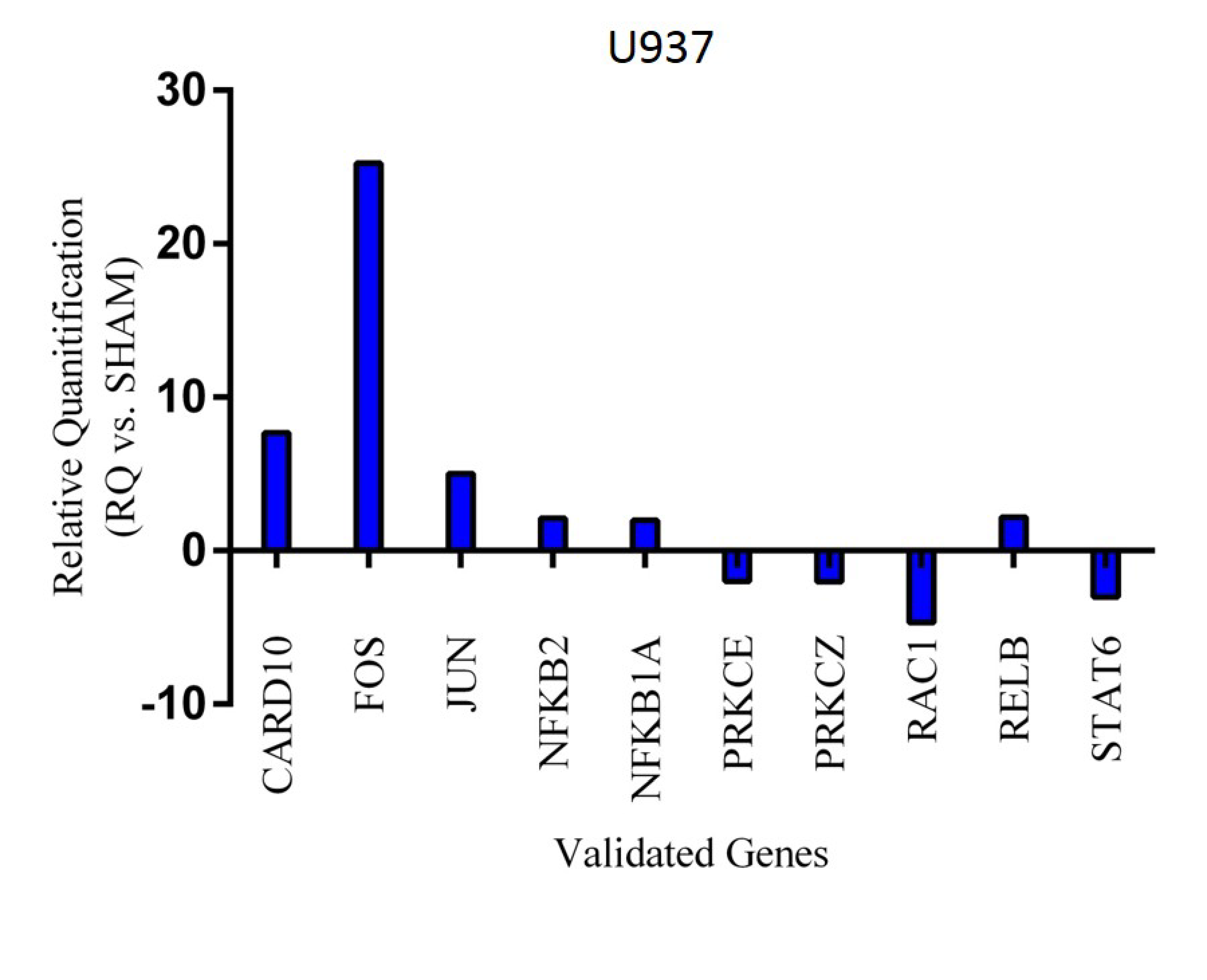

Supplement: S4 Fig — (TIF) [file pone.0154555.s004.tif]

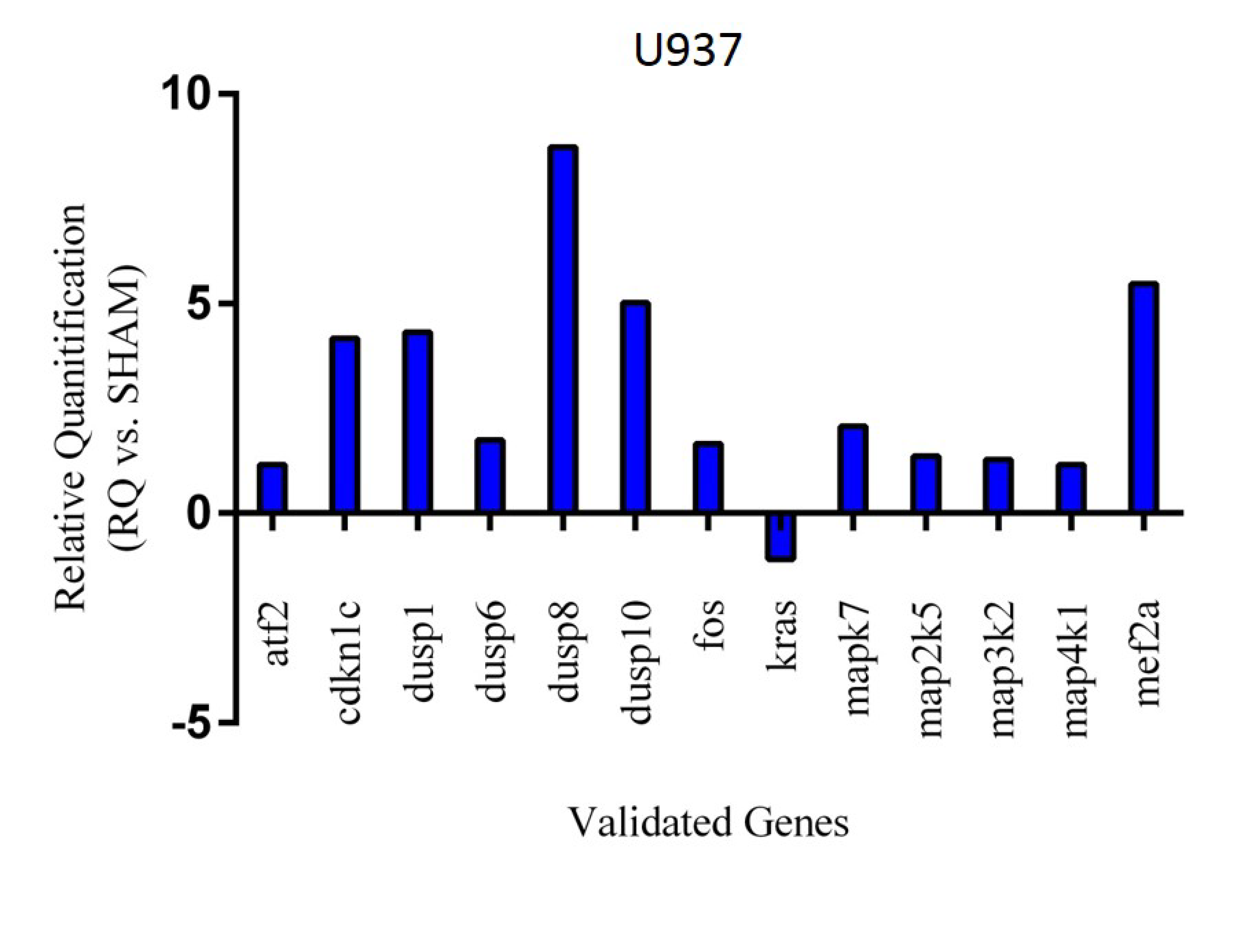

Supplement: S5 Fig — (TIF) [file pone.0154555.s005.tif]

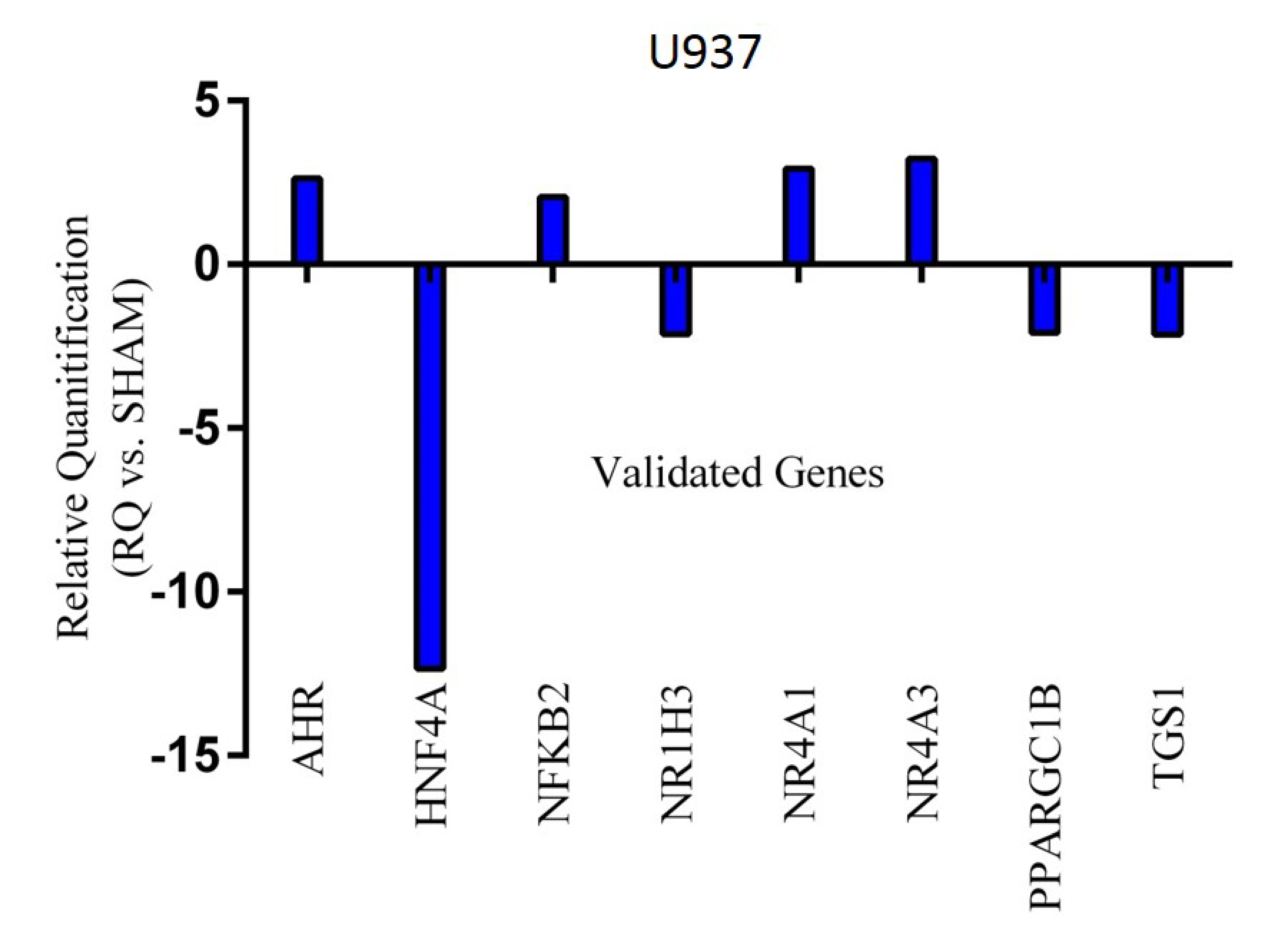

Supplement: S6 Fig — (TIF) [file pone.0154555.s006.tif]

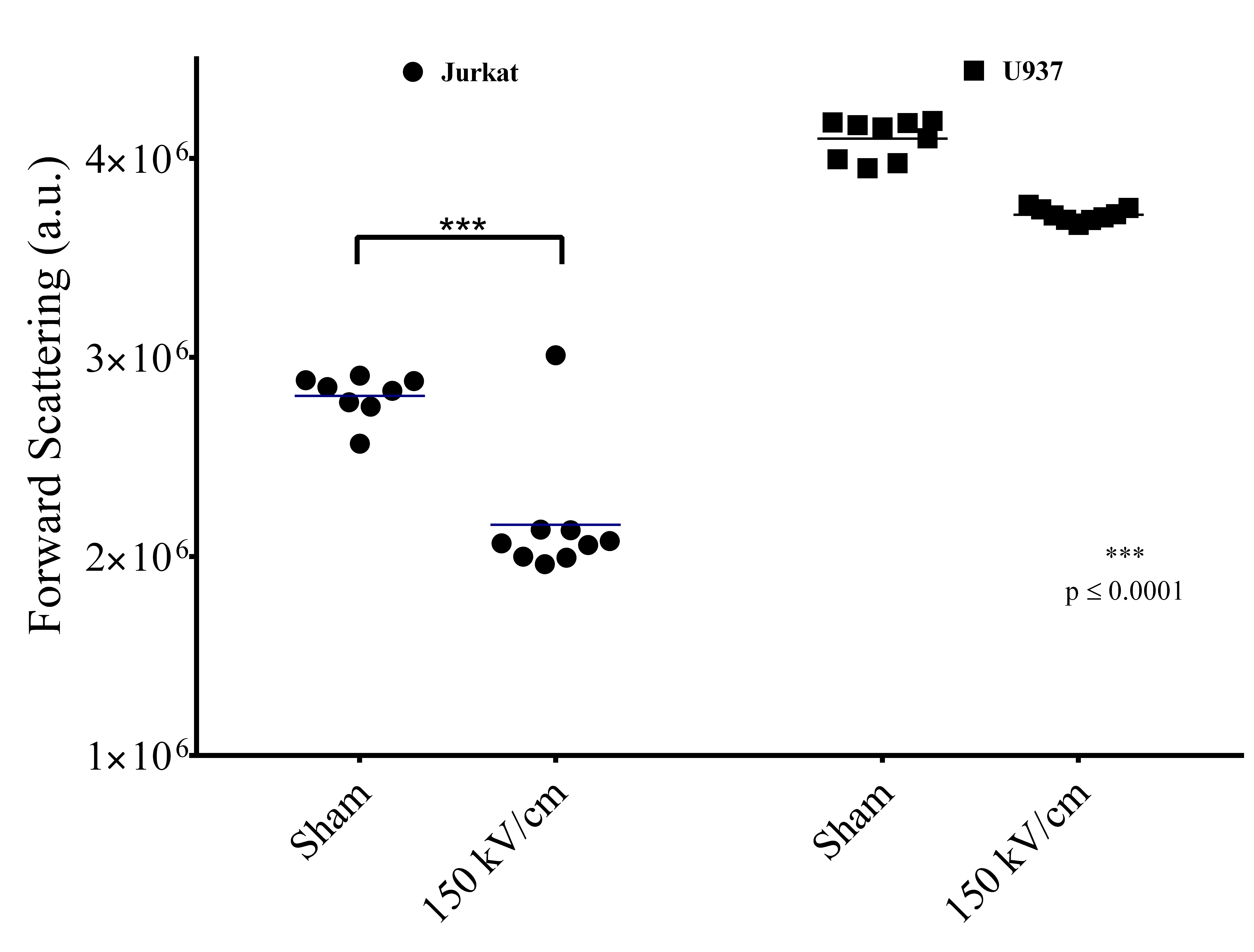

Supplement: S7 Fig — (JPG) [file pone.0154555.s007.jpg]
