# Supplementary material for: Evaluation of the Genetic Response of U937 and Jurkat Cells to 10-Nanosecond Electrical Pulses (nsEP)
Source: PLoS One. 2016 May 2;11(5):e0154555. doi: 10.1371/journal.pone.0154555 (PMC4852903; doi:10.1371/journal.pone.0154555)
Supplement: S1 Table — Genes were selected based on log ratio (≥2, or ≤ -2) and with a p-value of ≤ 0.05. (DOCX) [file pone.0154555.s008.docx]

Supplementary Table 1: Complete list of significant genes changing in U937 cells exposed to 100 pulses of 10 ns at 150kV/cm applied field. Genes were selected based on log ratio (≥2, or ≤ -2) and with a p-value of ≤ 0.05.

| **UniGene ID** | **Gene name** | **Symbol** | **Fold change 150kVnsEP vs. SHAM** | **p-Value**  **150kv nsEP**  **vs. SHAM** |
| --- | --- | --- | --- | --- |
| Hs.446125 | male germ cell-associated kinase | MAK | 4.483 | 0.00081 |
| Hs.155111 | hepatitis A virus cellular receptor 2 | HAVCR2 | 4.453 | 0.00479 |
| Hs.351316 | transmembrane 4 L six family member 1 | TM4SF1 | 4.417 | 0.01954 |
| Hs.491856 | Hypothetical protein FLJ39630 | FLJ39630 | 4.274 | 0.03237 |
| Hs.154057 | matrix metalloproteinase 19 | MMP19 | 4.122 | 0.00389 |
| Hs.370036 | chemokine (C-C motif) receptor 7 | CCR7 | 4.022 | 0.00779 |
| Hs.551526 | Brain-specific protein p25 alpha | TPPP | 3.991 | 0.00274 |
| Hs.267038 | premature ovarian failure, 1B | POF1B | 3.974 | 0.00078 |
| Hs.369063 | Zic family member 2 | ZIC2 | 3.944 | 0.00019 |
| Hs.351316 | transmembrane 4 L six family member 1 | TM4SF1 | 3.896 | 0.00504 |
| Hs.315369 | aquaporin 4 | AQP4 | 3.886 | 0.00755 |
| Hs.129794 | spermatogenesis associated 12 | SPATA12 | 3.745 | 0.00410 |
| Hs.414795 | serine (or cysteine) proteinase inhibitor, clade E | SERPINE1 | 3.692 | 0.00591 |
| Hs.244590 | BTB (POZ) domain containing 3 | BTBD3 | 3.684 | 0.01034 |
| Hs.362807 | interleukin 7 receptor /// interleukin 7 receptor | IL7R | 3.652 | 0.02727 |
| Hs.436298 | epithelial membrane protein 1 | EMP1 | 3.638 | 0.00018 |
| Hs.436550 | Hypothetical protein FLJ33996 | FLJ33996 | 3.606 | 0.02903 |
| Hs.504908 | LIM domain only 3 (rhombotin-like 2) | LMO3 | 3.551 | 0.00110 |
| Hs.91791 | Transmembrane protein 16C | TMEM16C | 3.468 | 0.00232 |
| Hs.249718 | eukaryotic translation initiation factor 4E | EIF4E | 3.417 | 0.01264 |
| Hs.514665 | Hypothetical protein MGC11082 | MGC11082 | 3.404 | 0.00187 |
| Hs.454036 | Similar to syndecan binding protein (syntenin), | --- | 3.388 | 0.00160 |
| Hs.448583 | DKFZP434B061 protein | DKFZP434B061 | 3.384 | 0.00087 |
| Hs.2258 | matrix metalloproteinase 10 (stromelysin 2) | MMP10 | 3.331 | 0.02632 |
| Hs.128753 | receptor tyrosine kinase-like orphan receptor 1 | ROR1 | 3.293 | 0.01011 |
| Hs.513195 | proto-oncogene 8 | HCC-8 | 3.247 | 0.00209 |
| Hs.532144 | histone 1, H3d | HIST1H3D | 3.239 | 0.00057 |
| Hs.445350 | flavin containing monooxygenase 3 | FMO3 | 3.238 | 0.01063 |
| Hs.494173 | Annexin A1 | ANXA1 | 3.236 | 0.02117 |
| Hs.252457 | mevalonate (diphospho) decarboxylase | MVD | 3.225 | 0.00118 |
| Hs.517581 | heme oxygenase (decycling) 1 | HMOX1 | 3.185 | 0.00036 |
| Hs.434606 | Homo sapiens, clone IMAGE:4838684, mRNA | --- | 3.179 | 0.00413 |
| Hs.75256 | regulator of G-protein signalling 1 | RGS1 | 3.152 | 0.00001 |
| Hs.449418 | hypothetical protein LOC286121 | LOC286121 | 3.119 | 0.00002 |
| Hs.130610 | L-threonine dehydrogenase | TDH | 3.106 | 0.00435 |
| Hs.195979 | polycystic kidney disease 1 like 1 | PKD1L1 | 3.101 | 0.00647 |
| Hs.550144 | CDNA clone IMAGE:3621839, partial cds | --- | 3.083 | 0.02312 |
| Hs.2250 | leukemia inhibitory factor (cholinergic differentiation factor) | LIF | 3.058 | 0.00524 |
| Hs.436298 | epithelial membrane protein 1 | EMP1 | 3.045 | 0.00011 |
| Hs.51133 | PTK6 protein tyrosine kinase 6 | PTK6 | 3.038 | 0.01481 |
| Hs.471200 | neuropilin 2 | NRP2 | 3.022 | 0.00311 |
| Hs.144297 | Homo sapiens, clone IMAGE:5311619, mRNA | --- | 3.017 | 0.00017 |
| Hs.75498 | chemokine (C-C motif) ligand 20 | CCL20 | 2.994 | 0.00112 |
| Hs.143929 | C-type lectin domain family 7, member A | CLEC7A | 2.991 | 0.01113 |
| Hs.233936 | LOC440476 | --- | 2.988 | 0.00785 |
| Hs.414795 | serine (or cysteine) proteinase inhibitor, clade E | SERPINE1 | 2.984 | 0.00003 |
| Hs.104633 | agouti related protein homolog (mouse) | AGRP | 2.969 | 0.00230 |
| Hs.169487 | v-maf musculoaponeurotic fibrosarcoma oncogene | MAFB | 2.965 | 0.00305 |
| Hs.351316 | transmembrane 4 L six family member 1 | TM4SF1 | 2.954 | 0.00212 |
| Hs.144333 | lin-7 homolog A (C. elegans) | LIN7A | 2.953 | 0.01102 |
| Hs.390567 | FYN oncogene related to SRC, FGR, YES | FYN | 2.920 | 0.00166 |
| Hs.220950 | Forkhead box O3A | FOXO3A | 2.914 | 0.00190 |
| Hs.505007 | SRY (sex determining region Y)-box 5 | SOX5 | 2.900 | 0.00550 |
| Hs.44277 | Hypothetical protein MGC14816 | MGC14816 | 2.899 | 0.03743 |
| Hs.524438 | keratin 6B | KRT6B | 2.897 | 0.00793 |
| Hs.143873 | S100 calcium binding protein A10 | S100A10 | 2.891 | 0.02345 |
| Hs.370699 | hypothetical protein LOC284801 | LOC284801 | 2.887 | 0.00089 |
| Hs.433297 | Chromosome 10 open reading frame 75 | C10orf75 | 2.880 | 0.04607 |
| Hs.162877 | Protein kinase C and casein kinase substrate | PACSIN2 | 2.870 | 0.00000 |
| Hs.169797 | Bol, boule-like (Drosophila) | BOLL | 2.868 | 0.02140 |
| Hs.446388 | CDNA FLJ41489 fis, clone BRTHA2004582 | --- | 2.865 | 0.02960 |
| Hs.549112 | pleckstrin homology-like domain, | PHLDA1 | 2.861 | 0.00022 |
| Hs.317659 | developmental pluripotency associated 4 | DPPA4 | 2.848 | 0.04391 |
| Hs.480615 | synaptopodin 2 | SYNPO2 | 2.833 | 0.01103 |
| Hs.477425 | solute carrier family 12, member 8 | SLC12A8 | 2.814 | 0.01663 |
| Hs.193122 | Fc fragment of IgA, receptor for | FCAR | 2.806 | 0.00665 |
| Hs.258314 | TNFRSF1A modulator | BRE | 2.801 | 0.02944 |
| Hs.185774 | eyes absent homolog 3 (Drosophila) | EYA3 | 2.800 | 0.04802 |
| Hs.465506 | phosphatidic acid phosphatase type 2C | PPAP2C | 2.771 | 0.00170 |
| Hs.66378 | HECT domain containing 2 | HECTD2 | 2.767 | 0.01259 |
| Hs.484885 | CDNA clone IMAGE:4514712, partial cds | --- | 2.763 | 0.00010 |
| Hs.385684 | CDNA clone IMAGE:4830552, partial cds | --- | 2.758 | 0.00211 |
| Hs.167046 | adenosine A2b receptor | ADORA2B | 2.755 | 0.03151 |
| Hs.549112 | pleckstrin homology-like domain | PHLDA1 | 2.754 | 0.00001 |
| Hs.128453 | frizzled-related protein | FRZB | 2.754 | 0.02448 |
| Hs.484779 | Homeobox C14 | LOC360030 | 2.752 | 0.00439 |
| Hs.497518 | Hypothetical protein DKFZp761N1114 | DKFZp761N1114 | 2.744 | 0.02623 |
| Hs.83169 | matrix metalloproteinase 1 | MMP1 | 2.744 | 0.00577 |
| Hs.533710 | fibronectin leucine rich transmembrane protein 2 | FLRT2 | 2.740 | 0.03154 |
| Hs.529892 | sequestosome 1 | SQSTM1 | 2.737 | 0.03563 |
| Hs.470117 | Low density lipoprotein-related protein 1B | LRP1B | 2.733 | 0.02973 |
| Hs.514814 | acyl-malonyl condensing enzyme 1-like 2 | AMAC1L2 | 2.733 | 0.00190 |
| Hs.445351 | Lectin, galactoside-binding, soluble, 1 ( | LGALS1 | 2.713 | 0.02200 |
| Hs.433445 | jagged 2 | JAG2 | 2.707 | 0.00476 |
| Hs.93836 | deafness, autosomal recessive 31 | DFNB31 | 2.689 | 0.00538 |
| Hs.547596 | Ig rearranged lambda-chain gene V-Jl2/l3-region | --- | 2.684 | 0.00220 |
| Hs.128056 | KIAA0241 protein | KIAA0241 | 2.681 | 0.00745 |
| Hs.527077 | T cell receptor, clone IGRB40 | --- | 2.681 | 0.00165 |
| Hs.385635 | Homo sapiens, clone IMAGE:4828073, mRNA | --- | 2.680 | 0.02563 |
| Hs.517155 | transmembrane, prostate androgen induced RNA | TMEPAI | 2.667 | 0.03042 |
| Hs.144348 | outer dense fiber of sperm tails 3-like 1 | ODF3L1 | 2.658 | 0.00399 |
| Hs.12723 | contactin 3 (plasmacytoma associated) | CNTN3 | 2.658 | 0.02216 |
| --- | zinc finger protein 322B | ZNF322B | 2.657 | 0.00659 |
| Hs.549223 | scinderin | SCIN | 2.654 | 0.00283 |
| Hs.624 | interleukin 8 | IL8 | 2.651 | 0.01917 |
| Hs.467236 | zinc finger protein 160 | ZNF160 | 2.646 | 0.01947 |
| Hs.251526 | chemokine (C-C motif) ligand 7 | CCL7 | 2.644 | 0.00872 |
| Hs.434375 | protein tyrosine phosphatase, receptor type, B | PTPRB | 2.644 | 0.00589 |
| Hs.548398 | CDNA clone IMAGE:5295023, partial cds | --- | 2.632 | 0.01804 |
| Hs.387262 | MCF.2 cell line derived transforming sequence | MCF2 | 2.632 | 0.02785 |
| Hs.414795 | serine (or cysteine) proteinase inhibitor, clade E | SERPINE1 | 2.625 | 0.00391 |
| Hs.433728 | mitogen-activated protein kinase 4 | MAPK4 | 2.616 | 0.01059 |
| Hs.429294 | ATP-binding cassette, sub-family A | ABCA1 | 2.613 | 0.00053 |
| Hs.370699 | hypothetical protein LOC284801 | LOC284801 | 2.610 | 0.00022 |
| Hs.505033 | v-Ki-ras2 Kirsten rat sarcoma viral oncogene homolog | KRAS | 2.607 | 0.04322 |
| Hs.73090 | nuclear factor of kappa light polypeptide gene enhancer in B-cells | NFKB2 | 2.604 | 0.00011 |
| Hs.516484 | S100 calcium binding protein A2 | S100A2 | 2.600 | 0.00279 |
| Hs.296323 | serum/glucocorticoid regulated kinase | SGK | 2.595 | 0.00007 |
| Hs.443685 | Hypothetical LOC147941 | --- | 2.592 | 0.00055 |
| Hs.494173 | annexin A1 | ANXA1 | 2.590 | 0.00509 |
| Hs.82906 | myeloproliferative leukemia virus oncogene | MPL | 2.589 | 0.03821 |
| Hs.374262 | Cofactor required for Sp1 transcriptional activation, subunit 8, | CRSP8 | 2.586 | 0.00088 |
| Hs.418055 | ataxia, cerebellar, Cayman type (caytaxin) | ATCAY | 2.583 | 0.00166 |
| Hs.220971 | FOS-like antigen 2 | FOSL2 | 2.580 | 0.00045 |
| Hs.220971 | FOS-like antigen 2 | FOSL2 | 2.579 | 0.02742 |
| Hs.362807 | Interleukin 7 receptor | IL7R | 2.577 | 0.01344 |
| Hs.184085 | crystallin, alpha A | CRYAA | 2.566 | 0.00090 |
| Hs.482730 | EGF-like repeats and discoidin I-like domains 3 | EDIL3 | 2.563 | 0.00888 |
| Hs.127735 | ankyrin repeat and SOCS box-containing 4 | ASB4 | 2.559 | 0.00399 |
| Hs.98890 | ring finger protein 180 | RNF180 | 2.555 | 0.03816 |
| Hs.482660 | zinc finger, FYVE domain containing 16 | ZFYVE16 | 2.552 | 0.02638 |
| Hs.82045 | Midkine (neurite growth-promoting factor 2) | MDK | 2.552 | 0.00581 |
| Hs.511486 | Similar to 60S ribosomal protein L37a | --- | 2.550 | 0.00180 |
| Hs.157344 | MRNA; cDNA DKFZp667A182 | --- | 2.540 | 0.02287 |
| Hs.482301 | Hypothetical protein FLJ13611 | FLJ13611 | 2.537 | 0.03613 |
| Hs.189585 | chromosome 21 open reading frame 106 | C21orf106 | 2.531 | 0.00092 |
| Hs.169487 | v-maf musculoaponeurotic fibrosarcoma oncogene homolog B | MAFB | 2.528 | 0.00198 |
| Hs.535723 | BC048124 | LOC348808 | 2.521 | 0.01080 |
| Hs.416862 | Homo sapiens, clone IMAGE:4822577, mRNA | --- | 2.517 | 0.02579 |
| Hs.133042 | Transcribed locus | --- | 2.514 | 0.03353 |
| Hs.100431 | chemokine (C-X-C motif) ligand 13 | CXCL13 | 2.513 | 0.04180 |
| Hs.61265 | family with sequence similarity 3, member D | FAM3D | 2.510 | 0.01071 |
| Hs.467338 | potassium inwardly-rectifying channel, subfamily J, member 13 | KCNJ13 | 2.509 | 0.01228 |
| Hs.283022 | triggering receptor expressed on myeloid cells 1 | TREM1 | 2.503 | 0.00147 |
| Hs.449209 | CDNA FLJ12301 fis, clone MAMMA1001858 | --- | 2.503 | 0.01440 |
| Hs.24684 | arrestin domain containing 3 | ARRDC3 | 2.502 | 0.01263 |
| Hs.8004 | kalirin, RhoGEF kinase | KALRN | 2.500 | 0.00283 |
| Hs.272804 | chromosome X open reading frame 48 | CXorf48 | 2.498 | 0.00486 |
| Hs.413416 | jumonji domain containing 1C | JMJD1C | 2.493 | 0.00065 |
| Hs.120591 | CDNA FLJ35632 fis, clone SPLEN2011678 | --- | 2.489 | 0.03657 |
| Hs.304792 | Proline synthetase co-transcribed homolog | PROSC | 2.487 | 0.00541 |
| Hs.128069 | hypothetical protein FLJ25660 | FLJ25660 | 2.483 | 0.00613 |
| Hs.79092 | protein tyrosine phosphatase, non-receptor type 5 | PTPN5 | 2.477 | 0.01181 |
| Hs.127383 | Hypothetical gene supported by AK129597 | --- | 2.477 | 0.02290 |
| Hs.309403 | ciliary rootlet coiled-coil, rootletin | CROCC | 2.468 | 0.02563 |
| Hs.279583 | DORA reverse strand protein 1 | DREV1 | 2.465 | 0.04447 |
| Hs.505033 | v-Ki-ras2 Kirsten rat sarcoma viral oncogene homolog | KRAS | 2.463 | 0.02171 |
| Hs.213289 | low density lipoprotein receptor | LDLR | 2.463 | 0.03855 |
| Hs.292843 | prostaglandin F receptor (FP) | PTGFR | 2.460 | 0.01388 |
| Hs.533317 | vimentin | VIM | 2.458 | 0.00071 |
| Hs.117545 | Phosphodiesterase 4D, cAMP-specific | PDE4D | 2.455 | 0.02799 |
| Hs.416061 | phosphodiesterase 1A, calmodulin-dependent | PDE1A | 2.445 | 0.04536 |
| Hs.525704 | v-jun sarcoma virus 17 oncogene homolog | JUN | 2.444 | 0.00009 |
| Hs.349077 | poly(A)-specific ribonuclease (PARN)-like domain containing 1 | PNLDC1 | 2.443 | 0.00377 |
| Hs.484885 | CDNA clone IMAGE:4514712, partial cds | --- | 2.436 | 0.00096 |
| Hs.443625 | collagen, type III, alpha 1 | COL3A1 | 2.421 | 0.01398 |
| Hs.24030 | Solute carrier family 31, member 2 | SLC31A2 | 2.420 | 0.02825 |
| Hs.59093 | calcium channel, voltage-dependent, beta 2 subunit | CACNB2 | 2.412 | 0.04091 |
| Hs.479853 | EPH receptor A5 | EPHA5 | 2.402 | 0.00668 |
| Hs.386365 | homeo box D4 | HOXD4 | 2.398 | 0.04197 |
| Hs.383008 | hypothetical protein LOC339834 | LOC339834 | 2.379 | 0.00599 |
| Hs.369982 | insulin-like growth factor binding protein 5 | IGFBP5 | 2.376 | 0.03960 |
| Hs.98594 | Rho guanine nucleotide exchange factor (GEF) 10 | ARHGEF10 | 2.375 | 0.00555 |
| Hs.2131 | arginine vasopressin receptor 1A | AVPR1A | 2.372 | 0.04308 |
| Hs.17267 | chromosome 9 open reading frame 93 | C9orf93 | 2.372 | 0.02214 |
| Hs.213389 | Golgi autoantigen, golgin subfamily b, macrogolgin | GOLGB1 | 2.367 | 0.04460 |
| Hs.552605 | chromosome 20 open reading frame 128 | C20orf128 | 2.362 | 0.00046 |
| Hs.306443 | MRNA; cDNA DKFZp434C2021 | --- | 2.362 | 0.00043 |
| Hs.386567 | guanylate binding protein 2, interferon-inducible | GBP2 | 2.362 | 0.01820 |
| Hs.531687 | hypothetical protein LOC151121 | LOC151121 | 2.361 | 0.04800 |
| Hs.493793 | hypothetical protein LOC158381 | LOC158381 | 2.357 | 0.00042 |
| Hs.387207 | Sarcoglycan, delta (35kDa dystrophin-associated glycoprotein) | SGCD | 2.352 | 0.00531 |
| Hs.126256 | interleukin 1, beta | IL1B | 2.343 | 0.00136 |
| Hs.464137 | acyl-Coenzyme A oxidase 1, palmitoyl | ACOX1 | 2.342 | 0.03410 |
| Hs.149363 | Homo sapiens, clone IMAGE:5266794, mRNA | --- | 2.341 | 0.01545 |
| Hs.529571 | retinol binding protein 1, cellular | RBP1 | 2.339 | 0.00746 |
| Hs.291196 | ATPase, Na+/K+ transporting, beta 1 polypeptide | ATP1B1 | 2.338 | 0.01909 |
| Hs.122523 | Staphylococcal nuclease domain containing 1 | SND1 | 2.336 | 0.00061 |
| Hs.301974 | low density lipoprotein-related protein 12 | LRP12 | 2.336 | 0.00164 |
| Hs.283148 | RAB38, member RAS oncogene family | RAB38 | 2.333 | 0.00241 |
| Hs.253146 | Pleckstrin homology domain containing, family A member 6 | PLEKHA6 | 2.331 | 0.02031 |
| Hs.514665 | hypothetical protein MGC11082 | MGC11082 | 2.328 | 0.01434 |
| Hs.518567 | Transcribed locus | --- | 2.324 | 0.01814 |
| Hs.21658 | Transcribed locus | --- | 2.323 | 0.04262 |
| Hs.549182 | PR domain containing 11 | PRDM11 | 2.322 | 0.04884 |
| Hs.6682 | solute carrier family 7, member 11 | SLC7A11 | 2.309 | 0.00401 |
| Hs.193122 | Fc fragment of IgA, receptor for | FCAR | 2.309 | 0.00490 |
| Hs.534309 | cysteine-rich protein 2 | CRIP2 | 2.307 | 0.00958 |
| Hs.75765 | chemokine (C-X-C motif) ligand 2 | CXCL2 | 2.305 | 0.00143 |
| Hs.371249 | pleiotrophin | PTN | 2.303 | 0.00831 |
| Hs.547764 | MRNA full length insert cDNA clone | --- | 2.302 | 0.00330 |
| Hs.436124 | hypothetical protein LOC154822 | LOC154822 | 2.302 | 0.00332 |
| Hs.380325 | folate hydrolase (prostate-specific membrane antigen) 1 | FOLH1 | 2.300 | 0.02604 |
| Hs.502867 | EH domain binding protein 1-like 1 | EHBP1L1 | 2.296 | 0.04740 |
| Hs.490330 | natriuretic peptide receptor A/guanylate cyclase A | NPR1 | 2.291 | 0.01183 |
| Hs.485489 | Chloride intracellular channel 5 | CLIC5 | 2.283 | 0.01403 |
| Hs.10041 | CDNA FLJ37509 fis, clone BRCAN1000065 | --- | 2.277 | 0.03766 |
| Hs.481068 | Full length insert cDNA clone YQ50C11 | --- | 2.274 | 0.02754 |
| Hs.528335 | hypothetical protein FLJ25477 | FLJ25477 | 2.273 | 0.00314 |
| Hs.194695 | DIRAS family, GTP-binding RAS-like 3 | DIRAS3 | 2.272 | 0.00680 |
| Hs.108614 | Cytoplasmic linker associated protein 2 | CLASP2 | 2.270 | 0.02960 |
| Hs.193122 | Fc fragment of IgA, receptor for | FCAR | 2.269 | 0.00968 |
| Hs.119983 | mannan-binding lectin serine protease 2 | MASP2 | 2.265 | 0.02826 |
| Hs.269364 | Rhesus blood group, D antigen | RHD | 2.263 | 0.01242 |
| --- | triggering receptor expressed on myeloid cells-like 3 | TREML3 | 2.262 | 0.04761 |
| Hs.90572 | PTK7 protein tyrosine kinase 7 | PTK7 | 2.262 | 0.03406 |
| Hs.134830 | Collagen, type VIII, alpha 1 | COL8A1 | 2.260 | 0.00604 |
| Hs.85155 | zinc finger protein 36, C3H type-like 1 | ZFP36L1 | 2.259 | 0.00054 |
| Hs.147062 | cyclic nucleotide gated channel beta 1 | CNGB1 | 2.257 | 0.00839 |
| Hs.66194 | CDNA clone IMAGE:4611512, partial cds | --- | 2.257 | 0.00167 |
| Hs.307926 | G protein interaction factor 2-like mRNA sequence | --- | 2.253 | 0.00003 |
| Hs.464391 | tubulin-specific chaperone d | TBCD | 2.253 | 0.00635 |
| Hs.446309 | glutathione S-transferase A1 | GSTA1 | 2.250 | 0.03110 |
| Hs.442609 | mitochondrial ribosomal protein L38 | MRPL38 | 2.245 | 0.01187 |
| Hs.364600 | chromosome 21 open reading frame 128 | C21orf128 | 2.245 | 0.01494 |
| Hs.446336 | paxillin | PXN | 2.241 | 0.02448 |
| Hs.530311 | lipocalin 1 (tear prealbumin) | LCN1 | 2.238 | 0.04484 |
| Hs.14691 | Transcribed locus | --- | 2.238 | 0.00230 |
| Hs.124366 | Bobby sox homolog (Drosophila) | BBX | 2.232 | 0.01799 |
| Hs.156832 | Neuronal PAS domain protein 2 | NPAS2 | 2.230 | 0.01178 |
| Hs.476075 | Homo sapiens, clone IMAGE:5298883, mRNA | --- | 2.221 | 0.04146 |
| Hs.443491 | Transcribed locus | --- | 2.219 | 0.04266 |
| Hs.532711 | testis-specific serine kinase 6 | TSSK6 | 2.216 | 0.00177 |
| Hs.531188 | olfactory receptor, family 4, subfamily D, member 1 | OR4D1 | 2.215 | 0.00021 |
| Hs.432706 | hypothetical protein DKFZp434D2328 | LOC91526 | 2.214 | 0.00563 |
| Hs.76556 | protein phosphatase 1, regulatory subunit 15A | PPP1R15A | 2.210 | 0.00025 |
| Hs.547104 | Homo sapiens, clone IMAGE:4823248, mRNA | --- | 2.206 | 0.00658 |
| Hs.278488 | myosin, heavy polypeptide 13, skeletal muscle | MYH13 | 2.203 | 0.01415 |
| Hs.466871 | plasminogen activator, urokinase receptor | PLAUR | 2.203 | 0.00020 |
| Hs.512440 | Homo sapiens, clone IMAGE:4861097, mRNA | --- | 2.201 | 0.02818 |
| Hs.514107 | chemokine (C-C motif) ligand 3 | CCL3 | 2.198 | 0.00323 |
| Hs.143961 | chemokine (C-C motif) ligand 18 | CCL18 | 2.192 | 0.00964 |
| Hs.624 | interleukin 8 | IL8 | 2.192 | 0.01803 |
| Hs.385570 | Homo sapiens, clone IMAGE:4830126, mRNA | --- | 2.190 | 0.04004 |
| Hs.478588 | B-cell CLL/lymphoma 6 (zinc finger protein 51) | BCL6 | 2.184 | 0.00780 |
| Hs.547730 | Transcribed locus | --- | 2.181 | 0.04072 |
| Hs.503451 | LOC440885 | LOC440885 | 2.181 | 0.00234 |
| Hs.212606 | glutaminase 2 (liver, mitochondrial) | GLS2 | 2.180 | 0.03190 |
| Hs.272787 | Hypothetical protein FLJ20184 | FLJ20184 | 2.178 | 0.00170 |
| Hs.435051 | Cyclin-dependent kinase inhibitor 2D (p19, inhibits CDK4) | CDKN2D | 2.177 | 0.04341 |
| Hs.97432 | protein kinase C, epsilon | PRKCE | 2.176 | 0.02355 |
| Hs.534352 | syntaxin binding protein 2 | STXBP2 | 2.172 | 0.01997 |
| Hs.458593 | similar to ubiquitin associated protein | LOC390595 | 2.171 | 0.04995 |
| Hs.262960 | transient recep. potential cation channel, subfamily C, member 4 | TRPC4 | 2.168 | 0.00198 |
| Hs.190284 | Smith-Magenis syndrome chromosome region, candidate 6 | SREBF1 | 2.167 | 0.00262 |
| Hs.439630 | Chromosome 5 open reading frame 12 | C5orf12 | 2.167 | 0.04145 |
| Hs.81328 | nuclear factor of kappa light polypeptide gene enhancer in B-cells | NFKBIA | 2.166 | 0.00225 |
| Hs.24587 | embryonal Fyn-associated substrate | EFS | 2.163 | 0.00252 |
| Hs.407510 | Homo sapiens, clone IMAGE:5269873, mRNA | --- | 2.156 | 0.01543 |
| Hs.24950 | Regulator of G-protein signalling 5 | RGS5 | 2.153 | 0.00161 |
| Hs.471162 | Ras association (RalGDS/AF-6) and pleckstrin homology domains 1 | RAPH1 | 2.151 | 0.02669 |
| Hs.289044 | Similar to RIKEN cDNA 2310016C16 | LOC493869 | 2.150 | 0.04445 |
| Hs.546573 | Hypothetical LOC388638 | --- | 2.149 | 0.00232 |
| Hs.546263 | killer cell immunoglobulin-like receptor, three domains, | KIR3DL2 | 2.146 | 0.04449 |
| Hs.134192 | CDNA FLJ27467 fis, clone ADG06160 | --- | 2.143 | 0.04783 |
| Hs.36980 | melanoma antigen family A, | MAGEA2 | 2.141 | 0.02781 |
| Hs.406781 | MRNA; cDNA DKFZp434M0835 | --- | 2.140 | 0.00745 |
| Hs.403933 | F-box protein 32 | FBXO32 | 2.137 | 0.00147 |
| Hs.485892 | Zinc finger protein 292 | ZNF292 | 2.133 | 0.02991 |
| Hs.405755 | dihydrodiol dehydrogenase (dimeric) | DHDH | 2.123 | 0.03760 |
| Hs.408676 | hypothetical protein MGC33887 | MGC33887 | 2.122 | 0.02696 |
| Hs.477869 | phospholipid scramblase 4 | PLSCR4 | 2.120 | 0.02673 |
| Hs.376950 | Hypothetical gene supported by AL832797 | --- | 2.118 | 0.03731 |
| Hs.385546 | homeobox C14 | LOC360030 | 2.107 | 0.02395 |
| Hs.511915 | enolase 2 (gamma, neuronal) | ENO2 | 2.106 | 0.00138 |
| Hs.409602 | Sulfatase 1 | SULF1 | 2.104 | 0.00757 |
| Hs.428214 | mastermind-like 2 (Drosophila) | MAML2 | 2.099 | 0.01422 |
| Hs.531081 | Lectin, galactoside-binding, soluble, 3 (galectin 3) | LGALS3 | 2.098 | 0.02110 |
| Hs.444947 | tribbles homolog 1 (Drosophila) | TRIB1 | 2.098 | 0.00374 |
| Hs.436298 | epithelial membrane protein 1 | EMP1 | 2.097 | 0.01083 |
| Hs.129543 | CDNA FLJ35982 fis, clone TESTI2013604 | --- | 2.096 | 0.04491 |
| Hs.434660 | Hypothetical protein LOC285045 | LOC285045 | 2.091 | 0.04571 |
| Hs.172928 | collagen, type I, alpha 1 | COL1A1 | 2.086 | 0.04424 |
| Hs.407709 | DRE1 protein | DRE1 | 2.084 | 0.04450 |
| Hs.126706 | 1-aminocyclopropane-1-carboxylate synthase | PHACS | 2.082 | 0.01555 |
| Hs.491359 | lamin A/C | LMNA | 2.080 | 0.00313 |
| Hs.518668 | CDNA FLJ37609 fis, clone BRCOC2011010. | --- | 2.073 | 0.00256 |
| Hs.550535 | Hypothetical protein FLJ13941 | FLJ13941 | 2.073 | 0.02539 |
| Hs.549132 | olfactory receptor, family 2, subfamily L, member 2 | OR2L2 | 2.072 | 0.00230 |
| Hs.528664 | potassium channel, subfamily K, member 15 | KCNK15 | 2.070 | 0.01655 |
| Hs.534498 | H2A histone family, member B3 | H2AFB3 | 2.068 | 0.04593 |
| Hs.298654 | dual specificity phosphatase 6 | DUSP6 | 2.064 | 0.00891 |
| Hs.409139 | LOC440312 | --- | 2.064 | 0.03582 |
| Hs.49407 | regenerating islet-derived 1 alpha | REG1A | 2.063 | 0.04034 |
| Hs.129126 | hypothetical protein LOC92922 | MGC10992 | 2.060 | 0.03332 |
| Hs.471200 | neuropilin 2 | NRP2 | 2.057 | 0.02851 |
| Hs.474880 | Hypothetical LOC388903 | --- | 2.056 | 0.03292 |
| Hs.552711 | CDNA FLJ11009 fis, clone PLACE1003108 | --- | 2.055 | 0.00050 |
| --- | hypothetical protein MGC12916 | MGC12916 | 2.053 | 0.04733 |
| Hs.208206 | TNFAIP3 interacting protein 3 | TNIP3 | 2.053 | 0.04569 |
| Hs.171285 | hypothetical protein LOC283887 | LOC283887 | 2.052 | 0.00168 |
| Hs.72307 | Transcribed locus | --- | 2.052 | 0.04274 |
| Hs.129895 | T-box 3 (ulnar mammary syndrome) | TBX3 | 2.052 | 0.00601 |
| Hs.408336 | otoancorin | OTOA | 2.046 | 0.02076 |
| Hs.185677 | Neural precursor cell expressed, developmentally down-regulated | NEDD4L | 2.045 | 0.02001 |
| --- | interleukin 4 induced 1 | IL4I1 | 2.043 | 0.01027 |
| Hs.220971 | FOS-like antigen 2 | FOSL2 | 2.040 | 0.00155 |
| Hs.89890 | pyruvate carboxylase | PC | 2.035 | 0.04286 |
| Hs.76095 | immediate early response 3 | IER3 | 2.033 | 0.00020 |
| Hs.4221 | hypothetical protein DKFZp761H039 | DKFZp761H039 | 2.031 | 0.00092 |
| Hs.130031 | triple functional domain (PTPRF interacting) | TRIO | 2.028 | 0.02110 |
| Hs.519514 | tripartite motif-containing 36 | TRIM36 | 2.018 | 0.03311 |
| Hs.368611 | ST3 beta-galactoside alpha-2,3-sialyltransferase 2 | ST3GAL2 | 2.018 | 0.01547 |
| Hs.220971 | FOS-like antigen 2 | FOSL2 | 2.008 | 0.00132 |
| Hs.369982 | insulin-like growth factor binding protein 5 | IGFBP5 | 2.007 | 0.02966 |
| Hs.377972 | Chromosome 13 open reading frame 21 | C13orf21 | 2.004 | 0.02375 |
| Hs.446484 | Casein kinase 2, alpha 1 polypeptide | CSNK2A1 | 2.001 | 0.03565 |
|  |  |  |  |  |
| Hs.476610 | CDNA FLJ12258 fis, clone MAMMA1001510 | --- | -2.013 | 0.04299 |
| Hs.503178 | spectrin, beta, non-erythrocytic 1 | SPTBN1 | -2.015 | 0.02397 |
| Hs.339846 | cardiac-MyBP-C associated Ca/CaM kinase | MLCK | -2.023 | 0.03829 |
| Hs.370359 | Nuclear factor I/B | NFIB | -2.024 | 0.01807 |
| Hs.377972 | Chromosome 13 open reading frame 21 | C13orf21 | -2.024 | 0.00460 |
| Hs.483701 | potassium channel tetramerisation domain containing 16 | KCTD16 | -2.029 | 0.02062 |
| Hs.98798 | hypothetical protein MGC11332 | MGC11332 | -2.037 | 0.02682 |
| Hs.188495 | WD repeat domain 37 | WDR37 | -2.042 | 0.01138 |
| Hs.303023 | tubulin, beta 1 /// tubulin, beta 1 | TUBB1 | -2.043 | 0.02973 |
| Hs.550549 | Hypothetical protein FLJ35801 | FLJ35801 | -2.043 | 0.04798 |
| Hs.546467 | Epithelial stromal interaction 1 (breast) | EPSTI1 | -2.047 | 0.03127 |
| Hs.200825 | hypothetical protein LOC150005 | LOC150005 | -2.053 | 0.03857 |
| Hs.509414 | Kinectin 1 (kinesin receptor) | KTN1 | -2.053 | 0.00384 |
| Hs.12827 | CDNA clone IMAGE:5260726, partial cds | --- | -2.059 | 0.04558 |
| Hs.529948 | MDN1, midasin homolog (yeast) | MDN1 | -2.061 | 0.00891 |
| Hs.156457 | abhydrolase domain containing 9 | ABHD9 | -2.063 | 0.01111 |
| Hs.213044 | FLJ39237 protein | FLJ39237 | -2.071 | 0.02060 |
| Hs.342009 | Transcribed locus | --- | -2.075 | 0.00716 |
| Hs.85155 | Zinc finger protein 36, C3H type-like 1 | ZFP36L1 | -2.076 | 0.03942 |
| Hs.137183 | Down syndrome cell adhesion molecule like 1 | DSCAML1 | -2.076 | 0.00948 |
| Hs.470126 | Kynureninase (L-kynurenine hydrolase) | KYNU | -2.086 | 0.00310 |
| Hs.201554 | Chromosome 9 open reading frame 14 | C9orf14 | -2.089 | 0.04210 |
| Hs.87383 | keratin 24 | KRT24 | -2.090 | 0.00055 |
| Hs.148274 | holocarboxylase | HLCS | -2.091 | 0.00234 |
| Hs.434683 | Homo sapiens, clone IMAGE:5721950, mRNA | --- | -2.091 | 0.00337 |
| Hs.225943 | UDP-Gal:betaGlcNAc beta 1,3-galactosyltransferase, polypeptide 5 | B3GALT5 | -2.091 | 0.00553 |
| Hs.23491 | hypothetical protein LOC148824 | LOC148824 | -2.095 | 0.04477 |
| Hs.533948 | neuromedin B receptor | NMBR | -2.096 | 0.02268 |
| Hs.436495 | Ring finger and KH domain containing 1 | RKHD1 | -2.098 | 0.00116 |
| Hs.1074 | surfactant, pulmonary-associated protein C | SFTPC | -2.099 | 0.02624 |
| Hs.546391 | zinc finger protein 571 | ZNF571 | -2.100 | 0.02522 |
| Hs.35433 | CDC42 binding protein kinase alpha (DMPK-like) | CDC42BPA | -2.105 | 0.01763 |
| Hs.407557 | Homo sapiens, clone IMAGE:5267652, mRNA | --- | -2.107 | 0.02644 |
| Hs.177968 | similar to CG32056-PA | LOC440981 | -2.108 | 0.04746 |
| Hs.438587 | peptidylprolyl isomerase (cyclophilin)-like 2 | PPIL2 | -2.111 | 0.00413 |
| Hs.269059 | Jumonji, AT rich interactive domain 2 | JARID2 | -2.117 | 0.03757 |
| Hs.289828 | ankyrin repeat domain 16 | ANKRD16 | -2.118 | 0.00325 |
| Hs.231829 | glutamate decarboxylase 2 | GAD2 | -2.118 | 0.00426 |
| Hs.378760 | MRNA; cDNA DKFZp313I1020 | --- | -2.120 | 0.01495 |
| Hs.380740 | hypothetical protein MGC33309 | MGC33309 | -2.124 | 0.02790 |
| Hs.439145 | Transcribed locus, | --- | -2.125 | 0.02836 |
| Hs.98510 | WD repeat domain 44 | WDR44 | -2.128 | 0.00207 |
| Hs.534907 | hypothetical LOC440346 | LOC440346 | -2.131 | 0.04820 |
| Hs.48706 | Clone IMAGE:284736, mRNA sequence | --- | -2.136 | 0.01253 |
| Hs.449629 | odorant binding protein 2B /// 2A | OBP2B -A | -2.138 | 0.01848 |
| Hs.493585 | Myeloid/lymphoid or mixed-lineage leukemia | MLLT3 | -2.142 | 0.02464 |
| Hs.109620 | cysteine-rich secretory protein 1 | CRISP1 | -2.151 | 0.00794 |
| Hs.221074 | Transcribed locus | --- | -2.151 | 0.00607 |
| Hs.552191 | Full length insert cDNA clone YO02C07 | --- | -2.155 | 0.00060 |
| Hs.193170 | PDZ domain containing, X chromosome | FLJ21687 | -2.162 | 0.00547 |
| Hs.118769 | Transcribed locus | --- | -2.163 | 0.00223 |
| Hs.282871 | cytochrome P450, family 2, subfamily C, polypeptide 8 | CYP2C8 | -2.188 | 0.01819 |
| Hs.367437 | hypothetical protein MGC33948 | MGC33948 | -2.189 | 0.01425 |
| Hs.302037 | chromosome 6 open reading frame 12 | C6orf12 | -2.191 | 0.01563 |
| Hs.535413 | Rheumatoid arthritis synovium immunoglobulin heavy chain variable region | --- | -2.191 | 0.01644 |
| Hs.171001 | heparan sulfate 6-O-sulfotransferase 3 | HS6ST3 | -2.195 | 0.04198 |
| Hs.443625 | collagen, type III, alpha 1 | COL3A1 | -2.197 | 0.02760 |
| Hs.524134 | GATA binding protein 3 | GATA3 | -2.198 | 0.04202 |
| --- | cadherin 12, type 2 (N-cadherin 2) | CDH12 | -2.199 | 0.04086 |
| Hs.458309 | protein phosphatase 1, regulatory (inhibitor) subunit 3A | PPP1R3A | -2.208 | 0.03061 |
| Hs.131017 | Spermatogenesis associated 13 | SPATA13 | -2.209 | 0.04669 |
| Hs.535499 | retinoic acid receptor, alpha | RARA | -2.209 | 0.01750 |
| Hs.546271 | Poly(rC) binding protein 2 | PCBP2 | -2.212 | 0.03635 |
| Hs.444106 | torsin family 2, member A /// tetratricopeptide repeat domain 16 | TOR2A /// TTC16 | -2.213 | 0.00959 |
| Hs.370147 | centrosome spindle pole associated protein | CSPP | -2.216 | 0.01708 |
| Hs.528721 | semaphorin 3E | SEMA3E | -2.224 | 0.04338 |
| Hs.463041 | arginine-glutamic acid dipeptide (RE) repeats | RERE | -2.224 | 0.04877 |
| Hs.127657 | ventricular zone expressed PH domain homolog 1 | VEPH1 | -2.230 | 0.03700 |
| Hs.383791 | MRNA; cDNA DKFZp434N2419 | --- | -2.231 | 0.02064 |
| Hs.97876 | coiled-coil domain containing 8 | CCDC8 | -2.233 | 0.02247 |
| Hs.382314 | Homo sapiens, clone IMAGE:4750462, mRNA | --- | -2.238 | 0.01582 |
| Hs.127011 | tubulointerstitial nephritis antigen | TINAG | -2.242 | 0.01067 |
| Hs.376033 | hypothetical protein LOC285224 | LOC285224 | -2.245 | 0.01294 |
| Hs.518834 | UDP-Gal:betaGlcNAc beta 1,3-galactosyltransferase, polypeptide 2 | B3GALT2 | -2.247 | 0.01590 |
| Hs.410455 | unc-119 homolog (C. elegans) | UNC119 | -2.250 | 0.00570 |
| Hs.436040 | Cadherin 13, H-cadherin (heart) | CDH13 | -2.251 | 0.04054 |
| Hs.50216 | zinc finger protein 588 | ZNF588 | -2.255 | 0.01524 |
| Hs.434803 | Homo sapiens, clone IMAGE:5760997, mRNA | --- | -2.263 | 0.04995 |
| Hs.524750 | Nucleoporin 98kDa | NUP98 | -2.265 | 0.04629 |
| Hs.522261 | Homo sapiens, clone IMAGE:4838261, mRNA | --- | -2.270 | 0.00031 |
| Hs.73680 | cardiomyopathy associated 3 | CMYA3 | -2.270 | 0.02545 |
| Hs.147276 | chromosome 14 open reading frame 166B | C14orf166B | -2.278 | 0.03174 |
| Hs.210013 | CDNA FLJ25684 fis, clone TST04185 | --- | -2.279 | 0.01840 |
| Hs.241416 | MRNA; cDNA DKFZp434K1021 | --- | -2.282 | 0.00639 |
| Hs.124161 | hyperpolarization activated cyclic nucleotide-gated K+ channel 2 | HCN2 | -2.284 | 0.00423 |
| Hs.475472 | Hypothetical protein BC015088 | MGC16471 | -2.299 | 0.00089 |
| Hs.437 | transcription factor 15 (basic helix-loop-helix) | TCF15 | -2.326 | 0.03745 |
| Hs.522143 | zinc finger protein 205 | ZNF205 | -2.330 | 0.00603 |
| Hs.375831 | Homo sapiens, clone IMAGE:4819678, | --- | -2.337 | 0.04239 |
| Hs.24587 | embryonal Fyn-associated substrate | EFS | -2.343 | 0.01096 |
| Hs.148590 | cornifelin /// cornifelin | CNFN | -2.346 | 0.01690 |
| Hs.158748 | Solute carrier family 35, member F3 | SLC35F3 | -2.348 | 0.00222 |
| Hs.380334 | Zinc finger protein 148 (pHZ-52) | ZNF148 | -2.354 | 0.04854 |
| Hs.533953 | KIAA1462 | KIAA1462 | -2.363 | 0.02840 |
| Hs.306670 | CDNA FLJ14169 fis, clone NT2RP2002056 | --- | -2.371 | 0.04945 |
| Hs.105323 | Homo sapiens, clone IMAGE:5167446, mRNA | --- | -2.392 | 0.02520 |
| Hs.123004 | chromosome 9 open reading frame 36 | C9orf36 | -2.392 | 0.03115 |
| Hs.523221 | leucine zipper, putative tumor suppressor 2 | LZTS2 | -2.393 | 0.02238 |
| Hs.549177 | RUN and FYVE domain containing 2 | RUFY2 | -2.395 | 0.02974 |
| Hs.491232 | Solute carrier family 39 (zinc transporter), member 14 | SLC39A14 | -2.395 | 0.02986 |
| Hs.155942 | transient recep. potential cation channel, subfamily M, member 1 | TRPM1 | -2.400 | 0.02668 |
| Hs.44329 | hypothetical protein FLJ32214 | FLJ32214 | -2.425 | 0.00655 |
| Hs.487670 | hypothetical protein MGC42090 | MGC42090 | -2.427 | 0.00715 |
| Hs.512797 | hematopoietic SH2 domain containing | HSH2D | -2.430 | 0.02239 |
| Hs.386404 | Ubiquitination factor E4B (UFD2 homolog, yeast) | UBE4B | -2.436 | 0.02920 |
| Hs.514016 | hypothetical protein LOC339263 | LOC339263 | -2.443 | 0.02510 |
| Hs.534482 | Hypothetical protein MGC1203 | MGC1203 | -2.445 | 0.01193 |
| Hs.445000 | prostaglandin E receptor 3 (subtype EP3) | PTGER3 | -2.450 | 0.01316 |
| Hs.505104 | Kelch domain containing 5 | KIAA1340 | -2.459 | 0.00830 |
| Hs.303454 | SEC15-like 2 (S. cerevisiae) | SEC15L2 | -2.460 | 0.01388 |
| Hs.197320 | Transducin-like enhancer of split 1 (E(sp1) | TLE1 | -2.461 | 0.04362 |
| Hs.442527 | cytochrome P450, family 3, subfamily A, polypeptide 4 | CYP3A4 | -2.465 | 0.00026 |
| Hs.97391 | CDNA FLJ40497 fis, clone TESTI2044892 | --- | -2.469 | 0.00101 |
| Hs.120260 | Fc receptor-like 4 /// Fc receptor-like 4 | FCRL4 | -2.489 | 0.00613 |
| Hs.496456 | protocadherin 11 X-linked | PCDH11X | -2.490 | 0.00237 |
| Hs.335034 | Dihydropyrimidine dehydrogenase | DPYD | -2.507 | 0.01900 |
| Hs.391781 | Protocadherin 20 | PCDH20 | -2.510 | 0.01321 |
| Hs.254335 | casein kinase 1, gamma 1 | CSNK1G1 | -2.516 | 0.02777 |
| Hs.370852 | Homo sapiens, clone IMAGE:5273088, mRNA | --- | -2.520 | 0.02722 |
| Hs.491558 | ankyrin 1, erythrocytic /// ankyrin 1, erythrocytic | ANK1 | -2.536 | 0.00062 |
| Hs.507348 | Heparan sulfate (glucosamine) 3-O-sulfotransferase 1 | HS3ST1 | -2.537 | 0.00010 |
| Hs.406166 | hypothetical LOC400590 | LOC400590 | -2.545 | 0.02174 |
| Hs.480825 | Ring finger protein 150 | RNF150 | -2.550 | 0.00639 |
| Hs.313263 | CDNA FLJ37122 fis, clone BRACE2022448 | --- | -2.562 | 0.03885 |
| Hs.118727 | hairy and enhancer of split 2 (Drosophila) | HES2 | -2.565 | 0.03152 |
| Hs.186579 | cancer-associated nucleoprotein | CANP | -2.566 | 0.00665 |
| Hs.499705 | family with sequence similarity 13, member C1 | FAM13C1 | -2.571 | 0.00372 |
| Hs.437186 | polymerase (RNA) III (DNA directed) polypeptide K, 12.3 kDa | POLR3K | -2.584 | 0.01202 |
| --- | olfactory receptor, family 6, subfamily B, member 1 | OR6B1 | -2.585 | 0.02414 |
| Hs.545998 | Homo sapiens, clone IMAGE:3685819, mRNA | --- | -2.588 | 0.01893 |
| Hs.147062 | cyclic nucleotide gated channel beta 1 | CNGB1 | -2.594 | 0.01661 |
| Hs.524574 /// Hs.546795 | Nucleoporin 107kDa | NUP107 | -2.597 | 0.00188 |
| Hs.252433 | Hypothetical protein LOC339803 | LOC339803 | -2.615 | 0.00967 |
| Hs.84136 | paired-like homeodomain transcription factor 1 | PITX1 | -2.620 | 0.03265 |
| Hs.162595 | Similar to prostate cancer associated protein 5 isoform NGEP long; | --- | -2.622 | 0.03496 |
| Hs.178186 | amyloid beta (A4) precursor protein-binding, family B, member 1 | --- | -2.631 | 0.00719 |
| Hs.131297 | ST7 overlapping transcript 2 | ST7OT2 | -2.631 | 0.04272 |
| Hs.128075 | chromosome 9 open reading frame 128 | C9orf128 | -2.633 | 0.00665 |
| Hs.161220 | Hypothetical gene CG018 | CG018 | -2.643 | 0.02608 |
| Hs.100686 | breast cancer membrane protein 11 | BCMP11 | -2.643 | 0.01006 |
| Hs.130774 | F-box protein 10 | FBXO10 | -2.644 | 0.00065 |
| Hs.458306 | hypothetical protein FLJ40083 | FLJ40083 | -2.653 | 0.00454 |
| Hs.369763 | Hypothetical protein LOC92558 | LOC92558 | -2.668 | 0.00445 |
| Hs.2689 | protein kinase, cGMP-dependent, type I | PRKG1 | -2.676 | 0.03041 |
| Hs.218040 | integrin, beta | ITGB3 | -2.684 | 0.00306 |
| Hs.481936 | hypothetical protein FLJ13231 | FLJ13231 | -2.688 | 0.04133 |
| Hs.151220 | Palladin | KIAA0992 | -2.712 | 0.02704 |
| Hs.428214 | Mastermind-like 2 (Drosophila) | MAML2 | -2.722 | 0.00348 |
| Hs.446077 | solute carrier family 38, member 4 | SLC38A4 | -2.728 | 0.00745 |
| Hs.471834 | sushi, nidogen and EGF-like domains 1 | SNED1 | -2.728 | 0.03891 |
| Hs.159650 | hypothetical protein FLJ35834 | FLJ35834 | -2.733 | 0.02891 |
| Hs.89626 | parathyroid hormone-like hormone | PTHLH | -2.739 | 0.00973 |
| Hs.410810 | RAN binding protein 17 | RANBP17 | -2.748 | 0.04354 |
| Hs.522997 | unc-5 homolog B (C. elegans) | UNC5B | -2.752 | 0.00129 |
| Hs.216653 | F-box protein 9 | FBXO9 | -2.754 | 0.03989 |
| Hs.552590 | HpaII tiny fragments locus 9C | HTF9C | -2.759 | 0.03873 |
| Hs.163629 | hypothetical protein FLJ20130 | FLJ20130 | -2.791 | 0.01646 |
| Hs.445241 | CDNA FLJ37214 fis, clone BRALZ2008484 | --- | -2.792 | 0.00567 |
| Hs.479214 | CD38 antigen (p45) | CD38 | -2.796 | 0.03872 |
| Hs.66378 | HECT domain containing 2 | HECTD2 | -2.797 | 0.01460 |
| Hs.128823 | CDNA FLJ44654 fis, clone BRACE2047232 | --- | -2.819 | 0.03467 |
| Hs.21107 | neuroligin 4, X-linked | NLGN4X | -2.825 | 0.00191 |
| Hs.161254 | CDNA FLJ36285 fis, clone THYMU2003470 | --- | -2.838 | 0.04246 |
| Hs.489052 | Hypothetical protein BC004923 | LOC85865 | -2.851 | 0.00014 |
| Hs.233325 | hemochromatosis | HFE | -2.854 | 0.00278 |
| Hs.150749 | B-cell CLL/lymphoma 2 | BCL2 | -2.860 | 0.00665 |
| Hs.176227 | hypothetical protein FLJ11155 | FLJ11155 | -2.863 | 0.01449 |
| Hs.98244 | hypothetical protein MGC50721 | MGC50721 | -2.865 | 0.00496 |
| Hs.126655 | similar to RIKEN cDNA 4933437K13 | LOC92017 | -2.875 | 0.02919 |
| Hs.436657 | Clusterin | CLU | -2.889 | 0.02209 |
| Hs.85902 | calmodulin-like 6 | CALML6 | -2.893 | 0.00794 |
| Hs.480400 | B-cell scaffold protein with ankyrin repeats 1 | BANK1 | -2.901 | 0.00000 |
| Hs.409602 | sulfatase 1 | SULF1 | -2.909 | 0.00129 |
| Hs.106015 | Development and differentiation enhancing factor 1 | DDEF1 | -2.910 | 0.00304 |
| Hs.246590 | poly(A) binding protein, cytoplasmic 5 | PABPC5 | -2.921 | 0.04907 |
| Hs.449585 | Immunoglobulin lambda variable 3-21 | IGLC2 | -2.922 | 0.01446 |
| Hs.2799 | Hyaluronan and proteoglycan link protein 1 | HAPLN1 | -2.922 | 0.02205 |
| Hs.550832 | Homo sapiens, clone IMAGE:4824588, mRNA | --- | -2.961 | 0.01688 |
| Hs.549092 | suppression of tumorigenicity 18 (breast carcinoma) | ST18 | -2.970 | 0.01527 |
| Hs.552134 | CDNA clone IMAGE:5301169, partial cds | --- | -2.983 | 0.03523 |
| Hs.144567 | alanine-glyoxylate aminotransferase | AGXT | -2.998 | 0.00017 |
| Hs.14637 | N-acetyltransferase 8 (camello like) | NAT8 | -3.002 | 0.00173 |
| Hs.160215 | Transcribed locus | --- | -3.004 | 0.01385 |
| Hs.401062 | NOL1/NOP2/Sun domain family, member 3 | NSUN3 | -3.008 | 0.00059 |
| Hs.388715 | hypothetical protein LOC285733 | LOC285733 | -3.009 | 0.02216 |
| Hs.433586 | Similar to Serine/threonine protein phosphatase 5 (PP5) | --- | -3.032 | 0.00243 |
| Hs.534859 | Hypothetical gene BC012394; BC053611 | --- | -3.066 | 0.00610 |
| Hs.177193 | synaptotagmin IX | SYT9 | -3.082 | 0.00727 |
| Hs.195298 | sarcoglycan zeta | SGCZ | -3.114 | 0.00341 |
| Hs.439713 | Zinc finger protein 154 (pHZ-92) | ZNF154 | -3.127 | 0.04344 |
| Hs.407538 | Homo sapiens, clone IMAGE:5538960, mRNA | --- | -3.133 | 0.03194 |
| Hs.549092 | Suppression of tumorigenicity 18 (breast carcinoma) | ST18 | -3.199 | 0.00272 |
| Hs.322444 | Homo sapiens, clone IMAGE:3604069, mRNA | --- | -3.224 | 0.00613 |
| Hs.4290 | CDNA FLJ37366 fis, clone BRAMY2024416 | --- | -3.255 | 0.00912 |
| Hs.208544 | Potassium channel, subfamily K, member 1 | KCNK1 | -3.273 | 0.00093 |
| Hs.537383 | olfactory receptor, family 5, subfamily H, member 1 | OR5H1 | -3.290 | 0.00431 |
| Hs.549149 | catenin (cadherin-associated protein), alpha 3 | CTNNA3 | -3.323 | 0.03010 |
| --- | hypothetical protein DKFZp547J222 | DKFZp547J222 | -3.357 | 0.00147 |
| Hs.131152 | similar to SERTA domain containing 4 | LOC401778 | -3.372 | 0.02482 |
| Hs.408453 | Wilms tumor 1 | WT1 | -3.440 | 0.00425 |
| Hs.471162 | Ras association (RalGDS/AF-6) and pleckstrin homology domains 1 | RAPH1 | -3.563 | 0.00105 |
| Hs.274264 | visual system homeobox 1 homolog, CHX10-like | VSX1 | -3.633 | 0.00772 |
| Hs.27043 | K+ voltage-gated channel, subfamily H (eag-related), member 5 | KCNH5 | -3.687 | 0.00549 |
| Hs.387367 | cytochrome P450, family 39, subfamily A, polypeptide 1 | CYP39A1 | -3.693 | 0.01892 |
| Hs.147471 | similar to R30217_1 | FLJ16360 | -3.713 | 0.00114 |
| Hs.302921 | Full length insert cDNA clone YR17E12 | --- | -3.763 | 0.00217 |
| Hs.173536 | protein kinase D3 | PRKD3 | -3.882 | 0.00010 |
| Hs.440722 | zinc finger protein 417 | ZNF417 | -3.996 | 0.00115 |
| Hs.146040 | chromosome 14 open reading frame 105 | C14orf105 | -4.364 | 0.00018 |
