# Supplementary material for: Evaluation of the Genetic Response of U937 and Jurkat Cells to 10-Nanosecond Electrical Pulses (nsEP)
Source: PLoS One. 2016 May 2;11(5):e0154555. doi: 10.1371/journal.pone.0154555 (PMC4852903; doi:10.1371/journal.pone.0154555)
Supplement: S2 Table — Genes were selected based on log ratio (≥2, or ≤ -2) and with a p-value of ≤ 0.05. (DOCX) [file pone.0154555.s009.docx]

Supplementary Table 2: Complete list of significant genes changing in Jurkat cells exposed to 100 pulses of 10 ns at 150kV/cm applied field. Genes were selected based on log ratio (≥2, or ≤ -2) and with a p-value of ≤ 0.05.

| **UniGene ID** | **Gene name** | **Symbol** | **Fold change 150kVnsEP vs. SHAM** | **p-Value**  **150kv nsEP**  **vs. SHAM** |
| --- | --- | --- | --- | --- |
| Hs.25647 | v-fos FBJ murine osteosarcoma viral oncogene homolog | FOS | 7.269 | 0.00031 |
| Hs.326035 | Early growth response 1 | EGR1 | 5.213 | 0.00031 |
| Hs.326035 | early growth response 1 | EGR1 | 4.941 | 0.00029 |
| Hs.549031 | early growth response 4 | EGR4 | 4.472 | 0.00244 |
| Hs.326035 | early growth response 1 | EGR1 | 4.349 | 0.00009 |
| Hs.549777 | Full length insert cDNA clone YP97D11 | --- | 4.303 | 0.00368 |
| Hs.494326 | basic leucine zipper nuclear factor 1 (JEM-1) | BLZF1 | 4.106 | 0.00281 |
| Hs.1395 | early growth response 2 | EGR2 | 3.981 | 0.02375 |
| Hs.536535 | dual specificity phosphatase 16 | DUSP16 | 3.832 | 0.00036 |
| Hs.148819 | Syntrophin, gamma 2 | SNTG2 | 3.741 | 0.00596 |
| Hs.529512 | zinc finger protein 167 | ZNF167 | 3.700 | 0.01073 |
| Hs.525704 | v-jun sarcoma virus 17 oncogene homolog | JUN | 3.606 | 0.00257 |
| Hs.195398 | oligodendrocyte transcription factor 3 | OLIG3 | 3.604 | 0.01432 |
| Hs.413099 | glycine receptor, alpha 3 | GLRA3 | 3.579 | 0.03397 |
| Hs.75678 | FBJ murine osteosarcoma viral oncogene homolog B | FOSB | 3.577 | 0.00137 |
| Hs.549086 | discs, large (Drosophila) homolog-associated protein 1 | DLGAP1 | 3.523 | 0.00130 |
| Hs.532933 | purinergic receptor P2Y, G-protein coupled, 12 | P2RY12 | 3.499 | 0.00649 |
| Hs.525704 | v-jun sarcoma virus 17 oncogene homolog | JUN | 3.466 | 0.00004 |
| Hs.519601 | Inhibitor of DNA binding 4, dominant negative helix-loop-helix | ID4 | 3.400 | 0.00024 |
| Hs.56247 | inducible T-cell co-stimulator | ICOS | 3.390 | 0.00468 |
| Hs.162246 | proline-rich protein PRP2 | PRP2 | 3.265 | 0.01988 |
| Hs.498513 | Aldo-keto reductase family 1, member C2 | AKR1C2 | 3.232 | 0.00894 |
| Hs.483329 | KIAA1961 gene | KIAA1961 | 3.178 | 0.04366 |
| Hs.482730 | EGF-like repeats and discoidin I-like domains 3 | EDIL3 | 3.175 | 0.02533 |
| Hs.464983 | hypothetical gene supported by BC011527; | LOC284260 | 3.147 | 0.01901 |
| Hs.55047 | Full length insert cDNA clone ZB77E08 | --- | 3.136 | 0.00446 |
| Hs.552756 | hypothetical protein FLJ21463, | --- | 3.117 | 0.00052 |
| Hs.99503 | Homo sapiens, clone IMAGE:4823221, mRNA | --- | 3.097 | 0.00094 |
| Hs.306691 | GLIS family zinc finger 1 | GLIS1 | 3.075 | 0.01730 |
| Hs.208358 | chromosome 21 open reading frame 63 | C21orf63 | 3.055 | 0.00588 |
| Hs.120633 | sestrin 3 | SESN3 | 3.053 | 0.00009 |
| --- | similar to Argininosuccinate synthase ( | LOC402295 | 2.981 | 0.00621 |
| Hs.167451 | KIAA0999 protein | KIAA0999 | 2.953 | 0.02490 |
| Hs.363081 | hypothetical protein LOC255480 | LOC255480 | 2.944 | 0.00110 |
| Hs.508141 | diaphanous homolog 3 (Drosophila) | DIAPH3 | 2.920 | 0.01929 |
| Hs.212511 | hypothetical protein MGC15730 | MGC15730 | 2.909 | 0.00248 |
| Hs.104672 | downregulated in ovarian cancer 1 | DOC1 | 2.907 | 0.00346 |
| Hs.292156 | dickkopf homolog 3 (Xenopus laevis) | DKK3 | 2.894 | 0.01492 |
| Hs.129452 | dachshund homolog 1 (Drosophila) | DACH1 | 2.877 | 0.03520 |
| Hs.406779 | TPTE and PTEN homologous inositol lipid phosphatase pseudogene | LOC374491 | 2.873 | 0.00544 |
| Hs.433391 | metallothionein 1G | MT1G | 2.871 | 0.04324 |
| Hs.7200 | NIMA (never in mitosis gene a)- related kinase 9 | NEK9 | 2.854 | 0.00175 |
| Hs.375752 | Clone BGL1 mRNA sequence | --- | 2.848 | 0.04483 |
| Hs.413297 | regulator of G-protein signalling 16 | RGS16 | 2.842 | 0.00787 |
| Hs.279806 | DEAD (Asp-Glu-Ala-Asp) box polypeptide 5 | DDX5 | 2.840 | 0.04144 |
| Hs.98967 | ATPase, H+ transporting, lysosomal V0 subunit a isoform 4 | ATP6V0A4 | 2.839 | 0.00918 |
| Hs.25377 | ATP-binding cassette, sub-family A (ABC1), member 10 | ABCA10 | 2.835 | 0.03597 |
| Hs.121017 | histone 1, H2ae | HIST1H2AE | 2.810 | 0.00334 |
| Hs.46730 | FBI4 protein | FBI4 | 2.805 | 0.01309 |
| Hs.518814 | chemokine (C-X-C motif) ligand 11 | CXCL11 | 2.798 | 0.00208 |
| --- | TPTE and PTEN homologous inositol lipid phosphatase pseudogene | TPTEps1 | 2.779 | 0.03932 |
| Hs.482573 | WD repeat domain 41 | WDR41 | 2.766 | 0.02929 |
| Hs.376950 | Hypothetical gene supported by AL832797 | --- | 2.755 | 0.03512 |
| Hs.284707 | Homo sapiens, clone IMAGE:5259731, mRNA | --- | 2.735 | 0.00040 |
| Hs.389678 | Hypothetical protein FLJ35220 | FLJ35220 | 2.713 | 0.00639 |
| Hs.476358 | Calcium channel, voltage-dependent, L type, alpha 1D subunit | CACNA1D | 2.711 | 0.01647 |
| Hs.155017 | nuclear receptor interacting protein 1 | NRIP1 | 2.683 | 0.00184 |
| Hs.406847 | CDNA: FLJ20931 fis, clone ADSE01282 | --- | 2.681 | 0.00267 |
| Hs.43322 | protein kinase, AMP-activated, alpha 1 catalytic subunit | PRKAA1 | 2.658 | 0.00420 |
| Hs.479693 | Splicing factor, arginine/serine-rich 11 | SFRS11 | 2.647 | 0.00160 |
| Hs.8373 | FIS | FIS | 2.644 | 0.01211 |
| Hs.485640 | primase, polypeptide 2A, 58kDa | PRIM2A | 2.634 | 0.02468 |
| Hs.347270 | major histocompatibility complex, class II, DP alpha 1 | HLA-DPA1 | 2.631 | 0.01615 |
| Hs.542781 | Homo sapiens, clone IMAGE:4701591, mRNA | --- | 2.624 | 0.00252 |
| Hs.221436 | RNA binding motif, single stranded interacting protein | RBMS3 | 2.616 | 0.01997 |
| Hs.511605 | annexin A2 | ANXA2 | 2.613 | 0.01474 |
| Hs.30917 | cellular repressor of E1A-stimulated genes 2 | CREG2 | 2.610 | 0.01812 |
| Hs.544161 | Homo sapiens, clone IMAGE:5267205, mRNA | --- | 2.604 | 0.01305 |
| Hs.424980 | 5-hydroxytryptamine (serotonin) receptor 2A | HTR2A | 2.603 | 0.00275 |
| Hs.350741 | hypothetical protein FLJ32310 | FLJ32310 | 2.599 | 0.00269 |
| Hs.73793 | vascular endothelial growth factor | VEGF | 2.594 | 0.03963 |
| Hs.155090 | guanine nucleotide binding protein (G protein), beta 5 | GNB5 | 2.594 | 0.00722 |
| Hs.120633 | Sestrin 3 | SESN3 | 2.587 | 0.03342 |
| Hs.525704 | v-jun sarcoma virus 17 oncogene homolog (avian) | JUN | 2.586 | 0.00150 |
| Hs.369646 | mucin 4, tracheobronchial | MUC4 | 2.568 | 0.00595 |
| Hs.2700 | glycine receptor, alpha 2 | GLRA2 | 2.568 | 0.00501 |
| Hs.200929 | interleukin 23 receptor | IL23R | 2.567 | 0.00434 |
| Hs.129174 | IQ motif containing with AAA domain | IQCA | 2.559 | 0.04366 |
| Hs.285193 | Full length insert cDNA clone YA80A03 | --- | 2.557 | 0.00020 |
| Hs.205865 | Hypothetical protein LOC143458 | LOC143458 | 2.553 | 0.00168 |
| Hs.523702 | membrane-spanning 4-domains, subfamily A, member 6A | MS4A6A | 2.549 | 0.00139 |
| Hs.461389 | contactin associated protein-like 4 | CNTNAP4 | 2.541 | 0.03503 |
| Hs.495656 | transducin (beta)-like 1X-linked | TBL1X | 2.534 | 0.02643 |
| --- | olfactory receptor, family 2, subfamily W, member 1 | OR2W1 | 2.522 | 0.04581 |
| Hs.367639 | hypothetical protein FLJ20032 | FLJ20032 | 2.508 | 0.00172 |
| Hs.528391 | similar to zinc finger protein 596 | LOC283202 | 2.503 | 0.02934 |
| Hs.499674 | mannose-binding lectin (protein C) 2, soluble | MBL2 | 2.499 | 0.04049 |
| Hs.445265 | LIM homeobox 2 | LHX2 | 2.498 | 0.00006 |
| Hs.371240 | A kinase (PRKA) anchor protein (gravin) 12 | AKAP12 | 2.490 | 0.00762 |
| Hs.211763 | MRNA; cDNA DKFZp434D1028 | --- | 2.485 | 0.03705 |
| Hs.61389 | chromosome 6 open reading frame 52 | C6orf52 | 2.482 | 0.02690 |
| Hs.514846 | protein inhibitor of activated STAT, 2 | PIAS2 | 2.475 | 0.02435 |
| Hs.370661 | phosphodiesterase 5A, cGMP-specific | PDE5A | 2.458 | 0.04610 |
| Hs.374147 | Hypothetical protein FLJ23235 | FLJ23235 | 2.448 | 0.00440 |
| Hs.535216 | similar to hypothetical protein FLJ35782 | LOC442444 | 2.425 | 0.00105 |
| Hs.454600 | DKFZP434L187 protein | DKFZP434L187 | 2.419 | 0.03645 |
| Hs.11355 | thymopoietin | TMPO | 2.417 | 0.03869 |
| Hs.227049 | CTP synthase II | CTPS2 | 2.405 | 0.02625 |
| Hs.527697 | Homo sapiens, clone IMAGE:3604678, mRNA | --- | 2.403 | 0.00049 |
| Hs.468688 | chromosome 10 open reading frame 137 | C10orf137 | 2.399 | 0.00660 |
| Hs.514116 | zinc finger protein 403 | ZNF403 | 2.395 | 0.00961 |
| Hs.385793 | Homo sapiens, clone IMAGE:5242593, mRNA | --- | 2.394 | 0.03335 |
| Hs.495912 | dystrophin | DMD | 2.390 | 0.02869 |
| Hs.384648 | Full length insert cDNA clone YT85D04 | --- | 2.387 | 0.02087 |
| Hs.379858 | Dimethylarginine dimethylaminohydrolase 1 | DDAH1 | 2.385 | 0.00702 |
| Hs.370108 | chromosome 21 open reading frame 84 | C21orf84 | 2.375 | 0.02381 |
| Hs.522561 | gap junction protein, beta 3, 31kDa (connexin 31) | GJB3 | 2.364 | 0.00069 |
| Hs.468908 | MAX dimerization protein 1 | MAD | 2.354 | 0.00951 |
| Hs.241431 | G-protein, alpha activating activity polypeptide O | GNAO1 | 2.331 | 0.04782 |
| Hs.520319 | solute carrier family 22 member 16 | SLC22A16 | 2.327 | 0.01343 |
| Hs.146604 | Chromosome 21 open reading frame 9 | C21orf9 | 2.322 | 0.00392 |
| Hs.250072 | solute carrier family 4, Na bicarbonate cotransporter, member 7 | SLC4A7 | 2.320 | 0.00332 |
| Hs.532901 | hypothetical protein FLJ32954 | FLJ32954 | 2.311 | 0.00423 |
| Hs.455089 | KIAA1729 protein | KIAA1729 | 2.310 | 0.01143 |
| Hs.460 | activating transcription factor 3 | ATF3 | 2.301 | 0.00052 |
| Hs.37482 | coatomer protein complex, subunit zeta 2 | COPZ2 | 2.298 | 0.02898 |
| Hs.76556 | protein phosphatase 1, regulatory (inhibitor) subunit 15A | PPP1R15A | 2.298 | 0.00315 |
| Hs.484918 | Cytidine monophosphate-N-acetylneuraminic acid hydroxylase | CMAH | 2.294 | 0.00078 |
| Hs.127657 | ventricular zone expressed PH domain homolog 1 | VEPH1 | 2.293 | 0.03467 |
| Hs.414110 | K+ voltage-gated channel, Shal-related subfamily, member 3 | KCND3 | 2.291 | 0.02239 |
| Hs.350581 | SH2 domain-containing molecule EAT2 | EAT2 | 2.291 | 0.04548 |
| Hs.277677 | NADH dehydrogenase (ubiquinone) 1 alpha subcomplex, 10, 42kDa | NDUFA10 | 2.286 | 0.00182 |
| Hs.485352 | EPS8-like 3 | EPS8L3 | 2.277 | 0.01133 |
| Hs.520406 | Non-coding transcript, polyA signal, clone 78-E/T5 | --- | 2.269 | 0.01507 |
| Hs.534313 | early growth response 3 | EGR3 | 2.265 | 0.02163 |
| Hs.189826 | zinc finger protein 236 | ZNF236 | 2.263 | 0.02015 |
| Hs.153952 | 5'-nucleotidase, ecto (CD73) | NT5E | 2.258 | 0.03407 |
| Hs.376206 | Kruppel-like factor 4 (gut) | KLF4 | 2.257 | 0.01711 |
| Hs.102471 | Phosphatase and actin regulator 2 | PHACTR2 | 2.255 | 0.04996 |
| Hs.145251 | mirror-image polydactyly 1 | MIPOL1 | 2.252 | 0.02111 |
| Hs.550470 | ceruloplasmin (ferroxidase) | CP | 2.244 | 0.00341 |
| Hs.171825 | basic helix-loop-helix domain containing, class B, 2 | BHLHB2 | 2.235 | 0.03379 |
| Hs.434537 | hypothetical gene supported by BC040853 | LOC440028 | 2.225 | 0.01474 |
| Hs.432364 | RAD26L hypothetical protein | LOC375748 | 2.222 | 0.02484 |
| Hs.440544 | Chloride intracellular channel 4 | CLIC4 | 2.222 | 0.00869 |
| Hs.374067 | Ubiquitin protein ligase E3B | UBE3B | 2.220 | 0.01668 |
| Hs.109655 | Sex comb on midleg-like 1 (Drosophila) | SCML1 | 2.218 | 0.00897 |
| Hs.213289 | low density lipoprotein receptor | LDLR | 2.218 | 0.00957 |
| Hs.120759 | apolipoprotein B (including Ag(x) antigen) | APOB | 2.208 | 0.03929 |
| Hs.462475 | TL132 protein | LOC220594 | 2.207 | 0.00067 |
| Hs.132121 | Hypothetical protein DKFZp761D221 | DKFZp761D221 | 2.204 | 0.01865 |
| Hs.535746 | Similar to ribosomal protein L26 | --- | 2.193 | 0.00679 |
| Hs.150556 | Hypothetical protein FLJ43663 | FLJ43663 | 2.193 | 0.00547 |
| Hs.510172 | Human immunodeficiency virus type I enhancer binding protein 2 | HIVEP2 | 2.188 | 0.04531 |
| Hs.272328 | zinc finger protein 354C | ZNF354C | 2.185 | 0.02719 |
| --- | olfactory receptor, family 1, subfamily D, member 4 & 5 | OR1D4-5 | 2.181 | 0.04667 |
| Hs.524278 | guanylate cyclase 2C | GUCY2C | 2.178 | 0.03395 |
| Hs.500812 | beta-transducin repeat containing | BTRC | 2.177 | 0.04912 |
| Hs.99231 | G protein-coupled receptor 74 | GPR74 | 2.171 | 0.00334 |
| Hs.180758 | Chondroitin sulfate GalNAcT-2 | GALNACT-2 | 2.159 | 0.04699 |
| Hs.517453 | immunoglobulin lambda joining 3 | IGLJ3 | 2.146 | 0.03317 |
| Hs.411402 | Homo sapiens, clone IMAGE:4469683, mRNA | --- | 2.145 | 0.03269 |
| Hs.117545 | phosphodiesterase 4D, cAMP-specific | PDE4D | 2.141 | 0.03516 |
| Hs.434948 | TGF-betaIIR beta | --- | 2.140 | 0.03957 |
| Hs.302016 | prostate and breast cancer overexpressed 1 | PBOV1 | 2.134 | 0.03489 |
| Hs.49573 | hypothetical gene supported by AK126569 | FLJ44606 | 2.133 | 0.03421 |
| Hs.208854 | CD69 antigen | CD69 | 2.132 | 0.00697 |
| Hs.497817 | Homo sapiens, clone IMAGE:5208312, mRNA | --- | 2.122 | 0.00937 |
| Hs.148907 | Solute carrier family 5 (sodium/glucose cotransporter), member 12 | SLC5A12 | 2.121 | 0.04663 |
| Hs.349150 | Purine-rich element binding protein B | PURB | 2.120 | 0.01198 |
| Hs.212838 | alpha-2-macroglobulin | A2M | 2.118 | 0.02127 |
| Hs.544373 | Homo sapiens, clone IMAGE:4838152, mRNA | --- | 2.114 | 0.03279 |
| Hs.551985 | Clone IMAGE:501887, mRNA sequence | --- | 2.109 | 0.00529 |
| Hs.513541 | similar to hect domain and RLD 2 | LOC440366 | 2.107 | 0.00471 |
| Hs.521295 | LR8 protein | LR8 | 2.103 | 0.02373 |
| Hs.479898 | sulfotransferase family 1E, estrogen-preferring, member 1 | SULT1E1 | 2.101 | 0.00252 |
| Hs.406976 | hypothetical protein LOC283874 | LOC283874 | 2.099 | 0.02201 |
| Hs.348434 | Homo sapiens, clone IMAGE:5286779, mRNA | --- | 2.093 | 0.03405 |
| Hs.121443 | Homeodomain-only protein | HOP | 2.086 | 0.00299 |
| Hs.160562 | insulin-like growth factor 1 (somatomedin C) | IGF1 | 2.083 | 0.00489 |
| Hs.175905 | AU RNA binding protein/enoyl-Coenzyme A hydratase | AUH | 2.083 | 0.02566 |
| Hs.471200 | Neuropilin 2 | NRP2 | 2.082 | 0.02952 |
| Hs.193133 | SAM and SH3 domain containing 1 | SASH1 | 2.081 | 0.00004 |
| Hs.445932 | Transcribed locus | --- | 2.079 | 0.02072 |
| Hs.510324 | Quaking homolog, KH domain RNA binding | QKI | 2.077 | 0.02401 |
| Hs.137154 | Phosphatidylinositol glycan, class A | PIGA | 2.070 | 0.00957 |
| Hs.389103 | glucagon-like peptide 1 receptor | GLP1R | 2.061 | 0.02818 |
| Hs.334603 | RALBP1 associated Eps domain containing 1 | REPS1 | 2.059 | 0.02257 |
| Hs.283011 | a disintegrin and metalloproteinase domain 30 | ADAM30 | 2.056 | 0.01432 |
| Hs.549355 | similar to echinoderm microtubule associated protein like 5 | LOC400954 | 2.053 | 0.02641 |
| Hs.547420 | Homo sapiens, clone IMAGE:5267175, mRNA | --- | 2.039 | 0.01971 |
| Hs.549084 | LATS, large tumor suppressor, homolog 1 | LATS1 | 2.036 | 0.01900 |
| Hs.495000 | T-cell antigen receptor alpha (TCRA) | --- | 2.035 | 0.00585 |
| Hs.434752 | Homo sapiens, clone IMAGE:5164889, mRNA | --- | 2.033 | 0.04900 |
| Hs.524828 | Zinc finger protein ZFOC1 | ZFOC1 | 2.027 | 0.04171 |
| Hs.306673 | hypothetical protein LOC286059 | LOC286059 | 2.027 | 0.02078 |
| Hs.444225 | estrogen-related receptor gamma | ESRRG | 2.026 | 0.02873 |
| Hs.129895 | T-box 3 (ulnar mammary syndrome) | TBX3 | 2.024 | 0.04201 |
| Hs.12440 | Clone 24734 mRNA sequence | --- | 2.022 | 0.00098 |
| Hs.405144 | Splicing factor, arginine/serine-rich 3 | SFRS3 | 2.020 | 0.03332 |
| Hs.182137 | histone 1, H2bg | HIST1H2BG | 2.020 | 0.02046 |
| Hs.78944 | regulator of G-protein signalling 2, 24kDa | RGS2 | 2.017 | 0.00850 |
| Hs.435413 | Metastasis associated 1 family, member 3 | MTA3 | 2.013 | 0.01089 |
| Hs.483873 | GM2 ganglioside activator | GM2A | 2.010 | 0.03344 |
| Hs.58679 | solute carrier family 7, member 10 | SLC7A10 | 2.010 | 0.01589 |
| Hs.543067 | MRNA; cDNA DKFZp761H2217 | --- | 2.010 | 0.00040 |
| Hs.552747 | Homo sapiens, clone IMAGE:4822128, mRNA | --- | 2.007 | 0.04588 |
| Hs.279245 | transforming, acidic coiled-coil containing protein 1 | TACC1 | 2.003 | 0.01910 |
| Hs.348365 | CARD only protein | COPl | 2.000 | 0.01001 |
|  |  |  |  |  |
| Hs.97461 | hypothetical protein LOC157931 | LOC157931 | -2.002 | 0.03796 |
| Hs.528024 | Microtubule associated monoxygenase, calponin LIM domain | MICAL3 | -2.005 | 0.00437 |
| Hs.75535 | myosin, light polypeptide 2, regulatory, cardiac, slow | MYL2 | -2.007 | 0.00013 |
| Hs.524161 | Ras suppressor protein 1 | RSU1 | -2.010 | 0.00152 |
| Hs.150556 | Hypothetical protein FLJ43663 | FLJ43663 | -2.013 | 0.04885 |
| Hs.515479 | Hypothetical BC282485_1 | LOC388554 | -2.015 | 0.00429 |
| Hs.445692 | CDNA FLJ41867 fis, clone OCBBF2005546 | --- | -2.017 | 0.04163 |
| Hs.34871 | Zinc finger homeobox 1b | ZFHX1B | -2.019 | 0.00231 |
| Hs.509447 | Glucocorticoid receptor DNA binding factor 1 | GRLF1 | -2.019 | 0.01070 |
| Hs.173716 | a disintegrin and metalloproteinase domain 33 | ADAM33 | -2.022 | 0.03416 |
| Hs.149414 | complement component (3b/4b) receptor 1-like | CR1L | -2.026 | 0.04419 |
| Hs.495880 | Polymerase (DNA directed), alpha | POLA | -2.030 | 0.03218 |
| Hs.490765 | K+ intermediate/small conductance Ca2+-activated channel, | KCNN3 | -2.033 | 0.00045 |
| Hs.370024 | SEC31-like 1 (S. cerevisiae) | SEC31L1 | -2.035 | 0.03846 |
| Hs.343244 | Adaptor-related protein complex 1, gamma 2 subunit | AP1G2 | -2.042 | 0.00333 |
| Hs.42151 | histamine N-methyltransferase | HNMT | -2.043 | 0.03514 |
| Hs.434207 | Histidyl-tRNA synthetase 2 | HARS2 | -2.047 | 0.00533 |
| Hs.444414 | lymphoid nuclear protein related to AF4 | LAF4 | -2.055 | 0.01504 |
| Hs.428027 | Pre-B-cell leukemia transcription factor 3 | PBX3 | -2.059 | 0.04635 |
| Hs.404102 | ATP-binding cassette, sub-family B (MDR/TAP), member 5 | ABCB5 | -2.059 | 0.00645 |
| Hs.483454 | Calponin 3, acidic | CNN3 | -2.063 | 0.01155 |
| Hs.430646 | cyclin C | CCNC | -2.065 | 0.03008 |
| Hs.126932 | Transcribed locus | --- | -2.071 | 0.01853 |
| Hs.542046 | hypothetical protein FLJ20378, | --- | -2.073 | 0.01266 |
| Hs.495674 | Chloride channel 4 | CLCN4 | -2.076 | 0.01105 |
| Hs.41735 | Purinergic receptor P2X, ligand-gated ion channel, 1 | P2RX1 | -2.082 | 0.03116 |
| Hs.132121 | hypothetical protein DKFZp761D221 | DKFZp761D221 | -2.082 | 0.02559 |
| Hs.525287 | Rho GTPase activating protein 5 | ARHGAP5 | -2.087 | 0.01892 |
| Hs.515056 | guanine nucleotide binding protein (G protein), alpha 11 (Gq class) | GNA11 | -2.088 | 0.00877 |
| Hs.523899 | potassium voltage-gated channel, Isk-related family, member 3 | KCNE3 | -2.092 | 0.03565 |
| Hs.208353 | hypothetical protein LOC55565 | LOC55565 | -2.093 | 0.04995 |
| Hs.531085 | T-box 2 | TBX2 | -2.096 | 0.02179 |
| Hs.470457 | COBL-like 1 | COBLL1 | -2.097 | 0.00943 |
| Hs.485489 | Chloride intracellular channel 5 | CLIC5 | -2.100 | 0.04623 |
| Hs.525017 | hypothetical protein FLJ11848 | FLJ11848 | -2.102 | 0.03494 |
| Hs.547072 | Homo sapiens, clone IMAGE:5296524, mRNA | --- | -2.105 | 0.02593 |
| Hs.463041 | arginine-glutamic acid dipeptide (RE) repeats | RERE | -2.105 | 0.03735 |
| Hs.434255 | pleckstrin and Sec7 domain containing 3 | PSD3 | -2.108 | 0.04724 |
| Hs.516651 | hypothetical protein FLJ25415 | FLJ25415 | -2.112 | 0.02090 |
| Hs.282113 | SNF1-like kinase | SNF1LK | -2.117 | 0.00085 |
| Hs.402352 | Hypothetical LOC283583 | --- | -2.119 | 0.03066 |
| Hs.223782 | extraembryonic, spermatogenesis, homeobox 1-like | ESX1L | -2.123 | 0.04355 |
| Hs.546880 | hypothetical protein LOC283901 | LOC283901 | -2.126 | 0.02069 |
| Hs.210013 | CDNA FLJ25684 fis, clone TST04185 | --- | -2.134 | 0.03089 |
| Hs.147851 | mast cell immunoreceptor signal transducer | MIST | -2.139 | 0.03569 |
| Hs.196952 | Hepatic leukemia factor | HLF | -2.149 | 0.00102 |
| Hs.24418 | Transcribed locus | --- | -2.155 | 0.01536 |
| Hs.408241 | Nucleoporin like 2 | NUPL2 | -2.157 | 0.03920 |
| Hs.527524 | KIAA1280 protein | KIAA1280 | -2.157 | 0.02842 |
| Hs.123024 | gamma-aminobutyric acid (GABA) A receptor, alpha 3 | GABRA3 | -2.161 | 0.04124 |
| Hs.377010 | Carbamoyl-phosphate synthetase 2, aspartate transcarbamylase, | CAD | -2.163 | 0.00012 |
| Hs.250493 | Zinc finger protein 219 | ZNF219 | -2.163 | 0.02378 |
| Hs.535734 | KIAA1430 | KIAA1430 | -2.174 | 0.04448 |
| Hs.117183 | Homo sapiens, clone IMAGE:4183247, mRNA | --- | -2.175 | 0.01715 |
| Hs.403828 | FLJ43654 protein | FLJ43654 | -2.177 | 0.01973 |
| Hs.275711 | peroxisome proliferative activated receptor, alpha | PPARA | -2.177 | 0.00566 |
| Hs.499209 | Supervillin | SVIL | -2.178 | 0.00733 |
| Hs.444049 | B-cell linker | BLNK | -2.190 | 0.01385 |
| Hs.386684 | Abelson helper integration site | AHI1 | -2.204 | 0.03915 |
| Hs.21691 | G protein-coupled receptor 75 | GPR75 | -2.204 | 0.02265 |
| Hs.430335 | Transcribed locus | --- | -2.230 | 0.02104 |
| Hs.376609 | CDNA FLJ34736 fis, clone MESAN2008222 | --- | -2.237 | 0.00688 |
| Hs.22393 | Density-regulated protein | DENR | -2.238 | 0.03522 |
| Hs.511093 | Nucleolar and spindle associated protein 1 | NUSAP1 | -2.244 | 0.04898 |
| Hs.82921 | chromosome 6 open reading frame 165 | C6orf165 | -2.244 | 0.00672 |
| Hs.400802 | Zinc finger and BTB domain containing 1 | ZBTB1 | -2.252 | 0.01085 |
| Hs.19987 | Hypothetical protein FLJ14213 | FLJ14213 | -2.258 | 0.04292 |
| Hs.252707 | hypothetical LOC284067 | LOC284067 | -2.260 | 0.00768 |
| Hs.531547 | KIAA0565 gene product | KIAA0565 | -2.264 | 0.04338 |
| Hs.112783 | Androgen-induced 1 | AIG1 | -2.266 | 0.03852 |
| Hs.405607 | phosphatase, orphan 1 | PHOSPHO1 | -2.269 | 0.00300 |
| --- | hypothetical protein PRO2964 | PRO2964 | -2.272 | 0.00057 |
| Hs.301431 | zinc finger protein 71 (Cos26) | ZNF71 | -2.276 | 0.00072 |
| Hs.250281 | Solute carrier family 13, member 3 | SLC13A3 | -2.279 | 0.04900 |
| Hs.382689 | Hypothetical gene supported by BC029593 | --- | -2.282 | 0.00511 |
| Hs.526594 | KIAA0657 protein | KIAA0657 | -2.284 | 0.02462 |
| Hs.106511 | Hypothetical protein LOC144997 | PCDH17 | -2.291 | 0.00462 |
| Hs.177959 | a disintegrin and metalloproteinase domain 2 | ADAM2 | -2.304 | 0.02224 |
| Hs.101480 | AP1 gamma subunit binding protein 1 | AP1GBP1 | -2.306 | 0.00831 |
| Hs.20107 | Kinesin 2 60/70kDa | KNS2 | -2.308 | 0.00080 |
| Hs.370312 | Farnesyltransferase, CAAX box, alpha | FNTA | -2.311 | 0.01523 |
| Hs.397100 | Similar to ATP synthase, H+ transporting, , | --- | -2.325 | 0.00495 |
| Hs.437365 | N-acylsphingosine amidohydrolase (acid ceramidase)-like | ASAHL | -2.326 | 0.01366 |
| Hs.5148 | FLN29 gene product | FLN29 | -2.327 | 0.00213 |
| Hs.500750 | heparanase 2 | HPSE2 | -2.333 | 0.00580 |
| Hs.525264 | complement component 1, r subcomponent-like | C1RL | -2.334 | 0.02915 |
| Hs.530075 | Transcribed locus | --- | -2.341 | 0.04817 |
| Hs.50802 | hypothetical protein FLJ14712 | FLJ14712 | -2.343 | 0.01496 |
| Hs.534937 | Similar to RIKEN cDNA 2310076L09 | --- | -2.344 | 0.00031 |
| Hs.66194 | CDNA clone IMAGE:4611512, partial cds | --- | -2.359 | 0.04120 |
| Hs.532326 | carbonic anhydrase VB-like | CA5BL | -2.377 | 0.00485 |
| Hs.209374 | Hypothetical protein LOC339529 | LOC339529 | -2.389 | 0.02526 |
| Hs.112621 | glutamate receptor, metabotropic 3 | GRM3 | -2.401 | 0.02413 |
| Hs.172928 | collagen, type I, alpha 1 | COL1A1 | -2.410 | 0.03231 |
| Hs.513313 | LOC124402 | LOC124402 | -2.414 | 0.00344 |
| Hs.171001 | Heparan sulfate 6-O-sulfotransferase 3 | HS6ST3 | -2.415 | 0.03364 |
| Hs.490981 | Methionine sulfoxide reductase A | MSRA | -2.427 | 0.01435 |
| Hs.128576 | CDNA clone IMAGE:3029742, partial cds | --- | -2.431 | 0.01645 |
| Hs.334910 | Similar to guanidinoacetate methyltransferase; GAMT | --- | -2.435 | 0.03200 |
| Hs.248201 | Noggin | NOG | -2.441 | 0.01621 |
| Hs.469615 | septin 10 | 9/10/2005 | -2.446 | 0.02642 |
| Hs.105621 | LOC441301 | --- | -2.448 | 0.04470 |
| Hs.548149 | hypothetical protein LOC285878 | LOC285878 | -2.468 | 0.01356 |
| Hs.170904 | Growth hormone regulated TBC protein 1 | GRTP1 | -2.481 | 0.01900 |
| Hs.62022 | laminin, beta 4 | LAMB4 | -2.481 | 0.00535 |
| Hs.61812 | Protein tyrosine phosphatase, non-receptor type 12 | PTPN12 | -2.484 | 0.04959 |
| Hs.152414 | homeo box D13 | HOXD13 | -2.490 | 0.00452 |
| Hs.477866 | procollagen-lysine, 2-oxoglutarate 5-dioxygenase 2 | PLOD2 | -2.493 | 0.03971 |
| Hs.213307 | hypothetical protein LOC285429 | LOC285429 | -2.512 | 0.01746 |
| Hs.478000 | Muscleblind-like (Drosophila) | MBNL1 | -2.516 | 0.03879 |
| Hs.316856 | Homo sapiens, clone IMAGE:4818734, mRNA | --- | -2.517 | 0.01749 |
| Hs.510191 | potassium channel, subfamily K, member 13 | KCNK13 | -2.522 | 0.04068 |
| Hs.408385 | progestin and adipoQ receptor family member IX | PAQR9 | -2.522 | 0.01255 |
| Hs.368851 | zinc finger, DHHC domain containing 11 | ZDHHC11 | -2.528 | 0.02193 |
| Hs.131887 | DnaJ homology subfamily A member 5 | LOC134218 | -2.537 | 0.02712 |
| Hs.150122 | oxysterol binding protein-like 10 | OSBPL10 | -2.542 | 0.00484 |
| Hs.129051 | Homer homolog 1 (Drosophila) | HOMER1 | -2.547 | 0.03931 |
| Hs.472054 | chromosome 20 open reading frame 42 | C20orf42 | -2.548 | 0.01233 |
| Hs.249591 | Chromosome 9 open reading frame 55 | C9orf55 | -2.563 | 0.02819 |
| Hs.550294 | CDNA: FLJ20870 fis, clone ADKA02524 | --- | -2.565 | 0.04913 |
| Hs.334831 | Fibroblast growth factor receptor substrate 2 | FRS2 | -2.571 | 0.01460 |
| Hs.528131 | chromosome 14 open reading frame 102 | C14orf102 | -2.572 | 0.01196 |
| Hs.407926 | Likely ortholog of mouse TORC2-specific protein AVO3 | AVO3 | -2.589 | 0.02431 |
| Hs.450694 | Hephaestin-like 1 | HEPHL1 | -2.595 | 0.02588 |
| Hs.552486 | Homo sapiens, clone IMAGE:4815589, mRNA | --- | -2.599 | 0.04338 |
| Hs.121899 | Transcribed locus | --- | -2.613 | 0.02044 |
| Hs.82614 | glycogen synthase 2 (liver) | GYS2 | -2.624 | 0.01066 |
| Hs.522621 | MRNA; cDNA DKFZp686H0940 | --- | -2.634 | 0.00501 |
| Hs.166271 | Transcribed locus | --- | -2.653 | 0.00308 |
| Hs.462833 | hypothetical protein FLJ31952 | FLJ31952 | -2.663 | 0.01740 |
| Hs.503787 | Aspartyl-tRNA synthetase | DARS | -2.667 | 0.00418 |
| Hs.133421 | Leukemia inhibitory factor receptor | LIFR | -2.683 | 0.00108 |
| Hs.475364 | SLIT-ROBO Rho GTPase activating protein 3 | SRGAP3 | -2.687 | 0.03678 |
| Hs.434951 | Ubiquitin specific protease 15 | USP15 | -2.691 | 0.01373 |
| Hs.462341 | Myosin phosphatase-Rho interacting protein | M-RIP | -2.692 | 0.00459 |
| Hs.145444 | CDNA FLJ11494 fis, clone HEMBA1001942 | --- | -2.693 | 0.00830 |
| Hs.378501 | tRNA splicing endonuclease 54 homolog | TSEN54 | -2.707 | 0.00577 |
| Hs.435535 | Zinc finger protein 395 | ZNF395 | -2.711 | 0.03103 |
| Hs.546322 | Nucleolar protein 4 | NOL4 | -2.719 | 0.01869 |
| Hs.114198 | Mitogen-activated protein kinase kinase 5 | MAP2K5 | -2.722 | 0.01825 |
| Hs.148675 | hypothetical protein LOC283484 | LOC283484 | -2.732 | 0.00383 |
| Hs.296679 | Similar to beta-1,4-mannosyltransferase; | --- | -2.744 | 0.01958 |
| Hs.9873 | homolog of rat kinase D-interacting substance | KIDINS220 | -2.769 | 0.04046 |
| Hs.98594 | Rho guanine nucleotide exchange factor (GEF) 10 | ARHGEF10 | -2.791 | 0.03823 |
| Hs.302341 | ST8 alpha-N-acetyl-neuraminide alpha-2,8-sialyltransferase 2 | ST8SIA2 | -2.792 | 0.01737 |
| Hs.297397 | hypothetical protein FLJ23865 | FLJ23865 | -2.808 | 0.01020 |
| Hs.54397 | cholinergic receptor, nicotinic, beta polypeptide 4 | CHRNB4 | -2.841 | 0.00681 |
| Hs.429434 | GRB2-associated binding protein 2 | GAB2 | -2.842 | 0.00610 |
| Hs.137569 | tumor protein p73-like | TP73L | -2.851 | 0.00094 |
| Hs.508757 | Hypothetical protein LOC283501 | LOC283501 | -2.872 | 0.00223 |
| Hs.2868 | peripheral myelin protein 2 | PMP2 | -2.873 | 0.01360 |
| Hs.434694 | Homo sapiens, clone IMAGE:5197767, mRNA | --- | -2.879 | 0.00193 |
| Hs.268606 | Carbohydrate kinase-like | CARKL | -2.883 | 0.00899 |
| Hs.371346 | hypothetical protein LOC157381 | LOC157381 | -2.904 | 0.01447 |
| Hs.377972 | Chromosome 13 open reading frame 21 | C13orf21 | -2.928 | 0.04599 |
| Hs.105738 | Transcribed locus | --- | -2.937 | 0.00499 |
| Hs.147694 | hypothetical protein FLJ37673 | FLJ37673 | -3.007 | 0.02241 |
| Hs.369263 | Androgen-induced proliferation inhibitor | APRIN | -3.059 | 0.03108 |
| Hs.530150 | CDNA FLJ34038 fis, clone FCBBF2005645 | --- | -3.082 | 0.00868 |
| Hs.382174 | CDNA clone IMAGE:5259382, partial cds | --- | -3.086 | 0.01254 |
| Hs.24115 | chromosome 13 open reading frame 25 | C13orf25 | -3.098 | 0.04827 |
| Hs.495410 | DEAD (Asp-Glu-Ala-Asp) box polypeptide 31 | DDX31 | -3.108 | 0.00012 |
| Hs.510093 | Transcribed locus | --- | -3.113 | 0.00354 |
| Hs.551839 | hypothetical protein LOC284600 | LOC284600 | -3.155 | 0.04887 |
| Hs.159234 | forkhead box E1 (thyroid transcription factor 2) | FOXE1 | -3.184 | 0.01954 |
| Hs.271791 | Ataxia telangiectasia and Rad3 related | ATR | -3.186 | 0.00019 |
| Hs.525700 | small nuclear ribonucleoprotein polypeptide N | SNRPN | -3.377 | 0.00003 |
| Hs.371903 | glycophorin E | GYPE | -3.398 | 0.01643 |
| Hs.72901 | cyclin-dependent kinase inhibitor 2B | CDKN2B | -3.451 | 0.00363 |
| Hs.550240 | KIAA0485 protein | KIAA0485 | -3.458 | 0.00009 |
| Hs.513632 | Chromosome 16 open reading frame 49 | DKFZP434K046 | -3.468 | 0.03053 |
| Hs.2799 | Hyaluronan and proteoglycan link protein 1 | HAPLN1 | -3.471 | 0.00454 |
| Hs.44685 | ring finger protein 141 | RNF141 | -3.474 | 0.04947 |
| Hs.552134 | CDNA clone IMAGE:5301169, partial cds | --- | -3.481 | 0.00285 |
| Hs.549172 | Ceroid-lipofuscinosis, neuronal 6, late infantile, | CLN6 | -3.495 | 0.00665 |
| Hs.187422 | Chromosome 17 open reading frame 39 | C17orf39 | -3.534 | 0.01526 |
| Hs.54973 | cadherin-like 26 | CDH26 | -3.551 | 0.03073 |
| Hs.519523 | Serine proteinase inhibitor, clade, member 6 | SERPINB6 | -3.560 | 0.00003 |
| Hs.76561 | zinc finger protein 404 | ZNF404 | -3.603 | 0.00177 |
| Hs.170849 | hypothetical protein FLJ31846 | FLJ31846 | -3.614 | 0.00129 |
| Hs.149566 | Formin-like 2 | FMNL2 | -3.619 | 0.00358 |
| Hs.389945 | Hypothetical protein FLJ10300 | FLJ10300 | -3.641 | 0.00213 |
| Hs.242520 | uromodulin-like 1 | UMODL1 | -3.680 | 0.01025 |
| Hs.436380 | MAM domain containing 1 | MAMDC1 | -3.703 | 0.02430 |
| Hs.72307 | Transcribed locus | --- | -3.720 | 0.01181 |
| Hs.387861 | disintegrin-like-metalloprotease thrombospondin type 1 motif, 16 | ADAMTS16 | -4.056 | 0.01430 |
| Hs.50873 | Transcribed locus | --- | -4.356 | 0.00006 |
