# Supplementary material for: Evaluation of the Genetic Response of U937 and Jurkat Cells to 10-Nanosecond Electrical Pulses (nsEP)
Source: PLoS One. 2016 May 2;11(5):e0154555. doi: 10.1371/journal.pone.0154555 (PMC4852903; doi:10.1371/journal.pone.0154555)
Supplement: S3 Table — Genes were selected based on log ratio (≥2, or ≤ -2) and with a p-value of ≤ 0.05. (DOCX) [file pone.0154555.s010.docx]

Supplementary Table 3: Complete list of significant genes changing in U937 cells exposed to 44°C for 40 min (heat shock, positive control) Genes were selected based on log ratio (≥2, or ≤ -2) and with a p-value of ≤ 0.05.

| **UniGene ID** | **Gene name** | **Symbol** | **Fold change 150kVnsEP vs. SHAM** | **p-Value**  **150kv nsEP**  **vs. SHAM** |
| --- | --- | --- | --- | --- |
| Hs.3268 | heat shock 70kDa protein 6 (HSP70B') | HSPA6 | 7.679 | 0.00000 |
| Hs.3268 | heat shock 70kDa protein 6 (HSP70B') | HSPA6 | 7.506 | 0.00006 |
| --- | ribonuclease P RNA component H1 | RPPH1 | 7.316 | 0.01026 |
| Hs.414795 | serine (or cysteine) proteinase inhibitor, clade E | SERPINE1 | 6.910 | 0.00278 |
| Hs.370699 | hypothetical protein LOC284801 | LOC284801 | 6.117 | 0.00041 |
| Hs.547721 | CDNA FLJ12144 fis, clone MAMMA1000361 | --- | 5.883 | 0.01919 |
| Hs.55468 | cDNA clone RZPDo834C0824D | --- | 5.876 | 0.02756 |
| Hs.172928 | collagen, type I, alpha 1 | COL1A1 | 5.498 | 0.01386 |
| Hs.48029 | snail homolog 1 (Drosophila) | SNAI1 | 5.473 | 0.00002 |
| Hs.172928 | collagen, type I, alpha 1 | COL1A1 | 5.284 | 0.00011 |
| Hs.477070 | Casein kinase 1, delta | CSNK1D | 5.260 | 0.01904 |
| Hs.171695 | dual specificity phosphatase 1 | DUSP1 | 5.250 | 0.00009 |
| Hs.181244 | HLA complex group 4 pseudogene 6 | HCG4P6 | 5.236 | 0.00079 |
| Hs.117545 | Phosphodiesterase 4D, cAMP-specific | PDE4D | 5.212 | 0.00569 |
| Hs.370699 | hypothetical protein LOC284801 | LOC284801 | 5.168 | 0.00142 |
| Hs.241558 | Ariadne homolog 2 (Drosophila) | ARIH2 | 5.121 | 0.00717 |
| Hs.532144 | histone 1, H3d | HIST1H3D | 5.075 | 0.01661 |
| Hs.408767 | crystallin, alpha B | CRYAB | 5.036 | 0.00484 |
| --- | histone 1, H2bb | HIST1H2BB | 4.990 | 0.00571 |
| Hs.8395 | hypothetical protein FLJ10781 | FLJ10781 | 4.803 | 0.00667 |
| Hs.197644 | headcase homolog (Drosophila) | HECA | 4.794 | 0.00926 |
| Hs.520028 | heat shock 70kDa protein 1A 1B | HSPA1A & 1B | 4.790 | 0.00001 |
| --- | mitochondrial glutaminase pseudogene | GA | 4.777 | 0.00435 |
| Hs.288478 | Homo sapiens, clone IMAGE:4214313, mRNA | --- | 4.685 | 0.02339 |
| Hs.353515 | hypothetical protein FLJ34047 | FLJ34047 | 4.616 | 0.01879 |
| Hs.459652 | Chromosome 16 open reading frame 30 | C16orf30 | 4.598 | 0.01296 |
| Hs.170600 | GRB2-related adaptor protein-like | LOC400581 | 4.595 | 0.00857 |
| Hs.550144 | CDNA clone IMAGE:3621839, partial cds | --- | 4.532 | 0.01858 |
| Hs.171695 | dual specificity phosphatase 1 | DUSP1 | 4.513 | 0.00180 |
| Hs.307926 | G protein interaction factor 2-like mRNA sequence | --- | 4.508 | 0.03284 |
| Hs.326035 | Early growth response 1 | EGR1 | 4.438 | 0.00008 |
| Hs.406166 | hypothetical LOC400590 | LOC400590 | 4.401 | 0.00111 |
| Hs.250666 | hairy and enhancer of split 1, (Drosophila) | HES1 | 4.377 | 0.01107 |
| Hs.75678 | FBJ murine osteosarcoma viral oncogene homolog B | FOSB | 4.363 | 0.00207 |
| Hs.205439 | CDNA clone IMAGE:3958634, partial cds | --- | 4.336 | 0.02240 |
| Hs.369982 | insulin-like growth factor binding protein 5 | IGFBP5 | 4.310 | 0.00445 |
| Hs.551509 | Nardilysin (N-arginine dibasic convertase) | NRD1 | 4.293 | 0.01219 |
| Hs.78944 | regulator of G-protein signalling 2, 24kDa | RGS2 | 4.271 | 0.00030 |
| Hs.25647 | v-fos FBJ murine osteosarcoma viral oncogene homolog | FOS | 4.261 | 0.00689 |
| Hs.35828 | MAP/microtubule affinity-regulating kinase 3 | MARK3 | 4.254 | 0.00120 |
| Hs.1027 | Ras-related associated with diabetes | RRAD | 4.237 | 0.00018 |
| Hs.113684 | heat shock 60kDa protein 1 (chaperonin) | HSPD1 | 4.232 | 0.00479 |
| Hs.548468 | Full length insert cDNA clone YB66H06 | --- | 4.221 | 0.00784 |
| Hs.143873 | S100 calcium binding protein A10 | S100A10 | 4.196 | 0.01512 |
| Hs.493096 | Pre-B-cell leukemia transcription factor 1 | PBX1 | 4.160 | 0.00574 |
| Hs.434236 | hypothetical protein LOC338862 | LOC338862 | 4.154 | 0.00032 |
| Hs.114111 | Limkain beta 2 | FLJ22471 | 4.117 | 0.00646 |
| Hs.137274 | Transcribed locus | --- | 4.104 | 0.01843 |
| Hs.81848 | RAD21 homolog (S. pombe) | RAD21 | 4.092 | 0.02130 |
| Hs.382349 | MRNA; cDNA DKFZp667P1917 | --- | 4.087 | 0.00221 |
| Hs.256184 | RNA binding motif protein 21 | RBM21 | 4.072 | 0.03956 |
| Hs.279580 | KIAA1279 | KIAA1279 | 4.060 | 0.00692 |
| Hs.147054 | 3-hydroxymethyl-3-methylglutaryl-Coenzyme A lyase-like 1 | HMGCLL1 | 4.024 | 0.03060 |
| Hs.367688 | Homo sapiens, clone IMAGE:4794726, mRNA | --- | 4.011 | 0.04004 |
| Hs.272398 | ets variant gene 7 (TEL2 oncogene) | ETV7 | 3.948 | 0.04582 |
| Hs.132701 | coiled-coil domain containing 11 | CCDC11 | 3.941 | 0.00054 |
| Hs.487536 | Hypothetical gene | --- | 3.940 | 0.00030 |
| Hs.509410 | Zinc finger protein 323 | ZNF323 | 3.912 | 0.01171 |
| Hs.445350 | flavin containing monooxygenase 3 | FMO3 | 3.910 | 0.00391 |
| Hs.388746 | ubiquitin-conjugating enzyme E2U (putative) | UBE2U | 3.904 | 0.00563 |
| Hs.548708 | Homo sapiens, clone IMAGE:5312957, mRNA | --- | 3.903 | 0.00890 |
| Hs.520917 | Radical S-adenosyl methionine and flavodoxin domains 1 | RSAFD1 | 3.894 | 0.04221 |
| Hs.220950 | Forkhead box O3A | FOXO3A | 3.890 | 0.01098 |
| Hs.326035 | early growth response 1 | EGR1 | 3.885 | 0.00204 |
| Hs.134999 | histone 1, H2am | HIST1H2AM | 3.864 | 0.02276 |
| Hs.200266 | Transcribed locus | --- | 3.827 | 0.00861 |
| Hs.62772 | Transcribed locus | --- | 3.826 | 0.00147 |
| Hs.530274 | aldolase B, fructose-bisphosphate | ALDOB | 3.820 | 0.00969 |
| Hs.197872 | ankyrin repeat domain 24 | ANKRD24 | 3.810 | 0.02299 |
| Hs.371887 | histone 1, H2ba | HIST1H2BA | 3.797 | 0.00162 |
| Hs.386365 | homeo box D4 | HOXD4 | 3.781 | 0.00005 |
| Hs.104901 | Similar to small proline-rich protein 2C, | --- | 3.758 | 0.00419 |
| Hs.87191 | fibroblast growth factor 18 | FGF18 | 3.748 | 0.00424 |
| Hs.525105 | SLIT and NTRK-like family, member 6 | SLITRK6 | 3.737 | 0.02001 |
| Hs.150556 | Hypothetical protein FLJ43663 | FLJ43663 | 3.733 | 0.02616 |
| Hs.124675 | GTPase, IMAP family member 7 | GIMAP7 | 3.731 | 0.01981 |
| Hs.180919 | inhibitor of DNA binding 2, dominant negative helix-loop-helix | ID2- ID2B | 3.728 | 0.00564 |
| Hs.274541 | FSHD region gene 2 protein | FRG2 | 3.722 | 0.03728 |
| Hs.470117 | low density lipoprotein-related protein 1B | LRP1B | 3.704 | 0.00031 |
| Hs.441073 | Hypothetical gene supported by BC072410 | --- | 3.700 | 0.02616 |
| Hs.129136 | RAS and EF hand domain containing | RASEF | 3.695 | 0.01388 |
| Hs.200412 | epiplakin 1 | EPPK1 | 3.692 | 0.00333 |
| Hs.306242 | RAN binding protein 9 | RANBP9 | 3.675 | 0.00334 |
| Hs.1872 | phosphoenolpyruvate carboxykinase 1 (soluble) | PCK1 | 3.664 | 0.00237 |
| Hs.529392 | MRNA; cDNA | --- | 3.660 | 0.00458 |
| Hs.27705 | Transcribed locus | --- | 3.651 | 0.01706 |
| Hs.8619 | SRY (sex determining region Y)-box 18 | SOX18 | 3.646 | 0.00036 |
| Hs.369063 | Zic family member 2 | ZIC2 | 3.643 | 0.03030 |
| Hs.324341 | hypothetical protein FLJ39502 | FLJ39502 | 3.636 | 0.00069 |
| Hs.525704 | v-jun sarcoma virus 17 oncogene homolog | JUN | 3.627 | 0.00025 |
| Hs.287660 | CDNA: FLJ21384 fis, clone COL03354 | --- | 3.622 | 0.01244 |
| Hs.352250 | GATA binding protein 5 | GATA5 | 3.615 | 0.00026 |
| Hs.144333 | lin-7 homolog A (C. elegans) | LIN7A | 3.601 | 0.00735 |
| Hs.551809 | Transcribed locus | --- | 3.601 | 0.03617 |
| Hs.445884 | wingless-type MMTV integration site family, member 3 | WNT3 | 3.596 | 0.00837 |
| Hs.548066 | CDNA FLJ40517 fis, clone TESTI2046686 | --- | 3.595 | 0.00789 |
| Hs.533514 | Acetylserotonin O-methyltransferase-like | ASMTL | 3.583 | 0.02448 |
| Hs.78781 | vascular endothelial growth factor B | VEGFB | 3.572 | 0.00476 |
| Hs.133916 | Hypothetical protein LOC152485 | LOC152485 | 3.567 | 0.02122 |
| Hs.520028 | heat shock 70kDa protein 1A | HSPA1A | 3.562 | 0.00008 |
| Hs.112667 | dynein, axonemal, intermediate polypeptide 1 | DNAI1 | 3.561 | 0.00292 |
| Hs.434392 | hypothetical gene supported by BC044741 | LOC387872 | 3.560 | 0.03428 |
| Hs.15725 | immediate early response 5 | IER5 | 3.560 | 0.00000 |
| Hs.408182 | collagen, type II, alpha 1 | COL2A1 | 3.558 | 0.00631 |
| Hs.437966 | Peroxisomal membrane protein 3, 35kDa | PXMP3 | 3.550 | 0.00034 |
| Hs.535735 | Full length insert cDNA clone YY72H05 | --- | 3.547 | 0.00341 |
| Hs.439630 | Chromosome 5 open reading frame 12 | C5orf12 | 3.539 | 0.00157 |
| Hs.176225 | CDNA clone IMAGE:5541269, partial cds | --- | 3.537 | 0.00247 |
| Hs.436053 | Transcribed locus | --- | 3.536 | 0.04357 |
| Hs.443491 | Transcribed locus | --- | 3.532 | 0.00248 |
| Hs.326035 | early growth response 1 | EGR1 | 3.529 | 0.00007 |
| Hs.489722 | zinc finger protein 277 | ZNF277 | 3.521 | 0.00925 |
| Hs.42572 | aldehyde dehydrogenase 1 family, member L2 | ALDH1L2 | 3.511 | 0.01079 |
| Hs.513053 | DnaJ (Hsp40) homolog, subfamily A, member 4 | DNAJA4 | 3.500 | 0.03059 |
| Hs.193011 | CDNA clone IMAGE:4151570, partial cds | --- | 3.492 | 0.00473 |
| Hs.549033 | fibroblast growth factor 7 | FGF7 | 3.492 | 0.04450 |
| Hs.125715 | Muscleblind-like 2 (Drosophila) | MBNL2 | 3.485 | 0.00021 |
| Hs.96940 | hypothetical gene supported by NM_175889 | LOC439914 | 3.478 | 0.00719 |
| Hs.143080 | histone 1, H4b | HIST1H4B | 3.473 | 0.03482 |
| Hs.515210 | DnaJ (Hsp40) homolog, subfamily B, member 1 | DNAJB1 | 3.472 | 0.00041 |
| Hs.550492 | phospholipase A2, group IVB (cytosolic) | PLA2G4B | 3.461 | 0.01712 |
| Hs.252387 | cadherin, EGF LAG seven-pass G-type receptor 1 | CELSR1 | 3.457 | 0.00305 |
| Hs.525704 | v-jun sarcoma virus 17 oncogene homolog | JUN | 3.443 | 0.00011 |
| Hs.272011 | UDP-Gal:betaGlcNAc beta 1,4- galactosyltransferase, polypeptide 1 | B4GALT1 | 3.439 | 0.00027 |
| Hs.160673 | Ras homolog gene family, member H | RHOH | 3.436 | 0.04428 |
| Hs.529369 | actin filament associated protein | AFAP | 3.427 | 0.02671 |
| Hs.464137 | Acyl-Coenzyme A oxidase 1, palmitoyl | ACOX1 | 3.422 | 0.04603 |
| Hs.458425 | G protein-coupled receptor 109B | GPR109B | 3.421 | 0.02048 |
| Hs.465929 | calponin 1, basic, smooth muscle | CNN1 | 3.417 | 0.00883 |
| Hs.436625 | hypothetical protein FLJ25393 | FLJ25393 | 3.411 | 0.02355 |
| Hs.128453 | frizzled-related protein | FRZB | 3.411 | 0.00207 |
| Hs.249829 | hypothetical protein FLJ40126 | FLJ40126 | 3.409 | 0.01934 |
| Hs.107740 | Kruppel-like factor 2 (lung) | KLF2 | 3.407 | 0.00452 |
| Hs.349077 | poly(A)-specific ribonuclease (PARN)-like domain containing 1 | PNLDC1 | 3.405 | 0.00091 |
| Hs.211828 | Hepatocellular carcinoma-associated antigen 66 | HCA66 | 3.401 | 0.00480 |
| Hs.198760 | neurofilament, heavy polypeptide 200kDa | NEFH | 3.394 | 0.00869 |
| Hs.479658 | leucine rich repeat containing 7 | LRRC7 | 3.392 | 0.02149 |
| Hs.76095 | immediate early response 3 | IER3 | 3.387 | 0.00022 |
| Hs.73962 | EPH receptor A7 | EPHA7 | 3.383 | 0.01579 |
| Hs.62905 | Hypothetical protein FLJ14834 | FLJ14834 | 3.378 | 0.00921 |
| Hs.127735 | ankyrin repeat and SOCS box-containing 4 | ASB4 | 3.375 | 0.00214 |
| Hs.150033 | Transcribed locus | --- | 3.364 | 0.01127 |
| Hs.552486 | Homo sapiens, clone IMAGE:4815589, mRNA | --- | 3.361 | 0.01674 |
| Hs.498519 | Homo sapiens, clone IMAGE:5261280, mRNA | --- | 3.361 | 0.00563 |
| Hs.525056 | FLJ40296 protein | FLJ40296 | 3.361 | 0.00711 |
| Hs.274402 | heat shock 70kDa protein 1B | HSPA1B | 3.359 | 0.00016 |
| Hs.504657 | C-type lectin domain family 4, member A | CLEC4A | 3.357 | 0.00787 |
| Hs.534498 | H2A histone family, member B3 | H2AFB3 | 3.351 | 0.02229 |
| Hs.114198 | Mitogen-activated protein kinase kinase 5 | MAP2K5 | 3.344 | 0.01796 |
| Hs.202542 | hypothetical protein FLJ20753 | FLJ20753 | 3.343 | 0.01101 |
| Hs.128201 | Mitochondrial ribosomal protein S25 | MRPS25 | 3.338 | 0.01204 |
| Hs.202676 | Synaptonemal complex protein 2 | SYCP2 | 3.335 | 0.03172 |
| Hs.149413 | protein phosphatase 3 (formerly 2B), | PPP3CC | 3.329 | 0.00264 |
| Hs.465529 | midnolin | MIDN | 3.327 | 0.01798 |
| Hs.190537 | CDNA FLJ26164 fis, clone ADG02723 | --- | 3.327 | 0.01647 |
| Hs.482301 | Hypothetical protein FLJ13611 | FLJ13611 | 3.324 | 0.01965 |
| Hs.386567 | guanylate binding protein 2, interferon-inducible | GBP2 | 3.308 | 0.00527 |
| Hs.315369 | aquaporin 4 | AQP4 | 3.299 | 0.02233 |
| Hs.496222 | angiopoietin-like 1 | ANGPTL1 | 3.295 | 0.00099 |
| Hs.163867 | CD14 antigen | CD14 | 3.277 | 0.01144 |
| Hs.515169 | Hypothetical protein FLJ20244 | FLJ20244 | 3.277 | 0.02408 |
| Hs.109655 | Sex comb on midleg-like 1 (Drosophila) | SCML1 | 3.273 | 0.00278 |
| Hs.252451 | Semaphoring 3A | SEMA3A | 3.273 | 0.01522 |
| Hs.385632 | Homo sapiens, clone IMAGE:4829538, mRNA | --- | 3.272 | 0.04262 |
| Hs.375806 | CDNA clone IMAGE:5285293, partial cds | --- | 3.271 | 0.04385 |
| Hs.407349 | Full length insert cDNA clone ZD86G04 | --- | 3.267 | 0.00747 |
| Hs.14706 | Clone DNA57836 GLPG464 (UNQ464) mRNA, | --- | 3.252 | 0.02458 |
| Hs.525589 | Maternally expressed 3 | MEG3 | 3.252 | 0.00006 |
| Hs.532144 | histone 1, H3d | HIST1H3D | 3.247 | 0.02083 |
| Hs.533710 | fibronectin leucine rich transmembrane protein 2 | FLRT2 | 3.234 | 0.00897 |
| Hs.535889 | Homo sapiens, clone IMAGE:5261153, mRNA | --- | 3.230 | 0.00306 |
| Hs.114198 | Mitogen-activated protein kinase kinase 5 | MAP2K5 | 3.228 | 0.00496 |
| Hs.549184 | a disintegrin-like and metalloprotease (reprolysin type) with thrombospondin type 1 motif, 9 | ADAMTS9 | 3.228 | 0.00249 |
| Hs.549182 | PR domain containing 11 | PRDM11 | 3.224 | 0.00781 |
| Hs.260903 | Protein phosphatase 1, regulatory (inhibitor) subunit 14B | ELF2 | 3.219 | 0.01076 |
| Hs.252433 | hypothetical protein LOC339803 | LOC339803 | 3.216 | 0.00039 |
| Hs.532901 | hypothetical protein FLJ32954 | FLJ32954 | 3.209 | 0.01271 |
| Hs.381372 | defensin, beta 114 | DEFB114 | 3.207 | 0.04691 |
| Hs.387207 | sarcoglycan, delta | SGCD | 3.203 | 0.00113 |
| Hs.145372 | Homo sapiens, clone IMAGE:5267399, mRNA | --- | 3.200 | 0.00047 |
| Hs.194121 | RNA terminal phosphate cyclase-like 1 | RCL1 | 3.192 | 0.02226 |
| Hs.486095 | Chromosome 6 open reading frame 210 | C6orf210 | 3.190 | 0.04586 |
| Hs.515210 | DnaJ (Hsp40) homolog, subfamily B, member 1 | DNAJB1 | 3.186 | 0.00748 |
| Hs.523309 | BCL2-associated athanogene 3 | BAG3 | 3.185 | 0.00826 |
| Hs.389945 | Hypothetical protein FLJ10300 | FLJ10300 | 3.182 | 0.00136 |
| Hs.529095 | LOC440156 | --- | 3.179 | 0.01133 |
| Hs.525171 | CDNA FLJ33739 fis, clone BRAWH2018601 | --- | 3.173 | 0.02030 |
| Hs.129195 | Family with sequence similarity 53, member B | FAM53B | 3.171 | 0.00908 |
| Hs.125715 | Muscleblind-like 2 (Drosophila) | MBNL2 | 3.166 | 0.00651 |
| Hs.346489 | CDNA FLJ35222 fis, clone PROST2000835 | --- | 3.166 | 0.04068 |
| Hs.534352 | syntaxin binding protein 2 | STXBP2 | 3.164 | 0.00343 |
| Hs.284707 | Homo sapiens, clone IMAGE:5259731, mRNA | --- | 3.159 | 0.01666 |
| Hs.464243 | Ubiquitin specific protease 36 | USP36 | 3.158 | 0.01200 |
| Hs.381370 | G protein-coupled receptor 111 | GPR111 | 3.157 | 0.00067 |
| Hs.520189 | ELOVL family member 5, elongation of long chain fatty acids | ELOVL5 | 3.152 | 0.00072 |
| Hs.17267 | chromosome 9 open reading frame 93 | C9orf93 | 3.149 | 0.01507 |
| Hs.10041 | CDNA FLJ37509 fis, clone BRCAN1000065 | --- | 3.149 | 0.04068 |
| Hs.120260 | Fc receptor-like 4 /// Fc receptor-like 4 | FCRL4 | 3.147 | 0.00155 |
| Hs.547057 | hypothetical protein FLJ90036 | FLJ90036 | 3.141 | 0.03488 |
| Hs.356226 | hypothetical protein LOC134121 | LOC134121 | 3.140 | 0.00115 |
| Hs.496916 | Clone 161455-2-3 B cell expressed mRNA | --- | 3.138 | 0.01915 |
| Hs.326728 | chromosome 19 open reading frame 30 | C19orf30 | 3.137 | 0.04358 |
| Hs.22815 | CDNA FLJ14143 fis, clone MAMMA1002892 | --- | 3.137 | 0.02929 |
| Hs.380334 | Zinc finger protein 148 (pHZ-52) | ZNF148 | 3.133 | 0.01462 |
| Hs.510324 | Quaking homolog, KH domain RNA binding | QKI | 3.122 | 0.01904 |
| Hs.550895 | Full length insert cDNA clone ZD65G03 | --- | 3.121 | 0.00163 |
| Hs.488157 | Williams Beuren syndrome chromosome region 19 | WBSCR19 | 3.120 | 0.00636 |
| Hs.381568 | Immunoglobulin J polypeptide, | IGJ | 3.116 | 0.03179 |
| Hs.459927 | Prothymosin, alpha (gene sequence 28) | PTMA | 3.113 | 0.02444 |
| Hs.266728 | hypothetical protein FLJ13639 | FLJ13639 | 3.098 | 0.00208 |
| Hs.23650 | similar to lymphocyte antigen 6 complex, | LOC112476 | 3.098 | 0.03456 |
| Hs.444245 | Transcribed locus | --- | 3.090 | 0.02769 |
| Hs.279583 | DORA reverse strand protein 1 | DREV1 | 3.089 | 0.00577 |
| Hs.446484 | Casein kinase 2, alpha 1 polypeptide | CSNK2A1 | 3.087 | 0.01588 |
| Hs.523774 | EH-domain containing 1 | EHD1 | 3.086 | 0.03178 |
| Hs.513661 | CDNA clone IMAGE:4795866, partial cds | --- | 3.081 | 0.01601 |
| Hs.407114 | MRNA; cDNA DKFZp313O2039 | --- | 3.078 | 0.00230 |
| Hs.167451 | KIAA0999 protein | KIAA0999 | 3.073 | 0.02816 |
| Hs.42091 | secretin receptor | SCTR | 3.073 | 0.00142 |
| Hs.543893 | RNA, U19 small nucleolar | RNU19 | 3.072 | 0.01882 |
| Hs.379253 | Full length insert cDNA clone ZD69D05 | --- | 3.072 | 0.00085 |
| Hs.464201 | Transcribed locus | --- | 3.071 | 0.00640 |
| Hs.547057 | Hypothetical protein FLJ90036 | FLJ90036 | 3.069 | 0.00289 |
| Hs.63788 | Propionyl Coenzyme A carboxylase, beta polypeptide | PCCB | 3.066 | 0.00195 |
| Hs.493793 | hypothetical protein LOC158381 | LOC158381 | 3.065 | 0.00015 |
| Hs.446192 | contactin associated protein-like 2 | CNTNAP2 | 3.064 | 0.00014 |
| Hs.413801 | Proteasome (prosome, macropain) activator subunit 4 | PSME4 | 3.059 | 0.00894 |
| Hs.156727 | Ankylosis, progressive homolog (mouse) | ANKH | 3.055 | 0.02690 |
| Hs.368084 | Leucine-rich PPR-motif containing | LRPPRC | 3.055 | 0.01255 |
| Hs.550510 | thioesterase, adipose associated | THEA | 3.047 | 0.04402 |
| Hs.171909 | U2(RNU2) small nuclear RNA auxiliary factor 1-like 2 | U2AF1L2 | 3.045 | 0.02214 |
| Hs.525589 | Maternally expressed 3 | MEG3 | 3.042 | 0.00155 |
| Hs.348434 | Homo sapiens, clone IMAGE:5286779, mRNA | --- | 3.040 | 0.00796 |
| Hs.458287 | putative N-acetyltransferase Camello 2 | CML2 | 3.039 | 0.01014 |
| Hs.12333 | Ring finger protein 13 | RNF13 | 3.039 | 0.00908 |
| Hs.488409 | hypothetical protein FLJ39639 | FLJ39639 | 3.037 | 0.00145 |
| Hs.449598 | Hepatitis B surface antigen antibody variable domain | --- | 3.037 | 0.00923 |
| Hs.436057 | Transcribed locus | --- | 3.036 | 0.00051 |
| Hs.550529 | Caspase recruitment domain family, member 14 | CARD14 | 3.034 | 0.03048 |
| Hs.435765 | glutamyl aminopeptidase (aminopeptidase A) | ENPEP | 3.032 | 0.02835 |
| Hs.168427 | Transcribed locus | --- | 3.029 | 0.01203 |
| Hs.133421 | Leukemia inhibitory factor receptor | LIFR | 3.027 | 0.03706 |
| Hs.386421 | CDNA: FLJ20892 fis, clone ADKA03430 | --- | 3.027 | 0.02101 |
| Hs.90093 | Heat shock 70kDa protein 4 | HSPA4 | 3.027 | 0.01412 |
| Hs.13704 | Transcribed locus | --- | 3.025 | 0.00011 |
| Hs.48924 | armadillo repeat containing, X-linked 2 | ARMCX2 | 3.023 | 0.00714 |
| Hs.21035 | UDP-N-acetyl-alpha-D-galactosamine:polypeptide N-acetylgalactosaminyltransferase-like 1 | GALNTL1 | 3.022 | 0.01064 |
| Hs.289105 | synovial sarcoma, X breakpoint 2 | SSX2 | 3.018 | 0.01133 |
| Hs.496138 | Homo sapiens, clone IMAGE:5399737, mRNA | --- | 3.016 | 0.01343 |
| Hs.147062 | cyclic nucleotide gated channel beta 1 | CNGB1 | 3.013 | 0.00558 |
| Hs.279583 | DORA reverse strand protein 1 | DREV1 | 2.999 | 0.02631 |
| Hs.389311 | Hypothetical protein MGC13057 | MGC13057 | 2.999 | 0.00193 |
| Hs.434660 | hypothetical protein LOC285045 | LOC285045 | 2.997 | 0.04821 |
| Hs.250618 | UL16 binding protein 2 | ULBP2 | 2.997 | 0.03519 |
| Hs.209249 | Transcribed locus | --- | 2.997 | 0.02227 |
| Hs.512805 | NYD-SP28 protein | NYD-SP28 | 2.994 | 0.02664 |
| Hs.253305 | soluble liver antigen/liver pancreas antigen | SLA/LP | 2.992 | 0.00576 |
| Hs.262558 | Transcribed locus | --- | 2.990 | 0.03160 |
| Hs.444213 | transducin-like enhancer of split 4 (E(sp1) | TLE4 | 2.990 | 0.04191 |
| Hs.475812 | Source of immunodominant MHC-associated peptides | SIMP | 2.984 | 0.02608 |
| Hs.389669 | similar to RIKEN cDNA 2600017H02 | LOC92162 | 2.982 | 0.00307 |
| Hs.153934 | Core-binding factor, runt domain, alpha subunit 2; translocated to, | CBFA2T2 | 2.975 | 0.01672 |
| Hs.233936 | LOC440476 | --- | 2.972 | 0.03656 |
| Hs.2563 | tachykinin, precursor 1 | TAC1 | 2.971 | 0.00655 |
| Hs.531188 | olfactory receptor, family 4, subfamily D, member 1 | OR4D1 | 2.969 | 0.01398 |
| Hs.44277 | Hypothetical protein MGC14816 | MGC14816 | 2.965 | 0.03461 |
| Hs.407480 | Homo sapiens, clone IMAGE:5265890, mRNA | --- | 2.961 | 0.01523 |
| Hs.533582 | CDC14 cell division cycle 14 homolog A | CDC14A | 2.960 | 0.04818 |
| Hs.157106 | Jumonji domain containing 2C | JMJD2C | 2.957 | 0.02033 |
| Hs.550815 | CDNA clone IMAGE:6602628, partial cds | --- | 2.953 | 0.03685 |
| Hs.134035 | CD5 antigen-like | CD5L | 2.950 | 0.00124 |
| Hs.520217 | LOC441157 | --- | 2.940 | 0.00005 |
| Hs.514516 | hypothetical LOC400622 | LOC400622 | 2.939 | 0.03178 |
| Hs.319301 | G protein-coupled receptor 89 | GPR89 | 2.937 | 0.04795 |
| Hs.125715 | Muscleblind-like 2 (Drosophila) | MBNL2 | 2.937 | 0.00073 |
| Hs.446240 | Protein kinase C binding protein 1 | PRKCBP1 | 2.936 | 0.03342 |
| Hs.381715 | T-box 5 | TBX5 | 2.932 | 0.00092 |
| Hs.13680 | Fring | RFFL | 2.928 | 0.02964 |
| Hs.197644 | Headcase homolog (Drosophila) | HECA | 2.924 | 0.02378 |
| Hs.527657 | cDNA clone CS0DI011YD16 of Placenta Cot 25- | --- | 2.922 | 0.00502 |
| Hs.407358 | Full length insert cDNA clone YZ87H02 | --- | 2.922 | 0.04694 |
| Hs.306842 | CDNA: FLJ22734 fis, clone HUV00109 | --- | 2.917 | 0.02434 |
| Hs.429179 | family with sequence similarity 43, member B | FAM43B | 2.914 | 0.02811 |
| Hs.482589 | CDNA FLJ40764 fis, clone TRACH2002954 | --- | 2.914 | 0.01366 |
| --- | early lymphoid activation protein | EPAG | 2.914 | 0.04583 |
| Hs.129136 | RAS and EF hand domain containing | RASEF | 2.913 | 0.03317 |
| Hs.239181 | chromosome 6 open reading frame 194 | C6orf194 | 2.911 | 0.02961 |
| Hs.198003 | Sarcosine dehydrogenase | SARDH | 2.903 | 0.02601 |
| Hs.369761 | DAZ associated protein 2 | DAZAP2 | 2.900 | 0.00861 |
| Hs.502328 | CD44 antigen | CD44 | 2.900 | 0.02215 |
| Hs.552900 | CDNA clone IMAGE:5186324, partial cds | --- | 2.898 | 0.02691 |
| Hs.434401 | Zinc finger protein 638 | ZNF638 | 2.897 | 0.00569 |
| Hs.549132 | olfactory receptor, family 2, subfamily L, member 2 | OR2L2 | 2.893 | 0.00082 |
| Hs.213289 | low density lipoprotein receptor | LDLR | 2.892 | 0.01138 |
| Hs.446336 | paxillin | PXN | 2.889 | 0.04400 |
| Hs.440332 | Ets2 repressor factor | ERF | 2.887 | 0.02172 |
| Hs.446690 | Hypothetical protein MGC20235 | MGC20235 | 2.884 | 0.00171 |
| Hs.433445 | jagged 2 | JAG2 | 2.880 | 0.04909 |
| Hs.325890 | Human immunodeficiency virus type I enhancer binding protein 3 | HIVEP3 | 2.874 | 0.00955 |
| Hs.475392 | 30 kDa protein | LOC55831 | 2.873 | 0.02888 |
| Hs.98328 | hypothetical protein MGC13040 | MGC13040 | 2.871 | 0.01025 |
| Hs.300897 | CDNA FLJ11624 fis, clone HEMBA1004193 | --- | 2.870 | 0.04908 |
| Hs.268788 | nebulin-related anchoring protein | NRAP | 2.867 | 0.00974 |
| Hs.404741 | Nuclear factor (erythroid-derived 2)-like 3 | NFE2L3 | 2.867 | 0.00361 |
| Hs.396189 | hypothetical protein FLJ39575 | FLJ39575 | 2.864 | 0.00470 |
| Hs.529133 | Full length insert cDNA clone ZA96G04 | --- | 2.864 | 0.00328 |
| Hs.293184 | Transcribed locus | --- | 2.854 | 0.02620 |
| Hs.467236 | zinc finger protein 160 | ZNF160 | 2.854 | 0.01399 |
| Hs.382989 | Homo sapiens, clone IMAGE:4616837, mRNA | --- | 2.854 | 0.04704 |
| Hs.291235 | hypothetical protein LOC200008 | LOC200008 | 2.848 | 0.00345 |
| Hs.508757 | Hypothetical protein LOC283501 | LOC283501 | 2.846 | 0.00153 |
| Hs.125715 | Muscleblind-like 2 (Drosophila) | MBNL2 | 2.840 | 0.00565 |
| Hs.477869 | phospholipid scramblase 4 | PLSCR4 | 2.840 | 0.00992 |
| Hs.233325 | hemochromatosis | HFE | 2.837 | 0.01877 |
| Hs.534601 | RPL13-2 pseudogene | LOC283345 | 2.833 | 0.02846 |
| Hs.407643 | protocadherin 9 | PCDH9 | 2.832 | 0.04429 |
| Hs.152385 | Hypothetical protein FLJ10980 | FLJ10980 | 2.831 | 0.02019 |
| Hs.551042 | Transcribed locus | --- | 2.831 | 0.00435 |
| Hs.98594 | Rho guanine nucleotide exchange factor (GEF) 10 | ARHGEF10 | 2.830 | 0.03372 |
| Hs.291587 | AT rich interactive domain 1B (SWI1-like) | ARID1B | 2.829 | 0.04426 |
| Hs.309539 | N-deacetylase/N-sulfotransferase 4 | NDST4 | 2.828 | 0.00190 |
| Hs.317243 | Hypothetical 55.1 kDa protein F09G8.5 | LOC220074 | 2.826 | 0.03896 |
| Hs.444950 | TBC1 domain family, member 10A | TBC1D10 | 2.824 | 0.02818 |
| Hs.384598 | serine (or cysteine) proteinase inhibitor, clade G | SERPING1 | 2.820 | 0.04592 |
| Hs.481478 | Solute carrier family 6, member 19 | SLC6A19 | 2.812 | 0.00490 |
| Hs.467370 | Similar to zinc finger protein KIAA1956 | --- | 2.811 | 0.02598 |
| Hs.525704 | v-jun sarcoma virus 17 oncogene homolog | JUN | 2.806 | 0.00012 |
| Hs.518994 | NMDA receptor regulated 1 | NARG1 | 2.806 | 0.02466 |
| Hs.459842 | PRbBP-39 | --- | 2.806 | 0.01811 |
| Hs.474880 | Hypothetical LOC388903 | --- | 2.804 | 0.03295 |
| Hs.146559 | angiopoietin-like 7 | ANGPTL7 | 2.803 | 0.03388 |
| Hs.385570 | Homo sapiens, clone IMAGE:4830126, mRNA | --- | 2.803 | 0.03029 |
| Hs.160572 | CDNA FLJ12030 fis, clone HEMBB1001868 | --- | 2.802 | 0.00881 |
| Hs.20395 | chromodomain helicase DNA binding protein 7 | CHD7 | 2.800 | 0.01032 |
| Hs.553298 | CDNA FLJ32896 fis, clone TESTI2005155 | --- | 2.798 | 0.00163 |
| Hs.444407 | Paired immunoglobin-like type 2 receptor alpha | PILRA | 2.792 | 0.02852 |
| Hs.192233 | periplakin | PPL | 2.792 | 0.02096 |
| Hs.373938 | butyrophilin, subfamily 2, member A2 | BTN2A2 | 2.789 | 0.02411 |
| Hs.466664 | ryanodine receptor 1 (skeletal) | RYR1 | 2.786 | 0.00006 |
| Hs.65756 | regulator of G-protein signalling 11 | RGS11 | 2.785 | 0.03497 |
| Hs.347537 | Chromosome 9 open reading frame 4 | C9orf4 | 2.783 | 0.01104 |
| Hs.132593 | PR domain containing 5 | PRDM5 | 2.783 | 0.02322 |
| Hs.535864 | Transcribed locus | --- | 2.783 | 0.03764 |
| Hs.252351 | HERV-H LTR-associating 2 | HHLA2 | 2.781 | 0.03525 |
| Hs.546917 | CDNA: FLJ23438 fis, clone HRC13275 | --- | 2.780 | 0.04655 |
| Hs.323537 | WD repeat domain 54 | FLJ12953 | 2.779 | 0.01967 |
| Hs.125240 | Kruppel-like factor 14 | KLF14 | 2.778 | 0.04408 |
| Hs.547764 | MRNA full length insert cDNA clone 2344436 | --- | 2.778 | 0.00372 |
| Hs.534318 | goosecoid-like | GSCL | 2.777 | 0.04049 |
| Hs.492974 | WNT1 inducible signaling pathway protein 1 | WISP1 | 2.766 | 0.02531 |
| Hs.383169 | Immunoglobulin heavy chain V region (Humha448) | --- | 2.766 | 0.04452 |
| Hs.505007 | SRY (sex determining region Y)-box 5 | SOX5 | 2.764 | 0.02483 |
| Hs.21107 | Neuroligin 4, X-linked | NLGN4X | 2.761 | 0.04333 |
| Hs.294147 | chromosome 9 open reading frame 111 | C9orf111 | 2.758 | 0.02199 |
| Hs.534190 | Chromosome 9 open reading frame 85 | C9orf85 | 2.756 | 0.01306 |
| Hs.201034 | netrin 4 | NTN4 | 2.755 | 0.00379 |
| Hs.429294 | ATP-binding cassette, sub-family A (ABC1), member 1 | ABCA1 | 2.755 | 0.00543 |
| Hs.523702 | membrane-spanning 4-domains, subfamily A, member 6A | MS4A6A | 2.755 | 0.00320 |
| Hs.470549 | hypothetical protein MGC2610 | MGC2610 | 2.755 | 0.00116 |
| Hs.269254 | Ubiquitin specific protease 49 | MGC20741 | 2.750 | 0.00585 |
| Hs.446077 | solute carrier family 38, member 4 | SLC38A4 | 2.749 | 0.02861 |
| Hs.535723 | BC048124 | LOC348808 | 2.746 | 0.01268 |
| Hs.116796 | DIX domain containing 1 | DIXDC1 | 2.745 | 0.00185 |
| Hs.306716 | CDNA: FLJ21228 fis, clone COL00739 | --- | 2.743 | 0.02868 |
| Hs.472031 | ubiquitin-conjugating enzyme E2D 3 | UBE2D3 | 2.743 | 0.00988 |
| Hs.434175 | hypothetical protein LOC151657 | LOC151657 | 2.741 | 0.00976 |
| Hs.18343 | Transcribed locus | --- | 2.738 | 0.00098 |
| Hs.2250 | leukemia inhibitory factor | LIF | 2.736 | 0.00974 |
| Hs.515048 | Adaptor-related protein complex 4, beta 1 subunit | AP4B1 | 2.734 | 0.01695 |
| Hs.533831 | Chromosome 13 open reading frame 22 | C13orf22 | 2.734 | 0.00293 |
| Hs.525392 | Spectrin repeat containing, nuclear envelope 2 | SYNE2 | 2.734 | 0.00505 |
| Hs.432400 | agouti signaling protein, nonagouti homolog ( | ASIP | 2.733 | 0.00797 |
| Hs.476782 | Eukaryotic translation initiation factor 4E member 3 | EIF4E3 | 2.727 | 0.01782 |
| Hs.407510 | Homo sapiens, clone IMAGE:5269873, mRNA | --- | 2.722 | 0.01499 |
| Hs.132648 | Hypothetical protein FLJ37964 | FLJ37964 | 2.722 | 0.00729 |
| Hs.24950 | Regulator of G-protein signalling 5 | RGS5 | 2.721 | 0.00727 |
| Hs.527973 | suppressor of cytokine signaling 3 | SOCS3 | 2.720 | 0.02146 |
| Hs.123232 | Chromosome 14 open reading frame 143 | C14orf143 | 2.720 | 0.00573 |
| Hs.134830 | Collagen, type VIII, alpha 1 | COL8A1 | 2.718 | 0.00657 |
| Hs.194695 | DIRAS family, GTP-binding RAS-like 3 | DIRAS3 | 2.717 | 0.02048 |
| Hs.53985 | glycoprotein 2 (zymogen granule membrane) | GP2 | 2.716 | 0.02726 |
| Hs.400688 | sperm-specific protein Izumo | IZUMO | 2.715 | 0.01079 |
| Hs.522818 | L1 cell adhesion molecule | L1CAM | 2.712 | 0.00250 |
| Hs.431498 | Forkhead box P1 | FOXP1 | 2.710 | 0.03944 |
| Hs.434614 | Homo sapiens, clone IMAGE:4822760, mRNA | --- | 2.708 | 0.03774 |
| Hs.58367 | glypican 4 | GPC4 | 2.706 | 0.00395 |
| Hs.511991 | nephronophthisis 3 (adolescent) | NPHP3 | 2.704 | 0.00571 |
| Hs.519180 | Homo sapiens, clone IMAGE:5271913, mRNA | --- | 2.704 | 0.00029 |
| Hs.1510 | interferon, alpha 4 | IFNA4 | 2.700 | 0.03797 |
| --- | hypothetical protein LOC146429 | LOC146429 | 2.697 | 0.00683 |
| Hs.112444 | TATA box binding protein (TBP)-associated factor | TAF11 | 2.696 | 0.04462 |
| Hs.352171 | Homo sapiens, clone IMAGE:4293443, mRNA | --- | 2.695 | 0.04111 |
| Hs.41688 | dual specificity phosphatase 8 | DUSP8 | 2.693 | 0.01031 |
| Hs.334910 | Similar to guanidinoacetate methyltransferase; | GAMT | 2.693 | 0.03872 |
| Hs.32417 | specifically androgen-regulated protein | SARG | 2.692 | 0.01085 |
| --- | hypothetical protein LOC338651 | LOC338651 | 2.691 | 0.00895 |
| Hs.28391 | latrophilin 3 | LPHN3 | 2.690 | 0.02306 |
| Hs.476389 | Chromosome 1 open reading frame 1 | C1orf1 | 2.685 | 0.01025 |
| Hs.385546 | homeobox C14 | LOC360030 | 2.685 | 0.01973 |
| Hs.49230 | vesicular membrane protein p24 | VMP | 2.684 | 0.03265 |
| Hs.516245 | hypothetical protein LOC151438 | LOC151438 | 2.683 | 0.03232 |
| Hs.445000 | prostaglandin E receptor 3 (subtype EP3) | PTGER3 | 2.683 | 0.00208 |
| Hs.293970 | aldehyde dehydrogenase 6 family, member A1 | ALDH6A1 | 2.679 | 0.03333 |
| Hs.536420 | Similar to LOC161538, clone IMAGE:5199550, | --- | 2.674 | 0.01737 |
| Hs.477425 | Solute carrier family 12, member 8 | SLC12A8 | 2.672 | 0.03757 |
| Hs.288262 | hypothetical gene | FLJ31485 | 2.671 | 0.01123 |
| Hs.23439 | hypothetical protein FLJ39370 | FLJ39370 | 2.668 | 0.03262 |
| Hs.269364 | Rhesus blood group, CcEe antigens & D antigen | RHCE /// RHD | 2.666 | 0.00059 |
| Hs.213137 | Chondroitin sulfate synthase 3 | CSS3 | 2.663 | 0.01239 |
| Hs.494751 | Dynamin 3 | DNM3 | 2.660 | 0.02679 |
| Hs.323583 | hypothetical protein DKFZp434L142 | DKFZp434L142 | 2.659 | 0.01641 |
| --- | hypothetical protein MGC15705 | MGC15705 | 2.658 | 0.00213 |
| Hs.523760 | Protein phosphatase 1, regulatory (inhibitor) subunit 14B | PPP1R14B | 2.655 | 0.00427 |
| Hs.270492 | B melanoma antigen family, member 4 | MLL3 | 2.655 | 0.01565 |
| Hs.368282 | KIAA0564 protein | KIAA0564 | 2.654 | 0.04546 |
| Hs.454779 | Similar to stromal cell derived factor receptor 2 | --- | 2.652 | 0.01830 |
| Hs.484738 | Myosin regulatory light chain interacting protein | MYLIP | 2.651 | 0.02680 |
| Hs.184507 | zymogen granule protein 16 | ZG16 | 2.649 | 0.04750 |
| Hs.208125 | CDNA FLJ30446 fis, clone BRACE2009255 | --- | 2.648 | 0.01400 |
| Hs.134353 | hypothetical protein LOC284402 | LOC284402 | 2.647 | 0.00084 |
| Hs.27214 | K+ voltage-gated channel, Shaw-related subfamily, member 2 | KCNC2 | 2.644 | 0.00438 |
| Hs.432706 | hypothetical protein DKFZp434D2328 | LOC91526 | 2.641 | 0.00270 |
| Hs.528664 | potassium channel, subfamily K, member 15 | KCNK15 | 2.640 | 0.01147 |
| Hs.406290 | Homo sapiens, clone IMAGE:4815265, mRNA | --- | 2.638 | 0.00031 |
| Hs.460217 | Hypothetical protein MGC35048 | MGC35048 | 2.637 | 0.03714 |
| Hs.524719 | pancreatic polypeptide receptor 1 | PPYR1 | 2.636 | 0.00233 |
| Hs.515985 | Smooth muscle myosin heavy chain 11 isoform SM1-like | LOC129285 | 2.636 | 0.04653 |
| Hs.267038 | premature ovarian failure, 1B | POF1B | 2.636 | 0.00315 |
| Hs.306307 | Rho-associated, coiled-coil containing protein kinase 1 | ROCK1 | 2.635 | 0.01119 |
| Hs.397978 | Abhydrolase domain containing 3 | ABHD3 | 2.634 | 0.01477 |
| Hs.546242 | calcitonin/calcitonin-related polypeptide, alpha | CALCA | 2.633 | 0.01032 |
| Hs.344165 | Ubiquitin-conjugating enzyme E2H | UBE2H | 2.633 | 0.01576 |
| Hs.250770 | kallikrein 15 | KLK15 | 2.633 | 0.02152 |
| Hs.435948 | ATPase family, AAA domain containing 1 | ATAD1 | 2.629 | 0.00020 |
| Hs.511504 | transcription factor 12 | TCF12 | 2.628 | 0.03800 |
| Hs.385753 | Homo sapiens, clone IMAGE:4796629, mRNA | --- | 2.626 | 0.03656 |
| Hs.2355 | B melanoma antigen | BAGE | 2.618 | 0.01690 |
| Hs.79033 | glutaminyl-peptide cyclotransferase | QPCT | 2.617 | 0.03151 |
| Hs.405659 | piwi-like 1 (Drosophila) | PIWIL1 | 2.613 | 0.03919 |
| Hs.549350 | similar to C10orf94 protein | LOC400547 | 2.612 | 0.02934 |
| Hs.546961 | Homo sapiens, clone IMAGE:5528155, mRNA | --- | 2.609 | 0.03958 |
| Hs.73853 | bone morphogenetic protein 2 | BMP2 | 2.608 | 0.01134 |
| Hs.71657 | Transcribed locus | --- | 2.604 | 0.00872 |
| Hs.482837 | hypothetical protein FLJ25680 | FLJ25680 | 2.602 | 0.01712 |
| Hs.291993 | Transcribed locus | --- | 2.600 | 0.00682 |
| Hs.499953 | H2A histone family, member Y2 | H2AFY2 | 2.600 | 0.03593 |
| Hs.306230 | leukocyte immunoglobulin-like receptor, subfamily B | LILRB5 | 2.600 | 0.00728 |
| Hs.18442 | E-1 enzyme | MASA | 2.597 | 0.00074 |
| Hs.56186 | EGF-like-domain, multiple 3 | EGFL3 | 2.597 | 0.02933 |
| Hs.310545 | synaptotagmin I | SYT1 | 2.596 | 0.01899 |
| Hs.548140 | MRNA; cDNA DKFZp686K1037 | --- | 2.595 | 0.00436 |
| Hs.89387 | cancer susceptibility candidate 2 | CASC2 | 2.593 | 0.04693 |
| Hs.80288 | heat shock 70kDa protein 1-like | HSPA1L | 2.593 | 0.00035 |
| Hs.194766 | Seizure related 6 homolog (mouse)-like | SEZ6L | 2.593 | 0.01970 |
| Hs.143746 | CDNA FLJ38433 fis, clone FEBRA2014578 | --- | 2.591 | 0.01911 |
| Hs.548634 | CDNA clone IMAGE:5296266, partial cds | --- | 2.590 | 0.04031 |
| Hs.158951 | Transcribed locus | --- | 2.589 | 0.00373 |
| Hs.500483 | Actin, alpha 2, smooth muscle, aorta | ACTA2 | 2.589 | 0.00373 |
| Hs.398156 | Similar to hypothetical protein FLJ21394, | --- | 2.589 | 0.01289 |
| Hs.483329 | KIAA1961 gene | KIAA1961 | 2.587 | 0.04166 |
| Hs.132696 | Transcribed locus | --- | 2.586 | 0.02723 |
| Hs.523724 | DKFZP434K028 protein | DKFZP434K028 | 2.583 | 0.00140 |
| Hs.518513 | tyrosine kinase, non-receptor, 2 | TNK2 | 2.583 | 0.04099 |
| Hs.545578 | Hypothetical LOC389295 | LOC153561 | 2.580 | 0.01044 |
| Hs.501423 | bruno-like 5, RNA binding protein (Drosophila) | BRUNOL5 | 2.579 | 0.04473 |
| Hs.372541 | kelch repeat and BTB (POZ) domain containing 2 | KBTBD2 | 2.577 | 0.00344 |
| Hs.115263 | epiregulin | EREG | 2.573 | 0.04296 |
| Hs.404175 | Homo sapiens, clone IMAGE:4836904, mRNA | --- | 2.570 | 0.01781 |
| Hs.334545 | Homo sapiens, clone IMAGE:3503939, mRNA | --- | 2.569 | 0.00044 |
| Hs.109438 | potassium channel tetramerisation domain containing 12 | KCTD12 | 2.564 | 0.00150 |
| Hs.314338 | WD repeat domain 9 | WDR9 | 2.563 | 0.04272 |
| Hs.503438 | Hypothetical protein FLJ37874 | FLJ37874 | 2.562 | 0.01181 |
| Hs.466550 | LOC440523 | LOC440523 | 2.559 | 0.03993 |
| Hs.134289 | ubiquitin specific protease 51 | USP51 | 2.559 | 0.00314 |
| Hs.476358 | Calcium channel, voltage-dependent, L type, alpha 1D subunit | CACNA1D | 2.556 | 0.00072 |
| Hs.525115 | DEAD (Asp-Glu-Ala-Asp) box polypeptide 10 | DDX10 | 2.555 | 0.00364 |
| Hs.513195 | proto-oncogene 8 | HCC-8 | 2.549 | 0.02775 |
| Hs.437451 | phospholipase A1 member A | PLA1A | 2.547 | 0.00500 |
| Hs.157859 | Homo sapiens, clone IMAGE:5259419, mRNA | --- | 2.546 | 0.04687 |
| Hs.27018 | RAS-like, family 12 | RASL12 | 2.545 | 0.02474 |
| Hs.128938 | similar to lipoxygenase homology domains 1 | LOC401460 | 2.543 | 0.00088 |
| Hs.519694 | chromosome 5 open reading frame 4 | C5orf4 | 2.540 | 0.03395 |
| Hs.519514 | tripartite motif-containing 36 | TRIM36 | 2.539 | 0.03492 |
| Hs.480190 | Myeloid/lymphoid or mixed-lineage leukemia translocated to, 2 | MLLT2 | 2.539 | 0.00208 |
| Hs.535229 | zinc finger and BTB domain containing 37 | ZBTB37 | 2.539 | 0.00889 |
| Hs.551210 | chromosome 14 open reading frame 56 | C14orf56 | 2.536 | 0.00961 |
| Hs.509707 | hypothetical protein FLJ33387 | FLJ33387 | 2.534 | 0.00160 |
| Hs.249718 | eukaryotic translation initiation factor 4E | EIF4E | 2.532 | 0.03386 |
| Hs.370885 | transmembrane protease, serine 6 | TMPRSS6 | 2.531 | 0.04384 |
| Hs.370510 | Immunoglobulin superfamily, member 4 | IGSF4 | 2.531 | 0.00422 |
| Hs.8261 | SPRY domain-containing SOCS box protein SSB-1 | SSB1 | 2.531 | 0.02495 |
| Hs.166313 | regulator of G-protein signalling 17 | RGS17 | 2.529 | 0.03430 |
| Hs.552900 | CDNA clone IMAGE:5186324, partial cds | --- | 2.528 | 0.00138 |
| Hs.414795 | serine (or cysteine) proteinase inhibitor, clade E | SERPINE1 | 2.527 | 0.01683 |
| Hs.134830 | collagen, type VIII, alpha 1 | COL8A1 | 2.527 | 0.03647 |
| Hs.529892 | Sequestosome 1 | SQSTM1 | 2.527 | 0.02651 |
| Hs.426296 | chromosome 10 open reading frame 104 | C10orf104 | 2.526 | 0.02616 |
| Hs.15725 | Immediate early response 5 | IER5 | 2.526 | 0.00624 |
| Hs.125898 | GNAS complex locus | GNAS | 2.525 | 0.00495 |
| Hs.110571 | growth arrest and DNA-damage-inducible, beta | GADD45B | 2.524 | 0.00008 |
| Hs.124246 | Chromosome 10 open reading frame 119 | C10orf119 | 2.523 | 0.01129 |
| Hs.91546 | cytochrome P450, family 26, subfamily B, polypeptide 1 | CYP26B1 | 2.521 | 0.02873 |
| Hs.210043 | sphingosine-1-phosphate phosphotase 2 | SGPP2 | 2.520 | 0.01990 |
| Hs.262960 | transient receptor potential cation channel, subfamily C, mem 4 | TRPC4 | 2.520 | 0.00162 |
| Hs.514006 | hypothetical gene supported by BC040875 | LOC400456 | 2.515 | 0.03592 |
| Hs.522255 | G kinase anchoring protein 1 | GKAP1 | 2.513 | 0.02577 |
| Hs.206501 | hypothetical protein from clone 643 | LOC57228 | 2.509 | 0.00067 |
| Hs.129895 | T-box 3 (ulnar mammary syndrome) | TBX3 | 2.508 | 0.00677 |
| Hs.4221 | hypothetical protein DKFZp761H039 | DKFZp761H039 | 2.508 | 0.00265 |
| Hs.508835 | telomerase-associated protein 1 | TEP1 | 2.506 | 0.03216 |
| Hs.107740 | Kruppel-like factor 2 (lung) | KLF2 | 2.506 | 0.02680 |
| Hs.18564 | Hypothetical protein MGC40405 | MGC40405 | 2.506 | 0.03406 |
| Hs.196102 | RB1-inducible coiled-coil 1 | RB1CC1 | 2.500 | 0.01929 |
| Hs.158351 | galanin receptor 2 | GALR2 | 2.499 | 0.02416 |
| Hs.464391 | tubulin-specific chaperone d | TBCD | 2.499 | 0.00734 |
| Hs.503429 | Hypothetical LOC399933 | --- | 2.496 | 0.03997 |
| Hs.101302 | Collagen, type XII, alpha 1 | COL12A1 | 2.496 | 0.01252 |
| Hs.522019 | ADAMTS-like 1 | ADAMTSL1 | 2.495 | 0.02139 |
| Hs.474970 | rotavirus X protein associated with NSP3 | RoXaN | 2.494 | 0.01183 |
| Hs.143751 | matrix metalloproteinase 11 (stromelysin 3) | MMP11 | 2.492 | 0.03853 |
| Hs.134812 | Transcribed locus | --- | 2.491 | 0.04549 |
| Hs.387381 | myeloid/lymphoid or mixed-lineage leukemia 2 | MLL2 | 2.491 | 0.03024 |
| Hs.388613 | Neogenin homolog 1 (chicken) | NEO1 | 2.490 | 0.04586 |
| Hs.133512 | Zinc finger CCCH type, antiviral 1 | ZC3HAV1 | 2.490 | 0.00131 |
| Hs.334846 | poliovirus receptor-related 1 | PVRL1 | 2.489 | 0.02909 |
| Hs.293411 | adaptor-related protein complex 4, sigma 1 subunit | AP4S1 | 2.488 | 0.03194 |
| Hs.278857 | Heterogeneous nuclear ribonucleoprotein H2 (H') | HNRPH2 | 2.487 | 0.00836 |
| Hs.176976 | Transcribed locus | --- | 2.487 | 0.00768 |
| Hs.447377 | VENT-like homeobox 2 pseudogene 1 | VENTX2P1 | 2.486 | 0.01105 |
| Hs.525389 | ras homolog gene family, member J | RHOJ | 2.485 | 0.02347 |
| Hs.252387 | cadherin, EGF LAG seven-pass G-type receptor 1 | CELSR1 | 2.484 | 0.04870 |
| Hs.501898 | Murine retrovirus integration site 1 homolog | MRVI1 | 2.483 | 0.01258 |
| Hs.540124 | Homo sapiens, clone IMAGE:5303042, mRNA | --- | 2.483 | 0.01710 |
| Hs.196584 | transmembrane protein 7 | TMEM7 | 2.483 | 0.01325 |
| Hs.406856 | CDNA: FLJ21735 fis, clone COLF3350 | --- | 2.483 | 0.04565 |
| Hs.288568 | monoacylglycerol O-acyltransferase 2 | MOGAT2 | 2.482 | 0.03073 |
| Hs.278564 | PBX/knotted 1 homeobox 2 | PKNOX2 | 2.482 | 0.00084 |
| Hs.514814 | acyl-malonyl condensing enzyme 1-like 2 | AMAC1L2-1 | 2.480 | 0.00186 |
| Hs.36927 | Heat shock 105kDa/110kDa protein 1 | HSPH1 | 2.478 | 0.00745 |
| Hs.16370 | CDNA FLJ11652 fis, clone HEMBA1004461 | --- | 2.476 | 0.02478 |
| --- | hypothetical protein LOC152719 | LOC152719 | 2.474 | 0.02811 |
| Hs.162121 | coatomer protein complex, subunit alpha | COPA | 2.474 | 0.03105 |
| Hs.522969 | peptidyl arginine deiminase, type IV | PADI4 | 2.473 | 0.01710 |
| Hs.514146 | titin-cap (telethonin) | TCAP | 2.472 | 0.03487 |
| Hs.472847 | Chromosome 20 open reading frame 35 | C20orf35 | 2.472 | 0.02756 |
| Hs.164226 | thrombospondin 1 | THBS1 | 2.471 | 0.04727 |
| Hs.209402 | Sp7 transcription factor | SP7 | 2.470 | 0.01960 |
| Hs.548140 | MRNA; cDNA DKFZp686K1037 | --- | 2.470 | 0.00494 |
| Hs.535921 | Homo sapiens, clone IMAGE:5225645, mRNA | --- | 2.462 | 0.03442 |
| Hs.5920 | Glucosamine (UDP-N-acetyl)-2-epimerase/N-acetylmannosamine kinase | GNE | 2.461 | 0.04693 |
| Hs.317659 | developmental pluripotency associated 4 | DPPA4 | 2.461 | 0.02850 |
| Hs.125898 | GNAS complex locus | GNAS | 2.457 | 0.00030 |
| Hs.435976 | Bruno-like 4, RNA binding protein (Drosophila) | BRUNOL4 | 2.454 | 0.02237 |
| Hs.370254 | BCL2-antagonist of cell death | BAD | 2.454 | 0.02453 |
| Hs.444947 | tribbles homolog 1 (Drosophila) | TRIB1 | 2.453 | 0.00891 |
| Hs.220971 | FOS-like antigen 2 | FOSL2 | 2.453 | 0.00986 |
| Hs.80962 | neurotensin | NTS | 2.451 | 0.02306 |
| Hs.502756 | AHNAK nucleoprotein (desmoyokin) | MGC5395 | 2.451 | 0.02989 |
| Hs.43071 | Hepatoma-derived growth factor-related protein 2 | HDGF2 | 2.449 | 0.04743 |
| Hs.464585 | Ankyrin repeat domain 12 | ANKRD12 | 2.448 | 0.02012 |
| Hs.458285 | Hypothetical LOC401131 | --- | 2.444 | 0.04168 |
| Hs.18946 | Mitochondrial ribosomal protein S26 | MRPS26 | 2.442 | 0.02914 |
| Hs.152803 | Chromosome 10 open reading frame 108 | C10orf108 | 2.441 | 0.01776 |
| Hs.469561 | UDP-glucuronate decarboxylase 1 | UXS1 | 2.439 | 0.02080 |
| Hs.272242 | chromosome 20 open reading frame 38 | C20orf38 | 2.436 | 0.04888 |
| Hs.461117 | Syntrophin, beta 2 | SNTB2 | 2.436 | 0.02370 |
| Hs.65756 | regulator of G-protein signalling 11 | RGS11 | 2.433 | 0.00330 |
| Hs.326730 | Armadillo repeat gene deletes in velocardiofacial syndrome | ARVCF | 2.432 | 0.02934 |
| Hs.185774 | eyes absent homolog 3 (Drosophila) | EYA3 | 2.431 | 0.03755 |
| --- | hypothetical protein LOC255654 | LOC255654 | 2.430 | 0.02945 |
| Hs.319171 | nuclear factor of kappa light polypeptide gene enhancer in B-cells | NFKBIZ | 2.429 | 0.00683 |
| Hs.148685 | G protein-coupled receptor, family C, group 5, member B | GPRC5B | 2.429 | 0.00554 |
| Hs.448972 | leucine-rich repeats and immunoglobulin-like domains 2 | LRIG2 | 2.428 | 0.01181 |
| Hs.518773 | Ubiquitin-conjugating enzyme E2D 3 | UBE2D3 | 2.428 | 0.01743 |
| Hs.161640 | Tyrosine aminotransferase | TAT | 2.427 | 0.03288 |
| Hs.279562 | myelin transcription factor 1 | MYT1 | 2.423 | 0.00066 |
| Hs.193170 | PDZ domain containing, X chromosome | FLJ21687 | 2.423 | 0.02334 |
| Hs.509637 | pleckstrin homology domain containing, family G member 3 | PLEKHG3 | 2.422 | 0.00412 |
| Hs.210469 | engulfment and cell motility 2 | ELMO2 | 2.422 | 0.00809 |
| Hs.518438 | SRY (sex determining region Y)-box 2 | SOX2 | 2.420 | 0.01472 |
| Hs.503074 | transmembrane protein 16A | TMEM16A | 2.418 | 0.00336 |
| Hs.405514 | hypothetical protein LOC284058 | LOC284058 | 2.418 | 0.02277 |
| Hs.534509 | keratin associated protein 2-2 2.1B | KRTAP2-2-2.1B | 2.416 | 0.03573 |
| --- | hypothetical protein LOC219731 | LOC219731 | 2.416 | 0.00349 |
| Hs.486063 | APG5 autophagy 5-like (S. cerevisiae) | APG5L | 2.413 | 0.00609 |
| Hs.478067 | latexin | LXN | 2.412 | 0.00528 |
| Hs.326303 | hypothetical protein LOC338809 | LOC338809 | 2.412 | 0.00768 |
| Hs.154296 | tolloid-like 2 /// tolloid-like 2 | TLL2 | 2.410 | 0.02297 |
| Hs.424980 | 5-hydroxytryptamine (serotonin) receptor 2A | HTR2A | 2.410 | 0.04820 |
| Hs.36761 | HRAS-like suppressor | HRASLS | 2.409 | 0.01927 |
| Hs.527295 | ectonucleotide pyrophosphatase/phosphodiesterase 1 | ENPP1 | 2.405 | 0.00601 |
| Hs.154140 | ovary-specific acidic protein | OSAP | 2.404 | 0.00145 |
| Hs.525401 | Adenylate cyclase 6 | ADCY6 | 2.404 | 0.01716 |
| Hs.272011 | UDP-Gal:betaGlcNAc beta 1,4- galactosyltransferase, polypeptide 1 | B4GALT1 | 2.402 | 0.04571 |
| Hs.517373 | protoporphyrinogen oxidase | PPOX | 2.401 | 0.04267 |
| Hs.343244 | Adaptor-related protein complex 1, gamma 2 subunit | AP1G2 | 2.400 | 0.04541 |
| Hs.532469 | polyamine oxidase (exo-N4-amino) | PAOX | 2.397 | 0.04519 |
| Hs.118166 | Hypothetical protein MGC24039 | MGC24039 | 2.397 | 0.02504 |
| Hs.524526 | solute carrier family 26, member 10 | SLC26A10 | 2.396 | 0.01777 |
| Hs.446678 | Nuclear receptor coactivator 2 | NCOA2 | 2.394 | 0.02299 |
| Hs.272062 | protein tyrosine phosphatase, receptor type, F | PTPRF | 2.393 | 0.00736 |
| Hs.132890 | (clone 33) macronuclear mRNA. | --- | 2.392 | 0.03541 |
| Hs.172510 | hypothetical protein LOC128344 | LOC128344 | 2.391 | 0.01224 |
| Hs.493275 | tripartite motif-containing 31 | TRIM31 | 2.391 | 0.01081 |
| Hs.209614 | MARVEL domain containing 1 | MARVELD1 | 2.390 | 0.04574 |
| Hs.439153 | Polymerase (DNA directed), eta | POLH | 2.389 | 0.01157 |
| Hs.446315 | Chromosome 10 open reading frame 45 | C10orf45 | 2.385 | 0.02190 |
| Hs.289015 | glycerophosphodiester phosphodiesterase domain containing 3 | GDPD3 | 2.383 | 0.01733 |
| Hs.505289 | YY1 associated factor 2 | YAF2 | 2.383 | 0.02683 |
| Hs.435001 | Kruppel-like factor 10 | KLF10 | 2.378 | 0.01264 |
| Hs.518773 | Ubiquitin-conjugating enzyme E2D 3 | UBE2D3 | 2.377 | 0.01031 |
| Hs.269528 | Mak3 homolog (S. cerevisiae) | MAK3 | 2.377 | 0.00430 |
| Hs.535029 | Hypothetical gene supported by BC047708 | --- | 2.375 | 0.03360 |
| Hs.436319 | parvin, alpha | PARVA | 2.375 | 0.03911 |
| Hs.549158 | phospholipase C, epsilon 1 | PLCE1 | 2.372 | 0.00934 |
| Hs.478230 | phospholipase D1, phophatidylcholine-specific | PLD1 | 2.372 | 0.04404 |
| Hs.190440 | ADP-ribosylation factor-like 6 interacting protein 2 | ARL6IP2 | 2.371 | 0.04281 |
| Hs.194698 | Cyclin B2 | CCNB2 | 2.367 | 0.02373 |
| Hs.32950 | keratin, hair, acidic, 3B | KRTHA3B | 2.367 | 0.00341 |
| Hs.327631 | Retinol dehydrogenase 13 (all-trans and 9-cis) | RDH13 | 2.365 | 0.02545 |
| Hs.543085 | kruppel-like zinc finger factor X17 | LOC377064 | 2.361 | 0.02433 |
| Hs.333059 | CDNA clone IMAGE:5744875, partial cds | --- | 2.361 | 0.04023 |
| Hs.106688 | chromosome X open reading frame 1 | CXorf1 | 2.359 | 0.00834 |
| Hs.203830 | protocadherin beta 7 | PCDHB7 | 2.358 | 0.01400 |
| Hs.471034 | elastase 3A, pancreatic (protease E) | ELA3A | 2.357 | 0.04929 |
| Hs.408182 | collagen, type II, alpha 1 | COL2A1 | 2.357 | 0.00340 |
| Hs.497353 | mediator of RNA polymerase II transcription, subunit 6 homolog | MED6 | 2.356 | 0.00977 |
| Hs.16229 | Assoc. molecule with the SH3 domain of STAM (AMSH) like protein | AMSH-LP | 2.355 | 0.01265 |
| Hs.193418 | tumor necrosis factor receptor superfamily, member 9 | TNFRSF9 | 2.351 | 0.00503 |
| Hs.110571 | growth arrest and DNA-damage-inducible, beta | GADD45B | 2.350 | 0.00150 |
| Hs.480615 | synaptopodin 2 | SYNPO2 | 2.349 | 0.00042 |
| Hs.157259 | Signal-induced proliferation-associated 1 like 3 | SIPA1L3 | 2.348 | 0.03831 |
| Hs.540419 | Transcribed locus | --- | 2.348 | 0.02466 |
| Hs.171054 | cadherin 6, type 2, K-cadherin (fetal kidney) | CDH6 | 2.348 | 0.00616 |
| Hs.557 | adrenergic, alpha-1D-, receptor | ADRA1D | 2.346 | 0.03471 |
| Hs.529976 | hypothetical protein LOC202451 | LOC202451 | 2.345 | 0.00330 |
| Hs.237886 | death-associated protein kinase 2 | DAPK2 | 2.345 | 0.01201 |
| Hs.149585 | glutamate-ammonia ligasedomain containing 1 | GLULD1 | 2.344 | 0.01072 |
| Hs.301296 | CDNA: FLJ23131 fis, clone LNG08502 | --- | 2.338 | 0.02363 |
| Hs.509113 | Neuronal PAS domain protein 3 | NPAS3 | 2.336 | 0.04305 |
| Hs.481068 | Full length insert cDNA clone YQ50C11 | --- | 2.334 | 0.02653 |
| Hs.64125 | RIM binding protein 2 | KIAA0318 | 2.333 | 0.02856 |
| Hs.21249 | chromosome X open reading frame 32 | CXorf32 | 2.333 | 0.00016 |
| Hs.501048 | Zinc finger protein 397 | ZNF397 | 2.331 | 0.04686 |
| Hs.522019 | ADAMTS-like 1 | ADAMTSL1 | 2.331 | 0.03522 |
| Hs.114198 | Mitogen-activated protein kinase kinase 5 | MAP2K5 | 2.329 | 0.03932 |
| Hs.445725 | SKI-like | SKIL | 2.328 | 0.00023 |
| Hs.406013 | keratin 18 | KRT18 | 2.326 | 0.01964 |
| Hs.99743 | CDNA FLJ35270 fis, clone PROST2005630 | --- | 2.325 | 0.00120 |
| Hs.299119 | Transcribed locus | --- | 2.318 | 0.00841 |
| Hs.347991 | nuclear receptor subfamily 2, group F, member 2 | NR2F2 | 2.317 | 0.02387 |
| Hs.546448 | protocadherin 11 X-linked | PCDH11X/Y | 2.315 | 0.00062 |
| Hs.86507 | chromosome 20 open reading frame 166 | C20orf166 | 2.313 | 0.00089 |
| Hs.460260 | Aldo-keto reductase family 1, member C2 | AKR1C1 | 2.311 | 0.03854 |
| Hs.436667 | pregnancy upregulated non-ubiquitously expressed CaM kinase | PNCK | 2.310 | 0.00123 |
| Hs.434618 | cyclic nucleotide gated channel alpha 4 | CNGA4 | 2.310 | 0.00957 |
| Hs.493239 | Transcribed locus | --- | 2.310 | 0.04033 |
| Hs.250687 | transient receptor potential cation channel, subfamily C, 1 | TRPC1 | 2.309 | 0.02099 |
| Hs.473648 | phosphoribosylglycinamide formyltransferase, | GART | 2.305 | 0.01717 |
| Hs.432924 | Full length insert cDNA YI37C01 | --- | 2.304 | 0.00351 |
| Hs.509664 | FYVE, RhoGEF and PH domain containing 2 | FGD2 | 2.304 | 0.00280 |
| Hs.445725 | SKI-like | SKIL | 2.301 | 0.00340 |
| Hs.435949 | zinc finger protein, subfamily 1A, 1 (Ikaros) | ZNFN1A1 | 2.299 | 0.01066 |
| Hs.518994 | NMDA receptor regulated 1 | NARG1 | 2.298 | 0.01256 |
| Hs.350868 | Hypothetical protein BC004360 | LOC87769 | 2.298 | 0.03640 |
| Hs.489033 | ATP-binding cassette, sub-family B (MDR/TAP), member 1 | ABCB1 | 2.298 | 0.03169 |
| Hs.501522 | O-6-methylguanine-DNA methyltransferase | MGMT | 2.292 | 0.03238 |
| Hs.435615 | calcium-sensing receptor | CASR | 2.290 | 0.01866 |
| Hs.534340 | protein kinase, cAMP-dependent, regulatory, type I, beta | PRKAR1B | 2.290 | 0.00060 |
| Hs.406793 | Uncharacterized gastric protein ZG32P | --- | 2.290 | 0.03791 |
| Hs.45127 | chondroitin sulfate proteoglycan 5 (neuroglycan C) | CSPG5 | 2.288 | 0.00361 |
| Hs.535423 | CDNA FLJ32287 fis, clone PROST2000274 | --- | 2.288 | 0.04214 |
| Hs.429365 | Similar to KIAA0454 protein | --- | 2.287 | 0.03226 |
| Hs.280604 | Protein phosphatase 3 (formerly 2B), | PPP3R1 | 2.286 | 0.03485 |
| Hs.150595 | cytochrome P450, family 26, subfamily A, polypeptide 1 | CYP26A1 | 2.286 | 0.03899 |
| Hs.262643 | Transcribed locus | --- | 2.285 | 0.00127 |
| Hs.193226 | UDP-glucose ceramide glucosyltransferase-like 2 | UGCGL2 | 2.283 | 0.04821 |
| Hs.137562 | Chromosome 18 open reading frame 45 | C18orf45 | 2.282 | 0.03765 |
| Hs.142908 | transcription factor Dp family, member 3 | TFDP3 | 2.282 | 0.02063 |
| Hs.434515 | CDNA FLJ40979 fis, clone UTERU2014398 | --- | 2.281 | 0.00748 |
| Hs.448589 | ankyrin repeat domain 1 (cardiac muscle) | ANKRD1 | 2.281 | 0.03711 |
| Hs.391480 | Hypothetical protein PRO2949 | PRO2949 | 2.280 | 0.03947 |
| Hs.544236 | Homo sapiens, clone IMAGE:4040324, mRNA | --- | 2.278 | 0.02324 |
| Hs.125715 | Muscleblind-like 2 (Drosophila) | MBNL2 | 2.277 | 0.00454 |
| Hs.513053 | DnaJ (Hsp40) homolog, subfamily A, member 4 | DNAJA4 | 2.277 | 0.03589 |
| Hs.180919 | inhibitor of DNA binding 2 | ID2 | 2.273 | 0.00700 |
| Hs.143314 | Similar to RIKEN cDNA 5430419M09 | --- | 2.273 | 0.04356 |
| Hs.494895 | AT-hook transcription factor | AKNA | 2.272 | 0.03207 |
| Hs.527973 | suppressor of cytokine signaling 3 | SOCS3 | 2.271 | 0.02198 |
| Hs.9613 | angiopoietin-like 4 | ANGPTL4 | 2.271 | 0.01544 |
| Hs.75182 | mannose receptor, C type 1 | MRC1/L1 | 2.269 | 0.04052 |
| Hs.474797 | CDC42 effector protein (Rho GTPase binding) 1 | CDC42EP1 | 2.269 | 0.03988 |
| Hs.147229 | hypothetical protein LOC145694 | LOC145694 | 2.268 | 0.03584 |
| Hs.490330 | natriuretic peptide receptor A/guanylate cyclase A | NPR1 | 2.267 | 0.00118 |
| Hs.143754 | Homo sapiens, clone IMAGE:5744974, mRNA | --- | 2.266 | 0.00458 |
| Hs.534575 | Hypothetical protein MGC2198 | MGC2198 | 2.263 | 0.01828 |
| Hs.253517 | similar to CHIA protein | LOC149620 | 2.262 | 0.03222 |
| Hs.179986 | flotillin 1 | FLOT1 | 2.259 | 0.01455 |
| Hs.1454 | gastric inhibitory polypeptide | GIP | 2.259 | 0.00640 |
| Hs.98321 | chromosome X open reading frame 36 | CXorf36 | 2.258 | 0.01302 |
| Hs.23187 | Transcribed locus | --- | 2.258 | 0.02677 |
| Hs.148819 | Syntrophin, gamma 2 | SNTG2 | 2.257 | 0.02298 |
| Hs.487046 | superoxide dismutase 2, mitochondrial | SOD2 | 2.254 | 0.04699 |
| Hs.60473 | chromosome 7 open reading frame 9 | C7orf9 | 2.254 | 0.02461 |
| Hs.127830 | Hypothetical protein LOC340156 | LOC340156 | 2.253 | 0.03054 |
| Hs.110571 | growth arrest and DNA-damage-inducible, beta | GADD45B | 2.251 | 0.00049 |
| Hs.130239 | chromosome 6 open reading frame 97 | C6orf97 | 2.250 | 0.04823 |
| Hs.189242 | MRNA; cDNA DKFZp434A202 | --- | 2.250 | 0.00584 |
| Hs.150556 | Hypothetical protein FLJ43663 | FLJ43663 | 2.250 | 0.02387 |
| Hs.156178 | Plasma glutamate carboxypeptidase | PGCP | 2.246 | 0.04354 |
| Hs.156471 | Transcription factor CP2-like 1 | TFCP2L1 | 2.245 | 0.04359 |
| Hs.372009 | Homo sapiens, clone IMAGE:5194204, mRNA | --- | 2.245 | 0.02673 |
| Hs.200644 | Hypothetical protein FLJ39609 | FLJ39609 | 2.245 | 0.02403 |
| Hs.445835 | SERTA domain containing 4 | SERTAD4 | 2.244 | 0.03455 |
| Hs.106311 | Similar to POM121 membrane glycoprotein-like 1 | DGCR9 | 2.244 | 0.02561 |
| Hs.112968 | forkhead box E3 | FOXE3 | 2.243 | 0.00216 |
| Hs.46332 | G protein-coupled receptor 6 | GPR6 | 2.243 | 0.00056 |
| Hs.512744 | hypothetical protein FLJ12056 | FLJ12056 | 2.240 | 0.04865 |
| Hs.349150 | Purine-rich element binding protein B | PURB | 2.240 | 0.04249 |
| Hs.283092 | hypothetical protein DKFZp547H025 | DKFZp547H025 | 2.238 | 0.02070 |
| Hs.190284 | Smith-Magenis syndrome chromosome region, candidate 6 | SREBF1 | 2.236 | 0.04664 |
| Hs.385677 | CDNA clone IMAGE:5164114, partial cds | --- | 2.234 | 0.03514 |
| Hs.370055 | Chromosome 6 open reading frame 117 | C6orf117 | 2.234 | 0.01880 |
| Hs.495674 | chloride channel 4 | CLCN4 | 2.233 | 0.03792 |
| Hs.129895 | T-box 3 (ulnar mammary syndrome) | TBX3 | 2.230 | 0.01091 |
| Hs.188401 | annexin A10 | ANXA10 | 2.228 | 0.00906 |
| Hs.533779 | claudin 6 | CLDN6 | 2.228 | 0.03632 |
| Hs.443481 | Hypothetical protein BC014022 | LOC116143 | 2.226 | 0.00618 |
| Hs.13261 | Brain-specific angiogenesis inhibitor 3 | BAI3 | 2.226 | 0.03765 |
| Hs.436625 | hypothetical protein FLJ25393 | FLJ25393 | 2.225 | 0.03775 |
| Hs.127249 | EAP30 subunit of ELL complex | EAP30 | 2.224 | 0.00770 |
| Hs.97579 | Transcribed locus | --- | 2.221 | 0.02869 |
| Hs.145675 | Sortilin-related VPS10 domain containing receptor 1 | SORCS1 | 2.219 | 0.01188 |
| Hs.415342 | KIAA1049 protein | KIAA1049 | 2.219 | 0.02930 |
| Hs.468736 | hypothetical protein FLJ32206 | FLJ32206 | 2.217 | 0.03960 |
| Hs.1219 | Alcohol dehydrogenase 4 (class II), pi polypeptide | ADH4 | 2.216 | 0.02496 |
| Hs.386791 | Phosphodiesterase 3A, cGMP-inhibited | PDE3A | 2.214 | 0.04543 |
| Hs.515860 | microtubule-associated protein, RP/EB family, member 3 | MAPRE3 | 2.214 | 0.01564 |
| --- | PRO1880 protein | PRO1880 | 2.214 | 0.02824 |
| Hs.28199 | Transcribed locus | --- | 2.211 | 0.01655 |
| Hs.403917 | FERM, RhoGEF (ARHGEF) and pleckstrin domain protein 1 | FARP1 | 2.209 | 0.03840 |
| Hs.472838 | serine/threonine kinase 4 | STK4 | 2.208 | 0.00116 |
| Hs.447458 | chromosome 10 open reading frame 6 | C10orf6 | 2.208 | 0.04560 |
| Hs.549128 | rab3 GTPase-activating protein, non-catalytic subunit (150kD) | RAB3-GAP150 | 2.207 | 0.03819 |
| Hs.434124 | hypothetical protein LOC149086 | LOC149086 | 2.206 | 0.01755 |
| Hs.435215 | vascular endothelial growth factor C | VEGFC | 2.206 | 0.04651 |
| Hs.157284 | Syntrophin, gamma 1 | SNTG1 | 2.203 | 0.04498 |
| Hs.403187 | Hypothetical protein LOC90529 | LOC90529 | 2.203 | 0.01259 |
| Hs.464205 | CDNA FLJ12232 fis, clone MAMMA1001206 | --- | 2.201 | 0.00608 |
| Hs.429365 | Similar to KIAA0454 protein | --- | 2.201 | 0.02893 |
| Hs.443150 | estrogen receptor 2 (ER beta) | ESR2 | 2.196 | 0.00961 |
| Hs.12292 | Transcribed locus | --- | 2.196 | 0.02217 |
| Hs.287523 | CDNA FLJ12367 fis, clone MAMMA1002413 | --- | 2.195 | 0.04611 |
| Hs.41735 | Purinergic receptor P2X, ligand-gated ion channel, 1 | P2RX1 | 2.192 | 0.04305 |
| Hs.293560 | LOC440460 | SH3GLP3 | 2.191 | 0.00246 |
| Hs.503510 | embryonic ectoderm development | EED | 2.189 | 0.00459 |
| Hs.485489 | Chloride intracellular channel 5 | CLIC5 | 2.189 | 0.00084 |
| Hs.383008 | hypothetical protein LOC339834 | LOC339834 | 2.188 | 0.01735 |
| Hs.87191 | Fibroblast growth factor 18 | FGF18 | 2.188 | 0.00134 |
| Hs.269127 | Hypothetical protein BC017868 | LOC159091 | 2.188 | 0.01011 |
| Hs.524278 | guanylate cyclase 2C | GUCY2C | 2.187 | 0.02217 |
| Hs.160411 | thyroid stimulating hormone receptor | TSHR | 2.187 | 0.01058 |
| Hs.129702 | beaded filament structural protein 1, filensin | BFSP1 | 2.186 | 0.04547 |
| Hs.298227 | chromosome 14 open reading frame 174 | C14orf174 | 2.185 | 0.04613 |
| Hs.232021 | REV3-like, catalytic subunit of DNA polymerase zeta | REV3L | 2.185 | 0.04819 |
| Hs.258855 | myeloid/lymphoid or mixed-lineage leukemia | MLL | 2.182 | 0.04502 |
| Hs.205098 | Transcribed locus | --- | 2.180 | 0.02770 |
| Hs.522109 | solute carrier family 6, member 14 | SLC6A14 | 2.179 | 0.03470 |
| Hs.149095 | solute carrier family 7, member 13 | SLC7A13 | 2.179 | 0.00275 |
| Hs.125715 | Muscleblind-like 2 (Drosophila) | MBNL2 | 2.177 | 0.03081 |
| Hs.371903 | glycophorin E | GYPE | 2.169 | 0.01253 |
| Hs.298079 | kinesin light chain 2-like | KLC2L | 2.168 | 0.01968 |
| Hs.154652 | hypothetical protein MGC39325 | MGC39325 | 2.167 | 0.02069 |
| Hs.22287 | Transcribed locus | --- | 2.162 | 0.04380 |
| Hs.493037 | potassium channel, subfamily K, member 9 | KCNK9 | 2.162 | 0.03637 |
| Hs.98523 | FAT tumor suppressor homolog 3 (Drosophila) | FAT3 | 2.161 | 0.03067 |
| Hs.126688 | choline dehydrogenase | CHDH | 2.160 | 0.03111 |
| Hs.471393 | solute carrier family 11 member 1 | SLC11A1 | 2.159 | 0.00089 |
| Hs.4859 | Cyclin L1 | CCNL1 | 2.159 | 0.00131 |
| Hs.525529 | K+ large conductance calcium-activated channel, subfamily M, beta member 4 | KCNMB4 | 2.158 | 0.03951 |
| Hs.532403 | hypothetical protein LOC284578 | LOC284578 | 2.157 | 0.03787 |
| Hs.272848 | hypothetical protein FLJ21019 | FLJ21019 | 2.155 | 0.01643 |
| Hs.172928 | collagen, type I, alpha 1 | COL1A1 | 2.155 | 0.00040 |
| Hs.549274 | hypothetical protein LOC201191 | LOC201191 | 2.152 | 0.04376 |
| Hs.302634 | frizzled homolog 8 | FZD8 | 2.151 | 0.04209 |
| Hs.1211 | Acid phosphatase 5, tartrate resistant | ACP5 | 2.147 | 0.00155 |
| Hs.513645 | P21(CDKN1A)-activated kinase 6 | PAK6 | 2.146 | 0.04210 |
| Hs.175955 | Splicing factor YT521-B | YT521 | 2.146 | 0.01863 |
| Hs.61812 | Protein tyrosine phosphatase, non-receptor type 12 | PTPN12 | 2.146 | 0.03137 |
| Hs.98661 | hypothetical LOC401022 | LOC401022 | 2.144 | 0.01699 |
| Hs.132340 | Chromosome 6 open reading frame 85 | C6orf85 | 2.144 | 0.01203 |
| Hs.549034 | Fibroblast growth factor receptor 1 ( | FGFR1 | 2.142 | 0.00163 |
| Hs.533994 | T cell receptor beta constant 1 | TRBC1 | 2.140 | 0.02421 |
| Hs.435742 | hypothetical protein FLJ25439 | FLJ25439 | 2.139 | 0.00826 |
| Hs.127383 | Hypothetical gene supported by AK129597 | --- | 2.138 | 0.01798 |
| Hs.268698 | methylenetetrahydrofolate dehydrogenase (NADP+ dependent) 1 | MTHFD1L | 2.137 | 0.00736 |
| Hs.512661 | KIAA1160 protein | KIAA1160 | 2.137 | 0.03092 |
| Hs.288284 | PQ loop repeat containing 1 | PQLC1 | 2.137 | 0.01403 |
| Hs.49050 | tigger transposable element derived 3 | TIGD3 | 2.137 | 0.02782 |
| Hs.255664 | Cytoplasmic linker 2 | CYLN2 | 2.135 | 0.01276 |
| Hs.531614 | BTB (POZ) domain containing 14B | BTBD14B | 2.133 | 0.03125 |
| Hs.65735 | Hypothetical protein LOC90835 | LOC90835 | 2.133 | 0.00035 |
| Hs.382152 | Homo sapiens, clone IMAGE:5111803, mRNA | --- | 2.132 | 0.00287 |
| Hs.511397 | melanoma cell adhesion molecule | MCAM | 2.132 | 0.02761 |
| Hs.112728 | Protein phosphatase 1F | PPM1F | 2.129 | 0.04954 |
| Hs.522863 | chromosome Y open reading frame 15A | CYorf15A | 2.129 | 0.00760 |
| Hs.472737 | Topoisomerase (DNA) I | TOP1 | 2.127 | 0.04361 |
| Hs.520189 | ELOVL family member 5, elongation of long chain fatty acids | ELOVL5 | 2.127 | 0.00692 |
| Hs.125293 | RasGEF domain family, member 1A | RASGEF1A | 2.127 | 0.01464 |
| Hs.307052 | lactate dehydrogenase A-like 6B | LDHAL6B | 2.121 | 0.04184 |
| Hs.122121 | Ring finger protein 12 | RNF12 | 2.121 | 0.04007 |
| Hs.19156 | Dishevelled associated activator of morphogenesis 1 | DAAM1 | 2.121 | 0.03596 |
| Hs.481986 | Similar to Interferon-induced guanylate-binding protein 1 | LOC400759 | 2.121 | 0.02735 |
| Hs.213424 | secreted frizzled-related protein 1 | SFRP1 | 2.120 | 0.01766 |
| Hs.82045 | Midkine (neurite growth-promoting factor 2) | MDK | 2.119 | 0.00156 |
| Hs.270525 | LAG1 longevity assurance homolog 5 | LASS5 | 2.118 | 0.03789 |
| Hs.194301 | microtubule-associated protein 1A | MAP1A | 2.117 | 0.00718 |
| Hs.514554 | KIAA1618 | KIAA1618 | 2.116 | 0.02855 |
| Hs.456065 | Homo sapiens, clone IMAGE:4822139, mRNA | --- | 2.113 | 0.04746 |
| Hs.301904 | Hypothetical protein FLJ12671 | FLJ12671 | 2.113 | 0.00230 |
| Hs.124953 | Cut-like 2 (Drosophila) | CUTL2 | 2.113 | 0.04775 |
| Hs.248149 | neurogenin 1 | NEUROG1 | 2.112 | 0.04641 |
| Hs.549626 | hypothetical protein MGC2848 | MGC2848 | 2.112 | 0.04260 |
| Hs.434953 | high-mobility group box 2 | HMGB2 | 2.110 | 0.00826 |
| Hs.146978 | G protein-coupled receptor 64 | GPR64 | 2.109 | 0.00949 |
| Hs.528462 | PI-3-kinase-related kinase SMG-1-like | KIAA0220 | 2.108 | 0.00638 |
| Hs.534313 | early growth response 3 | EGR3 | 2.108 | 0.00454 |
| Hs.444362 | LIM domain containing preferred translocation partner in lipoma | LPP | 2.108 | 0.02274 |
| Hs.95162 | KCNQ1 overlapping transcript 1 | KCNQ1OT1 | 2.107 | 0.03986 |
| Hs.371240 | A kinase (PRKA) anchor protein (gravin) 12 | AKAP12 | 2.104 | 0.00683 |
| Hs.407619 | chromosome 10 open reading frame 44 | C10orf44 | 2.103 | 0.02091 |
| Hs.319171 | nuclear factor of kappa light polypeptide gene enhancer in B-cells | NFKBIZ | 2.103 | 0.00998 |
| Hs.421281 | wingless-type MMTV integration site family, member 8B | WNT8B | 2.102 | 0.03535 |
| Hs.326371 | Poliovirus receptor-related 2 | PVRL2 | 2.102 | 0.00059 |
| Hs.541692 | Homo sapiens, clone IMAGE:4093039, mRNA | --- | 2.102 | 0.01188 |
| Hs.37014 | carbonic anhydrase VII | CA7 | 2.101 | 0.04109 |
| Hs.177151 | Transcribed locus | --- | 2.098 | 0.04457 |
| Hs.79299 | lipoma HMGIC fusion partner-like 2 | LHFPL2 | 2.098 | 0.01676 |
| Hs.368808 | EH-domain containing 3 | EHD3 | 2.097 | 0.04478 |
| Hs.181867 | Clone TESTIS-814 mRNA sequence | --- | 2.097 | 0.01021 |
| Hs.193251 | cell division cycle 2-like 6 (CDK8-like) | CDC2L6 | 2.096 | 0.04647 |
| Hs.8102 | Ribosomal protein S20 | RPS20 | 2.095 | 0.01307 |
| Hs.519313 | ALL1 fused gene from 5q31 | AF5Q31 | 2.095 | 0.01887 |
| Hs.150276 | cytochrome P450, family 3, subfamily A, polypeptide 5 | CYP3A5 | 2.094 | 0.03797 |
| Hs.543647 | FGF-2 activity-associated protein 3 (GAFA3) | --- | 2.094 | 0.04357 |
| Hs.201340 | Semaphoring 3D | SEMA3D | 2.093 | 0.04234 |
| Hs.182625 | vesicle-associated membrane protein-associated protein B and C | VAPB | 2.093 | 0.02921 |
| --- | acetylserotonin O-methyltransferase | ASMT | 2.092 | 0.00369 |
| Hs.552946 | Homo sapiens, clone IMAGE:4045462, mRNA | --- | 2.091 | 0.02697 |
| Hs.445000 | Prostaglandin E receptor 3 (subtype EP3) | PTGER3 | 2.091 | 0.00118 |
| Hs.180946 | Hypothetical LOC388650 | LOC388650 | 2.090 | 0.03883 |
| Hs.118241 | Chaperone, ABC1 activity of bc1 complex like | CABC1 | 2.089 | 0.02819 |
| Hs.236557 | prostate/rectrum and colon protein no. 2 | PRAC2 | 2.087 | 0.01703 |
| Hs.26770 | fatty acid binding protein 7, brain | FABP7 | 2.086 | 0.04392 |
| Hs.385986 | ubiquitin-conjugating enzyme E2B | UBE2B | 2.085 | 0.00054 |
| Hs.390729 | V-erb-a erythroblastic leukemia viral oncogene homolog 4 | ERBB4 | 2.083 | 0.02984 |
| Hs.112577 | KIAA1853 protein | KIAA1853 | 2.082 | 0.00050 |
| Hs.488143 | biliverdin reductase A | BLVRA | 2.080 | 0.01534 |
| Hs.247700 | forkhead box P3 | FOXP3 | 2.080 | 0.04045 |
| Hs.421430 | Hypothetical protein LOC149478 | LOC149478 | 2.079 | 0.02868 |
| Hs.44402 | Hypothetical protein LOC283267 | CSTF3 | 2.079 | 0.04522 |
| Hs.463412 | Sarcoglycan, alpha | SGCA | 2.078 | 0.01226 |
| Hs.148178 | RAP1, GTPase activating protein 1 | RAP1GA1 | 2.074 | 0.02530 |
| Hs.269059 | Jumonji, AT rich interactive domain 2 | JARID2 | 2.073 | 0.03066 |
| Hs.549748 | CDNA FLJ13712 fis, clone PLACE2000394 | --- | 2.073 | 0.00674 |
| Hs.435052 | ATPase, aminophospholipid transporter (APLT), Class I, type 8A, 1 | ATP8A1 | 2.073 | 0.01078 |
| Hs.508848 | Heterogeneous nuclear ribonucleoprotein C (C1/C2) | HNRPC | 2.072 | 0.02397 |
| Hs.357589 | hypothetical protein FLJ22789, clone | --- | 2.071 | 0.01921 |
| Hs.406696 | prion protein 2 (dublet) | PRND | 2.071 | 0.02421 |
| Hs.471778 | CDNA FLJ13876 fis, clone THYRO1001401 | --- | 2.069 | 0.00087 |
| Hs.258314 | Brain and reproductive organ-expressed | BRE | 2.069 | 0.00729 |
| Hs.493096 | pre-B-cell leukemia transcription factor 1 | PBX1 | 2.069 | 0.01961 |
| Hs.445476 | Transcribed locus | --- | 2.065 | 0.04403 |
| Hs.19492 | protocadherin 8 | PCDH8 | 2.065 | 0.01527 |
| Hs.524491 | 3'-phosphoadenosine 5'-phosphosulfate synthase 2 | PAPSS2 | 2.065 | 0.00235 |
| Hs.388918 | reversion-inducing-cysteine-rich protein with kazal motifs | RECK | 2.065 | 0.00520 |
| Hs.37125 | neuropeptide Y receptor Y2 | NPY2R | 2.063 | 0.04521 |
| Hs.518802 | hypothetical gene supported by BC042042 | LOC440704 | 2.061 | 0.03001 |
| Hs.390567 | FYN oncogene related to SRC, FGR, YES | FYN | 2.060 | 0.04225 |
| Hs.410810 | RAN binding protein 17 | RANBP17 | 2.055 | 0.00492 |
| Hs.170838 | Transcribed locus | --- | 2.055 | 0.00646 |
| Hs.115903 | Mannosyl (alpha-1,6-)-glycoprotein beta-1,6-N-acetyl-glucosaminyltransferase | MGAT5 | 2.053 | 0.03449 |
| Hs.306992 | CDNA clone IMAGE:3139291, partial cds | --- | 2.052 | 0.00194 |
| Hs.380133 | Zinc fingers and homeoboxes 3 | ZHX3 | 2.051 | 0.02782 |
| Hs.406976 | hypothetical protein LOC283874 | LOC283874 | 2.050 | 0.01390 |
| Hs.211236 | Netrin G1 | NTNG1 | 2.049 | 0.01852 |
| Hs.529901 | X (inactive)-specific transcript | XIST | 2.048 | 0.00523 |
| --- | glycophorin B (includes Ss blood group) | GYPB | 2.048 | 0.01173 |
| Hs.1048 | KIT ligand | KITLG | 2.044 | 0.01288 |
| Hs.510324 | Quaking homolog, KH domain RNA binding | QKI | 2.041 | 0.00853 |
| --- | hypothetical protein LOC285986 | LOC285986 | 2.040 | 0.04065 |
| Hs.368315 | Testis specific, 14 | TSGA14 | 2.039 | 0.02281 |
| Hs.514402 | Hypothetical protein MGC10986 | MGC10986 | 2.037 | 0.04374 |
| Hs.385567 | Homo sapiens, clone IMAGE:4838320, mRNA | --- | 2.037 | 0.03147 |
| Hs.546451 | solute carrier family 4, Na2+ bicarbonate cotransporter, member 9 | SLC4A9 | 2.035 | 0.00080 |
| Hs.347841 | Homo sapiens, clone IMAGE:5263088, mRNA | --- | 2.034 | 0.03648 |
| Hs.513735 | CDNA FLJ46637 fis, clone TRACH3002293 | --- | 2.033 | 0.00470 |
| Hs.204575 | chromosome 21 open reading frame 5 | C21orf5 | 2.033 | 0.03424 |
| Hs.459183 | alpha-kinase 3 | ALPK3 | 2.032 | 0.03113 |
| Hs.474693 | Similar to circumsporozoite protein precursor - | --- | 2.032 | 0.03640 |
| Hs.311208 | hypothetical protein LOC146443 | LOC146443 | 2.029 | 0.03204 |
| Hs.505323 | Testis-specific serine kinase 2 | STK22B | 2.028 | 0.02725 |
| Hs.449575 | Ig lambda light chain variable region | --- | 2.028 | 0.04088 |
| Hs.517397 | hypothetical protein FLJ40542 | FLJ40542 | 2.027 | 0.03377 |
| --- | hypothetical protein FLJ30672 | FLJ30672 | 2.025 | 0.02560 |
| Hs.129 | cholecystokinin A receptor | CCKAR | 2.024 | 0.03328 |
| Hs.398636 | hemoglobin, alpha 2 | HBA2 | 2.023 | 0.01550 |
| Hs.481022 | secreted frizzled-related protein 2 | SFRP2 | 2.021 | 0.02401 |
| Hs.513856 | Candidate tumor suppressor in ovarian cancer 2 | DPH2L1 | 2.019 | 0.01899 |
| Hs.24684 | arrestin domain containing 3 | ARRDC3 | 2.019 | 0.02336 |
| Hs.546543 | RAR-related orphan receptor C | RORC | 2.018 | 0.04082 |
| Hs.533391 | testis derived transcript (3 LIM domains) | TES | 2.018 | 0.00536 |
| Hs.435071 | Transcribed locus | --- | 2.017 | 0.04401 |
| Hs.520341 | 5'-nucleotidase, cytosolic II-like 1 | NT5C2L1 | 2.016 | 0.04848 |
| Hs.419763 | zinc finger protein 208 | ZNF208 | 2.016 | 0.04950 |
| Hs.90572 | PTK7 protein tyrosine kinase 7 | PTK7 | 2.016 | 0.04577 |
| Hs.226483 | Diaphanous homolog 2 (Drosophila) | DIAPH2 | 2.015 | 0.03190 |
| Hs.491767 | V-yes-1 Yamaguchi sarcoma viral related oncogene homolog | LYN | 2.008 | 0.01940 |
| Hs.476284 | dedicator of cytokinesis 3 | DOCK3 | 2.008 | 0.02573 |
| Hs.148778 | Oxidation resistance 1 | OXR1 | 2.005 | 0.01038 |
| Hs.445497 | heterogeneous nuclear ribonucleoprotein L-like | HNRPLL | 2.002 | 0.00807 |
| Hs.507971 | Leucine-rich repeats and calponin homology (CH) domain 1 | LRCH1 | 2.001 | 0.01025 |
| Hs.370950 | AXIN1 up-regulated 1 | AXUD1 | 2.000 | 0.02340 |
|  |  |  |  |  |
| Hs.532504 | G protein-coupled receptor 132 | GPR132 | -2.019 | 0.01157 |
| Hs.535413 | Rheumatoid arthritis synovium immunoglobulin heavy chain | --- | -2.023 | 0.00321 |
| Hs.414489 | K+ voltage-gated channel, delayed-rectifier, subfamily S, member 3 | KCNS3 | -2.028 | 0.03610 |
| Hs.170853 | Similar to MGC53446 protein | --- | -2.031 | 0.04444 |
| Hs.483944 | A disintegrin and metalloproteinase domain 19 | ADAM19 | -2.033 | 0.00325 |
| Hs.417549 | Protein tyrosine phosphatase, non-receptor type 1 | PTPN1 | -2.038 | 0.00958 |
| Hs.446689 | Similar to RIKEN cDNA 1110002C08 gene | MGC9564 | -2.041 | 0.01729 |
| Hs.291623 | TAO kinase 2 | TAOK2 | -2.041 | 0.00032 |
| Hs.433460 | Transcribed locus,] | --- | -2.052 | 0.03280 |
| Hs.408581 | similar to supervillin isoform 2; | LOC387648 | -2.057 | 0.01136 |
| Hs.368296 | MGC4707 protein | MGC4707 | -2.101 | 0.00950 |
| Hs.529948 | MDN1, midasin homolog (yeast) | MDN1 | -2.103 | 0.01554 |
| Hs.516954 | Rho guanine nucleotide exchange factor (GEF) 11 | ARHGEF11 | -2.104 | 0.01572 |
| Hs.233325 | hemochromatosis | HFE | -2.110 | 0.00695 |
| Hs.127897 | Rap guanine nucleotide exchange factor (GEF) 1 | RAPGEF1 | -2.118 | 0.01836 |
| Hs.380169 | similar to tRNA synthetase class II | DKFZp727A071 | -2.131 | 0.00460 |
| Hs.444172 | TNF receptor-associated factor 6 | TRAF6 | -2.133 | 0.00726 |
| Hs.197320 | Transducin-like enhancer of split 1 (E(sp1) | TLE1 | -2.140 | 0.00468 |
| Hs.192039 | protein tyrosine phosphatase, receptor type, C | PTPRC | -2.152 | 0.02933 |
| Hs.529544 | Transcribed locus | --- | -2.153 | 0.02050 |
| Hs.547593 | Homo sapiens, clone IMAGE:4551281, mRNA | --- | -2.159 | 0.04546 |
| Hs.200285 | Transcription factor 4 | TCF4 | -2.167 | 0.04849 |
| Hs.128279 | L(3)mbt-like 4 (Drosophila) | L3MBTL4 | -2.176 | 0.03686 |
| Hs.62661 | guanylate binding protein 1, interferon-inducible, 67kDa | GBP1 | -2.177 | 0.04566 |
| Hs.373857 | Kruppel-like factor 12 | KLF12 | -2.183 | 0.00287 |
| Hs.499620 | gem (nuclear organelle) associated protein 4 | GEMIN4 | -2.191 | 0.00044 |
| Hs.434248 | plectin 1, intermediate filament binding protein 500kDa | PLEC1 | -2.201 | 0.01426 |
| Hs.460934 | cancer/testis antigen CT45-2 | CT45-2 or 5 | -2.201 | 0.00829 |
| Hs.497301 | chromosome 14 open reading frame 150 | C14orf150 | -2.202 | 0.00217 |
| Hs.109620 | cysteine-rich secretory protein 1 | CRISP1 | -2.211 | 0.00715 |
| Hs.339453 | PTX1 protein | PTX1 | -2.215 | 0.03901 |
| Hs.347963 | chorionic somatomammotropin hormone 1 | CSH1 | -2.225 | 0.03248 |
| Hs.464829 | cadherin 2, type 1, N-cadherin (neuronal) | CDH2 | -2.237 | 0.02673 |
| Hs.517830 | biotinidase | BTD | -2.242 | 0.01233 |
| Hs.507348 | Heparan sulfate (glucosamine) 3-O-sulfotransferase 1 | HS3ST1 | -2.261 | 0.00044 |
| Hs.230188 | zinc finger protein 471 | ZNF471 | -2.275 | 0.00147 |
| Hs.522997 | unc-5 homolog B (C. elegans) | UNC5B | -2.281 | 0.01187 |
| Hs.423598 | pancreatic lipase-related protein 2 | PNLIPRP2 | -2.308 | 0.00032 |
| Hs.125706 | hypothetical protein LOC150527 | LOC150527 | -2.312 | 0.03367 |
| Hs.501535 | Similar to Cohesin subunit SA-3 | --- | -2.314 | 0.01656 |
| Hs.174743 | Similar to LOC166075 | LOC401097 | -2.324 | 0.00043 |
| Hs.532236 | hypothetical protein FLJ40722 | FLJ40722 | -2.333 | 0.01099 |
| Hs.503178 | Spectrin, beta, non-erythrocytic 1 | SPTBN1 | -2.334 | 0.00013 |
| Hs.434418 | myelin transcription factor 1-like | MYT1L | -2.351 | 0.00329 |
| Hs.458313 | DEAD (Asp-Glu-Ala-Asp) box polypeptide 28 | DDX28 | -2.368 | 0.01725 |
| Hs.545050 | CDNA: FLJ22466 fis, clone HRC10308 | --- | -2.372 | 0.01382 |
| Hs.129907 | Transcribed locus | --- | -2.413 | 0.01431 |
| Hs.84136 | paired-like homeodomain transcription factor 1 | PITX1 | -2.415 | 0.01483 |
| Hs.98244 | hypothetical protein MGC50721 | MGC50721 | -2.434 | 0.00226 |
| Hs.529680 | hypothetical protein FLJ25770 | FLJ25770 | -2.449 | 0.02938 |
| Hs.74050 | Follicular lymphoma variant translocation 1 | FVT1 | -2.460 | 0.01407 |
| --- | brain-derived neurotrophic factor opposite strand | BDNFOS | -2.460 | 0.00906 |
| Hs.446271 | Hypothetical gene supported by AK091718 | --- | -2.474 | 0.02404 |
| Hs.406557 | CDC-like kinase 4 | CLK4 | -2.488 | 0.03174 |
| Hs.258798 | chromosome 10 open reading frame 86 | C10orf86 | -2.524 | 0.01701 |
| Hs.437422 | erythrocyte membrane protein band 4.1-like 1 | EPB41L1 | -2.539 | 0.00311 |
| Hs.241546 | Clone 25074 mRNA sequence | --- | -2.540 | 0.03205 |
| Hs.13249 | CDNA FLJ30709 fis, clone FCBBF2001243 | --- | -2.541 | 0.00149 |
| Hs.490415 | Zyxin | ZYX | -2.547 | 0.01953 |
| Hs.311790 | CDNA FLJ11433 fis, clone HEMBA1001121 | --- | -2.552 | 0.03547 |
| Hs.382168 | nuclear receptor coactivator 3 | NCOA3 | -2.636 | 0.01007 |
| Hs.1048 | KIT ligand | KITLG | -2.646 | 0.00263 |
| Hs.515081 | dipeptidylpeptidase 9 | DPP9 | -2.658 | 0.00520 |
| Hs.547943 | CDNA FLJ23791 fis, clone HEP21711 | --- | -2.683 | 0.03514 |
| Hs.161254 | CDNA FLJ36285 fis, clone THYMU2003470 | --- | -2.704 | 0.02056 |
| Hs.23748 | LIM domain binding 2 | LDB2 | -2.704 | 0.00655 |
| Hs.33032 | Mediator of RNA polymerase II transcription, subunit 28 homolog | MED28 | -2.726 | 0.00413 |
| Hs.550862 | Transcribed locus | --- | -2.729 | 0.03956 |
| Hs.549092 | suppression of tumorigenicity 18 | ST18 | -2.804 | 0.00094 |
| Hs.355899 | tumor necrosis factor receptor superfamily, member 12A | TNFRSF12A | -2.810 | 0.00429 |
| Hs.516978 | Hypothetical protein LOC284804 | LOC284804 | -2.811 | 0.00708 |
| Hs.200260 | CDNA FLJ39181 fis, clone OCBBF2004235 | --- | -2.823 | 0.00053 |
| Hs.529618 | transferrin receptor (p90, CD71) | TFRC | -2.844 | 0.00701 |
| Hs.89626 | parathyroid hormone-like hormone | PTHLH | -2.850 | 0.00106 |
| Hs.549092 | Suppression of tumorigenicity 18 | ST18 | -2.870 | 0.00660 |
| Hs.401062 | NOL1/NOP2/Sun domain family, member 3 | NSUN3 | -2.883 | 0.00397 |
| Hs.132526 | melanoma antigen | LOC51152 | -2.902 | 0.00338 |
| Hs.515475 | symplekin | SYMPK | -2.913 | 0.00389 |
| Hs.349096 | hypothetical protein DKFZp313A2432 | DKFZp313A2432 | -2.978 | 0.00729 |
| Hs.409270 | THAP domain containing 9 | THAP9 | -3.017 | 0.00653 |
| --- | hypothetical protein LOC339929 | LOC339929 | -3.105 | 0.03286 |
| Hs.88630 | zinc finger protein 597 | ZNF597 | -3.139 | 0.02818 |
| Hs.207069 | Hypothetical LOC389362 | --- | -3.167 | 0.01056 |
| Hs.128166 | CDNA FLJ32992 fis, clone THYMU1000098 | --- | -3.215 | 0.00826 |
| Hs.784 | Epstein-Barr virus induced gene 2 | EBI2 | -3.247 | 0.00028 |
| Hs.519304 | pelota homolog (Drosophila) | PELO | -3.260 | 0.02096 |
| Hs.533291 | MRS2-like, magnesium homeostasis factor | MRS2L | -3.277 | 0.00698 |
| Hs.24115 | chromosome 13 open reading frame 25 | C13orf25 | -3.300 | 0.00008 |
| Hs.146040 | chromosome 14 open reading frame 105 | C14orf105 | -3.361 | 0.00075 |
| Hs.133183 | Hypothetical protein LOC284591 | LOC284591 | -3.455 | 0.00072 |
| Hs.369592 | thyroid adenoma associated | THADA | -3.469 | 0.01133 |
| Hs.416707 | ATP-binding cassette, sub-family A (ABC1), member 4 | ABCA4 | -3.487 | 0.00055 |
| Hs.406166 | hypothetical LOC400590 | LOC400590 | -3.747 | 0.00151 |
| Hs.38218 | Transcribed locus | --- | -4.881 | 0.01064 |
| Hs.250821 | zinc finger protein 557 | ZNF557 | -5.316 | 0.00000 |
