# Supplementary material for: Evaluation of the Genetic Response of U937 and Jurkat Cells to 10-Nanosecond Electrical Pulses (nsEP)
Source: PLoS One. 2016 May 2;11(5):e0154555. doi: 10.1371/journal.pone.0154555 (PMC4852903; doi:10.1371/journal.pone.0154555)
Supplement: S4 Table — Genes were selected based on log ratio (≥2, or ≤ -2) and with a p-value of ≤ 0.05. (DOCX) [file pone.0154555.s011.docx]

Supplementary Table 4: Complete list of significant genes changing in Jurkat exposed to 44°C for 40 min (heat shock, positive control). Genes were selected based on log ratio (≥2, or ≤ -2) and with a p-value of ≤ 0.05.

| **UniGene ID** | **Gene name** | **Symbol** | **Fold change 150kVnsEP vs. SHAM** | **p-Value**  **150kv nsEP**  **vs. SHAM** |
| --- | --- | --- | --- | --- |
| Hs.520028 | heat shock 70kDa protein 1A | HSPA1A | 9.558 | 2.2E-05 |
| Hs.25647 | v-fos FBJ murine osteosarcoma viral oncogene homolog | FOS | 9.196 | 2.3E-05 |
| Hs.3268 | heat shock 70kDa protein 6 (HSP70B') | HSPA6 | 8.766 | 1.4E-06 |
| Hs.3268 | heat shock 70kDa protein 6 (HSP70B') | HSPA6 | 8.251 | 5.2E-06 |
| Hs.169487 | v-maf musculoaponeurotic fibrosarcoma oncogene homolog B | MAFB | 7.337 | 2.0E-03 |
| Hs.326035 | Early growth response 1 | EGR1 | 7.098 | 2.3E-05 |
| Hs.1872 | phosphoenolpyruvate carboxykinase 1 (soluble) | PCK1 | 7.043 | 1.4E-05 |
| Hs.78944 | regulator of G-protein signalling 2, 24kDa | RGS2 | 6.866 | 2.7E-05 |
| Hs.75678 | FBJ murine osteosarcoma viral oncogene homolog B | FOSB | 6.565 | 1.3E-06 |
| Hs.520028 | heat shock 70kDa protein 1A /// heat shock 70kDa protein 1B | HSPA1A-1B | 6.443 | 4.5E-06 |
| Hs.326035 | early growth response 1 | EGR1 | 6.432 | 3.8E-05 |
| --- | ribonuclease P RNA component H1 | RPPH1 | 6.242 | 8.5E-05 |
| Hs.525704 | v-jun sarcoma virus 17 oncogene homolog (avian) | JUN | 6.216 | 3.7E-05 |
| Hs.137274 | Transcribed locus, | --- | 6.078 | 8.4E-04 |
| Hs.19492 | protocadherin 8 | PCDH8 | 6.000 | 3.4E-04 |
| Hs.408767 | crystallin, alpha B | CRYAB | 5.937 | 6.6E-04 |
| Hs.180919 | inhibitor of DNA binding 2, dominant negative helix-loop-helix | ID2 /// ID2B | 5.917 | 1.2E-05 |
| Hs.14706 | Clone DNA57836 GLPG464 (UNQ464) | --- | 5.875 | 1.3E-04 |
| Hs.80288 | heat shock 70kDa protein 1-like | HSPA1L | 5.748 | 2.7E-04 |
| Hs.326035 | early growth response 1 | EGR1 | 5.703 | 4.4E-07 |
| Hs.9701 | growth arrest and DNA-damage-inducible, gamma | GADD45G | 5.587 | 6.1E-05 |
| Hs.110571 | growth arrest and DNA-damage-inducible, beta | GADD45B | 5.459 | 1.6E-06 |
| Hs.180919 | inhibitor of DNA binding 2, dominant negative helix-loop-helix | ID2 | 5.420 | 8.5E-06 |
| Hs.241579 | serine (or cysteine) proteinase inhibitor, clade H | SERPINH1 | 5.410 | 4.2E-03 |
| Hs.468410 | Endothelial PAS domain protein 1 | EPAS1 | 5.395 | 2.7E-03 |
| Hs.525704 | v-jun sarcoma virus 17 oncogene homolog (avian) | JUN | 5.320 | 3.4E-06 |
| Hs.306327 | RAB3 GTPase-activating protein | RAB3GAP | 5.252 | 1.1E-02 |
| Hs.1395 | early growth response 2 | EGR2 | 5.229 | 6.9E-03 |
| Hs.549031 | early growth response 4 | EGR4 | 5.215 | 1.1E-03 |
| Hs.40888 | activity-regulated cytoskeleton-associated protein | ARC | 5.195 | 4.8E-04 |
| Hs.55468 | cDNA clone RZPDo834C0824D | --- | 5.183 | 8.1E-03 |
| Hs.48029 | snail homolog 1 (Drosophila) | SNAI1 | 5.151 | 7.0E-04 |
| Hs.547721 | CDNA FLJ12144 fis, clone MAMMA1000361 | --- | 5.112 | 3.4E-05 |
| Hs.25829 | RAS, dexamethasone-induced 1 | RASD1 | 5.040 | 1.0E-03 |
| Hs.57690 | crystallin, beta A4 | CRYBA4 | 5.035 | 4.1E-04 |
| Hs.200412 | epiplakin 1 | EPPK1 | 5.009 | 4.0E-04 |
| Hs.414795 | serine (or cysteine) proteinase inhibitor, clade E | SERPINE1 | 4.956 | 4.2E-04 |
| Hs.110571 | growth arrest and DNA-damage-inducible, beta | GADD45B | 4.941 | 2.3E-04 |
| Hs.182137 | histone 1, H2bg | HIST1H2BG | 4.928 | 4.6E-03 |
| Hs.110571 | growth arrest and DNA-damage-inducible, beta | GADD45B | 4.889 | 4.0E-05 |
| Hs.180919 | inhibitor of DNA binding 2, dominant negative helix-loop-helix | ID2 /// ID2B | 4.856 | 7.3E-05 |
| Hs.477070 | Casein kinase 1, delta | CSNK1D | 4.845 | 3.1E-03 |
| Hs.525704 | v-jun sarcoma virus 17 oncogene homolog (avian) | JUN | 4.828 | 9.9E-07 |
| Hs.379912 | delta-like 1 (Drosophila) | DLL1 | 4.792 | 1.0E-03 |
| Hs.163867 | CD14 antigen /// CD14 antigen | CD14 | 4.779 | 1.0E-03 |
| Hs.484738 | myosin regulatory light chain interacting protein | MYLIP | 4.732 | 9.7E-04 |
| Hs.171695 | dual specificity phosphatase 1 | DUSP1 | 4.708 | 2.4E-04 |
| Hs.413297 | regulator of G-protein signalling 16 | RGS16 | 4.695 | 1.0E-03 |
| Hs.113684 | heat shock 60kDa protein 1 (chaperonin) | HSPD1 | 4.654 | 4.7E-04 |
| Hs.376617 | MICAL-like 2 | MICAL-L2 | 4.603 | 5.9E-03 |
| Hs.296323 | serum/glucocorticoid regulated kinase | SGK | 4.552 | 1.8E-04 |
| Hs.353515 | hypothetical protein FLJ34047 | FLJ34047 | 4.549 | 8.3E-03 |
| Hs.509410 | Zinc finger protein 323 | ZNF323 | 4.500 | 2.8E-05 |
| Hs.220971 | FOS-like antigen 2 | FOSL2 | 4.476 | 1.5E-02 |
| Hs.459652 | Chromosome 16 open reading frame 30 | C16orf30 | 4.470 | 3.1E-04 |
| Hs.192233 | periplakin | PPL | 4.437 | 9.4E-05 |
| Hs.282735 | nuclear receptor subfamily 1, group H, member 4 | NR1H4 | 4.418 | 1.1E-04 |
| Hs.408182 | collagen, type II, alpha 1 | COL2A1 | 4.359 | 2.2E-06 |
| Hs.195403 | Dedicator of cytokinesis 5 | DOCK5 | 4.354 | 2.2E-04 |
| Hs.503074 | transmembrane protein 16A | TMEM16A | 4.312 | 9.0E-03 |
| Hs.208036 | transmembrane 7 superfamily member 4 | TM7SF4 | 4.282 | 3.0E-04 |
| Hs.8373 | FIS | FIS | 4.279 | 3.8E-04 |
| Hs.250666 | hairy and enhancer of split 1, (Drosophila) | HES1 | 4.279 | 1.9E-03 |
| Hs.89714 | chemokine (C-X-C motif) ligand 5 | CXCL5 | 4.273 | 1.3E-02 |
| Hs.406779 | TPTE and PTEN homologous inositol lipid phosphatase | LOC374491 | 4.216 | 2.6E-04 |
| Hs.435001 | Kruppel-like factor 10 | KLF10 | 4.198 | 1.9E-03 |
| Hs.441047 | adrenomedullin | ADM | 4.189 | 7.8E-05 |
| Hs.369554 | solute carrier family 16 (monocarboxylic acid transporters), member 5 | SLC16A5 | 4.179 | 3.1E-03 |
| Hs.522261 | Homo sapiens, clone IMAGE:4838261, mRNA | --- | 4.177 | 4.4E-03 |
| Hs.534313 | early growth response 3 | EGR3 | 4.163 | 3.9E-05 |
| Hs.529857 | immediate early response 5-like | IER5L | 4.156 | 2.2E-04 |
| Hs.541841 | Acyl-Coenzyme A dehydrogenase family, member 9 | ACAD9 | 4.144 | 6.2E-03 |
| Hs.546288 | Ribosomal protein S9 | RPS9 | 4.114 | 1.0E-03 |
| Hs.112895 | BTB (POZ) domain containing 14A | BTBD14A | 4.101 | 1.8E-03 |
| Hs.502876 | ras homolog gene family, member B | RHOB | 4.090 | 2.7E-04 |
| Hs.551774 | CDNA clone IMAGE:5297318, partial cds | --- | 4.089 | 3.3E-05 |
| Hs.34871 | zinc finger homeobox 1b | ZFHX1B | 4.079 | 1.2E-03 |
| Hs.356624 | nidogen (enactin) | NID | 4.065 | 7.9E-03 |
| Hs.121539 | sonic hedgehog homolog (Drosophila) | SHH | 4.053 | 1.8E-03 |
| Hs.386791 | Phosphodiesterase 3A, cGMP-inhibited | PDE3A | 4.041 | 2.9E-03 |
| Hs.518814 | chemokine (C-X-C motif) ligand 11 | CXCL11 | 4.035 | 1.7E-03 |
| Hs.134999 | histone 1, H2am | HIST1H2AM | 3.993 | 1.6E-04 |
| Hs.503510 | embryonic ectoderm development | EED | 3.978 | 2.1E-02 |
| Hs.533956 | ATPase, Ca++ transporting, plasma membrane 3 | ATP2B3 | 3.967 | 3.6E-03 |
| Hs.515210 | DnaJ (Hsp40) homolog, subfamily B, member 1 | DNAJB1 | 3.961 | 5.8E-05 |
| Hs.528111 | interleukin 27 | IL27 | 3.957 | 2.6E-03 |
| Hs.334688 | phytanoyl-CoA hydroxylase interacting protein | PHYHIP | 3.947 | 6.5E-04 |
| Hs.36927 | Heat shock 105kDa/110kDa protein 1 | HSPH1 | 3.907 | 4.6E-05 |
| Hs.169487 | v-maf musculoaponeurotic fibrosarcoma oncogene homolog B | MAFB | 3.905 | 2.4E-04 |
| Hs.501252 | Transforming, acidic coiled-coil containing protein 2 | TACC2 | 3.894 | 7.3E-03 |
| Hs.172928 | collagen, type I, alpha 1 | COL1A1 | 3.892 | 1.7E-03 |
| Hs.164070 | C-terminal modulator protein | CTMP | 3.889 | 2.4E-02 |
| Hs.484738 | myosin regulatory light chain interacting protein | MYLIP | 3.869 | 9.4E-06 |
| Hs.376206 | Kruppel-like factor 4 (gut) | KLF4 | 3.866 | 2.8E-05 |
| Hs.195048 | Centaurin, gamma 3 | CENTG3 | 3.855 | 6.0E-04 |
| Hs.174193 | zinc finger protein 335 | ZNF335 | 3.855 | 3.9E-04 |
| Hs.406691 | histone 1, H2aj | HIST1H2AJ | 3.834 | 2.6E-03 |
| Hs.76556 | protein phosphatase 1, regulatory (inhibitor) subunit 15A | PPP1R15A | 3.829 | 4.9E-04 |
| Hs.272398 | ets variant gene 7 (TEL2 oncogene) | ETV7 | 3.827 | 4.1E-02 |
| Hs.549257 | DnaJ (Hsp40) homolog, subfamily B, member 7 | DNAJB7 | 3.817 | 3.6E-02 |
| Hs.550529 | Caspase recruitment domain family, member 14 | CARD14 | 3.817 | 4.6E-03 |
| Hs.524838 | KIAA1906 protein | KIAA1906 | 3.796 | 6.6E-05 |
| Hs.448872 | SNAP25-interacting protein | SNIP | 3.793 | 4.2E-03 |
| Hs.377972 | Chromosome 13 open reading frame 21 | C13orf21 | 3.784 | 1.8E-03 |
| Hs.546863 | Homo sapiens, clone IMAGE:5170250, mRNA | --- | 3.763 | 7.4E-03 |
| Hs.72550 | Hyaluronan-mediated motility receptor (RHAMM) | HMMR | 3.746 | 4.2E-04 |
| Hs.73793 | vascular endothelial growth factor | VEGF | 3.737 | 6.7E-03 |
| Hs.382179 | CDNA clone IMAGE:4828683, partial cds | --- | 3.729 | 4.8E-02 |
| Hs.543963 | MRNA; cDNA DKFZp434L042 | --- | 3.722 | 5.9E-04 |
| Hs.463412 | Sarcoglycan, alpha | SGCA | 3.711 | 2.5E-04 |
| Hs.485071 | diffuse panbronchiolitis critical region 1 | DPCR1 | 3.709 | 7.6E-04 |
| Hs.484990 | Histone 1, H3j | HIST1H3J | 3.701 | 2.5E-03 |
| Hs.370041 | forkhead box J1 | FOXJ1 | 3.698 | 4.6E-05 |
| Hs.520525 | fibronectin type III domain containing 1 | FNDC1 | 3.696 | 3.1E-02 |
| Hs.47314 | Transcribed locus | --- | 3.694 | 2.1E-02 |
| Hs.118681 | v-erb-b2 erythroblastic leukemia viral oncogene homolog 3 | ERBB3 | 3.673 | 1.1E-02 |
| Hs.334545 | Homo sapiens, clone IMAGE:3503939, mRNA | --- | 3.644 | 8.4E-03 |
| Hs.128316 | BAI1-associated protein 2 | BAIAP2 | 3.642 | 2.8E-02 |
| Hs.480825 | Ring finger protein 150 | RNF150 | 3.638 | 2.8E-04 |
| Hs.133982 | Endoplasmic reticulum to nucleus signalling 1 | ERN1 | 3.635 | 1.9E-04 |
| Hs.480190 | Myeloid/lymphoid or mixed-lineage leukemia | MLLT2 | 3.635 | 5.6E-06 |
| Hs.408182 | collagen, type II, alpha 1 | COL2A1 | 3.626 | 3.1E-02 |
| Hs.97386 | hypothetical protein LOC253573 | LOC253573 | 3.625 | 9.5E-03 |
| Hs.274402 | heat shock 70kDa protein 1B | HSPA1B | 3.623 | 1.1E-06 |
| Hs.534370 | histone 2, H4 | HIST2H4 | 3.623 | 1.0E-02 |
| Hs.348434 | Homo sapiens, clone IMAGE:5286779, mRNA | --- | 3.607 | 5.7E-03 |
| Hs.375001 | Talin 1 | TLN1 | 3.606 | 4.7E-04 |
| Hs.529857 | immediate early response 5-like | IER5L | 3.588 | 6.6E-03 |
| Hs.247813 | histone 1, H3g | HIST1H3G | 3.579 | 3.2E-03 |
| Hs.172928 | collagen, type I, alpha 1 | COL1A1 | 3.578 | 1.4E-03 |
| Hs.372394 | hypothetical protein FLJ36144 | FLJ36144 | 3.578 | 5.3E-03 |
| Hs.536535 | dual specificity phosphatase 16 | DUSP16 | 3.554 | 4.2E-04 |
| Hs.501080 | Transcription factor 7-like 2 | TCF7L2 | 3.541 | 4.2E-03 |
| Hs.17958 | galactose-3-O-sulfotransferase 1 | GAL3ST1 | 3.491 | 1.4E-03 |
| Hs.61857 | Transcribed locus | --- | 3.490 | 3.2E-02 |
| Hs.468410 | Endothelial PAS domain protein 1 | EPAS1 | 3.489 | 1.9E-02 |
| Hs.532144 | histone 1, H3d | HIST1H3D | 3.489 | 1.2E-02 |
| Hs.380282 | DnaJ (Hsp40) homolog, subfamily B, member 4 | DNAJB4 | 3.487 | 1.0E-04 |
| Hs.120633 | Sestrin 3 | SESN3 | 3.483 | 1.3E-02 |
| Hs.250666 | hairy and enhancer of split 1, (Drosophila) | HES1 | 3.478 | 1.9E-03 |
| Hs.278908 | microrchidia homolog (mouse) | MORC | 3.477 | 1.9E-02 |
| Hs.552585 | Zinc finger, CW type with coiled-coil domain 1 | ZCWCC1 | 3.472 | 2.3E-03 |
| Hs.502328 | CD44 antigen | CD44 | 3.471 | 2.6E-03 |
| Hs.398636 | hemoglobin, alpha 2 | HBA2 | 3.465 | 1.9E-03 |
| Hs.8118 | KIAA0650 protein | KIAA0650 | 3.461 | 2.2E-03 |
| Hs.97849 | Transcribed locus | --- | 3.454 | 6.1E-03 |
| Hs.189810 | sulfotransferase family 4A, member 1 | SULT4A1 | 3.453 | 4.4E-05 |
| Hs.282410 | Calmodulin 1 (phosphorylase kinase, delta) | CALM1 | 3.450 | 1.2E-03 |
| Hs.374774 | ankyrin repeat domain 29 | ANKRD29 | 3.445 | 2.1E-02 |
| Hs.298863 | KPL2 protein | FLJ23577 | 3.445 | 6.7E-03 |
| Hs.160550 | Solute carrier family 43, member 2 | SLC43A2 | 3.439 | 1.1E-02 |
| Hs.7886 | Pellino homolog 1 (Drosophila) | PELI1 | 3.436 | 2.7E-02 |
| Hs.182137 | histone 1, H2bg | HIST1H2BG | 3.434 | 9.0E-04 |
| Hs.432453 | Mitogen-activated protein kinase kinase kinase 8 | MAP3K8 | 3.431 | 2.4E-02 |
| Hs.87191 | fibroblast growth factor 18 | FGF18 | 3.428 | 8.2E-03 |
| Hs.278906 | variable charge, X-linked | VCX | 3.427 | 5.1E-03 |
| Hs.213389 | Golgi autoantigen, golgin subfamily b, macrogolgin 1 | GOLGB1 | 3.412 | 1.2E-02 |
| Hs.321637 | semaphorin 5A | SEMA5A | 3.412 | 3.8E-03 |
| Hs.388304 | doublecortin and CaM kinase-like 3 | DCAMKL3 | 3.411 | 6.6E-04 |
| Hs.445725 | SKI-like | SKIL | 3.403 | 1.5E-04 |
| Hs.127126 | Cytoplasmic polyadenylation element binding protein 4 | CPEB4 | 3.402 | 4.3E-02 |
| Hs.545322 | CDNA clone IMAGE:5537695, partial cds | --- | 3.397 | 1.8E-03 |
| Hs.494023 | hypothetical gene | LOC401155 | 3.395 | 3.9E-03 |
| --- | hypothetical protein MGC12916 | MGC12916 | 3.393 | 2.8E-04 |
| Hs.48348 | hypothetical protein HH114 | HH114 | 3.393 | 3.4E-02 |
| Hs.98785 | Ksp37 protein | KSP37 | 3.393 | 1.4E-02 |
| Hs.288478 | Homo sapiens, clone IMAGE:4214313, mRNA | --- | 3.389 | 9.8E-04 |
| Hs.515217 | Solute carrier family 1, member 6 | SLC1A6 | 3.377 | 2.5E-02 |
| Hs.171695 | dual specificity phosphatase 1 | DUSP1 | 3.376 | 3.1E-05 |
| Hs.478588 | B-cell CLL/lymphoma 6 (zinc finger protein 51) | BCL6 | 3.373 | 1.7E-02 |
| Hs.483793 | hypothetical protein FLJ36748 | FLJ36748 | 3.370 | 1.5E-02 |
| Hs.288945 | Hypothetical protein FLJ13448 | FLJ13448 | 3.368 | 2.2E-02 |
| Hs.546315 | histone 1, H3a | HIST1H3A | 3.363 | 5.8E-03 |
| Hs.377660 | CDNA FLJ26242 fis, clone DMC00770 | --- | 3.362 | 2.4E-03 |
| Hs.533831 | Chromosome 13 open reading frame 22 | C13orf22 | 3.358 | 1.8E-04 |
| --- | similar to Argininosuccinate synthase | LOC402295 | 3.354 | 2.0E-03 |
| Hs.547272 | Skin-specific protein (xp32) | --- | 3.340 | 6.8E-03 |
| Hs.352298 | DNA-damage inducible protein 1 | PDGFD | 3.337 | 6.9E-03 |
| Hs.502314 | zinc finger-like | LOC400713 | 3.331 | 1.2E-02 |
| Hs.385677 | CDNA clone IMAGE:5164114, partial cds | --- | 3.328 | 1.5E-02 |
| Hs.249196 | distal-less homeo box 6 | DLX6 | 3.322 | 3.0E-03 |
| Hs.15725 | immediate early response 5 | IER5 | 3.318 | 1.9E-05 |
| Hs.234434 | hairy/enhancer-of-split related with YRPW motif 1 | HEY1 | 3.313 | 7.1E-04 |
| Hs.388622 | zinc finger protein, subfamily 1A, 4 (Eos) | ZNFN1A4 | 3.304 | 1.0E-03 |
| Hs.21435 | Transcribed locus | --- | 3.298 | 4.3E-02 |
| Hs.413801 | Proteasome (prosome, macropain) activator subunit 4 | PSME4 | 3.297 | 1.7E-02 |
| Hs.370699 | hypothetical protein LOC284801 | LOC284801 | 3.291 | 5.9E-05 |
| Hs.487536 | Hypothetical gene | --- | 3.291 | 6.6E-03 |
| Hs.28346 | glial cells missing homolog 1 (Drosophila) | GCM1 | 3.283 | 2.7E-02 |
| Hs.478589 | Hypothetical LOC389185 | --- | 3.281 | 4.3E-03 |
| Hs.87191 | Fibroblast growth factor 18 | FGF18 | 3.281 | 7.6E-03 |
| Hs.445725 | SKI-like | SKIL | 3.277 | 1.9E-02 |
| Hs.291993 | Transcribed locus | --- | 3.274 | 2.6E-04 |
| Hs.370699 | hypothetical protein LOC284801 | LOC284801 | 3.273 | 3.6E-05 |
| Hs.21081 | hypothetical protein FLJ13646 | FLJ13646 | 3.269 | 2.8E-04 |
| Hs.131956 | histone 1, H1b | HIST1H1B | 3.269 | 6.9E-03 |
| Hs.424980 | 5-hydroxytryptamine (serotonin) receptor 2A /// 5-hydroxytryptamine (serotonin) receptor 2A | HTR2A | 3.266 | 1.2E-03 |
| Hs.546743 | Homo sapiens, clone IMAGE:5272066, mRNA | --- | 3.265 | 1.4E-04 |
| Hs.121017 | histone 1, H2ae | HIST1H2AE | 3.264 | 3.4E-02 |
| Hs.476610 | CDNA FLJ12258 fis, clone MAMMA1001510 | --- | 3.255 | 1.5E-03 |
| Hs.298258 | MRNA; cDNA DKFZp313C0240 | --- | 3.252 | 2.9E-02 |
| Hs.434330 | Hypothetical gene supported by AK057431 | --- | 3.252 | 7.7E-03 |
| Hs.300304 | aristaless related homeobox | ARX | 3.244 | 2.4E-02 |
| Hs.12513 | hypothetical protein LOC157627 | LOC157627 | 3.238 | 2.2E-03 |
| Hs.162246 | proline-rich protein PRP2 | PRP2 | 3.233 | 8.8E-03 |
| Hs.499674 | mannose-binding lectin (protein C) 2, soluble | MBL2 | 3.232 | 2.1E-02 |
| Hs.118769 | Transcribed locus | --- | 3.218 | 1.8E-02 |
| --- | protocadherin gamma subfamily B, 4 8 | PCDHGB4-8 | 3.211 | 1.5E-02 |
| Hs.368431 | runt-related transcription factor 1; translocated to, 1 (cyclin D-related) | RUNX1T1 | 3.210 | 7.2E-03 |
| Hs.259432 | hypothetical protein LOC339168 | LOC339168 | 3.204 | 1.1E-03 |
| Hs.550815 | CDNA clone IMAGE:6602628, partial cds | --- | 3.200 | 3.9E-03 |
| Hs.407577 | Homo sapiens, clone IMAGE:5547271, mRNA | --- | 3.199 | 1.6E-04 |
| Hs.164226 | thrombospondin 1 | THBS1 | 3.199 | 4.3E-02 |
| Hs.57971 | hairy and enhancer of split 5 (Drosophila) | HES5 | 3.197 | 7.9E-03 |
| Hs.533292 | histone 1, H3b | HIST1H3B | 3.192 | 2.5E-04 |
| Hs.527697 | Homo sapiens, clone IMAGE:3604678, mRNA | --- | 3.191 | 9.4E-05 |
| Hs.27688 | Full-length cDNA clone CS0DF012YD09 of Fetal brain of Homo sapiens (human) | --- | 3.191 | 6.9E-03 |
| Hs.208124 | estrogen receptor 1 | ESR1 | 3.186 | 9.3E-03 |
| Hs.550529 | caspase recruitment domain family, member 14 | CARD14 | 3.183 | 2.5E-02 |
| Hs.513260 | DDHD domain containing 1 | DDHD1 | 3.179 | 3.2E-02 |
| --- | --- | --- | 3.161 | 1.0E-02 |
| Hs.75182 | mannose receptor, C type 1 /// mannose receptor, C type 1-like 1 | MRC1 /// MRC1L1 | 3.148 | 3.1E-02 |
| Hs.551265 | CDNA FLJ39495 fis, clone PROST2016499 | --- | 3.144 | 4.5E-02 |
| Hs.511723 | T-cell receptor beta chain (TCRBV10S1J2S gene) | --- | 3.143 | 2.2E-03 |
| Hs.515210 | DnaJ (Hsp40) homolog, subfamily B, member 1 | DNAJB1 | 3.140 | 3.1E-06 |
| Hs.515642 | Glycoprotein, synaptic 2 | GPSN2 | 3.139 | 4.9E-02 |
| Hs.442703 | Hypothetical LOC200159 | --- | 3.139 | 1.6E-03 |
| Hs.434948 | TGF-betaIIR beta | --- | 3.137 | 1.3E-02 |
| --- | --- | --- | 3.134 | 1.7E-04 |
| Hs.4859 | Cyclin L1 | CCNL1 | 3.130 | 9.2E-05 |
| Hs.283011 | a disintegrin and metalloproteinase domain 30 | ADAM30 | 3.127 | 2.6E-03 |
| --- | DNA binding protein for surfactant protein B | HUMBINDC | 3.122 | 5.7E-03 |
| Hs.376950 | Hypothetical gene supported by AL832797 | --- | 3.118 | 5.0E-02 |
| Hs.397978 | Abhydrolase domain containing 3 | ABHD3 | 3.118 | 8.4E-04 |
| Hs.467304 | interleukin 11 | IL11 | 3.115 | 2.6E-02 |
| Hs.471162 | Ras association (RalGDS/AF-6) and pleckstrin homology domains 1 | RAPH1 | 3.113 | 1.0E-02 |
| Hs.533293 | histone 1, H1t | HIST1H1T | 3.111 | 8.8E-03 |
| Hs.552747 | Homo sapiens, clone IMAGE:4822128, mRNA | --- | 3.107 | 7.2E-05 |
| Hs.502876 | ras homolog gene family, member B | RHOB | 3.104 | 1.8E-03 |
| Hs.380282 | DnaJ (Hsp40) homolog, subfamily B, member 4 | DNAJB4 | 3.104 | 9.7E-04 |
| Hs.396189 | hypothetical protein FLJ39575 | FLJ39575 | 3.104 | 1.5E-03 |
| Hs.474388 | ureidopropionase, beta | UPB1 | 3.101 | 3.7E-03 |
| --- | --- | --- | 3.100 | 3.6E-05 |
| --- | --- | --- | 3.100 | 2.8E-04 |
| Hs.319924 | CDNA FLJ35483 fis, clone SMINT2008277 | --- | 3.098 | 5.4E-04 |
| Hs.467304 | interleukin 11 | IL11 | 3.092 | 4.8E-03 |
| Hs.552612 | rhomboid, veinlet-like 4 (Drosophila) | RHBDL4 | 3.088 | 9.6E-04 |
| Hs.468663 | Ubiquitin specific protease 34 | USP34 | 3.083 | 7.1E-04 |
| Hs.12272 | Beclin 1 (coiled-coil, myosin-like BCL2 interacting protein) | BECN1 | 3.081 | 3.6E-03 |
| Hs.484738 | myosin regulatory light chain interacting protein | MYLIP | 3.079 | 1.7E-04 |
| Hs.551700 | CDNA clone IMAGE:5297041, partial cds | --- | 3.076 | 3.3E-02 |
| Hs.7720 | dynein, cytoplasmic, heavy polypeptide 1 | DNCH1 | 3.076 | 4.6E-02 |
| Hs.444721 | Homo sapiens, clone IMAGE:5301129, mRNA | --- | 3.075 | 2.6E-02 |
| Hs.386217 | Transcribed locus, strongly similar to XP_508034.1 similar to programmed cell death 4 isoform 1; nuclear antigen H731 [Pan troglodytes] | --- | 3.073 | 7.5E-05 |
| Hs.440544 | Chloride intracellular channel 4 | CLIC4 | 3.063 | 7.1E-03 |
| Hs.171132 | Hypothetical gene supported by AK124699 | --- | 3.062 | 9.6E-03 |
| Hs.482301 | Hypothetical protein FLJ13611 | FLJ13611 | 3.057 | 5.2E-03 |
| Hs.436468 | lysozyme-like | LYG2 | 3.056 | 1.1E-02 |
| Hs.334603 | RALBP1 associated Eps domain containing 1 | REPS1 | 3.047 | 1.3E-03 |
| Hs.118076 | Protein (peptidyl-prolyl cis/trans isomerase) NIMA-interacting, 4 (parvulin) | PIN4 | 3.046 | 1.4E-02 |
| Hs.241407 | serine (or cysteine) proteinase inhibitor, clade B (ovalbumin), member 13 | SERPINB13 | 3.046 | 3.5E-02 |
| Hs.489722 | zinc finger protein 277 | ZNF277 | 3.046 | 7.8E-05 |
| Hs.534190 | Chromosome 9 open reading frame 85 | C9orf85 | 3.042 | 2.6E-02 |
| --- | --- | --- | 3.040 | 2.5E-02 |
| Hs.87191 | fibroblast growth factor 18 | FGF18 | 3.040 | 1.8E-02 |
| Hs.536609 | MRNA; cDNA DKFZp686K0736 (from clone DKFZp686K0736) | --- | 3.038 | 8.2E-03 |
| Hs.132314 | EGF, latrophilin and seven transmembrane domain containing 1 | ELTD1 | 3.036 | 1.2E-02 |
| Hs.307926 | G protein interaction factor 2-like mRNA sequence | --- | 3.035 | 1.0E-02 |
| Hs.284707 | Homo sapiens, clone IMAGE:5259731, mRNA | --- | 3.033 | 1.2E-03 |
| Hs.87191 | fibroblast growth factor 18 | FGF18 | 3.032 | 1.3E-02 |
| Hs.459927 | Prothymosin, alpha (gene sequence 28) | PTMA | 3.029 | 2.4E-02 |
| Hs.269011 | Full length insert cDNA clone YW24B11 | --- | 3.026 | 2.2E-03 |
| Hs.193226 | UDP-glucose ceramide glucosyltransferase-like 2 | UGCGL2 | 3.025 | 2.6E-02 |
| Hs.435948 | ATPase family, AAA domain containing 1 | ATAD1 | 3.025 | 4.7E-04 |
| Hs.514116 | zinc finger protein 403 | ZNF403 | 3.025 | 1.8E-03 |
| Hs.530899 | Hypothetical protein LOC162073 | LOC162073 | 3.022 | 3.9E-02 |
| --- | ret finger protein-like 1 antisense | RFPL1S | 3.019 | 1.1E-02 |
| Hs.546573 | Hypothetical LOC388638 | --- | 3.014 | 2.7E-02 |
| Hs.111256 | arachidonate 15-lipoxygenase, second type | ALOX15B | 3.014 | 4.4E-03 |
| Hs.183291 | Zinc finger protein 268 | ZNF268 | 3.014 | 1.4E-04 |
| --- | --- | --- | 3.013 | 1.3E-03 |
| Hs.21816 | CDNA clone IMAGE:4815736, partial cds | --- | 3.010 | 3.4E-03 |
| Hs.494312 | neurotrophic tyrosine kinase, receptor, type 2 | NTRK2 | 3.009 | 4.0E-04 |
| Hs.485352 | EPS8-like 3 | EPS8L3 | 3.007 | 4.2E-03 |
| Hs.104941 | hypothetical protein FLJ25402 | FLJ25402 | 3.005 | 3.1E-04 |
| Hs.369042 | hypothetical protein FLJ20605 | FLJ20605 | 3.001 | 3.8E-02 |
| Hs.112667 | dynein, axonemal, intermediate polypeptide 1 | DNAI1 | 2.999 | 2.7E-02 |
| --- | --- | --- | 2.998 | 5.3E-03 |
| Hs.213861 | laminin, alpha 4 | LAMA4 | 2.997 | 1.4E-03 |
| Hs.371887 | histone 1, H2ba | HIST1H2BA | 2.990 | 4.3E-02 |
| Hs.519719 | fatty acid binding protein 6, ileal (gastrotropin) | FABP6 | 2.989 | 3.8E-02 |
| --- | --- | --- | 2.988 | 1.5E-02 |
| Hs.106070 | Cyclin-dependent kinase inhibitor 1C (p57, Kip2) | CDKN1C | 2.987 | 7.6E-04 |
| Hs.549172 | Ceroid-lipofuscinosis, neuronal 6, late infantile, variant | CLN6 | 2.983 | 4.7E-02 |
| Hs.118704 | Transcribed locus, weakly similar to XP_517655.1 similar to KIAA0825 protein [Pan troglodytes] | --- | 2.983 | 7.6E-06 |
| Hs.484047 | KIBRA protein | KIBRA | 2.981 | 2.5E-04 |
| --- | --- | --- | 2.974 | 3.9E-02 |
| Hs.8619 | SRY (sex determining region Y)-box 18 | SOX18 | 2.969 | 2.2E-06 |
| Hs.464983 | hypothetical gene supported by BC011527; BC021928; BC011527; BC021928 | LOC284260 | 2.967 | 3.5E-02 |
| Hs.385606 | hypothetical protein LOC152024 | LOC152024 | 2.967 | 2.0E-02 |
| Hs.279806 | DEAD (Asp-Glu-Ala-Asp) box polypeptide 5 | DDX5 | 2.964 | 4.0E-02 |
| Hs.523309 | BCL2-associated athanogene 3 | BAG3 | 2.963 | 2.9E-04 |
| Hs.134885 | zinc finger protein 300 | ZNF300 | 2.962 | 2.4E-04 |
| --- | --- | --- | 2.959 | 1.2E-03 |
| Hs.181244 | HLA complex group 4 pseudogene 6 | HCG4P6 | 2.958 | 2.8E-03 |
| Hs.198760 | neurofilament, heavy polypeptide 200kDa | NEFH | 2.954 | 2.5E-02 |
| Hs.435579 | BCR downstream signaling 1 | BRDG1 | 2.948 | 1.8E-02 |
| Hs.387057 | hypothetical protein FLJ13710 | FLJ13710 | 2.941 | 2.6E-02 |
| Hs.421737 | histone 1, H4h | HIST1H4H | 2.940 | 3.5E-02 |
| Hs.476782 | Eukaryotic translation initiation factor 4E member 3 | EIF4E3 | 2.940 | 1.7E-02 |
| Hs.147472 | dynein, axonemal, intermediate polypeptide 2 | DNAI2 | 2.938 | 1.9E-02 |
| Hs.434392 | hypothetical gene supported by BC044741 | LOC387872 | 2.932 | 3.4E-03 |
| Hs.10784 | family with sequence similarity 46, member A | FAM46A | 2.928 | 1.8E-06 |
| --- | --- | --- | 2.928 | 5.9E-03 |
| Hs.445932 | Transcribed locus, weakly similar to XP_510104.1 similar to hypothetical protein FLJ25224 [Pan troglodytes] | --- | 2.921 | 3.5E-03 |
| Hs.194766 | seizure related 6 homolog (mouse)-like | SEZ6L | 2.918 | 3.1E-02 |
| Hs.434729 | Homo sapiens, clone IMAGE:5171726, mRNA | --- | 2.913 | 9.2E-03 |
| Hs.124565 | solute carrier family 23 (nucleobase transporters), member 3 | SLC23A3 | 2.912 | 4.8E-02 |
| Hs.440829 | CCAAT/enhancer binding protein (C/EBP), delta | CEBPD | 2.911 | 4.1E-04 |
| Hs.501898 | Murine retrovirus integration site 1 homolog | MRVI1 | 2.909 | 2.4E-04 |
| Hs.97270 | Family with sequence similarity 13, member A1 | FAM13A1 | 2.905 | 1.1E-02 |
| Hs.458287 | putative N-acetyltransferase Camello 2 | CML2 | 2.903 | 2.9E-03 |
| Hs.248133 | histone 1, H1e | HIST1H1E | 2.902 | 1.5E-02 |
| Hs.27018 | RAS-like, family 12 | RASL12 | 2.901 | 6.9E-05 |
| Hs.415342 | KIAA1049 protein | KIAA1049 | 2.899 | 1.8E-02 |
| Hs.34871 | Zinc finger homeobox 1b | ZFHX1B | 2.899 | 2.5E-04 |
| Hs.484738 | myosin regulatory light chain interacting protein | MYLIP | 2.899 | 2.7E-04 |
| --- | --- | --- | 2.899 | 3.7E-02 |
| Hs.24598 | Rho guanine nucleotide exchange factor (GEF) 12 | ARHGEF12 | 2.896 | 1.5E-04 |
| Hs.9333 | Phosphodiesterase 8A | PDE8A | 2.896 | 3.7E-02 |
| Hs.176227 | hypothetical protein FLJ11155 | FLJ11155 | 2.896 | 3.0E-02 |
| Hs.268698 | methylenetetrahydrofolate dehydrogenase (NADP+ dependent) 1-like | MTHFD1L | 2.895 | 2.2E-02 |
| Hs.432562 | Intersectin 2 | ITSN2 | 2.890 | 1.5E-02 |
| Hs.80756 | betaine-homocysteine methyltransferase | BHMT | 2.888 | 1.3E-03 |
| Hs.531817 | similar to Dynein heavy chain at 16F | LOC200383 | 2.888 | 1.4E-02 |
| Hs.347270 | major histocompatibility complex, class II, DP alpha 1 | HLA-DPA1 | 2.886 | 4.7E-02 |
| Hs.549706 | hypothetical protein MGC16037 | MGC16037 | 2.885 | 5.2E-06 |
| --- | --- | --- | 2.882 | 7.8E-03 |
| Hs.234434 | hairy/enhancer-of-split related with YRPW motif 1 | HEY1 | 2.879 | 4.0E-04 |
| Hs.162121 | coatomer protein complex, subunit alpha | COPA | 2.878 | 5.8E-03 |
| --- | --- | --- | 2.866 | 4.0E-02 |
| Hs.276724 | pancreatic lipase-related protein 3 | PNLIPRP3 | 2.865 | 2.8E-03 |
| Hs.487046 | superoxide dismutase 2, mitochondrial | SOD2 | 2.860 | 3.6E-03 |
| Hs.464585 | Ankyrin repeat domain 12 | ANKRD12 | 2.859 | 2.8E-02 |
| Hs.349150 | Purine-rich element binding protein B | PURB | 2.859 | 9.6E-04 |
| Hs.481466 | Centrosomal protein 72 kDa | Cep72 | 2.858 | 3.5E-02 |
| Hs.334545 | Homo sapiens, clone IMAGE:3503939, mRNA | --- | 2.856 | 1.4E-02 |
| Hs.18858 | phospholipase A2, group IVC (cytosolic, calcium-independent) | PLA2G4C | 2.854 | 3.2E-02 |
| Hs.256184 | RNA binding motif protein 21 | RBM21 | 2.852 | 4.5E-04 |
| Hs.235660 | Homo sapiens, clone IMAGE:5267015, mRNA | --- | 2.852 | 7.8E-03 |
| Hs.347270 | major histocompatibility complex, class II, DP alpha 1 | HLA-DPA1 | 2.849 | 1.2E-02 |
| Hs.550936 | MRNA; cDNA DKFZp586I2322 (from clone DKFZp586I2322) | --- | 2.844 | 2.5E-02 |
| Hs.201776 | Paternally expressed 3 | PEG3 | 2.841 | 3.2E-02 |
| --- | --- | --- | 2.840 | 4.4E-02 |
| Hs.552486 | Homo sapiens, clone IMAGE:4815589, mRNA | --- | 2.840 | 3.0E-02 |
| Hs.485572 | Suppressor of cytokine signaling 2 | SOCS2 | 2.838 | 3.6E-02 |
| Hs.546344 | connector enhancer of kinase suppressor of Ras 2 | CNKSR2 | 2.838 | 2.2E-02 |
| Hs.350624 | CDNA FLJ25058 fis, clone CBL04608 | --- | 2.837 | 3.8E-03 |
| Hs.132371 | similar to bA90M5.1 (novel protein) | LOC440131 | 2.836 | 4.9E-04 |
| Hs.195922 | Sp8 transcription factor | SP8 | 2.836 | 6.0E-04 |
| Hs.462598 | 82-kD FMRP Interacting Protein | 182-FIP | 2.834 | 4.6E-02 |
| Hs.171909 | U2(RNU2) small nuclear RNA auxiliary factor 1-like 2 | U2AF1L2 | 2.833 | 2.9E-02 |
| Hs.179675 | Xg blood group (pseudoautosomal boundary-divided on the X chromosome) | XG | 2.831 | 8.8E-03 |
| Hs.125878 | Synapsin III | SYN3 | 2.826 | 1.6E-02 |
| Hs.533953 | KIAA1462 | KIAA1462 | 2.823 | 3.3E-02 |
| Hs.10784 | family with sequence similarity 46, member A | FAM46A | 2.822 | 1.5E-03 |
| Hs.458657 | neurofilament 3 (150kDa medium) | NEF3 | 2.822 | 2.2E-02 |
| Hs.160562 | insulin-like growth factor 1 (somatomedin C) | IGF1 | 2.816 | 2.2E-03 |
| Hs.504301 | Transmembrane protein 45B | LOC120224 | 2.816 | 7.4E-03 |
| --- | --- | --- | 2.815 | 1.8E-02 |
| Hs.1288 | Actin, alpha 1, skeletal muscle | ACTA1 | 2.815 | 2.0E-02 |
| --- | --- | --- | 2.813 | 4.6E-02 |
| Hs.232026 | tripartite motif-containing 43 | TRIM43 | 2.813 | 8.7E-03 |
| Hs.150556 | Hypothetical protein FLJ43663 | FLJ43663 | 2.812 | 3.8E-02 |
| Hs.449291 | HESB like domain containing 2 | HBLD2 | 2.807 | 8.5E-03 |
| Hs.52931 | Adrenergic, alpha-1A-, receptor | ADRA1A | 2.807 | 2.1E-02 |
| Hs.194746 | calcium channel, voltage-dependent, alpha 1G subunit | CACNA1G | 2.803 | 3.4E-02 |
| Hs.442578 | LIM homeobox 9 | LHX9 | 2.797 | 2.8E-02 |
| Hs.445403 | Isoleucine-tRNA synthetase | IARS | 2.796 | 2.1E-02 |
| --- | --- | --- | 2.793 | 5.3E-03 |
| Hs.527973 | suppressor of cytokine signaling 3 | SOCS3 | 2.793 | 1.5E-02 |
| Hs.533582 | CDC14 cell division cycle 14 homolog A (S. cerevisiae) | CDC14A | 2.790 | 1.6E-02 |
| Hs.148389 | Transcribed locus | --- | 2.788 | 3.3E-02 |
| Hs.441062 | Homo sapiens, clone IMAGE:5180681, mRNA | --- | 2.783 | 3.3E-02 |
| Hs.30917 | cellular repressor of E1A-stimulated genes 2 | CREG2 | 2.783 | 4.9E-02 |
| Hs.146186 | single-minded homolog 2 (Drosophila) | SIM2 | 2.781 | 2.6E-02 |
| Hs.387804 | Poly(A) binding protein, cytoplasmic 1 | PABPC1 | 2.780 | 1.5E-03 |
| Hs.530461 | histone 2, H2aa | HIST2H2AA | 2.778 | 5.3E-04 |
| Hs.118722 | Fucosyltransferase 8 (alpha (1,6) fucosyltransferase) | FUT8 | 2.777 | 9.0E-03 |
| Hs.520319 | solute carrier family 22 (organic cation transporter), member 16 | SLC22A16 | 2.774 | 1.3E-04 |
| Hs.291899 | CDNA clone IMAGE:4794631, partial cds | --- | 2.772 | 8.2E-03 |
| Hs.200644 | Hypothetical protein FLJ39609 | FLJ39609 | 2.770 | 3.4E-03 |
| Hs.542925 | MRNA; cDNA DKFZp313P0714 (from clone DKFZp313P0714) | --- | 2.765 | 2.3E-02 |
| Hs.256067 | protein kinase, AMP-activated, alpha 2 catalytic subunit | PRKAA2 | 2.764 | 3.4E-02 |
| Hs.460 | activating transcription factor 3 | ATF3 | 2.756 | 1.2E-04 |
| Hs.177983 | FLJ27505 protein | FLJ27505 | 2.755 | 1.8E-03 |
| Hs.34558 | CDNA: FLJ21199 fis, clone COL00235 | --- | 2.755 | 4.9E-04 |
| Hs.375108 | CD24 antigen (small cell lung carcinoma cluster 4 antigen) | CD24 | 2.753 | 3.3E-02 |
| Hs.123450 | junctophilin 3 | JPH3 | 2.753 | 7.2E-04 |
| Hs.467338 | potassium inwardly-rectifying channel, subfamily J, member 13 | KCNJ13 | 2.753 | 4.1E-02 |
| --- | dynein, axonemal, heavy polypeptide 1 | DNAH1 | 2.753 | 3.5E-02 |
| Hs.192586 | similar to mouse 2310016A09Rik gene | LOC134147 | 2.753 | 1.5E-02 |
| Hs.250723 | Transmembrane anchor protein 1 | TMAP1 | 2.752 | 3.7E-03 |
| Hs.465087 | SMAD, mothers against DPP homolog 7 (Drosophila) | SMAD7 | 2.752 | 2.6E-03 |
| Hs.114191 | zinc finger, CCHC domain containing 2 | ZCCHC2 | 2.751 | 3.1E-02 |
| Hs.8867 | cysteine-rich, angiogenic inducer, 61 | CYR61 | 2.750 | 1.4E-02 |
| Hs.495674 | chloride channel 4 | CLCN4 | 2.746 | 1.4E-02 |
| Hs.335034 | Dihydropyrimidine dehydrogenase | DPYD | 2.745 | 8.5E-03 |
| Hs.238094 | heat shock protein, alpha-crystallin-related, B9 | HSPB9 | 2.745 | 4.0E-04 |
| Hs.194766 | Seizure related 6 homolog (mouse)-like | SEZ6L | 2.742 | 3.9E-03 |
| Hs.106070 | cyclin-dependent kinase inhibitor 1C (p57, Kip2) | CDKN1C | 2.740 | 3.0E-02 |
| Hs.546736 | Transcribed locus | --- | 2.739 | 3.2E-03 |
| Hs.388622 | zinc finger protein, subfamily 1A, 4 (Eos) | ZNFN1A4 | 2.737 | 1.3E-02 |
| Hs.32043 | Transcribed locus, weakly similar to XP_371797.1 hypothetical LOC389365 [Homo sapiens] | --- | 2.733 | 3.6E-02 |
| --- | --- | --- | 2.731 | 8.8E-03 |
| Hs.500822 | F-box and WD-40 domain protein 4 | SHFM3 | 2.731 | 5.5E-03 |
| Hs.272011 | UDP-Gal:betaGlcNAc beta 1,4- galactosyltransferase, polypeptide 1 | B4GALT1 | 2.731 | 1.3E-04 |
| Hs.549032 | eyes absent homolog 4 (Drosophila) | EYA4 | 2.731 | 1.9E-02 |
| Hs.542781 | Homo sapiens, clone IMAGE:4701591, mRNA | --- | 2.730 | 1.4E-02 |
| Hs.486246 | putative homeodomain transcription factor 1 | PHTF1 | 2.729 | 1.8E-03 |
| Hs.131152 | similar to SERTA domain containing 4 | LOC401778 | 2.729 | 1.1E-02 |
| Hs.480068 | RasGEF domain family, member 1B | RASGEF1B | 2.727 | 1.4E-02 |
| Hs.413494 | Collagen, type XXIII, alpha 1 | COL23A1 | 2.725 | 3.3E-03 |
| Hs.131543 | CCCTC-binding factor (zinc finger protein)-like | CTCFL | 2.723 | 3.7E-02 |
| Hs.490817 | vasoactive intestinal peptide receptor 2 /// vasoactive intestinal peptide receptor 2 | VIPR2 | 2.723 | 1.9E-02 |
| Hs.355264 | cytochrome b-561 | CYB561 | 2.723 | 8.4E-03 |
| Hs.534035 | histone 1, H2ai | HIST1H2AI | 2.720 | 3.5E-02 |
| --- | similar to mouse 1700027M21Rik gene | LOC493861 | 2.714 | 5.3E-04 |
| Hs.531695 | Homo sapiens, clone IMAGE:4826083, mRNA | --- | 2.705 | 4.0E-02 |
| Hs.471200 | Neuropilin 2 | NRP2 | 2.703 | 2.7E-02 |
| Hs.434686 | Homo sapiens, clone IMAGE:4723759, mRNA | --- | 2.701 | 2.8E-03 |
| --- | --- | --- | 2.699 | 2.3E-02 |
| Hs.436018 | Transcribed locus | --- | 2.696 | 2.5E-03 |
| Hs.73839 | ribonuclease, RNase A family, 3 (eosinophil cationic protein) | RNASE3 | 2.696 | 5.5E-05 |
| --- | --- | --- | 2.693 | 7.4E-03 |
| Hs.143873 | S100 calcium binding protein A10 (annexin II ligand, calpactin I, light polypeptide (p11)) | S100A10 | 2.690 | 2.1E-02 |
| Hs.513313 | LOC124402 | LOC124402 | 2.686 | 2.0E-03 |
| Hs.523702 | membrane-spanning 4-domains, subfamily A, member 6A | MS4A6A | 2.686 | 2.3E-03 |
| Hs.144221 | hypothetical protein HUMYZ82H07 | HUMYZ82H07 | 2.684 | 3.3E-02 |
| Hs.427229 | Transcribed locus | --- | 2.682 | 1.5E-02 |
| Hs.546343 | chloride channel, calcium activated, family member 4 | CLCA4 | 2.680 | 4.7E-02 |
| Hs.56186 | EGF-like-domain, multiple 3 | EGFL3 | 2.679 | 1.5E-02 |
| Hs.422688 | retinol binding protein 7, cellular | RBP7 | 2.677 | 3.9E-03 |
| Hs.190365 | Hypothetical LOC401048 | --- | 2.676 | 1.5E-02 |
| Hs.522561 | gap junction protein, beta 3, 31kDa (connexin 31) | GJB3 | 2.672 | 3.3E-02 |
| Hs.197644 | headcase homolog (Drosophila) | HECA | 2.671 | 3.2E-04 |
| Hs.165258 | nuclear receptor subfamily 4, group A, member 2 | NR4A2 | 2.669 | 4.7E-02 |
| Hs.372914 | N-myc downstream regulated gene 1 | NDRG1 | 2.668 | 7.9E-03 |
| Hs.127951 | hypothetical protein FLJ14503 | FLJ14503 | 2.668 | 1.5E-02 |
| Hs.154429 | Synovial sarcoma translocation gene on chromosome 18-like 1 | SS18L1 | 2.667 | 6.9E-04 |
| Hs.149239 | ephrin-B2 | EFNB2 | 2.663 | 6.5E-03 |
| Hs.129136 | RAS and EF hand domain containing | RASEF | 2.663 | 2.6E-04 |
| Hs.523774 | EH-domain containing 1 | EHD1 | 2.660 | 1.0E-03 |
| Hs.460 | activating transcription factor 3 | ATF3 | 2.659 | 2.2E-02 |
| Hs.282931 | Solute carrier family 17 (sodium phosphate), member 4 | SLC17A4 | 2.654 | 3.4E-02 |
| Hs.126980 | Homo sapiens, clone IMAGE:5266948, mRNA | --- | 2.654 | 4.0E-02 |
| Hs.279522 | nuclear receptor subfamily 4, group A, member 3 | NR4A3 | 2.652 | 7.0E-03 |
| Hs.48950 | dapper homolog 1, antagonist of beta-catenin (xenopus) | DACT1 | 2.651 | 4.5E-02 |
| --- | endogenous retroviral family W, env(C7), member 1 (syncytin) | ERVWE1 | 2.649 | 6.0E-04 |
| Hs.227049 | CTP synthase II | CTPS2 | 2.649 | 2.5E-02 |
| Hs.73793 | vascular endothelial growth factor | VEGF | 2.649 | 1.2E-03 |
| Hs.248178 | histone 1, H4a | HIST1H4A | 2.642 | 1.1E-03 |
| Hs.133397 | Integrin, alpha 6 | ITGA6 | 2.641 | 1.3E-02 |
| Hs.46730 | FBI4 protein | FBI4 | 2.636 | 5.0E-02 |
| Hs.209492 | Transcribed locus, weakly similar to XP_510104.1 similar to hypothetical protein FLJ25224 [Pan troglodytes] | --- | 2.636 | 3.3E-02 |
| --- | hypothetical protein LOC338651 | LOC338651 | 2.635 | 6.7E-04 |
| Hs.439031 | Transcribed locus, weakly similar to XP_510104.1 similar to hypothetical protein FLJ25224 [Pan troglodytes] | --- | 2.634 | 3.3E-02 |
| Hs.531753 | Homo sapiens, clone IMAGE:5171428, mRNA | --- | 2.633 | 3.3E-03 |
| Hs.167371 | RAS protein activator like 2 | RASAL2 | 2.632 | 3.0E-02 |
| Hs.21423 | CDNA FLJ30424 fis, clone BRACE2008881, weakly similar to ZINC FINGER PROTEIN 195 | --- | 2.631 | 3.8E-02 |
| --- | --- | --- | 2.629 | 6.1E-04 |
| Hs.193326 | fibroblast growth factor receptor-like 1 | FGFRL1 | 2.627 | 1.6E-02 |
| Hs.122186 | nephrosis 1, congenital, Finnish type (nephrin) | NPHS1 | 2.627 | 2.4E-02 |
| Hs.76556 | protein phosphatase 1, regulatory (inhibitor) subunit 15A | PPP1R15A | 2.625 | 1.1E-04 |
| Hs.154036 | pleckstrin homology-like domain, family A, member 2 | PHLDA2 | 2.623 | 1.0E-02 |
| Hs.401045 | zinc finger protein 501 | ZNF501 | 2.621 | 2.4E-03 |
| Hs.252387 | cadherin, EGF LAG seven-pass G-type receptor 1 (flamingo homolog, Drosophila) | CELSR1 | 2.620 | 2.8E-02 |
| Hs.307052 | lactate dehydrogenase A-like 6B | LDHAL6B | 2.618 | 4.3E-04 |
| Hs.549792 | Transcribed locus, weakly similar to XP_510104.1 similar to hypothetical protein FLJ25224 [Pan troglodytes] | --- | 2.617 | 4.6E-02 |
| Hs.435064 | KIAA1608 | KIAA1608 | 2.615 | 1.5E-02 |
| Hs.412421 | Hypothetical protein MGC13168 | MGC13168 | 2.612 | 3.5E-02 |
| Hs.110571 | Growth arrest and DNA-damage-inducible, beta | GADD45B | 2.612 | 1.8E-05 |
| Hs.523454 | tripeptidyl peptidase I | TPP1 | 2.610 | 4.8E-02 |
| Hs.468058 | UDP-N-acetyl-alpha-D-galactosamine:polypeptide N-acetylgalactosaminyltransferase 14 (GalNAc-T14) | GALNT14 | 2.607 | 1.2E-03 |
| Hs.126005 | Homo sapiens, clone IMAGE:5266772, mRNA | --- | 2.606 | 4.7E-02 |
| Hs.175687 | peroxisomal proliferator-activated receptor A interacting complex 285 | PRIC285 | 2.604 | 3.4E-02 |
| Hs.143751 | matrix metalloproteinase 11 (stromelysin 3) | MMP11 | 2.603 | 3.0E-02 |
| Hs.150858 | chromosome 6 open reading frame 50 | C6orf50 | 2.599 | 1.3E-02 |
| Hs.513541 | similar to hect domain and RLD 2 | LOC440366 | 2.596 | 1.9E-02 |
| Hs.535921 | Homo sapiens, clone IMAGE:5225645, mRNA | --- | 2.596 | 6.3E-03 |
| Hs.9740 | Heat shock regulated 1 | XLHSRF-1 | 2.593 | 4.8E-02 |
| Hs.34341 | CD58 antigen, (lymphocyte function-associated antigen 3) | CD58 | 2.591 | 2.1E-02 |
| --- | --- | --- | 2.589 | 2.2E-02 |
| Hs.54973 | cadherin-like 26 | CDH26 | 2.589 | 3.9E-03 |
| Hs.296529 | CDNA FLJ30762 fis, clone FEBRA2000575 | --- | 2.587 | 8.7E-03 |
| Hs.35861 | Ras-induced senescence 1 | RIS1 | 2.587 | 1.8E-02 |
| Hs.268724 | Transcribed locus | --- | 2.587 | 2.4E-02 |
| Hs.497369 | Neuron navigator 1 | NAV1 | 2.586 | 2.3E-02 |
| Hs.203594 | Chromosome 16 open reading frame 46 | FLJ32702 | 2.581 | 7.7E-04 |
| --- | --- | --- | 2.581 | 2.8E-04 |
| Hs.129136 | RAS and EF hand domain containing | RASEF | 2.581 | 3.3E-04 |
| Hs.821 | biglycan /// serologically defined colon cancer antigen 33 | BGN /// SDCCAG33 | 2.580 | 4.1E-03 |
| Hs.132161 | Forkhead box K2 | FOXK2 | 2.576 | 2.0E-03 |
| Hs.247812 | hypothetical protein MGC12935 | MGC12935 | 2.572 | 1.8E-03 |
| Hs.550979 | CDNA FLJ10031 fis, clone HEMBA1000867 | --- | 2.569 | 3.9E-02 |
| Hs.123106 | Transcribed locus | --- | 2.568 | 3.5E-05 |
| Hs.200639 | Transcribed locus | --- | 2.567 | 3.9E-02 |
| --- | --- | --- | 2.567 | 3.3E-02 |
| Hs.486109 | sex comb on midleg-like 4 (Drosophila) | SCML4 | 2.566 | 2.0E-03 |
| Hs.471525 | collagen, type IV, alpha 3 (Goodpasture antigen) | COL4A3 | 2.564 | 2.5E-02 |
| Hs.23589 | Full length insert cDNA YI09H09 | --- | 2.562 | 4.6E-02 |
| Hs.385635 | Homo sapiens, clone IMAGE:4828073, mRNA | --- | 2.562 | 2.4E-02 |
| Hs.535651 | Transcribed locus | --- | 2.561 | 1.4E-03 |
| Hs.128316 | BAI1-associated protein 2 | BAIAP2 | 2.558 | 7.0E-03 |
| Hs.176225 | CDNA clone IMAGE:5541269, partial cds | --- | 2.558 | 2.5E-02 |
| Hs.145519 | FKSG87 protein | FCA/MR | 2.557 | 7.6E-03 |
| Hs.158336 | netrin 2-like (chicken) | NTN2L | 2.554 | 4.9E-02 |
| Hs.524894 | gap junction protein, beta 2, 26kDa (connexin 26) | GJB2 | 2.553 | 1.6E-03 |
| Hs.31664 | frizzled homolog 10 (Drosophila) | FZD10 | 2.552 | 1.4E-02 |
| Hs.109437 | Hormonally upregulated Neu-associated kinase | HUNK | 2.551 | 2.0E-02 |
| Hs.444225 | estrogen-related receptor gamma | ESRRG | 2.547 | 4.6E-05 |
| Hs.517718 | hypothetical LOC388914 | LOC388914 | 2.547 | 2.2E-02 |
| --- | --- | --- | 2.545 | 3.8E-02 |
| Hs.325960 | membrane-spanning 4-domains, subfamily A, member 4 | MS4A4A | 2.545 | 4.5E-03 |
| Hs.532680 | atonal homolog 1 (Drosophila) | ATOH1 | 2.539 | 3.1E-02 |
| Hs.369999 | Transcribed locus | --- | 2.538 | 2.9E-03 |
| Hs.76152 | aquaporin 1 (channel-forming integral protein, 28kDa) | AQP1 | 2.538 | 6.4E-03 |
| Hs.365706 | matrix Gla protein | MGP | 2.537 | 6.2E-04 |
| Hs.484738 | myosin regulatory light chain interacting protein | MYLIP | 2.534 | 1.7E-03 |
| Hs.507087 | Signal peptide peptidase 3 | SPPL3 | 2.534 | 1.1E-02 |
| Hs.446050 | ATP-binding cassette, sub-family C (CFTR/MRP), member 9 | ABCC9 | 2.531 | 4.6E-02 |
| Hs.104944 | Homo sapiens, Similar to hypothetical protein PRO2852, clone IMAGE:4837965, mRNA | --- | 2.530 | 2.8E-02 |
| Hs.178749 | synovial sarcoma, X breakpoint 3 | SSX3 | 2.529 | 4.7E-02 |
| Hs.35101 | proline rich Gla (G-carboxyglutamic acid) 2 | PRRG2 | 2.527 | 5.4E-03 |
| Hs.12249 | Transcribed locus | --- | 2.526 | 1.3E-03 |
| Hs.380027 | transcriptional activator of the c-fos promoter | CROC4 | 2.525 | 2.1E-03 |
| Hs.531803 | Homo sapiens, clone IMAGE:5271111, mRNA | --- | 2.522 | 2.3E-02 |
| Hs.193011 | CDNA clone IMAGE:4151570, partial cds | --- | 2.522 | 3.5E-02 |
| Hs.493275 | tripartite motif-containing 31 | TRIM31 | 2.521 | 1.3E-02 |
| Hs.435479 | Protein phosphatase 1H (PP2C domain containing) | PPM1H | 2.520 | 1.3E-02 |
| Hs.497822 | dual specificity phosphatase 10 | DUSP10 | 2.519 | 6.4E-05 |
| Hs.309288 | CUG triplet repeat, RNA binding protein 2 | CUGBP2 | 2.519 | 3.6E-03 |
| Hs.528335 | Hypothetical protein FLJ25477 | FLJ25477 | 2.519 | 3.0E-03 |
| Hs.35125 | hypothetical protein FLJ10357 | FLJ10357 | 2.515 | 4.1E-03 |
| Hs.474388 | Ureidopropionase, beta | UPB1 | 2.515 | 3.1E-02 |
| Hs.523789 | trophoblast-derived noncoding RNA | TncRNA | 2.510 | 6.2E-03 |
| Hs.148078 | retinoblastoma-associated factor 600 | RBAF600 | 2.510 | 4.5E-02 |
| Hs.524971 | Ribosomal protein L37 | RPL37 | 2.509 | 1.5E-02 |
| Hs.418062 | UDP-Gal:betaGlcNAc beta 1,3-galactosyltransferase, polypeptide 3 | B3GALT3 | 2.506 | 5.7E-03 |
| Hs.193170 | PDZ domain containing, X chromosome | FLJ21687 | 2.505 | 5.6E-03 |
| Hs.458986 | Zinc finger protein 291 | ZNF291 | 2.500 | 5.0E-02 |
| --- | --- | --- | 2.496 | 5.7E-03 |
| Hs.546361 | ATPase, Ca++ transporting, type 2C, member 1 | ATP2C1 | 2.496 | 4.9E-02 |
| Hs.502116 | neuron navigator 2 | NAV2 | 2.496 | 2.1E-02 |
| Hs.17267 | Chromosome 9 open reading frame 93 | C9orf93 | 2.494 | 5.0E-02 |
| Hs.15725 | Immediate early response 5 | IER5 | 2.492 | 1.4E-03 |
| Hs.432648 | heat shock 70kDa protein 2 | HSPA2 | 2.490 | 2.5E-05 |
| Hs.479898 | sulfotransferase family 1E, estrogen-preferring, member 1 | SULT1E1 | 2.485 | 2.3E-03 |
| Hs.364337 | FLJ45224 protein | FLJ45224 | 2.484 | 2.8E-02 |
| Hs.550144 | CDNA clone IMAGE:3621839, partial cds | --- | 2.483 | 2.5E-03 |
| Hs.76095 | immediate early response 3 | IER3 | 2.482 | 6.1E-04 |
| Hs.143080 | histone 1, H4b | HIST1H4B | 2.482 | 1.8E-04 |
| Hs.97084 | natural cytotoxicity triggering receptor 1 | NCR1 | 2.482 | 1.3E-02 |
| Hs.445725 | SKI-like | SKIL | 2.480 | 7.1E-05 |
| Hs.374067 | Ubiquitin protein ligase E3B | UBE3B | 2.480 | 8.5E-04 |
| Hs.61389 | chromosome 6 open reading frame 52 | C6orf52 | 2.477 | 2.3E-02 |
| Hs.461688 | Kelch domain containing 4 | KLHDC4 | 2.476 | 2.8E-03 |
| --- | --- | --- | 2.476 | 2.8E-02 |
| Hs.523789 | Trophoblast-derived noncoding RNA | TncRNA | 2.476 | 2.5E-03 |
| Hs.478553 | eukaryotic translation initiation factor 4A, isoform 2 | EIF4A2 | 2.475 | 1.5E-03 |
| Hs.369935 | Homo sapiens, clone IMAGE:4838568, mRNA | --- | 2.474 | 1.4E-02 |
| --- | --- | --- | 2.473 | 6.0E-03 |
| Hs.471918 | MCP-1 treatment-induced protein | MCPIP | 2.470 | 1.5E-04 |
| Hs.13680 | rififylin | RFFL | 2.469 | 4.4E-03 |
| Hs.448871 | Homo sapiens, clone IMAGE:4940467, mRNA | --- | 2.466 | 2.9E-02 |
| --- | --- | --- | 2.465 | 3.7E-05 |
| Hs.43977 | chromosome 20 open reading frame 85 | C20orf85 | 2.464 | 1.2E-02 |
| Hs.445725 | SKI-like | SKIL | 2.464 | 3.0E-02 |
| Hs.414110 | potassium voltage-gated channel, Shal-related subfamily, member 3 | KCND3 | 2.460 | 6.4E-03 |
| Hs.102471 | Phosphatase and actin regulator 2 | PHACTR2 | 2.450 | 4.1E-02 |
| Hs.159013 | Ras homolog enriched in brain like 1 | RHEBL1 | 2.450 | 1.2E-02 |
| Hs.181552 | zinc finger protein 140 (clone pHZ-39) | ZNF140 | 2.448 | 2.2E-02 |
| Hs.82028 | Transforming growth factor, beta receptor II (70/80kDa) | TGFBR2 | 2.447 | 6.8E-03 |
| Hs.471751 | Chemokine orphan receptor 1 | CMKOR1 | 2.444 | 2.2E-02 |
| Hs.369042 | hypothetical protein FLJ20605 | FLJ20605 | 2.444 | 4.6E-02 |
| Hs.472690 | KIAA1755 protein | KIAA1755 | 2.442 | 3.5E-02 |
| Hs.534462 | Hypothetical protein FLJ10213 | FLJ10213 | 2.440 | 3.7E-02 |
| Hs.347991 | nuclear receptor subfamily 2, group F, member 2 | NR2F2 | 2.439 | 6.8E-03 |
| Hs.445015 | glutamate receptor, ionotropic, N-methyl D-aspartate 2D | GRIN2D | 2.439 | 4.8E-02 |
| Hs.100874 | KIAA0494 gene product | KIAA0494 | 2.438 | 3.6E-05 |
| Hs.55047 | Full length insert cDNA clone ZB77E08 | --- | 2.435 | 3.8E-02 |
| Hs.131755 | chromosome 14 open reading frame 161 | C14orf161 | 2.433 | 3.5E-02 |
| Hs.406847 | CDNA: FLJ20931 fis, clone ADSE01282 | --- | 2.430 | 1.6E-04 |
| Hs.552756 | Homo sapiens, Similar to hypothetical protein FLJ21463, clone IMAGE:4794524, mRNA | --- | 2.429 | 1.2E-02 |
| Hs.98947 | similar to RIKEN cDNA 1700016G05 | LOC136242 | 2.427 | 1.8E-02 |
| Hs.149566 | Formin-like 2 | FMNL2 | 2.426 | 1.5E-02 |
| Hs.396189 | hypothetical protein FLJ39575 | FLJ39575 | 2.425 | 7.6E-03 |
| Hs.58351 | ATP-binding cassette, sub-family A (ABC1), member 8 | ABCA8 | 2.424 | 1.9E-02 |
| --- | --- | --- | 2.424 | 4.7E-02 |
| Hs.415766 | Zic family member 4 | ZIC4 | 2.423 | 1.7E-04 |
| --- | --- | --- | 2.423 | 1.3E-02 |
| Hs.515260 | Elongation factor RNA polymerase II | ELL | 2.422 | 1.0E-02 |
| Hs.549543 | Homo sapiens, clone IMAGE:5215917, mRNA | --- | 2.421 | 1.8E-02 |
| Hs.100874 | KIAA0494 gene product | KIAA0494 | 2.421 | 2.6E-04 |
| Hs.529892 | Sequestosome 1 | SQSTM1 | 2.421 | 4.0E-04 |
| Hs.321273 | SEC8-like 1 (S. cerevisiae) | SEC8L1 | 2.416 | 4.5E-02 |
| Hs.62646 | Homo sapiens, clone IMAGE:5557975, mRNA | --- | 2.416 | 4.2E-02 |
| Hs.133916 | Hypothetical protein LOC152485 | LOC152485 | 2.415 | 2.2E-02 |
| Hs.365207 | thyroid hormone receptor associated protein 5 | THRAP5 | 2.414 | 4.2E-02 |
| Hs.492618 | Exostoses (multiple) 1 | EXT1 | 2.410 | 2.6E-03 |
| Hs.485616 | dystonin | DST | 2.406 | 3.9E-02 |
| Hs.130544 | Transcribed locus | --- | 2.406 | 1.4E-02 |
| Hs.75360 | carboxypeptidase E | CPE | 2.404 | 2.6E-02 |
| Hs.339 | purinergic receptor P2Y, G-protein coupled, 2 | P2RY2 | 2.404 | 3.8E-02 |
| Hs.489051 | six transmembrane epithelial antigen of the prostate 2 | STEAP2 | 2.402 | 2.4E-02 |
| Hs.533887 | KIAA2010 | KIAA2010 | 2.400 | 2.7E-03 |
| Hs.502756 | AHNAK nucleoprotein (desmoyokin) | AHNAK | 2.400 | 1.6E-04 |
| Hs.365689 | immunoglobulin superfamily, member 4B | IGSF4B | 2.399 | 2.7E-03 |
| Hs.389103 | glucagon-like peptide 1 receptor | GLP1R | 2.398 | 7.1E-03 |
| Hs.129966 | Ciliary neurotrophic factor receptor | CNTFR | 2.393 | 7.3E-03 |
| Hs.206500 | cortactin | CTTN | 2.390 | 3.5E-02 |
| Hs.546416 | KIAA1632 | KIAA1632 | 2.390 | 2.3E-03 |
| Hs.502977 | Cardiotrophin-like cytokine factor 1 | CLC | 2.390 | 6.1E-03 |
| Hs.435988 | Transcribed locus | --- | 2.388 | 7.5E-03 |
| Hs.386567 | guanylate binding protein 2, interferon-inducible | GBP2 | 2.381 | 7.7E-03 |
| Hs.510324 | Quaking homolog, KH domain RNA binding (mouse) | QKI | 2.378 | 2.7E-04 |
| Hs.22634 | ets variant gene 1 /// hypothetical protein LOC221810 | ETV1 /// LOC221810 | 2.377 | 3.8E-02 |
| Hs.145675 | Sortilin-related VPS10 domain containing receptor 1 | SORCS1 | 2.375 | 3.2E-03 |
| Hs.160063 | FLJ16124 protein | FLJ16124 | 2.373 | 9.1E-03 |
| Hs.73849 | Apolipoprotein C-III | APOC3 | 2.372 | 2.1E-02 |
| Hs.331981 | DAN domain family, member 5 | DAND5 | 2.372 | 3.7E-04 |
| Hs.256036 | HLH-PAS transcription factor NXF | NXF | 2.372 | 1.6E-02 |
| --- | --- | --- | 2.372 | 2.1E-02 |
| Hs.520710 | KIAA0265 protein | KIAA0265 | 2.372 | 3.9E-02 |
| Hs.443139 | hypothetical protein KIAA1833 | KIAA1833 | 2.371 | 4.6E-02 |
| --- | --- | --- | 2.367 | 5.8E-03 |
| Hs.117688 | Transcribed locus | --- | 2.364 | 1.0E-03 |
| Hs.284450 | Transcribed locus | --- | 2.364 | 2.8E-02 |
| Hs.514289 | homeo box B2 | HOXB2 | 2.364 | 1.1E-02 |
| --- | --- | --- | 2.362 | 1.3E-02 |
| Hs.148590 | cornifelin /// cornifelin | CNFN | 2.361 | 3.1E-02 |
| Hs.484991 | histone 1, H2bo | HIST1H2BO | 2.360 | 1.5E-02 |
| --- | --- | --- | 2.360 | 3.2E-03 |
| Hs.523446 | collagen, type XI, alpha 1 | COL11A1 | 2.360 | 2.1E-02 |
| Hs.130086 | Chromosome 9 open reading frame 52 | C9orf52 | 2.356 | 4.3E-02 |
| Hs.172550 | Polypyrimidine tract binding protein 1 | PTBP1 | 2.350 | 1.8E-03 |
| Hs.545458 | CDNA clone IMAGE:5310996, partial cds | --- | 2.349 | 1.2E-02 |
| Hs.435458 | SET binding protein 1 | SETBP1 | 2.348 | 4.7E-02 |
| Hs.28773 | Transcribed locus | --- | 2.346 | 4.0E-02 |
| Hs.488143 | biliverdin reductase A | BLVRA | 2.345 | 4.9E-02 |
| Hs.552392 | Nucleoporin (GYLZ-RCC18) mRNA, GYLZ-RCC18-NUP2 allele | --- | 2.344 | 9.5E-03 |
| --- | --- | --- | 2.343 | 4.3E-04 |
| --- | --- | --- | 2.343 | 3.4E-04 |
| --- | --- | --- | 2.343 | 4.6E-02 |
| Hs.370666 | Forkhead box O1A (rhabdomyosarcoma) | FOXO1A | 2.340 | 2.2E-02 |
| Hs.120893 | Transcribed locus | --- | 2.339 | 1.7E-02 |
| Hs.514814 | acyl-malonyl condensing enzyme 1 | AMAC1 | 2.334 | 4.9E-02 |
| Hs.520721 | CDNA FLJ31668 fis, clone NT2RI2004916 | --- | 2.334 | 1.5E-02 |
| Hs.389374 | Hypothetical protein LOC257106 | LOC257106 | 2.333 | 3.3E-02 |
| Hs.195040 | hydroxysteroid (11-beta) dehydrogenase 1 | HSD11B1 | 2.332 | 2.6E-02 |
| --- | --- | --- | 2.332 | 4.4E-03 |
| Hs.113912 | Rap guanine nucleotide exchange factor (GEF) 2 | RAPGEF2 | 2.331 | 2.0E-02 |
| --- | --- | --- | 2.330 | 2.5E-02 |
| Hs.549129 | Deleted in a mouse model of primary ciliary dyskinesia | DPCD | 2.330 | 2.4E-03 |
| Hs.296832 | PTR7 mRNA for repetitive sequence | --- | 2.329 | 9.3E-04 |
| Hs.115838 | transmembrane channel-like 5 | TMC5 | 2.329 | 3.0E-02 |
| Hs.125898 | GNAS complex locus | GNAS | 2.329 | 1.9E-04 |
| Hs.272295 | interleukin 17F | IL17F | 2.328 | 1.3E-02 |
| Hs.414396 | PERQ amino acid rich, with GYF domain 1 | PERQ1 | 2.327 | 2.7E-02 |
| --- | --- | --- | 2.327 | 4.1E-02 |
| --- | --- | --- | 2.326 | 1.3E-03 |
| Hs.136900 | Membrane-associated ring finger (C3HC4) 1 | MARCH-I | 2.326 | 4.1E-02 |
| --- | --- | --- | 2.326 | 1.4E-02 |
| Hs.125898 | GNAS complex locus | GNAS | 2.325 | 2.9E-02 |
| Hs.35828 | MAP/microtubule affinity-regulating kinase 3 | MARK3 | 2.325 | 4.9E-02 |
| Hs.6421 | hypothetical protein DKFZp761N09121 | DKFZP761N09121 | 2.325 | 4.8E-02 |
| Hs.381568 | Immunoglobulin J polypeptide, linker protein for immunoglobulin alpha and mu polypeptides | IGJ | 2.324 | 4.6E-02 |
| Hs.248171 | histone 3, H3 | HIST3H3 | 2.323 | 4.9E-03 |
| --- | --- | --- | 2.323 | 4.9E-02 |
| Hs.130774 | F-box protein 10 | FBXO10 | 2.323 | 4.2E-02 |
| Hs.530311 | lipocalin 1 (tear prealbumin) | LCN1 | 2.322 | 3.6E-02 |
| --- | --- | --- | 2.322 | 2.3E-02 |
| Hs.523841 | Aldehyde dehydrogenase 3 family, member B1 | ALDH3B1 | 2.321 | 1.9E-02 |
| Hs.518234 | Homo sapiens, clone IMAGE:4822964, mRNA | --- | 2.321 | 9.1E-03 |
| Hs.405144 | Splicing factor, arginine/serine-rich 3 | SFRS3 | 2.320 | 1.5E-02 |
| Hs.104633 | agouti related protein homolog (mouse) | AGRP | 2.319 | 1.1E-02 |
| Hs.317740 | Transcribed locus | --- | 2.319 | 9.8E-03 |
| Hs.509182 | chromosome 14 open reading frame 25 | C14orf25 | 2.318 | 3.1E-04 |
| Hs.191591 | HSPC324 | --- | 2.317 | 1.7E-02 |
| Hs.432854 | Pepsinogen 5, group I (pepsinogen A) | PGA5 | 2.317 | 2.8E-02 |
| Hs.355236 | chromosome 15 open reading frame 16 | C15orf16 | 2.317 | 3.3E-02 |
| Hs.183114 | Rho GTPase activating protein 28 | ARHGAP28 | 2.312 | 3.1E-02 |
| Hs.447968 | gap junction protein, alpha 5, 40kDa (connexin 40) | GJA5 | 2.310 | 3.5E-03 |
| Hs.81848 | RAD21 homolog (S. pombe) | RAD21 | 2.309 | 3.0E-02 |
| Hs.433722 | KIAA1967 | KIAA1967 | 2.307 | 4.0E-02 |
| Hs.154140 | ovary-specific acidic protein | OSAP | 2.307 | 3.7E-04 |
| Hs.519018 | SH3 domain protein D19 | EVE1 | 2.306 | 2.9E-02 |
| Hs.165904 | epsin 3 | EPN3 | 2.306 | 2.7E-02 |
| Hs.515016 | melanoma associated antigen (mutated) 1 | MUM1 | 2.305 | 1.8E-02 |
| Hs.385753 | Homo sapiens, clone IMAGE:4796629, mRNA | --- | 2.302 | 3.4E-02 |
| --- | --- | --- | 2.302 | 3.4E-02 |
| Hs.52931 | adrenergic, alpha-1A-, receptor | ADRA1A | 2.302 | 1.4E-03 |
| Hs.450694 | Hephaestin-like 1 | HEPHL1 | 2.301 | 1.0E-02 |
| Hs.248158 | POU domain, class 3, transcription factor 3 | POU3F3 | 2.298 | 2.3E-02 |
| Hs.432706 | Hypothetical protein DKFZp434D2328 | LOC91526 | 2.298 | 4.7E-02 |
| Hs.397553 | CDNA clone IMAGE:6049242, partial cds | --- | 2.298 | 2.8E-03 |
| Hs.176225 | CDNA clone IMAGE:5541269, partial cds | --- | 2.297 | 3.7E-02 |
| Hs.267749 | unc-93 homolog A (C. elegans) | UNC93A | 2.297 | 2.8E-02 |
| Hs.476636 | hypothetical protein LOC151878 | LOC151878 | 2.297 | 5.0E-02 |
| --- | --- | --- | 2.296 | 2.0E-03 |
| Hs.461819 | Hypothetical protein FLJ10979 | FLJ10979 | 2.295 | 3.2E-02 |
| --- | --- | --- | 2.293 | 7.5E-03 |
| Hs.472031 | ubiquitin-conjugating enzyme E2D 3 (UBC4/5 homolog, yeast) | UBE2D3 | 2.293 | 4.6E-03 |
| Hs.522730 | G protein-coupled receptor associated sorting protein 1 | GPRASP1 | 2.291 | 2.0E-03 |
| Hs.535012 | Hypothetical gene supported by AF131741 | --- | 2.290 | 1.2E-02 |
| Hs.466589 | Zinc finger protein 585B | ZNF585B | 2.286 | 3.9E-02 |
| Hs.462400 | Similar to Envoplakin (210 kDa paraneoplastic pemphigus antigen) (p210) (210 kDa cornified envelope precursor) | --- | 2.285 | 4.5E-02 |
| Hs.181973 | cytochrome P450, family 2, subfamily A, polypeptide 13 | CYP2A13 | 2.285 | 1.7E-02 |
| Hs.191475 | Hypothetical protein LOC149401 | LOC149401 | 2.284 | 2.4E-03 |
| Hs.351316 | transmembrane 4 L six family member 1 | TM4SF1 | 2.283 | 3.8E-03 |
| Hs.294147 | chromosome 9 open reading frame 111 | C9orf111 | 2.279 | 2.3E-02 |
| Hs.72885 | azurocidin 1 (cationic antimicrobial protein 37) | AZU1 | 2.278 | 4.8E-02 |
| Hs.87225 | cancer/testis antigen 2 | CTAG2 | 2.275 | 3.9E-04 |
| --- | --- | --- | 2.273 | 3.9E-02 |
| Hs.177841 | basic helix-loop-helix domain containing, class B, 3 | BHLHB3 | 2.269 | 4.6E-02 |
| Hs.287827 | ATP-binding cassette, sub-family B (MDR/TAP), member 4 | ABCB4 | 2.269 | 1.6E-02 |
| Hs.385569 | Homo sapiens, clone IMAGE:4828937, mRNA | --- | 2.267 | 5.4E-03 |
| --- | --- | --- | 2.267 | 1.7E-04 |
| Hs.444947 | tribbles homolog 1 (Drosophila) | TRIB1 | 2.265 | 1.0E-02 |
| Hs.523789 | trophoblast-derived noncoding RNA | TncRNA | 2.265 | 1.6E-04 |
| Hs.303653 | hypothetical protein MGC13053 /// hypothetical protein MGC13053 | MGC13053 | 2.265 | 4.5E-03 |
| Hs.324335 | chromosome 19 open reading frame 15 | C19orf15 | 2.263 | 4.0E-03 |
| Hs.171825 | basic helix-loop-helix domain containing, class B, 2 | BHLHB2 | 2.261 | 4.0E-02 |
| Hs.78854 | ATPase, Na+/K+ transporting, beta 2 polypeptide | ATP1B2 | 2.260 | 6.7E-03 |
| Hs.501080 | Transcription factor 7-like 2 (T-cell specific, HMG-box) | TCF7L2 | 2.259 | 2.1E-02 |
| --- | --- | --- | 2.258 | 6.4E-03 |
| --- | protocadherin gamma subfamily A, 12 /// protocadherin gamma subfamily A, 11 /// protocadherin gamma subfamily A, 10 /// protocadherin gamma subfamily A, 6 /// protocadherin gamma subfamily A, 5 /// protocadherin gamma subfamily A, 3 | PCDHGA12 /// PCDHGA11 /// PCDHGA10 /// PCDHGA6 /// PCDHGA5 /// PCDHGA3 | 2.255 | 3.2E-02 |
| Hs.247816 | histone 1, H4f | HIST1H4F | 2.254 | 1.1E-04 |
| Hs.496753 | Similar to hypothetical protein | --- | 2.253 | 3.4E-02 |
| Hs.24178 | echinoderm microtubule associated protein like 2 | EML2 | 2.252 | 3.2E-02 |
| Hs.2780 | jun D proto-oncogene | JUND | 2.252 | 4.4E-02 |
| --- | --- | --- | 2.251 | 1.5E-02 |
| Hs.250281 | Solute carrier family 13 (sodium-dependent dicarboxylate transporter), member 3 | SLC13A3 | 2.251 | 9.3E-03 |
| --- | --- | --- | 2.250 | 1.5E-04 |
| Hs.144372 | Nuclear localized factor 2 | NLF2 | 2.250 | 1.8E-02 |
| Hs.21213 | myosin VA (heavy polypeptide 12, myoxin) | MYO5A | 2.249 | 3.6E-02 |
| Hs.129169 | Transcribed locus | --- | 2.246 | 4.7E-04 |
| Hs.99960 | membrane-spanning 4-domains, subfamily A, member 3 (hematopoietic cell-specific) | MS4A3 | 2.244 | 3.3E-02 |
| Hs.143713 | Transcribed locus, weakly similar to XP_524313.1 LOC468928 [Pan troglodytes] | --- | 2.244 | 7.9E-03 |
| Hs.153934 | core-binding factor, runt domain, alpha subunit 2; translocated to, 2 | CBFA2T2 | 2.244 | 4.4E-02 |
| Hs.270291 | Actinin, alpha 4 | ACTN4 | 2.243 | 8.9E-03 |
| Hs.511265 | sema domain, transmembrane domain (TM), and cytoplasmic domain, (semaphorin) 6D | SEMA6D | 2.241 | 4.6E-03 |
| Hs.435071 | Transcribed locus | --- | 2.241 | 7.4E-05 |
| Hs.82071 | Cbp/p300-interacting transactivator, with Glu/Asp-rich carboxy-terminal domain, 2 | CITED2 | 2.238 | 1.5E-03 |
| Hs.432453 | mitogen-activated protein kinase kinase kinase 8 | MAP3K8 | 2.238 | 2.1E-03 |
| Hs.37982 | Neural precursor cell expressed, developmentally down-regulated 9 | NEDD9 | 2.238 | 3.1E-03 |
| Hs.440588 | Transcribed locus | --- | 2.236 | 4.0E-02 |
| Hs.196484 | OTTHUMP00000013724 | FLJ22588 | 2.235 | 2.7E-03 |
| Hs.491223 | retinoic acid induced 16 | RAI16 | 2.235 | 2.9E-03 |
| Hs.247565 | rhodopsin (opsin 2, rod pigment) (retinitis pigmentosa 4, autosomal dominant) | RHO | 2.235 | 3.3E-03 |
| Hs.136164 | TSPY-like 2 | TSPYL2 | 2.234 | 1.6E-02 |
| Hs.302123 | Z-DNA binding protein 1 /// Z-DNA binding protein 1 | ZBP1 | 2.234 | 4.1E-02 |
| Hs.368226 | SRY (sex determining region Y)-box 6 | SOX6 | 2.233 | 3.5E-02 |
| Hs.47115 | hydrocephalus inducing | HYDIN | 2.232 | 3.2E-02 |
| --- | --- | --- | 2.229 | 6.4E-03 |
| Hs.253576 | chromosome 10 open reading frame 80 | C10orf80 | 2.229 | 1.6E-03 |
| Hs.515465 | apolipoprotein E | APOE | 2.228 | 7.5E-03 |
| Hs.170298 | Hypothetical protein FLJ13236 | FLJ13236 | 2.225 | 9.2E-03 |
| --- | --- | --- | 2.225 | 9.9E-03 |
| Hs.127126 | Cytoplasmic polyadenylation element binding protein 4 | CPEB4 | 2.224 | 1.8E-03 |
| --- | --- | --- | 2.224 | 2.9E-02 |
| Hs.436854 | family with sequence similarity 19 (chemokine (C-C motif)-like), member A5 | FAM19A5 | 2.224 | 3.0E-02 |
| Hs.98587 | CDNA clone IMAGE:4557810, partial cds | --- | 2.224 | 3.0E-02 |
| Hs.76716 | inter-alpha (globulin) inhibitor H3 | ITIH3 | 2.221 | 2.4E-02 |
| Hs.117545 | phosphodiesterase 4D, cAMP-specific (phosphodiesterase E3 dunce homolog, Drosophila) | PDE4D | 2.220 | 3.3E-02 |
| Hs.248139 | potassium voltage-gated channel, shaker-related subfamily, member 2 | KCNA2 | 2.219 | 8.0E-03 |
| Hs.250493 | Zinc finger protein 219 | ZNF219 | 2.219 | 1.6E-02 |
| --- | --- | --- | 2.217 | 1.0E-02 |
| Hs.16355 | Myosin, heavy polypeptide 10, non-muscle | MYH10 | 2.216 | 2.6E-02 |
| Hs.515247 | Janus kinase 3 (a protein tyrosine kinase, leukocyte) | JAK3 | 2.216 | 1.3E-02 |
| Hs.155569 | hypothetical protein MGC4504 | MGC4504 | 2.216 | 7.2E-04 |
| Hs.172928 | collagen, type I, alpha 1 | COL1A1 | 2.214 | 2.1E-03 |
| Hs.518773 | Ubiquitin-conjugating enzyme E2D 3 (UBC4/5 homolog, yeast) | UBE2D3 | 2.211 | 3.6E-03 |
| Hs.368281 | microtubule-associated protein 2 | MAP2 | 2.211 | 4.7E-03 |
| Hs.458973 | zinc finger homeodomain 4 | ZFHX4 | 2.205 | 4.4E-02 |
| --- | --- | --- | 2.203 | 3.8E-02 |
| Hs.434900 | PDZ domain containing RING finger 3 | PDZRN3 | 2.203 | 1.2E-02 |
| Hs.149168 | tumor necrosis factor receptor superfamily, member 19 | TNFRSF19 | 2.202 | 2.6E-02 |
| Hs.283148 | RAB38, member RAS oncogene family | RAB38 | 2.200 | 5.5E-03 |
| Hs.491856 | hypothetical protein FLJ39630 | FLJ39630 | 2.200 | 4.9E-02 |
| Hs.274605 | FLJ41649 protein | FLJ41649 | 2.199 | 3.5E-02 |
| Hs.208274 | Transcribed locus | --- | 2.197 | 1.5E-03 |
| Hs.248140 | potassium voltage-gated channel, shaker-related subfamily, member 10 | KCNA10 | 2.196 | 4.1E-03 |
| Hs.46772 | CDNA FLJ35878 fis, clone TESTI2008567 | --- | 2.195 | 2.8E-02 |
| Hs.509554 | Hypoxia-inducible factor 1, alpha subunit (basic helix-loop-helix transcription factor) | HIF1A | 2.195 | 5.8E-04 |
| Hs.158529 | calsyntenin 2 | CLSTN2 | 2.191 | 3.1E-03 |
| Hs.409523 | lymphocyte-activation gene 3 | LAG3 | 2.190 | 4.1E-02 |
| Hs.325838 | CTD-binding SR-like protein rA9 | KIAA1542 | 2.189 | 2.0E-02 |
| Hs.5940 | mucin 13, epithelial transmembrane | MUC13 | 2.188 | 4.4E-02 |
| Hs.205173 | Preimplantation protein 3 | PREI3 | 2.188 | 2.4E-03 |
| Hs.301016 | CDNA FLJ13060 fis, clone NT2RP3001607 | --- | 2.185 | 4.8E-02 |
| Hs.201340 | Sema domain, immunoglobulin domain (Ig), short basic domain, secreted, (semaphorin) 3D | SEMA3D | 2.184 | 2.7E-02 |
| Hs.374613 | Inositol 1,4,5-triphosphate receptor, type 1 | ITPR1 | 2.184 | 4.3E-02 |
| Hs.124519 | hypothetical protein FLJ20449 | FLJ20449 | 2.184 | 3.9E-02 |
| Hs.429819 | Phosphatidylinositol transfer protein, alpha | PITPNA | 2.183 | 5.4E-04 |
| Hs.49768 | Transcribed locus | --- | 2.183 | 4.5E-02 |
| Hs.193825 | Protein phosphatase 2 (formerly 2A), regulatory subunit B (PR 52), beta isoform | PPP2R2B | 2.182 | 1.9E-02 |
| Hs.278036 | LOC442512 | LOC442512 | 2.179 | 1.5E-02 |
| Hs.510324 | Quaking homolog, KH domain RNA binding (mouse) | QKI | 2.178 | 2.9E-02 |
| Hs.419240 | Solute carrier family 2 (facilitated glucose transporter), member 3 | SLC2A3 | 2.178 | 3.5E-04 |
| Hs.194121 | RNA terminal phosphate cyclase-like 1 | RCL1 | 2.177 | 4.7E-02 |
| Hs.22815 | CDNA FLJ14143 fis, clone MAMMA1002892 | --- | 2.177 | 5.1E-03 |
| Hs.121536 | family with sequence similarity 54, member A | FAM54A | 2.175 | 3.6E-02 |
| Hs.439631 | KIAA2018 | KIAA2018 | 2.175 | 3.9E-02 |
| Hs.187898 | Neurofibromin 2 (bilateral acoustic neuroma) | NF2 | 2.175 | 6.0E-03 |
| Hs.210283 | Collagen, type V, alpha 1 | COL5A1 | 2.174 | 4.8E-02 |
| Hs.495897 | astrotactin | ASTN | 2.174 | 4.4E-02 |
| Hs.137183 | Down syndrome cell adhesion molecule like 1 | DSCAML1 | 2.173 | 2.8E-02 |
| Hs.313471 | KIAA1683 | KIAA1683 | 2.171 | 4.1E-02 |
| Hs.376894 | similar to Serine/threonine-protein kinase PRKX (Protein kinase PKX1) | LOC441733 | 2.169 | 1.1E-02 |
| Hs.20395 | chromodomain helicase DNA binding protein 7 | CHD7 | 2.169 | 3.1E-02 |
| Hs.449942 | inositol polyphosphate-5-phosphatase, 75kDa | INPP5B | 2.168 | 4.5E-02 |
| Hs.500468 | growth hormone 1 | GH1 | 2.166 | 4.6E-02 |
| Hs.175955 | Splicing factor YT521-B | YT521 | 2.165 | 4.8E-03 |
| Hs.408155 | Homo sapiens, clone IMAGE:4694422, mRNA | --- | 2.164 | 4.0E-02 |
| Hs.117688 | Transcribed locus | --- | 2.164 | 1.0E-03 |
| Hs.284707 | Homo sapiens, clone IMAGE:5259731, mRNA | --- | 2.160 | 5.0E-02 |
| Hs.278483 | histone 1, H4k /// histone 1, H4j | HIST1H4K /// HIST1H4J | 2.160 | 6.8E-03 |
| Hs.550478 | heparan sulfate proteoglycan 2 (perlecan) | HSPG2 | 2.160 | 4.4E-02 |
| Hs.21417 | zinc finger, CCHC domain containing 12 | ZCCHC12 | 2.158 | 1.0E-02 |
| Hs.546441 | Polyhomeotic like 3 (Drosophila) | PHC3 | 2.157 | 4.8E-02 |
| --- | --- | --- | 2.157 | 2.1E-02 |
| Hs.352240 | Hypothetical protein MGC15523 | MGC15523 | 2.157 | 2.0E-03 |
| Hs.412421 | Hypothetical protein MGC13168 | MGC13168 | 2.155 | 1.8E-02 |
| Hs.524688 | Hippocalcin like 4 | HPCAL4 | 2.152 | 2.9E-02 |
| Hs.347991 | Nuclear receptor subfamily 2, group F, member 2 | NR2F2 | 2.150 | 4.3E-03 |
| Hs.30332 | Glutamine-fructose-6-phosphate transaminase 2 | GFPT2 | 2.149 | 2.1E-04 |
| Hs.373571 | CDNA FLJ39665 fis, clone SMINT2007294 | --- | 2.149 | 4.6E-02 |
| Hs.525056 | FLJ40296 protein | FLJ40296 | 2.148 | 2.8E-02 |
| Hs.494567 | hyaluronan binding protein 4 | HABP4 | 2.147 | 3.6E-02 |
| Hs.153692 | monogenic, audiogenic seizure susceptibility 1 homolog (mouse) | MASS1 | 2.147 | 3.3E-02 |
| Hs.1219 | alcohol dehydrogenase 4 (class II), pi polypeptide | ADH4 | 2.146 | 4.3E-02 |
| Hs.552605 | chromosome 20 open reading frame 128 | C20orf128 | 2.145 | 5.3E-03 |
| Hs.65756 | regulator of G-protein signalling 11 | RGS11 | 2.145 | 5.2E-03 |
| Hs.131824 | hypothetical protein LOC126536 | LOC126536 | 2.144 | 2.3E-02 |
| Hs.413297 | regulator of G-protein signalling 16 | RGS16 | 2.142 | 2.3E-02 |
| Hs.386283 | a disintegrin and metalloproteinase domain 12 (meltrin alpha) | ADAM12 | 2.142 | 4.2E-02 |
| Hs.435001 | Kruppel-like factor 10 | KLF10 | 2.142 | 3.2E-04 |
| Hs.109590 | Genethonin 1 | GENX-3414 | 2.142 | 3.8E-02 |
| Hs.278483 | histone 1, H4k /// histone 1, H4j | HIST1H4K /// HIST1H4J | 2.139 | 6.6E-03 |
| Hs.95612 | desmocollin 2 | DSC2 | 2.139 | 2.0E-02 |
| Hs.406550 | IgH chain VDJH region | --- | 2.137 | 5.3E-04 |
| Hs.434618 | cyclic nucleotide gated channel alpha 4 | CNGA4 | 2.137 | 3.3E-03 |
| Hs.78036 | solute carrier family 6 (neurotransmitter transporter, noradrenalin), member 2 | SLC6A2 | 2.136 | 3.3E-02 |
| Hs.433702 | Eukaryotic translation initiation factor 5 | EIF5 | 2.135 | 3.2E-05 |
| Hs.322444 | Homo sapiens, clone IMAGE:3604069, mRNA | --- | 2.135 | 3.4E-02 |
| Hs.544835 | olfactory receptor, family 9, subfamily A, member 1, pseudogene | OR9A1P | 2.134 | 1.6E-02 |
| Hs.525287 | Rho GTPase activating protein 5 | ARHGAP5 | 2.133 | 1.3E-03 |
| Hs.198003 | sarcosine dehydrogenase | SARDH | 2.132 | 2.0E-02 |
| Hs.131673 | glucocorticoid induced transcript 1 | GLCCI1 | 2.132 | 2.9E-02 |
| Hs.509523 | Menage a trois 1 (CAK assembly factor) | MNAT1 | 2.130 | 2.3E-02 |
| Hs.519601 | Inhibitor of DNA binding 4, dominant negative helix-loop-helix protein | ID4 | 2.130 | 2.6E-02 |
| Hs.532144 | histone 1, H3d | HIST1H3D | 2.129 | 3.2E-04 |
| Hs.532655 | ephrin-A2 | EFNA2 | 2.129 | 4.1E-02 |
| Hs.412355 | growth differentiation factor 1 /// LAG1 longevity assurance homolog 1 (S. cerevisiae) | GDF1 /// LASS1 | 2.128 | 4.9E-02 |
| Hs.169358 | Hypothetical protein DJ971N18.2 | DJ971N18.2 | 2.128 | 1.9E-04 |
| Hs.434401 | Zinc finger protein 638 | ZNF638 | 2.127 | 1.8E-03 |
| Hs.143545 | solute carrier family 30 (zinc transporter), member 2 | SLC30A2 | 2.126 | 2.4E-02 |
| Hs.205439 | CDNA clone IMAGE:3958634, partial cds | --- | 2.126 | 1.6E-03 |
| Hs.520973 | heat shock 27kDa protein 1 | HSPB1 | 2.125 | 8.1E-03 |
| Hs.547470 | Transcribed locus | --- | 2.124 | 1.1E-03 |
| Hs.306834 | FK506 binding protein 1B, 12.6 kDa | FKBP1B | 2.119 | 3.6E-03 |
| Hs.438 | mesenchyme homeo box 1 | MEOX1 | 2.119 | 2.5E-02 |
| Hs.156369 | tenascin N | TNN | 2.118 | 3.2E-02 |
| Hs.271903 | amyotrophic lateral sclerosis 2 (juvenile) chromosome region, candidate 19 | ALS2CR19 | 2.117 | 3.9E-02 |
| Hs.513555 | spermatogenesis associated 1 | SPATA1 | 2.117 | 3.6E-02 |
| Hs.155488 | hypothetical protein FLJ39743 | FLJ39743 | 2.116 | 3.3E-02 |
| Hs.546880 | hypothetical protein LOC283901 | LOC283901 | 2.115 | 1.8E-02 |
| Hs.119298 | Homo sapiens, clone IMAGE:3839141, mRNA | --- | 2.115 | 1.0E-02 |
| Hs.134816 | Zinc finger protein 305 | ZNF305 | 2.111 | 3.3E-02 |
| Hs.550464 | ATPase, Ca++ transporting, cardiac muscle, fast twitch 1 | ATP2A1 | 2.109 | 1.9E-02 |
| Hs.527348 | A kinase (PRKA) anchor protein (yotiao) 9 | AKAP9 | 2.108 | 9.8E-03 |
| Hs.495656 | transducin (beta)-like 1X-linked | TBL1X | 2.107 | 3.9E-02 |
| Hs.155048 | Lutheran blood group (Auberger b antigen included) | LU | 2.106 | 3.3E-02 |
| Hs.523873 | nuclear mitotic apparatus protein 1 | NUMA1 | 2.105 | 5.5E-03 |
| Hs.550659 | similar to tripartite motif-containing 4 | LOC493829 | 2.104 | 4.1E-02 |
| Hs.488130 | Transcribed locus, weakly similar to NP_061913.2 elongation protein 4 homolog (S. cerevisiae) [Homo sapiens] | --- | 2.102 | 2.6E-04 |
| Hs.389103 | glucagon-like peptide 1 receptor | GLP1R | 2.099 | 1.3E-04 |
| Hs.126706 | 1-aminocyclopropane-1-carboxylate synthase | PHACS | 2.099 | 3.2E-02 |
| Hs.283961 | guanine nucleotide binding protein (G protein), gamma 8 | GNG8 | 2.099 | 2.7E-02 |
| Hs.533659 | synaptopodin 2-like | SYNPO2L | 2.097 | 1.7E-02 |
| --- | --- | --- | 2.096 | 4.3E-02 |
| Hs.434251 | KIAA0690 | KIAA0690 | 2.095 | 8.4E-03 |
| Hs.517617 | v-maf musculoaponeurotic fibrosarcoma oncogene homolog F (avian) | MAFF | 2.091 | 1.8E-03 |
| Hs.2363 | potassium inwardly-rectifying channel, subfamily J, member 12 | KCNJ12 | 2.091 | 2.1E-02 |
| Hs.385790 | chromosome 9 open reading frame 107 | C9orf107 | 2.090 | 4.0E-02 |
| Hs.211461 | CDNA FLJ39613 fis, clone SKNSH2009357 | --- | 2.089 | 2.1E-02 |
| Hs.125571 | transmembrane protease, serine 12 | TMPRSS12 | 2.089 | 4.2E-02 |
| Hs.453951 | neuregulin 1 | NRG1 | 2.088 | 4.3E-02 |
| Hs.252351 | HERV-H LTR-associating 2 | HHLA2 | 2.087 | 3.9E-02 |
| Hs.1837 | POU domain, class 3, transcription factor 1 | POU3F1 | 2.087 | 1.7E-04 |
| Hs.141308 | myelin oligodendrocyte glycoprotein | MOG | 2.087 | 1.2E-02 |
| Hs.518438 | SRY (sex determining region Y)-box 2 | SOX2 | 2.086 | 2.0E-02 |
| Hs.132001 | ST3 beta-galactoside alpha-2,3-sialyltransferase 3 | ST3GAL3 | 2.081 | 1.1E-03 |
| Hs.508234 | Kruppel-like factor 5 (intestinal) | KLF5 | 2.078 | 1.7E-03 |
| Hs.414300 | sialidase 4 | NEU4 | 2.078 | 1.9E-02 |
| Hs.503178 | Spectrin, beta, non-erythrocytic 1 | SPTBN1 | 2.077 | 3.2E-02 |
| Hs.157378 | Ankyrin repeat and MYND domain containing 2 | ANKMY2 | 2.076 | 2.4E-04 |
| Hs.435052 | ATPase, aminophospholipid transporter (APLT), Class I, type 8A, member 1 | ATP8A1 | 2.075 | 1.9E-02 |
| Hs.269180 | erythrocyte membrane protein band 4.1 like 4B | EPB41L4B | 2.070 | 3.6E-02 |
| Hs.507027 | CDNA FLJ41016 fis, clone UTERU2018784 | --- | 2.069 | 1.7E-02 |
| Hs.157145 | Tetracycline transporter-like protein | TETRAN | 2.068 | 2.8E-04 |
| Hs.530461 | histone 2, H2aa | HIST2H2AA | 2.067 | 6.7E-04 |
| Hs.450763 | SLIT-ROBO Rho GTPase activating protein 1 | SRGAP1 | 2.064 | 4.3E-02 |
| Hs.19699 | Chromosome 16 open reading frame 35 | C16orf35 | 2.062 | 3.2E-02 |
| Hs.475812 | Source of immunodominant MHC-associated peptides | SIMP | 2.061 | 4.7E-03 |
| Hs.524430 | nuclear receptor subfamily 4, group A, member 1 | NR4A1 | 2.061 | 2.2E-03 |
| Hs.370984 | immunoglobulin superfamily, member 4C | IGSF4C | 2.061 | 3.8E-03 |
| --- | --- | --- | 2.059 | 2.0E-02 |
| Hs.471156 | Abl interactor 2 | ABI2 | 2.059 | 2.3E-02 |
| Hs.203717 | fibronectin 1 | FN1 | 2.059 | 2.3E-02 |
| Hs.533122 | splicing factor, arginine/serine-rich 10 (transformer 2 homolog, Drosophila) | SFRS10 | 2.058 | 9.7E-03 |
| Hs.278945 | zinc finger, CCHC domain containing 4 | ZCCHC4 | 2.057 | 9.3E-03 |
| Hs.266308 | transmembrane protease, serine 13 | TMPRSS13 | 2.057 | 1.1E-02 |
| --- | --- | --- | 2.053 | 8.0E-04 |
| --- | --- | --- | 2.053 | 1.5E-02 |
| Hs.499984 | sphingosine-1-phosphate lyase 1 | SGPL1 | 2.053 | 3.8E-02 |
| --- | --- | --- | 2.052 | 6.8E-04 |
| Hs.489615 | Pre-B-cell colony enhancing factor 1 | PBEF1 | 2.049 | 7.4E-04 |
| Hs.388622 | zinc finger protein, subfamily 1A, 4 (Eos) | ZNFN1A4 | 2.047 | 9.5E-05 |
| Hs.127043 | Homo sapiens, clone IMAGE:4819956, mRNA, partial cds | --- | 2.047 | 3.5E-02 |
| Hs.152812 | Transcribed locus | --- | 2.046 | 1.5E-02 |
| Hs.424414 | msh homeo box homolog 1 (Drosophila) | MSX1 | 2.045 | 2.4E-05 |
| Hs.518727 | RNA-binding protein | FLJ20273 | 2.044 | 3.2E-02 |
| --- | --- | --- | 2.044 | 3.4E-03 |
| Hs.552876 | CDNA FLJ37868 fis, clone BRSSN2017297 | --- | 2.043 | 4.2E-02 |
| Hs.283023 | G-protein coupled receptor 173 | GPR173 | 2.042 | 4.5E-03 |
| Hs.498519 | Homo sapiens, clone IMAGE:5261280, mRNA | --- | 2.042 | 2.2E-05 |
| Hs.455323 | aquaporin 7 | AQP7 | 2.041 | 4.7E-02 |
| Hs.462257 | Myocardin | MYOCD | 2.041 | 8.4E-04 |
| Hs.523789 | trophoblast-derived noncoding RNA | TncRNA | 2.040 | 4.5E-02 |
| Hs.519294 | fibrillin 2 (congenital contractural arachnodactyly) | FBN2 | 2.040 | 6.4E-04 |
| Hs.299127 | potassium channel tetramerisation domain containing 19 | KCTD19 | 2.039 | 4.3E-02 |
| --- | --- | --- | 2.039 | 2.4E-02 |
| Hs.137367 | Ankyrin 2, neuronal | ANK2 | 2.039 | 4.2E-02 |
| Hs.124673 | chromosome 18 open reading frame 4 | C18orf4 | 2.039 | 1.5E-02 |
| Hs.16732 | Transcribed locus | --- | 2.039 | 6.0E-03 |
| Hs.518727 | RNA-binding protein | FLJ20273 | 2.038 | 7.3E-03 |
| Hs.253305 | soluble liver antigen/liver pancreas antigen | SLA/LP | 2.037 | 4.3E-02 |
| Hs.23671 | lectin, galactoside-binding, soluble, 13 (galectin 13) | LGALS13 | 2.037 | 3.3E-03 |
| Hs.143591 | KIAA0789 gene product | KIAA0789 | 2.037 | 2.1E-02 |
| Hs.503997 | Transcribed locus, moderately similar to XP_512541.1 similar to hypothetical protein [Pan troglodytes] | --- | 2.036 | 8.5E-04 |
| Hs.434752 | Homo sapiens, clone IMAGE:5164889, mRNA | --- | 2.035 | 2.4E-02 |
| Hs.310333 | sialic acid binding Ig-like lectin 5 /// sialic acid binding Ig-like lectin 5 | SIGLEC5 | 2.032 | 2.0E-02 |
| Hs.545134 | period 4 pseudogene | PER4 | 2.030 | 1.7E-02 |
| Hs.125898 | GNAS complex locus | GNAS | 2.029 | 1.1E-03 |
| Hs.187866 | Stromal cell derived factor receptor 1 | SDFR1 | 2.028 | 1.9E-05 |
| Hs.279472 | Transcribed locus | --- | 2.027 | 1.7E-02 |
| Hs.259605 | Phosphatidylinositol glycan, class V | PIGV | 2.026 | 1.5E-02 |
| Hs.374180 | Secretory carrier membrane protein 5 | SCAMP5 | 2.025 | 9.3E-03 |
| Hs.484950 | histone 1, H2ac | HIST1H2AC | 2.024 | 4.7E-02 |
| Hs.533317 | vimentin | VIM | 2.024 | 5.4E-03 |
| --- | dynein, axonemal, heavy polypeptide 1 | DNAH1 | 2.023 | 2.0E-03 |
| Hs.440981 | CDNA clone IMAGE:5272084, partial cds | --- | 2.023 | 4.9E-02 |
| Hs.539003 | Trapped 3' terminal exon, clone C2B5 | --- | 2.022 | 3.7E-05 |
| Hs.317593 | Chromosome 13 open reading frame 11 | C13orf11 | 2.019 | 4.3E-02 |
| --- | --- | --- | 2.019 | 2.4E-02 |
| Hs.170600 | GRB2-related adaptor protein-like | LOC400581 | 2.016 | 1.4E-03 |
| Hs.348365 | CARD only protein | COPl | 2.016 | 4.8E-02 |
| Hs.504895 | Serine/threonine kinase receptor associated protein | STRAP | 2.015 | 5.8E-03 |
| Hs.72981 | neurogenic differentiation 1 | NEUROD1 | 2.015 | 2.9E-02 |
| --- | --- | --- | 2.014 | 5.5E-04 |
| Hs.544913 | Full length insert cDNA clone YR42A07 | --- | 2.014 | 1.2E-02 |
| Hs.197082 | Transcribed locus | --- | 2.014 | 2.7E-02 |
| Hs.549350 | similar to C10orf94 protein | LOC400547 | 2.013 | 2.7E-03 |
| --- | histone 1, H2bi | HIST1H2BI | 2.010 | 2.7E-02 |
| Hs.405755 | dihydrodiol dehydrogenase (dimeric) | DHDH | 2.010 | 3.5E-02 |
| Hs.233283 | CDNA FLJ36254 fis, clone THYMU2002157 | --- | 2.009 | 3.7E-02 |
| Hs.531644 | CDNA FLJ40285 fis, clone TESTI2027820 | --- | 2.008 | 1.3E-02 |
| Hs.411402 | Homo sapiens, clone IMAGE:4469683, mRNA | --- | 2.007 | 4.5E-02 |
| Hs.524280 | H2A histone family, member J | H2AFJ | 2.004 | 7.9E-04 |
| Hs.118366 | CDNA clone IMAGE:6201773 | --- | 2.003 | 2.3E-02 |
| Hs.370510 | immunoglobulin superfamily, member 4 | IGSF4 | 2.001 | 9.4E-03 |
| Hs.20013 | GCIP-interacting protein p29 | P29 | 2.000 | 1.2E-03 |
|  |  |  |  |  |
| Hs.466148 | nuclear receptor subfamily 2, group F, member 6 | NR2F6 | -2.004 | 4.7E-02 |
| Hs.326822 | Ras and Rab interactor 3 | RIN3 | -2.008 | 2.8E-02 |
| Hs.377010 | Carbamoyl-phosphate synthetase 2, aspartate transcarbamylase, and dihydroorotase | CAD | -2.017 | 2.4E-02 |
| Hs.517356 | Collagen, type XVIII, alpha 1 | COL18A1 | -2.017 | 3.3E-02 |
| Hs.464442 | chromosome 18 open reading frame 2 | C18orf2 | -2.018 | 4.8E-02 |
| Hs.502338 | solute carrier family 1 (glial high affinity glutamate transporter), member 2 | SLC1A2 | -2.022 | 3.6E-02 |
| Hs.112160 | chromosome 15 open reading frame 20 | C15orf20 | -2.025 | 3.6E-02 |
| Hs.485892 | Zinc finger protein 292 | ZNF292 | -2.029 | 1.7E-02 |
| Hs.181112 | mediator of RNA polymerase II transcription, subunit 4 homolog (yeast) | MED4 | -2.031 | 9.8E-04 |
| Hs.8859 | calcium activated nucleotidase 1 | CANT1 | -2.033 | 2.9E-02 |
| Hs.530543 | CDNA FLJ41452 fis, clone BRSTN2010363 | --- | -2.033 | 6.4E-03 |
| Hs.534828 | Similar to interspersed repeat antigen, putative | --- | -2.045 | 4.2E-03 |
| Hs.534574 | zinc finger protein 564 | ZNF564 | -2.050 | 1.7E-04 |
| Hs.145956 | zinc finger protein 226 | ZNF226 | -2.051 | 2.0E-05 |
| Hs.376474 | hypothetical protein LOC286436 | LOC286436 | -2.053 | 2.9E-03 |
| Hs.335163 | KIAA1102 protein | KIAA1102 | -2.067 | 3.3E-03 |
| Hs.534534 | Smith-Magenis syndrome chromosome region, candidate 7 | SMCR7 | -2.069 | 3.2E-02 |
| Hs.445061 | Vac14 homolog (S. cerevisiae) | VAC14 | -2.072 | 2.8E-02 |
| Hs.231829 | glutamate decarboxylase 2 (pancreatic islets and brain, 65kDa) | GAD2 | -2.077 | 2.1E-02 |
| Hs.391480 | hypothetical protein PRO2949 | PRO2949 | -2.077 | 2.8E-03 |
| Hs.10101 | hypothetical protein FLJ12875 | FLJ12875 | -2.101 | 2.2E-02 |
| Hs.274408 | CASK interacting protein 2 | CASKIN2 | -2.106 | 8.8E-03 |
| Hs.176376 | Transcribed locus | --- | -2.114 | 2.1E-02 |
| Hs.153692 | monogenic, audiogenic seizure susceptibility 1 homolog (mouse) | MASS1 | -2.117 | 1.1E-02 |
| Hs.476130 | coiled-coil domain containing 12 | CCDC12 | -2.121 | 3.9E-04 |
| Hs.352156 | A disintegrin-like and metalloprotease (reprolysin type) with thrombospondin type 1 motif, 14 | ADAMTS14 | -2.121 | 8.0E-04 |
| Hs.129115 | Transcribed locus | --- | -2.123 | 4.8E-02 |
| Hs.462492 | ubiquitin specific protease 22 | USP22 | -2.126 | 2.4E-02 |
| Hs.142736 | hypothetical protein LOC144233 | LOC144233 | -2.127 | 6.4E-03 |
| Hs.393201 | ARP2 actin-related protein 2 homolog (yeast) | ACTR2 | -2.128 | 3.5E-03 |
| Hs.143818 | gem (nuclear organelle) associated protein 6 | GEMIN6 | -2.136 | 8.1E-06 |
| Hs.132591 | solute carrier family 10 (sodium/bile acid cotransporter family), member 4 | SLC10A4 | -2.149 | 1.3E-02 |
| Hs.513440 | G protein-coupled receptor 65 | GPR65 | -2.151 | 2.5E-03 |
| Hs.138701 | T cell receptor interacting molecule | TCRIM | -2.159 | 4.8E-03 |
| Hs.314141 | matrix metalloproteinase 21 | MMP21 | -2.164 | 1.1E-02 |
| Hs.36859 | WD repeat domain 20 | WDR20 | -2.175 | 2.0E-02 |
| Hs.516651 | hypothetical protein FLJ25415 | FLJ25415 | -2.178 | 1.1E-02 |
| Hs.522351 | KIAA0674 | KIAA0674 | -2.178 | 4.8E-02 |
| --- | --- | --- | -2.180 | 3.8E-03 |
| Hs.157101 | hypothetical gene supported by AK092138 | LOC400690 | -2.189 | 2.8E-02 |
| --- | --- | --- | -2.189 | 4.6E-02 |
| Hs.86131 | Fas (TNFRSF6)-associated via death domain | FADD | -2.199 | 6.9E-04 |
| --- | --- | --- | -2.210 | 3.1E-04 |
| Hs.529680 | hypothetical protein FLJ25770 | FLJ25770 | -2.215 | 3.7E-03 |
| Hs.128959 | Pre-mRNA cleavage complex II protein Pcf11 | PCF11 | -2.225 | 4.7E-02 |
| Hs.387405 | Transcribed locus | --- | -2.226 | 5.6E-03 |
| Hs.270435 | Hypothetical protein FLJ12985 | FLJ12985 | -2.226 | 7.0E-04 |
| Hs.509083 | A kinase (PRKA) anchor protein 6 | AKAP6 | -2.227 | 1.7E-03 |
| Hs.435512 | Protein phosphatase 3 (formerly 2B), catalytic subunit, alpha isoform (calcineurin A alpha) | PPP3CA | -2.229 | 8.5E-03 |
| Hs.370267 | tankyrase, TRF1-interacting ankyrin-related ADP-ribose polymerase | TNKS | -2.234 | 2.3E-02 |
| Hs.517094 | neuralized-like 2 (Drosophila) | NEURL2 | -2.236 | 3.1E-03 |
| Hs.507475 | replication factor C (activator 1) 1, 145kDa | RFC1 | -2.236 | 2.5E-02 |
| Hs.279914 | Zinc finger protein 232 | ZNF232 | -2.238 | 3.0E-02 |
| Hs.516904 | brevican | BCAN | -2.244 | 1.2E-03 |
| --- | --- | --- | -2.251 | 1.7E-02 |
| Hs.371889 | ATPase, Na+/K+ transporting, alpha 1 polypeptide | ATP1A1 | -2.257 | 1.4E-03 |
| Hs.87464 | Sex comb on midleg homolog 1 (Drosophila) | SCMH1 | -2.268 | 2.6E-02 |
| Hs.283658 | proline-rich protein BstNI subfamily 1 /// proline-rich protein BstNI subfamily 2 | PRB1 /// PRB2 | -2.275 | 2.9E-04 |
| --- | --- | --- | -2.279 | 8.4E-03 |
| Hs.148675 | hypothetical protein LOC283484 | LOC283484 | -2.309 | 5.9E-03 |
| Hs.478275 | Tumor necrosis factor (ligand) superfamily, member 10 /// Tumor necrosis factor (ligand) superfamily, member 10 | TNFSF10 | -2.318 | 2.2E-02 |
| Hs.191130 | Homo sapiens, clone IMAGE:5763888, mRNA | --- | -2.324 | 1.1E-02 |
| --- | --- | --- | -2.325 | 2.4E-05 |
| Hs.12865 | NSFL1 (p97) cofactor (p47) | NSFL1C | -2.333 | 2.3E-02 |
| Hs.339453 | PTX1 protein | PTX1 | -2.335 | 3.7E-02 |
| Hs.376639 | Homo sapiens, clone IMAGE:5209198, mRNA | --- | -2.336 | 9.7E-03 |
| --- | --- | --- | -2.346 | 4.0E-02 |
| Hs.405607 | phosphatase, orphan 1 | PHOSPHO1 | -2.363 | 6.5E-03 |
| Hs.419195 | hypothetical protein FLJ35954 | FLJ35954 | -2.371 | 4.2E-02 |
| Hs.101774 | Chromosome 20 open reading frame 23 | C20orf23 | -2.386 | 3.4E-02 |
| Hs.304947 | CDNA FLJ40647 fis, clone THYMU2017522 | --- | -2.388 | 2.0E-02 |
| Hs.106511 | Hypothetical protein LOC144997 | PCDH17 | -2.402 | 9.2E-03 |
| Hs.301431 | zinc finger protein 71 (Cos26) | ZNF71 | -2.405 | 3.9E-03 |
| Hs.460777 | Selectin ligand interactor cytoplasmic-1 | SLIC1 | -2.405 | 8.4E-05 |
| --- | --- | --- | -2.423 | 8.6E-04 |
| Hs.525256 | leukotriene B4 receptor | LTB4R | -2.425 | 1.8E-02 |
| Hs.403828 | FLJ43654 protein | FLJ43654 | -2.451 | 2.0E-02 |
| Hs.36915 | SMAD, mothers against DPP homolog 3 (Drosophila) | SMAD3 | -2.453 | 4.2E-02 |
| Hs.549076 | Zinc finger protein 91 (HPF7, HTF10) | ZNF91 | -2.463 | 5.4E-04 |
| Hs.257352 | apolipoprotein L, 6 | APOL6 | -2.469 | 4.8E-02 |
| Hs.306690 | Colon cancer clone PM102. | --- | -2.469 | 4.6E-02 |
| Hs.334587 | RNA binding protein with multiple splicing | RBPMS | -2.478 | 3.4E-03 |
| Hs.346575 | chromosome 19 open reading frame 26 | C19orf26 | -2.489 | 2.7E-03 |
| Hs.380169 | similar to tRNA synthetase class II | DKFZp727A071 | -2.490 | 8.4E-03 |
| Hs.553018 | Homo sapiens, clone IMAGE:4696935, mRNA | --- | -2.495 | 2.2E-02 |
| Hs.93564 | Homer homolog 2 (Drosophila) | HOMER2 | -2.509 | 3.0E-02 |
| Hs.272278 | cholinergic receptor, nicotinic, alpha polypeptide 9 | CHRNA9 | -2.511 | 9.7E-05 |
| Hs.75256 | regulator of G-protein signalling 1 | RGS1 | -2.516 | 4.6E-02 |
| Hs.316856 | Homo sapiens, clone IMAGE:4818734, mRNA | --- | -2.517 | 1.7E-02 |
| Hs.28872 | KIAA1946 | KIAA1946 | -2.521 | 1.6E-02 |
| Hs.531247 | hypothetical protein LOC285014 | LOC285014 | -2.540 | 4.0E-02 |
| Hs.24088 | hypothetical protein FLJ20125 | FLJ20125 | -2.554 | 2.6E-03 |
| Hs.181112 | mediator of RNA polymerase II transcription, subunit 4 homolog (yeast) | MED4 | -2.556 | 4.0E-04 |
| Hs.74137 | Transmembrane trafficking protein | TMP21 | -2.563 | 2.5E-03 |
| Hs.147554 | zinc finger and BTB domain containing 3 | ZBTB3 | -2.568 | 3.1E-02 |
| Hs.470654 | cell division cycle associated 7 | CDCA7 | -2.573 | 3.3E-04 |
| Hs.152414 | homeo box D13 | HOXD13 | -2.581 | 5.4E-03 |
| Hs.99836 | MRNA; cDNA DKFZp686B0610 (from clone DKFZp686B0610) | --- | -2.583 | 2.0E-02 |
| Hs.200632 | hypothetical gene supported by AY338954 | LOC440161 | -2.586 | 1.8E-02 |
| Hs.547277 | Transcribed locus, strongly similar to XP_524527.1 similar to regulatory erythroid kinase long form [Pan troglodytes] | --- | -2.600 | 1.3E-02 |
| Hs.72307 | Transcribed locus | --- | -2.605 | 2.0E-02 |
| Hs.499620 | gem (nuclear organelle) associated protein 4 | GEMIN4 | -2.610 | 9.6E-04 |
| Hs.538399 | Homo sapiens, clone IMAGE:4795493, mRNA, partial cds | --- | -2.616 | 7.1E-03 |
| Hs.431498 | forkhead box P1 | FOXP1 | -2.635 | 1.2E-02 |
| Hs.355944 | Transcribed locus, strongly similar to XP_511906.1 similar to KIAA0612 protein [Pan troglodytes] | --- | -2.654 | 2.8E-02 |
| --- | --- | --- | -2.656 | 2.9E-04 |
| Hs.519018 | SH3 domain protein D19 | EVE1 | -2.694 | 7.4E-03 |
| Hs.547593 | Homo sapiens, clone IMAGE:4551281, mRNA | --- | -2.694 | 5.1E-03 |
| Hs.402752 | TAF15 RNA polymerase II, TATA box binding protein (TBP)-associated factor, 68kDa | TAF15 | -2.700 | 6.1E-04 |
| Hs.49329 | CDNA clone IMAGE:5263455, partial cds | --- | -2.708 | 1.1E-03 |
| Hs.459952 | Stannin | SNN | -2.714 | 1.0E-04 |
| Hs.371249 | pleiotrophin (heparin binding growth factor 8, neurite growth-promoting factor 1) | PTN | -2.722 | 3.6E-02 |
| Hs.546441 | Polyhomeotic like 3 (Drosophila) | PHC3 | -2.735 | 8.4E-04 |
| Hs.550853 | Transcribed locus | --- | -2.736 | 1.5E-02 |
| Hs.119594 | citron (rho-interacting, serine/threonine kinase 21) | CIT | -2.737 | 4.1E-03 |
| Hs.408453 | Wilms tumor 1 | WT1 | -2.759 | 6.8E-03 |
| Hs.112783 | Androgen-induced 1 | AIG1 | -2.777 | 2.1E-02 |
| Hs.243564 | CD48 antigen (B-cell membrane protein) | CD48 | -2.812 | 1.2E-03 |
| Hs.50802 | hypothetical protein FLJ14712 | FLJ14712 | -2.842 | 9.1E-03 |
| Hs.131490 | Transcribed locus | --- | -2.857 | 4.4E-02 |
| Hs.503743 | Glutamate receptor, ionotrophic, AMPA 4 | GRIA4 | -2.881 | 1.9E-02 |
| Hs.517331 | Chromosome 21 open reading frame 2 | C21orf2 | -2.887 | 6.9E-03 |
| Hs.368226 | SRY (sex determining region Y)-box 6 | SOX6 | -2.892 | 3.1E-02 |
| Hs.44685 | ring finger protein 141 | RNF141 | -2.911 | 5.0E-04 |
| --- | --- | --- | -2.922 | 3.9E-02 |
| Hs.489786 | cystic fibrosis transmembrane conductance regulator, ATP-binding cassette (sub-family C, member 7) | CFTR | -2.926 | 2.5E-02 |
| Hs.3376 | TBC1 domain family, member 13 | TBC1D13 | -2.945 | 1.3E-04 |
| Hs.193370 | LIM domains containing 1 | LIMD1 | -2.958 | 1.4E-02 |
| Hs.463129 | KIAA0553 protein | KIAA0553 | -2.991 | 1.6E-02 |
| Hs.162025 | Transcribed locus | --- | -3.008 | 6.1E-03 |
| Hs.432419 | Hypothetical LOC541471 protein | --- | -3.048 | 1.5E-02 |
| Hs.170853 | Similar to MGC53446 protein | --- | -3.117 | 1.1E-03 |
| Hs.368538 | glutamate dehydrogenase 2 | GLUD2 | -3.118 | 1.6E-02 |
| Hs.441122 | Similar to leucine rich repeat containing 10 | --- | -3.136 | 1.8E-02 |
| Hs.355944 | Transcribed locus, strongly similar to XP_511906.1 similar to KIAA0612 protein [Pan troglodytes] | --- | -3.142 | 2.2E-04 |
| Hs.189084 | CDNA FLJ33564 fis, clone BRAMY2010135 | --- | -3.157 | 4.3E-03 |
| Hs.477537 | WD repeat domain 10 | WDR10 | -3.159 | 8.2E-03 |
| Hs.546481 | AHA1, activator of heat shock 90kDa protein ATPase homolog 2 (yeast) | AHSA2 | -3.177 | 8.8E-04 |
| Hs.497431 | Transcribed locus, strongly similar to XP_514603.1 similar to Eukaryotic translation initiation factor 6 (eIF-6) (B4 integrin interactor) (CAB) (p27(BBP)) (B(2)GCN homolog) (OK/SW-cl.27) [Pan troglodytes] | --- | -3.184 | 1.5E-03 |
| Hs.474536 | myotubularin related protein 3 | MTMR3 | -3.204 | 1.4E-02 |
| --- | hypothetical protein LOC338739 | LOC338739 | -3.217 | 5.2E-03 |
| Hs.106019 | protein phosphatase 1, regulatory subunit 10 | PPP1R10 | -3.226 | 2.6E-03 |
| Hs.434909 | Transcribed locus | --- | -3.229 | 2.9E-02 |
| Hs.24115 | chromosome 13 open reading frame 25 | C13orf25 | -3.241 | 6.9E-04 |
| Hs.531807 | Rho GTPase activating protein 25 | ARHGAP25 | -3.255 | 3.3E-06 |
| Hs.370336 | Transcribed locus, weakly similar to NP_775735.1 l(3)mbt-like 4 (Drosophila) [Homo sapiens] | --- | -3.255 | 2.4E-02 |
| Hs.130866 | CDNA FLJ30885 fis, clone FEBRA2004987 | --- | -3.278 | 1.0E-02 |
| Hs.371722 | HSPC049 protein | HSPC049 | -3.320 | 2.9E-03 |
| --- | --- | --- | -3.344 | 2.2E-02 |
| Hs.506670 | selectin P ligand | SELPLG | -3.366 | 1.8E-03 |
| Hs.279840 | zinc finger protein 222 | ZNF222 | -3.379 | 1.5E-03 |
| Hs.250821 | zinc finger protein 557 | ZNF557 | -3.681 | 2.1E-02 |
| Hs.445414 | CDNA FLJ41270 fis, clone BRAMY2036387 | --- | -3.698 | 6.7E-05 |
| Hs.549119 | jumonji domain containing 3 | JMJD3 | -3.701 | 1.0E-03 |
| Hs.549563 | CDNA clone IMAGE:30349460, partial cds | --- | -3.805 | 2.3E-03 |
| Hs.38218 | Transcribed locus | --- | -3.861 | 1.4E-03 |
| Hs.172982 | similar to expressed sequence AI593442 | LOC399947 | -3.902 | 1.0E-02 |
| Hs.258798 | chromosome 10 open reading frame 86 | C10orf86 | -4.129 | 4.6E-03 |
